# Chemical Proteomics Reveal the Inventory of Pyrroloquinoline Quinone Binding Proteins in Bacteria

**DOI:** 10.1021/jacs.6c03427

**Published:** 2026-04-02

**Authors:** Tao Wang, Rahel Mühlhofer, E Lei, Wei Ding, Andreas S. Klein, Cathleen Zeymer, Stephan A. Sieber

**Affiliations:** Center for Functional Protein Assemblies (CPA), Department of Bioscience, TUM School of Natural Sciences, 9184 Technical University of Munich (TUM), Ernst-Otto-Fischer-Straße 8 85748, Garching, Germany

## Abstract

Pyrroloquinoline quinone (**PQQ**) is a bacterial redox cofactor enabling enzyme catalysis in various sugar and alcohol dehydrogenases. However, its proposed additional role as a “longevity vitamin” lacks a clear molecular basis and is thus highly debated. Here, we applied chemical proteomics to identify previously unknown classes of **PQQ**-binding proteins. We designed and synthesized a structurally diverse suite of five **PQQ** probes equipped with a diazirine photo-cross-linker and an alkyne handle for target identification. The fidelity of the probes was first evaluated for two well-characterized bacterial **PQQ**-dependent enzymes, demonstrating not only probe binding but also the reconstitution of catalytic activity. We then commenced with proteome profiling of *Escherichia coli* and *Pseudomonas putida* cells and unraveled a distinct set of putative **PQQ**-binding proteins. Recombinant expression of selected hits, including several chaperones, validated **PQQ** binding. Notably, in some cases, **PQQ** even formed covalent adducts with selected lysine residues, for instance, in the AAA+ ATPase RuvB involved in DNA remodeling. Overall, our work highlights the utility of **PQQ** probes to further unravel the complement of cofactor-binding proteins in whole cells. It also provides a basis for future mechanistic studies of **PQQ** functions beyond redox catalysis.

## Graphical abstract

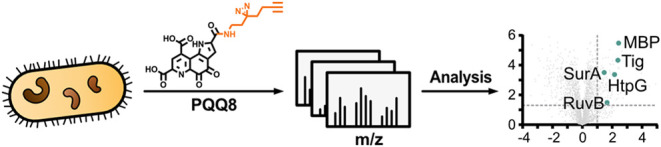

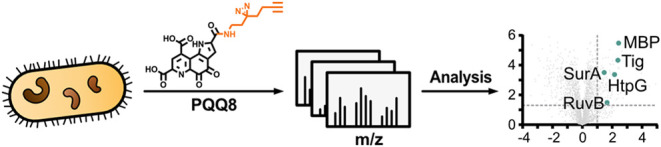

## Introduction

Cofactors play a crucial role in enzyme catalysis and thereby support essential cellular processes such as metabolism, energy supply, and detoxification.^1,2^ They comprise inorganic metal ions, as well as organic molecules often derived from vitamins or essential nutrients. While the first cofactors, including nicotinamide adenine dinucleotide (NAD^+^)^3^ and heme,^4^ were identified more than 100 years ago, one of the latest discoveries was pyrroloquinoline quinone (**PQQ**, Figure 1A),^5^ structurally characterized in the late 1970s.^6,7^ **PQQ**-dependent enzymes^8–13^ have been predominantly identified in bacteria, where **PQQ** complements nicotinamide and flavin-based redox cofactors and is utilized by periplasmic alcohol and sugar dehydrogenases. Most **PQQ**-producing bacteria are proteobacteria, which biosynthesize the cofactor from a ribosomally derived peptide.^13–15^ Other bacteria, such as *E. coli*,^16^ are unable to synthesize **PQQ**, but express proteins for **PQQ** uptake from the environment into their periplasm to ensure a sufficient cofactor supply.^17,18^

**Figure 1:**
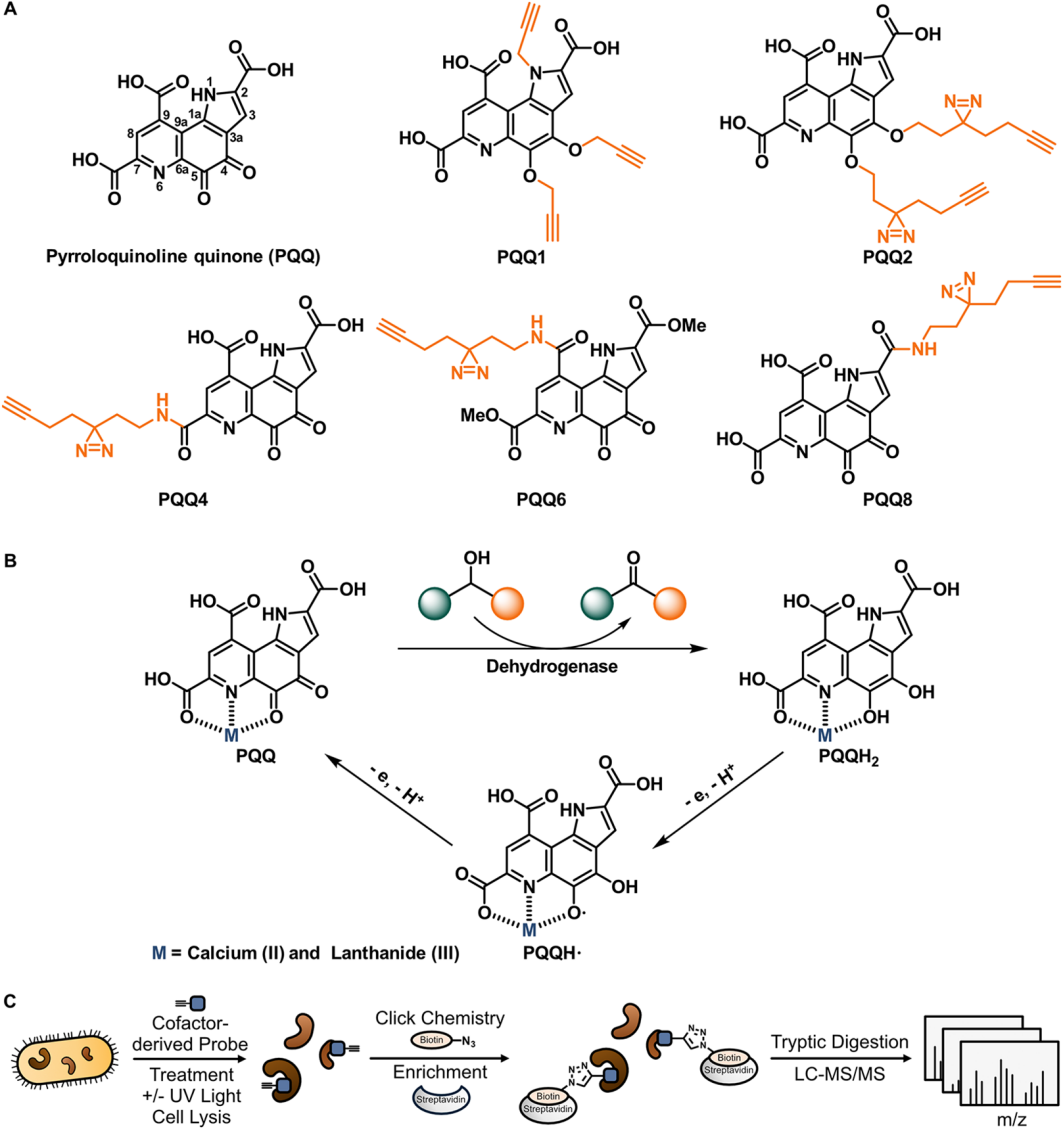
Redox cofactor **PQQ**: structure, function, and chemoproteomic profiling strategy. (A) Structures of **PQQ** and representative **PQQ**-derived probes for chemical proteomics. (B) Redox states of **PQQ** in the catalytic cycle of alcohol and sugar dehydrogenases. (C) Affinity-based protein profiling (AfBPP)^53^ workflow. Intact living cells are treated with cofactor-derived probes, followed by UV irradiation, cell lysis, click reaction with biotin azide, enrichment of cofactor-binding proteins on streptavidin beads, digestion by trypsin, and finally analysis by mass spectrometry.

Crystal structures of **PQQ** enzymes^19–21^ reveal a characteristic β-propeller fold, often also comprising a conserved disulfide bond above the cofactor binding site. Another hallmark of these structures is an essential metal ion,^22–24^ either Ca^2+^ or a trivalent lanthanide ion,^23,25,26^ which is coordinated by **PQQ** together with Asn, Asp, and Glu side chains or backbone carbonyl groups of the enzyme. In addition to its structural role for binding **PQQ** in the active site, the metal ion acts as a Lewis acid, enhancing the electrophilicity of **PQQ**’s C_5_ position. Two possible mechanisms of catalysis have been postulated,^27,28^ involving either hydride transfer or hemiketal formation at C_5_. After substrate oxidation, the fully reduced cofactor **PQQH**_2_ must be reoxidized to close the catalytic cycle (Figure 1B). This happens stepwise via the formation of the semiquinone radical **PQQH** ^•^.^29,30^ Recent structural and biochemical insights indicate that the conserved disulfide bond may assist in this process.^31^ Furthermore, the electrophilic C_5_ has been shown to engage in direct adduct formation with nucleophiles such as amines.^32–35^ This led us to hypothesize that **PQQ** may have more, so far unknown and potentially covalent, protein binding partners in the proteome.

Two **PQQ-**dependent enzymes are known in *E. coli*, the membrane-bound glucose dehydrogenase Gcd^36^ and the periplasmic soluble aldose sugar dehydrogenase YliI.^37^ While Gcd oxidizes glucose to gluconolactone to feed electrons into the respiratory chain, YliI exhibits a broader sugar substrate specificity.^17,37^ Another soluble and well-characterized quinoprotein is the alcohol dehydrogenase PedH from the **PQQ**-producing organism *P. putida*.^38^ This periplasmic enzyme is involved in metabolizing volatile alcohols,^39^ but has also been engineered to accept and oxidize larger substrates.^40^ Interestingly, its active site selectively coordinates a lanthanide instead of a calcium ion for catalytic activity.^38^ **PQQ**-dependent dehydrogenases, including YliI and PedH, have recently been repurposed as photoenzymes, promoting enantioselective radical cyclizations upon visible-light irradiation.^41^ This supports the hypothesis that **PQQ** may have further, yet undiscovered, functions in catalysis and beyond.

Despite the knowledge of **PQQ** biosynthesis, uptake, and redox enzyme function in bacteria, there is only limited understanding of the full inventory of proteins binding to or utilizing the **PQQ** cofactor.^42,43^ Without such a comprehensive and molecular-level basis, many of **PQQ**’s additionally proposed functions as radical scavenger and antioxidant, cellular stress mediator, or even “longevity vitamin” remain enigmatic and thus highly debated.^44–46^ We recently introduced chemoproteomic cofactor profiling as a method to mine cellular proteomes for cofactor-dependent enzymes (Figure 1C). The cofactors pyridoxal phosphate,^47–49^ heme,^50,51^ and lipoic acid^52^ were modified with an alkyne handle, incubated with living cells, followed by cell lysis. Proteomes were clicked to biotin azide for the enrichment of labeled proteins on avidin beads, followed by tryptic digestion. Mass spectrometric analysis (MS) revealed known members of the respective cofactor-dependent enzyme classes but also several proteins of uncharacterized function. In-depth studies of these enigmatic enzymes led to new insights into their cellular function. Here, we envision a similar approach for **PQQ**.

We synthesized a set of **PQQ** probes and benchmarked them using YliI and PedH. Several probes showed binding to these known **PQQ**-dependent enzymes, and some even reconstituted their catalysis, highlighting the success of the complementary probe design. Labeling in intact *E. coli* as well as *P. putida* cells followed by MS-based target identification revealed a panel of putative quinoproteins. Overall, our results suggest that the quinoproteome is larger than was previously anticipated.

## Results

### Design and Synthesis of **PQQ** Probes

An important starting point for this project was the development of different **PQQ** probes to address a variety of structurally diverse **PQQ-**binding pockets. Probe design was guided by synthetic accessibility and the known binding modes of the cofactor based on crystal structures of dehydrogenases,^24^ which allowed us to identify suitable sites for the incorporation of a chemical tag. Based on the crucial contacts of **PQQ** with the enzyme pocket via its carboxylic acid groups (at position C_2_ and C_9_) and the N_6_ nitrogen, carbonyl C_5_, and carboxylate at C_7_ being essential for metal coordination, the selection of anchoring points was not trivial (Figure 1A). Double as well as triple modifications were realized with the diazirine alkyne (**PQQ2**) or the pure alkyne (**PQQ1**), respectively. In addition, to account for binding to yet unknown quinoproteins with potentially different interactions with **PQQ**, we systematically varied the anchoring points across the molecule. These include the introduction of a minimal diazirine alkyne tag via condensation of carboxylic acid at C_2_ (**PQQ8**), C_7_ (**PQQ4**), as well as C_9_ (**PQQ6**, with additional methyl esters at C_2_ and C_9_).

Our synthetic strategy toward the desired probes was based on the direct decoration of the **PQQ** skeleton within a few steps (instead of de novo **PQQ** probe synthesis starting from feed stocks), making our synthesis more efficient (Scheme 1 and Scheme S1). The synthesis started by direct esterification of commercially available **PQQ** with dimethyl sulfate to afford the universal intermediate, **PQQ** trimethyl ester **1**, up to a 2 g scale in 77% yield. This triester **1** was reduced by phenyl hydrazine to almost quantitatively yield ester **PQQH**_2_ **2**. Then **2** was next alkylated with propargyl bromide or iodo diazirine-alkyne **3** and hydrolyzed with LiOH to obtain trialkynylated **PQQ1** in 8% yield and C_4_, C_5_-photo-cross-linker-functionalized **PQQ2** in 10% yield, respectively. The C_7_-photo-cross-linker **PQQ4** was obtained by selective hydrolysis of **PQQ** trimethyl ester **1** with TFA, quantitatively yielding **PQQ-C**_7_ **-COOH 4**, which was conjugated with diazirine-alkyne amine **5**. Subsequent hydrolysis with LiOH generated C_7_-photo-cross-linker **PQQ4** in 4% yield. The C_9_-photo-cross-linker **PQQ6** was designed and synthesized based on the same strategy: the key intermediate **PQQ-C**_9_ **-COOH 6** was acquired via hydrolysis of **PQQ** trimethyl ester **1** with Cs_2_CO_3_ in 98% yield and selectively esterified with concentrated sulfuric acid in MeOH to deliver **PQQ-C**_9_ **-COOH 7** in 94% yield, followed by conjugation of the intermediate **7** with diazirine-alkyne amine **5** delivering **PQQ6** in 8% yield. Unfortunately, further hydrolysis of **PQQ6** via different bases to the free acid **PQQ6-COOH** failed. As the direct decoration of C_2_-photo-cross-linker **PQQ8** turned out to be challenging, we utilized a build-decorate strategy instead: the indole acid **9**, obtained from the hydrolysis of indole ester **8** (almost quantitatively, up to 4.5 g), was reacted with ketene ester **10** via Doebner–von Miller type annulation^54,55^ to afford the tetrahydroquinoline intermediate **11**. This intermediate **11** was dehydrated with HCl and aromatized with air as the oxidant and Cu­(OAc)_2_ as the catalyst to construct the skeleton of **PQQ** to achieve phenyl ether **12** in 68% yield. Then **12** was oxidized by CAN to deliver **PQQ-C**_2_ **-COOH 13** in 58% yield. Finally, The C_2_-photo-cross-linker **PQQ8** was acquired by the conjugation of **13** with diazirine-alkyne amine **5** following hydrolysis with LiOH in 3% yield.

**Scheme 1:**
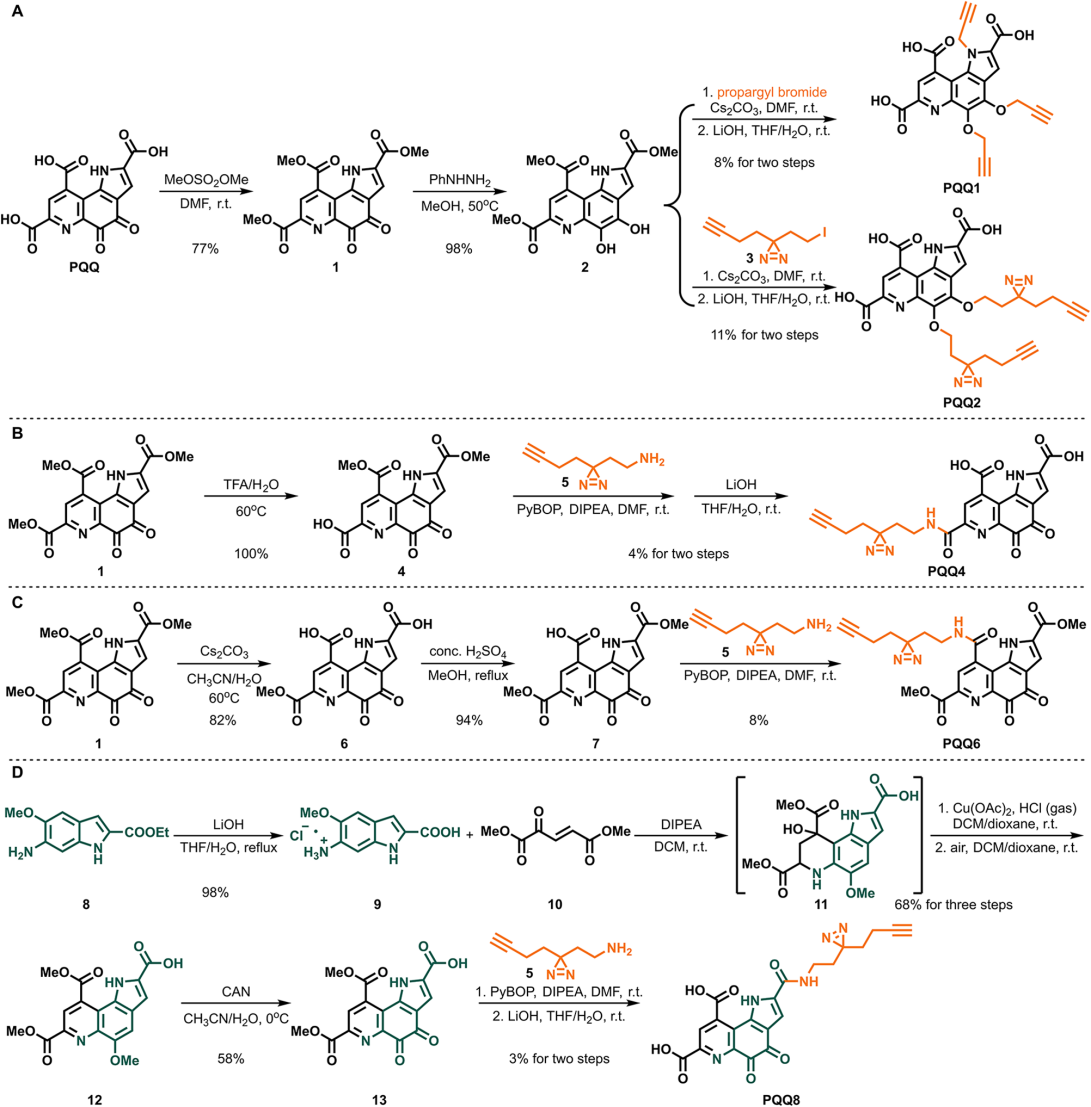
Synthesis of PQQ Probes^a^

a For details on Regents and Conditions, see the Supporting Information.

### A Subset of Probes Binds Known **PQQ**-Dependent Dehydrogenases and Reconstitutes Their Activity

To evaluate the binding of the diverse cofactor probes to known **PQQ**-dependent dehydrogenases, we recombinantly expressed and purified PedH and YliI as two representative and well-characterized examples and performed UV/vis absorption spectroscopy. Free **PQQ** exhibits characteristic absorption bands at 350 and 500 nm^41^ (Figure 2A/D), while binding to YliI and PedH is associated with spectral shifts (new maximum at 430 nm) that cause a color change from orange to yellow. As the different probes have chemical modifications in various positions, they exhibit distinct absorption spectra. We incubated both proteins with all probes and then removed unbound molecules by buffer exchange. If the spectroscopic signature of the respective probe was still clearly present in the protein sample, the probe was considered binding-competent. This was the case for all probes in the case of PedH and all probes except for **PQQ1** and **PQQ2** for YliI (Figures 2B,E and S1).

**Figure 2:**
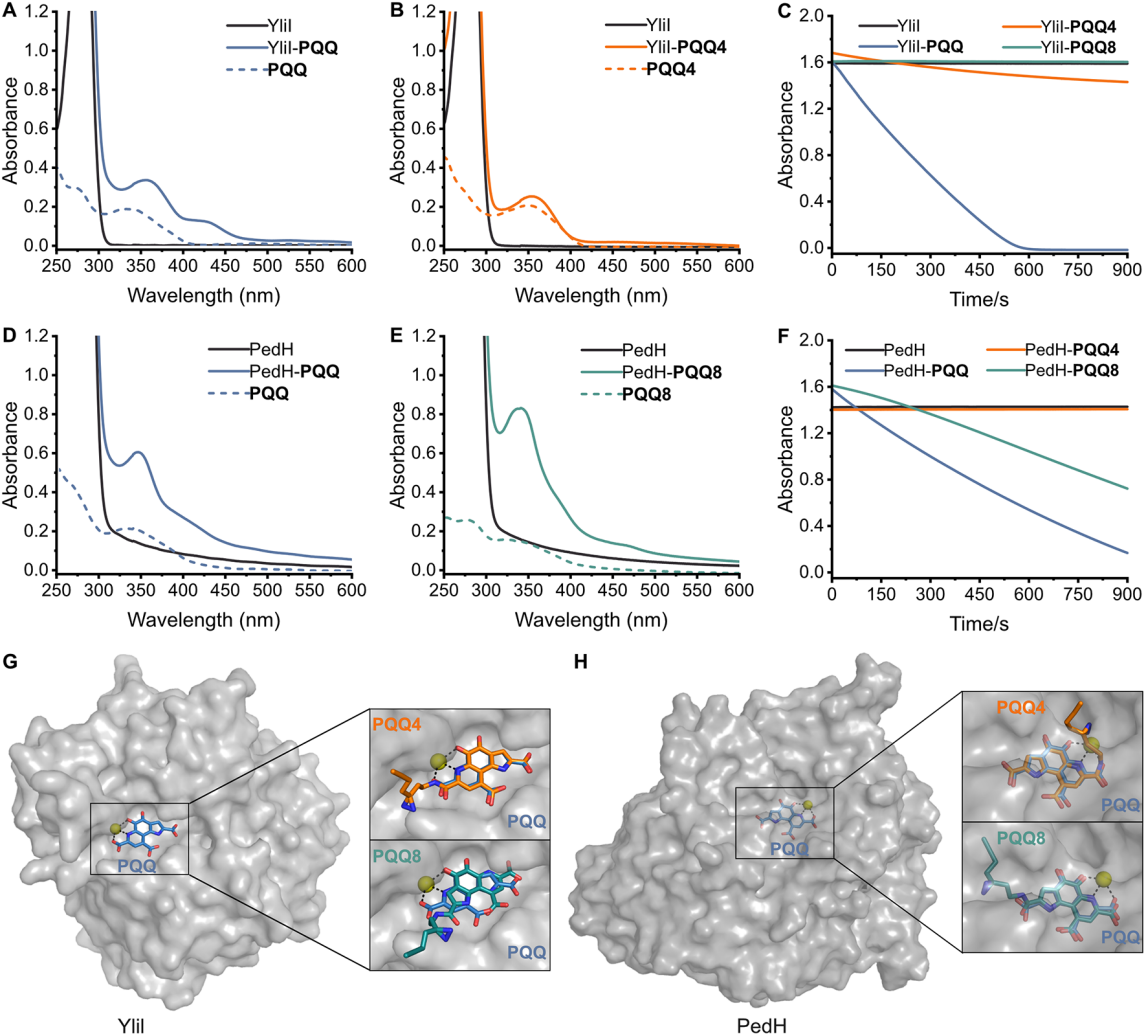
**PQQ** probe binding and catalytic activity of known **PQQ**-dependent dehydrogenases. (A) Absorption spectra of cofactor-free YliI, YliI reconstituted with **PQQ**, and free **PQQ**. (B) Absorption spectra of cofactor-free YliI, YliI reconstituted with **PQQ4**, and free **PQQ4**. (C) Glucose dehydrogenase activity of YliI reconstituted with **PQQ** or **PQQ** probes was measured in a coupled colorimetric assay using 100 nM reconstituted enzyme and 1.25 M glucose. (D) Absorption spectra of cofactor-free PedH, PedH reconstituted with **PQQ**, and free **PQQ**. (E) Absorption spectra of cofactor-free PedH, PedH reconstituted with **PQQ8**, and free **PQQ8**. (F) Ethanol dehydrogenase activity of PedH reconstituted with **PQQ** or **PQQ** probes was measured in a coupled colorimetric assay using 100 nM reconstituted enzyme and 50 mM ethanol. (G) Computational model of **PQQ4** or **PQQ8** binding in the active site of YliI in comparison to the native **PQQ** cofactor. (H) Computational model of **PQQ4** or **PQQ8** binding in the active site of PedH in comparison to the native **PQQ** cofactor.

Importantly, the reconstitution of YliI with **PQQ4** and PedH with **PQQ8** resulted in catalytically active enzymes, as measured in a coupled colorimetric assay (Figure 2C,F). Glucose oxidation by **PQQ4**-bound YliI was significantly reduced, while ethanol oxidation by **PQQ8**-bound PedH was almost at the native level. Both probes have unmodified quinone systems and are, thus, in principle capable of catalysis. To rationalize the complementary probe preference of YliI and PedH, we performed computational modeling using the Boltz-1 ^56^ and Boltz-2 ^57^ structure prediction tools and carefully compared the predicted probe binding to the native **PQQ-**binding mode. In contrast to classic docking, the AI-based tool allows for some flexibility and slight adaptation of the protein structure to the new ligands. Both **PQQ4** and **PQQ8** fit into the open and accessible active site of YliI, but only **PQQ4** binds in the catalytically productive orientation of the native **PQQ** cofactor, which enables catalysis (Figure 2G). Also for PedH, both **PQQ4** and **PQQ8** fit into the active site and even bind in orientations similar to the native **PQQ**. However, in our model, the click handle side-chain of **PQQ4** blocks the relatively narrow substrate entry tunnel leading toward the buried active site of PedH, while **PQQ8**’s click handle can be accommodated in a pocket inside the protein, thus allowing the substrate to enter (Figure 2H).

### Labeling of **PQQ**-Dependent Enzymes YliI and PedH in Complex Proteomes

In a next step, we tested if recombinant PedH and YliI can be labeled by the **PQQ** probes in the background of a complex proteome. For this, we spiked the purified cofactor-free proteins in *E. coli* K-12 lysate and added the **PQQ** probes at various concentrations for 1 h at room temperature under UV treatment. Labeled proteomes were clicked to rhodamine azide and visualized via SDS-PAGE followed by fluorescent scanning. Varying the concentration of spiked proteins revealed a sensitive detection of proteins down to 1 μM, which represents a suitable starting point for the detection of endogenous **PQQ**-binding proteins (Figures S2A,B and S3A,B). In addition, a **PQQ4** concentration of 50 μM seemed optimal for the saturated labeling of both proteins (Figures S2C,D and S3C,D). Among the tested probes, **PQQ2**, **PQQ4**, and **PQQ8** turned out as best binders for YliI and PedH, which is in line with the UV-shift assays (Figure S1). Of note, while some probes (**PQQ2** and **PQQ6**) show a strong labeling dependence on photo-cross-linking via UV-irradiation, **PQQ1** (no diazirine), **PQQ4**, and **PQQ8** seem to bear an intrinsic, photo-cross-linking-independent protein reactivity (Figure 3A,B). Probe binding was outcompeted by the addition of a 20-fold molar excess of native **PQQ** demonstrating that the **PQQ** probes target the same binding pocket (Figures 3C,D, S2F and S3F). Overall, these results highlight the importance of synthesizing a chemically diverse panel of probes to maximize the enzyme coverage.

**Figure 3:**
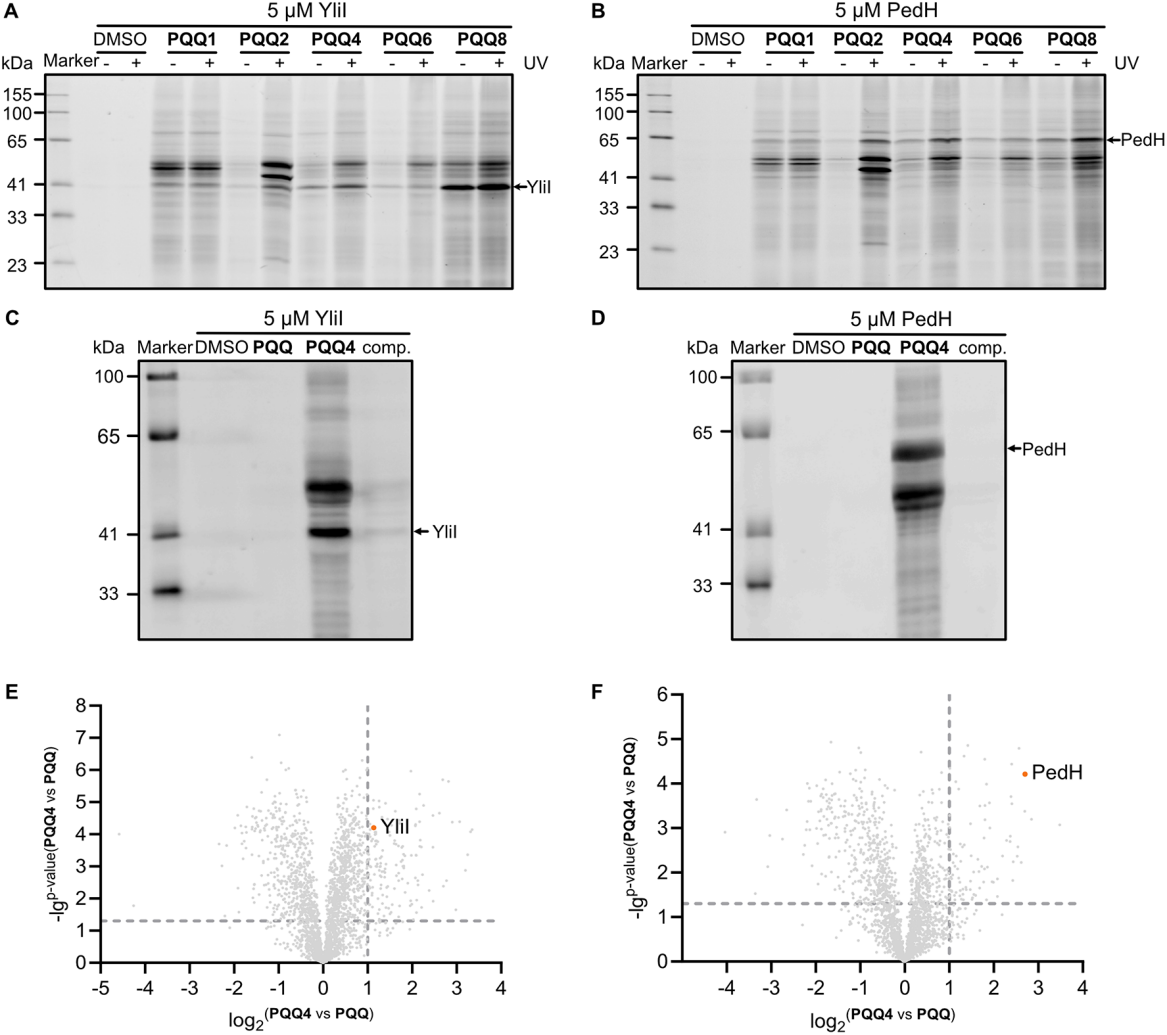
Labeling and enrichment of **PQQ**-dependent proteins by **PQQ** probes analyzed with SDS-PAGE and proteomics. (A) SDS-PAGE of *E. coli* K-12 lysate, with 5 μM spiked YliI, labeled by 50 μM **PQQ** probes compared to DMSO. (B) SDS-PAGE of *E. coli* K-12 lysate, with 5 μM spiked PedH, labeled by 50 μM **PQQ** probes compared to DMSO. (C) SDS-PAGE of *E. coli* K-12 lysate, with 5 μM spiked YliI, labeled by 50 μM **PQQ4** as well as competition with **PQQ** (50 μM **PQQ4** + 1000 μM **PQQ**) compared to DMSO and 50 μM **PQQ**. (D) SDS-PAGE, as described in Figure 3C, with 5 μM spiked PedH. (E) Volcano plot of *E. coli* BL21 (Tuner_YliI) treated with 50 μM **PQQ4** compared to 50 μM **PQQ**. The experiment was conducted in 4 biological replicates. The vertical and horizontal dashed lines represent a log_2_-fold change of 1 and a −log_10_ p-value of 1.3, respectively. (F) Volcano plot of *E. coli* BL21 (Tuner_PedH) treated with 50 μM **PQQ4** compared to 50 μM **PQQ**. The experimental details and cutoff criteria for volcano plot are shown in Figure 3E.

With validated **PQQ** tools at hand, we aimed to decipher the full complement of targets in *E. coli* by quantitative LC–MS/MS. *E. coli* expresses only two known **PQQ**-dependent dehydrogenases, Gcd and YliI, of which the latter is typically of low abundance.^37,58,59^ Given this limited number of expected endogenous hits, we first performed positive controls with two *E. coli* strains overexpressing YliI and PedH (Tuner_YliI and Tuner_PedH). Due to the leaky vector system, IPTG was not needed to induce protein overexpression in Tuner_YliI/Tuner_PedH cells for in situ labeling, and comparable amounts of both enzymes were expressed (Figure S4). *E. coli* cells were incubated with 50 μM **PQQ2** and **PQQ4**, UV-irradiated, lysed and clicked to biotin azide (for SDS-PAGE, see Figure S5A–E). Enrichment of labeled proteins on avidin beads followed by tryptic digest yielded peptides that were analyzed by LC–MS/MS (see Figure 1C for the overall workflow). Corresponding volcano plots depict the enrichment of proteins upon probe binding compared to **PQQ** (to account for any cellular response leading to a change in protein expression) as well as the significance. Proteins are considered hits if they exhibit a *p*-value < 0.01, *q*-value < 0.02, *t*-value > 4, and log_2_-fold enrichment of > 1. Satisfyingly, both YliI and PedH were detected as significantly enriched in the corresponding expression strains (Figures 3E,F, S5C,F and Table S13) and their peptide abundance correlated with probe concentration as well as the presence of the expression plasmid (profile plot in Figure S5G,H).

### Identification of **PQQ**-Binding Proteins in Living Cells

We commenced with the identification of **PQQ**-binding proteins in wild-type *E. coli* K-12. To maximize the coverage of enriched proteins with the probes, 4 h of starvation of *E. coli* K-12 cells in minimal medium was implemented before in situ labeling (Figure S6A). Concentration-dependent labeling of all probes with intact *E. coli* cells, followed by ±UV-irradiation, lysis, click chemistry to rhodamine azide, and visualization of fluorescent SDS-PAGE, revealed the labeling of several proteins throughout the proteome, indicating a larger number of **PQQ**-binding proteins as anticipated (Figures 4A and S6B). A concentration of 50 μM probe was sufficient to achieve saturated labeling and was thus subsequently selected for quantitative enrichment (Figure S6B). Based on this result, we selected probes **PQQ2**, **PQQ4**, **PQQ6**, and **PQQ8** (from here on referred to as **PQQ** probes), showing the most intense and comprehensive labeling, for further quantitative profiling. After treatment of intact *E. coli* cells grown in minimal medium with 50 μM probes (for 20/100 μM **PQQ4** labeling, see Figure S8E,F), the cells were processed for LC–MS/MS detection as described above. As a control, we treated cells with native **PQQ** to account for any cellular response leading to a change in protein expression. To determine the extent of direct protein binding without UV-photo-cross-linking, **PQQ** probes were investigated ±UV-treatment. In general, comparison of probe-treated cells in the presence and absence of UV light showed a much higher enrichment upon irradiation, demonstrating the need of photoprobes to induce covalent binding (Figures 4B,E, S7 and S8).

**Figure 4:**
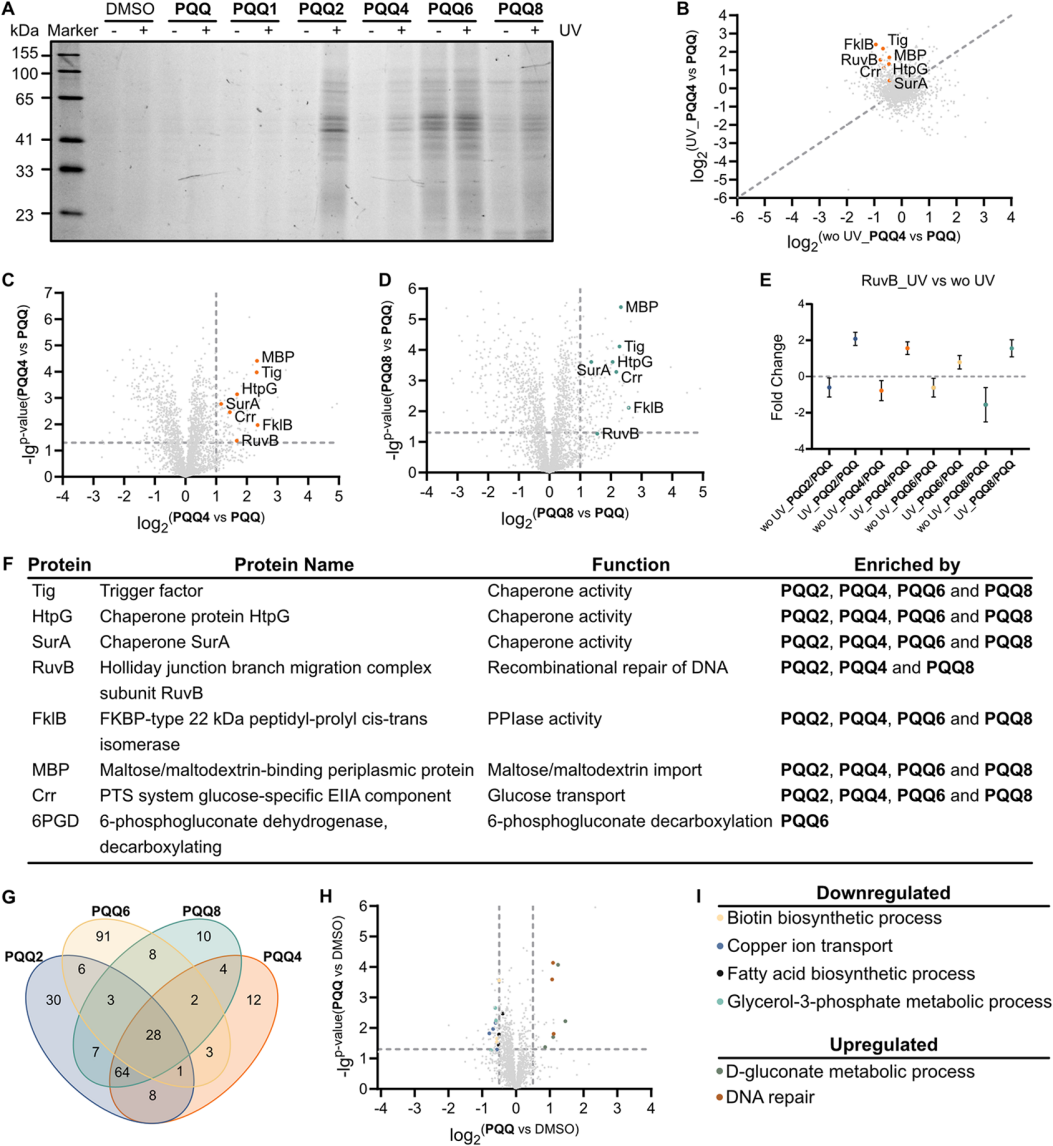
Target identification by chemical proteomics in *E. coli* K-12 cells. (A) SDS-PAGE of *E. coli* K-12 in situ labeled by 50 μM **PQQ** probes with ±UV treatment compared to DMSO. (B) Scatter plot of *E. coli* K-12 treated with 50 μM **PQQ4** with ±UV treatment compared to 50 μM **PQQ**. Both experiments were conducted in 3 biological replicates. Proteins above the dashed line exhibit higher enrichment upon UV-irradiation, which is the case for all significant hits with *p*-value < 0.05 and *q*-value < 0.05. (C) Volcano plot of *E. coli* K-12 treated with 50 μM **PQQ4** compared to 50 μM **PQQ**. The experiment was conducted in 3 biological replicates The vertical and horizontal dash lines represent a log_2_-fold change of 1 and a −log_10_ *p*-value of 1.3, respectively. (D) Volcano plot of *E. coli* K-12 treated with 50 μM **PQQ8** compared to 50 μM **PQQ**. The experimental details and cutoff criteria for volcano plot are shown in Figure 4C. (E) Summary data plot of RuvB treated with different **PQQ** probes under ±UV conditions. The experiment was conducted in 3 biological replicates. The horizontal dashed line represents a log_2_-fold change of 0 and the data represents standard error of mean (SEM) of averaged triplicates of *n* = 3 biologically independent experiments. (F) Table of representative *E. coli* K-12 proteins found in all AfBPP experiments, including 4 chaperone proteins (Tig, HtpG, SurA, and FklB), DNA repair protein RuvB and 3 sugar-related proteins (Crr, MBP, and 6PGD). (G) Venn diagram of enriched *E. coli* K-12 hits by different **PQQ** probes. Enriched proteins with log_2_-fold change > 1, *p*-value < 0.05, and *q*-value < 0.05 are shown. (H) Volcano plot of *E. coli* K-12 full proteome treated with 50 μM **PQQ** compared to DMSO. The experiment was conducted in 4 biological replicates. The vertical and horizontal dash lines represent a log_2_-fold change of ±0.5 and a −log_10_ *p*-value of 1.3, respectively. Colored dots (log_2_-fold change > 0.5 or < −0.5, *p*-value < 0.05, *t*-value > 2 or < −2) show functional upregulated and downregulated proteins. (I) Table of up-/down-regulated proteins from *E. coli* K-12 full proteome. The related gene ontology of biological process was analyzed with String database with “Group Similarity” > = 0.8 and high confidence > = 0.7.

Overall, 277 proteins met the cutoff criteria (log_2_-fold > 1, *p*-value < 0.05, *q*-value < 0.02, and *t*-value > 2) and 28 were consistently found with all four probes upon UV irradiation (Figures 4C,D and S8G,H). The distribution of hit proteins across all probes emphasizes that their diverse structural composition is needed for comprehensive profiling (Figure 4F,G). A closer inspection of the 28 hit proteins consistently enriched by all four probes revealed several molecular chaperones with either peptidyl-prolyl isomerization activity or ATPase activity, such as trigger factor (Tig), SurA, FklB, and the bacterial Hsp90 analogue HtpG, respectively. In addition, RuvB, a AAA+ ATPase and subunit of the Holliday junction branch migration complex involved in DNA remodeling, was also enriched by all **PQQ** probes except **PQQ6**. Moreover, several proteins related to sugar metabolism pathways were enriched, e.g., maltose-binding protein (MBP), PTS system glucose-specific EIIA component (Crr), and 6-phosphogluconate dehydrogenase (decarboxylating, 6PGD), see Figure 4F. In situ labeling with **PQQ4** and competition with native **PQQ** in *E. coli* K-12 revealed that a large panel of proteins were competed by native **PQQ**, such as Tig, SurA, FklB, HtpG, RuvB, and MBP (Figure S9). The membrane-associated **PQQ**-dependent dehydrogenase Gcd was enriched (fold change > 1.5, *p*-value < 0.01, *q*-value < 0.01, and *t*-value > 5) in wild-type *E. coli* K-12 solely under rich conditions without starvation (grown in LB medium, Figure S10 and Table S16). Full proteome comparison of **PQQ**-treated and untreated *E. coli* cells revealed upregulation of proteins involved in glucose metabolism as well as DNA repair and downregulation of proteins involved in biotin, glycerolphospholipid, fatty acid metabolism, and copper/iron ion transport (Figures 4H,I, S12 and S13).

The same workflow was performed with *P. putida* KT2440, a strain capable of producing **PQQ**. *P. putida* KT2440 was labeled with **PQQ** probes in mineral salts medium for 1 h, which revealed significantly enriched proteins (fold change > 1, *p*-value < 0.05, *q*-value < 0.05, and *t*-value > 2.5) (Figures 5A,B and S14). In general, 285 proteins were significantly enriched, and 11 proteins, especially chaperones (Tig, HtpG, SurA, FklB-I, and PpiD) and AAA+ ATPase involved in DNA remodeling (RuvB), were consistently enriched by all four probes (Figure 5C,D). Moreover, the outer membrane protein assembly factor BamB,^60^ a nonessential subunit of the Bam complex and previously annotated as a **PQQ** binder due to its β-propeller fold, was identified (Figure 5A,B). Furthermore, the pyrroloquinoline quinone synthase (PqqC)^13,14,61^ responsible for the last step in **PQQ** biosynthesis was also enriched (Figure 5A). PqqC catalyzes the oxidation and cyclization of a **PQQ** precursor to generate **PQQ**, which is subsequently released and transported to the periplasmic, where it fulfills its function as a redox cofactor. Full proteome analysis of **PQQ**-treated cells revealed a complementary picture compared to *E. coli* with downregulated proteins in tyrosine, phenylalanine, aromatic compounds (fluorobenzoate), and organic substances (valine, leucine, and isoleucine) catabolic processes (Figures S15 and S16). Overall, 23 proteins were mutually enriched by *E. coli* K-12 and *P. putida* KT2440, including the molecular chaperones trigger factor, HtpG, SurA, FklB, and the AAA+ ATPase RuvB involved in DNA remodeling (Figure 5D,E). This conserved set of targets across species was selected for in-depth validation.

**Figure 5:**
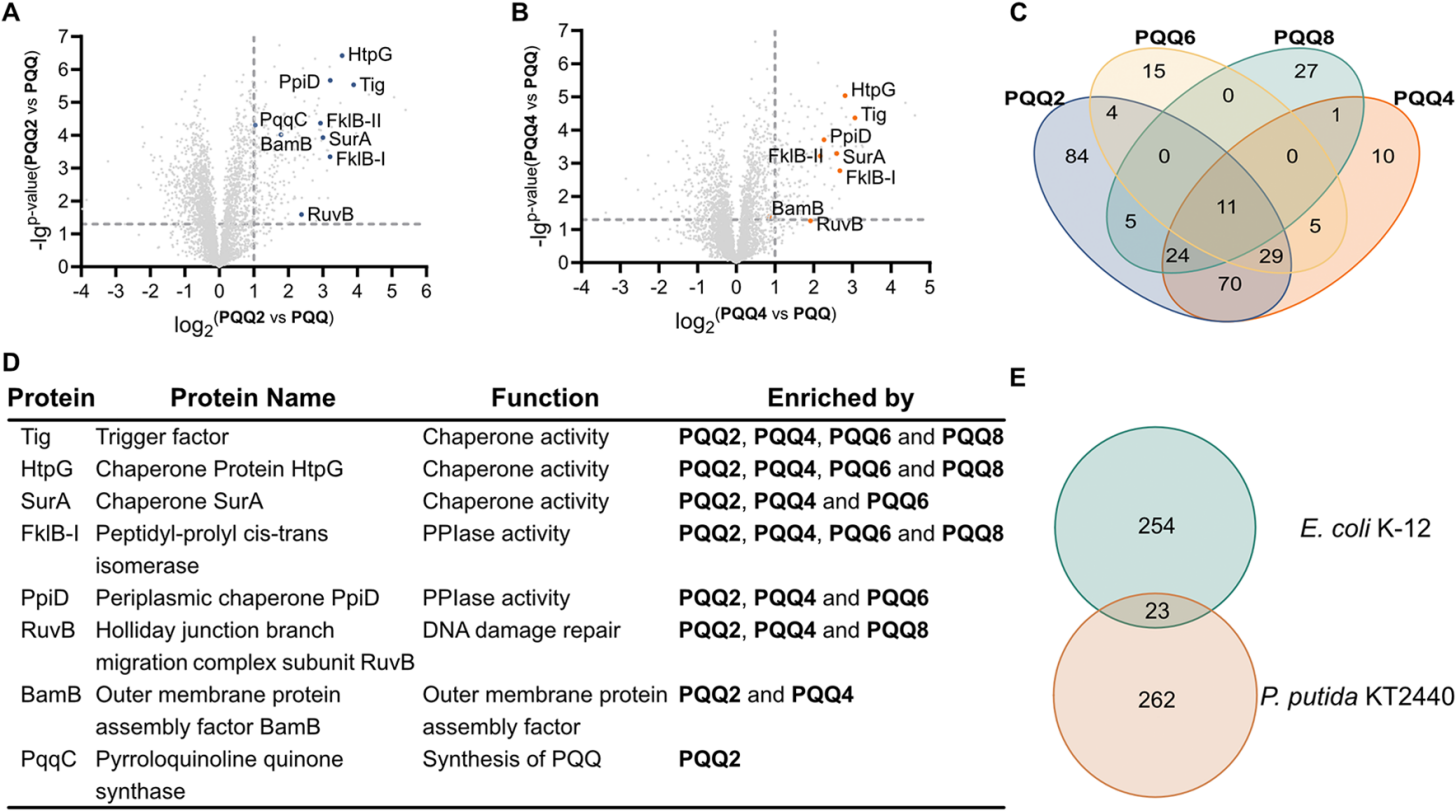
Target identification by chemical proteomics in *P. putida* KT2440 cells. (A) Volcano plot of *P. putida* KT2440 treated with 50 μM **PQQ2** compared to 50 μM **PQQ**. The experiment was conducted in 4 biological replicates. The vertical and horizontal dash lines represent a log_2_-fold change of 1 and a −log_10_ *p*-value of 1.3, respectively. (B) Volcano plot of *P. putida* KT2440 treated with 50 μM **PQQ4** compared to 50 μM **PQQ**. The experimental details and cutoff criteria for volcano plot are shown in Figure 5A. (C) Venn diagram of enriched *P. putida* KT2440 hits by different **PQQ** probes. The hits represent the enriched proteins in the volcano plot with log_2_-fold change > 1, *p*-value < 0.05, and *q*-value < 0.05. (D) Table of representative *P. putida* KT2440 proteins in all AfBPP experiments, including 5 chaperone proteins (Tig, HtpG, SurA, FklB, and PpiD), DNA repair protein RuvB, BamB as outer membrane protein assembly factor, and pyrroloquinoline quinone synthase PqqC. (E) Venn diagram of **PQQ** probe hits in *E. coli* K-12 and *P. putida* KT2440 (log_2_-fold change > 1, *p*-value < 0.05, and *q*-value < 0.05).

Intrigued by the significant extent of **PQQ-**binding protein and the previous discovery of lactate dehydrogenase (LDH) as a **PQQ-**binding protein in mouse pull down studies,^62^ we gained initial insights into proteins bound by **PQQ** in human cells. HepG2 cells were treated with 50 μM **PQQ2** and **PQQ4**, followed by the standard proteomics workflow. 314 proteins were significantly enriched, including glycosyltransferase family members and chaperones (Figure S17). Of note, the enrichment of l-lactate dehydrogenase A-like 6B (LDH6B) further validates previous studies describing it as **PQQ** binder. These results give a first indication of a pronounced and diverse proteome binding of **PQQ**, providing a basis for future studies on human quinone-binding proteins and the enigmatic impact of the cofactor on health and disease.

### PQQ Binds Covalently to Selected Proteins

For further in-depth validation on putative **PQQ** binding, we selected the most common hits, Tig, HtpG, FklB, SurA, PpiD, MBP, and RuvB overlapping between both bacterial strains. The proteins were recombinantly expressed in *E. coli* BL21 and purified in the absence of **PQQ** (Figures S18 and S19). We spiked the recombinant proteins in *E. coli* K-12 lysate and performed labeling with **PQQ4** with and without the addition of a 20-fold molar excess of **PQQ** for 1 h of incubation at room temperature, followed by UV treatment. The proteomes were clicked to rhodamine azide and visualized via SDS-PAGE. All recombinant proteins were labeled with **PQQ4**, and this labeling was outcompeted with an excess of free **PQQ** (Figures 6A–C,J–L and S20). Furthermore, the addition of the cofactor revealed characteristic spectroscopic shifts comparable to those of YliI and PedH, indeed confirming **PQQ** binding (Figures 6D–F, M–O and S21A). As a negative control, we included BSA and Gsk as non-**PQQ-**binding proteins (Figure S21B,C) and did not observe a signal change, which validates the selected hits listed above as true positives.

**Figure 6:**
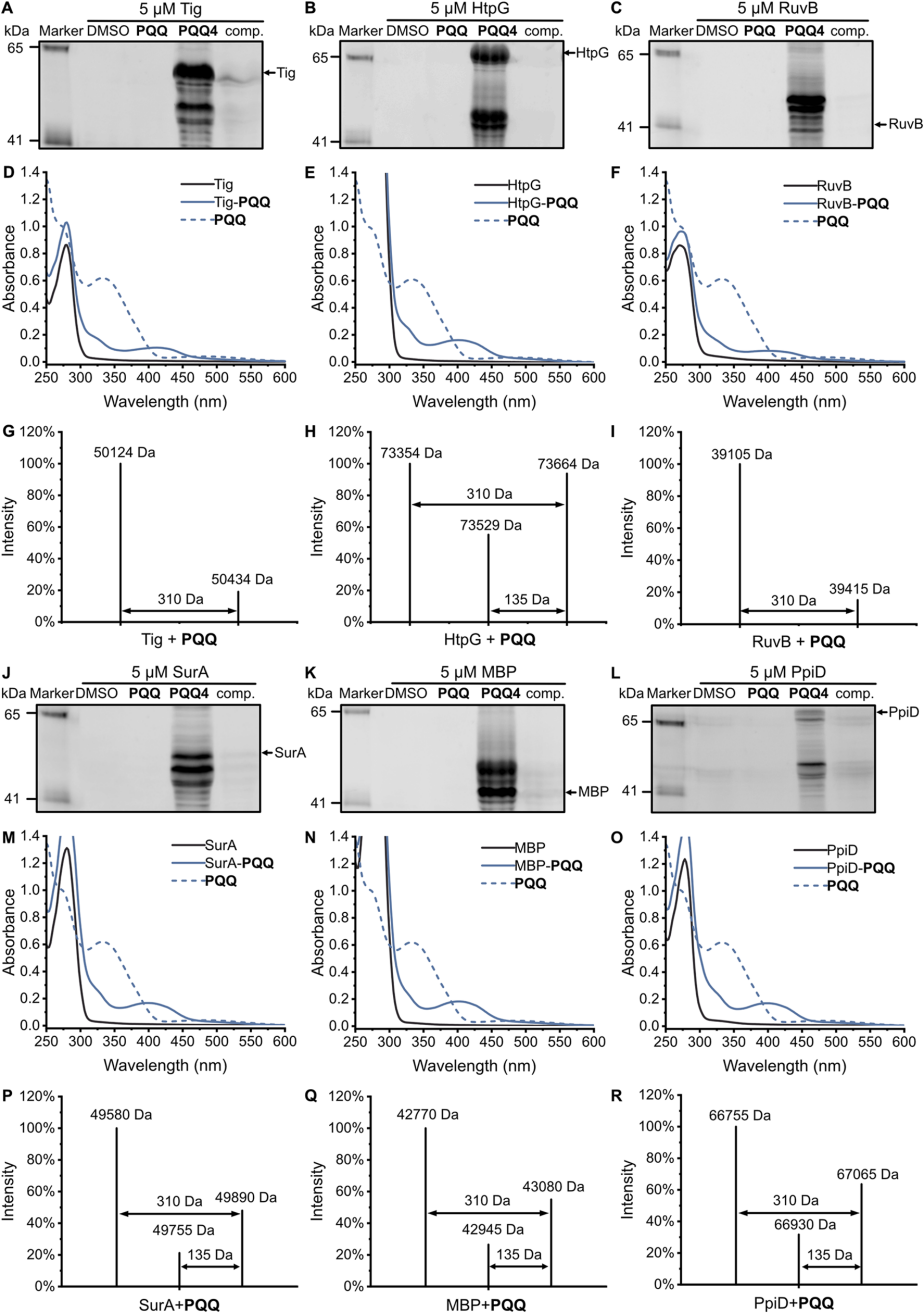
Identification of covalent **PQQ-**binding proteins. (A) SDS-PAGE of *E. coli* K-12 lysate, with 5 μM spiked Tig, labeled by 50 μM **PQQ4** as well as competition with **PQQ** (50 μM **PQQ4** + 1000 μM **PQQ**) compared to DMSO and 50 μM **PQQ**. (B) SDS-PAGE, as described in Figure 6A, with 5 μM spiked HtpG. (C) SDS-PAGE, as described in Figure 6A, with 5 μM spiked RuvB. (D) Absorption spectra of 100 μM Tig, 100 μM Tig-**PQQ**, and 100 μM **PQQ**. (E) Absorption spectra of 100 μM HtpG, 100 μM HtpG-**PQQ**, and 100 μM **PQQ**. (F) Absorption spectra of 100 μM RuvB, 100 μM RuvB-**PQQ**, and 100 μM **PQQ**. (G) IPMS of Tig treated with 3-fold molar excess **PQQ**. Here, the intensity indicates the deconvoluted mass intensity by UniDec,^70^ same applies for Figure 6H,I,P–R. (H) IPMS of HtpG treated with 3-fold molar excess **PQQ**. (I) IPMS of RuvB treated with 3-fold molar excess **PQQ**. (J) SDS-PAGE, as described in Figure 6A, with 5 μM spiked SurA. (K) SDS-PAGE, as described in Figure 6A, with 5 μM spiked MBP. (L) SDS-PAGE, as described in Figure 6A, with 5 μM spiked PpiD. (M) Absorption spectra of 100 μM SurA, 100 μM SurA-**PQQ**, and 100 μM **PQQ**. (N) Absorption spectra of 100 μM MBP, 100 μM MBP-**PQQ**, and 100 μM **PQQ**. (O) Absorption spectra of 100 μM PpiD, 100 μM PpiD-**PQQ**, and 100 μM **PQQ**. (P) IPMS of SurA treated with 3-fold molar excess **PQQ**. (Q) IPMS of MBP treated with 3-fold molar excess **PQQ**. (R) IPMS of PpiD treated with 3-fold molar excess **PQQ**.

Given the labeling of some proteins, even without UV irradiation (Figure S8 and Table S14), we first tested if some of the hits may covalently bind to **PQQ**. 100 μM recombinant Tig, HtpG, MBP, FklB, RuvB, SurA, and PpiD were incubated with 300 μM **PQQ** at 4 °C overnight, followed by intact protein LC–MS analysis (Figures 6G–I, P–R and S21D–J). Notably, deconvoluted spectra indeed confirmed a characteristic 310 Da mass shift indicative of covalent **PQQ** binding in the case of Tig, HtpG, MBP, RuvB, SurA, and PpiD, but not for FklB as well as PedH (Figure S21J,K). Moreover, the characteristic peak at 73529 Da for HtpG indicates decarboxylation (–3COOH) of the **PQQ** moiety, resulting in a 135 Da shift (Figure 6H, see also SurA, MBP, and PpiD in Figure 6P–R).^63^ The extend of modification varied between 10 – 30% and could be increased up to 20 – 60% at higher **PQQ** concentrations (Figure S21D–I). BSA was included as a negative control and was not modified under the same conditions (Figure S21L). These results suggest that some identified proteins are covalently modified by **PQQ**.

This notion was supported by MS/MS analysis of **PQQ-**treated *E. coli* K-12 lysate. The proteome was cleaned up via carboxylated beads, followed by tryptic digestion and mass spectrometric analysis (MS/MS), which was then analyzed with Fragpipe^64–66^ and Maxquant^67–69^ (Figure 7A). As a control, we performed the same workflow with untreated *E. coli* lysate. Data processing with MSFragger revealed a mass shift of 309.9860 Da, which is in line with the intact protein mass spectrometry (IPMS) mass difference of 310 Da. A subsequent offset search with this signature mass revealed that **PQQ** shows a preference for covalently modifying lysine (Figure 7B). The identified peptides in the following closed search were identified as true modified peptides if the MS/MS and MBR (match between runs) as the match type > = 2, PSMs (total peptide spectrum match) ≥ 2, Peptide prophet Probability > 0.75, and standard deviation of log_10_-intensity < 0.5. The closed search of 309.9860 Da with specifying lysine as a **PQQ-**modified amino acid confirmed Tig and 6GPD as covalent binding protein hits (Figures 7C,D and S22–S24); Gene ontology analysis revealed that the identified proteins are involved in translation, protein/ribosome assembly, and peptide/amide biosynthesis (Figure 7E).

**Figure 7:**
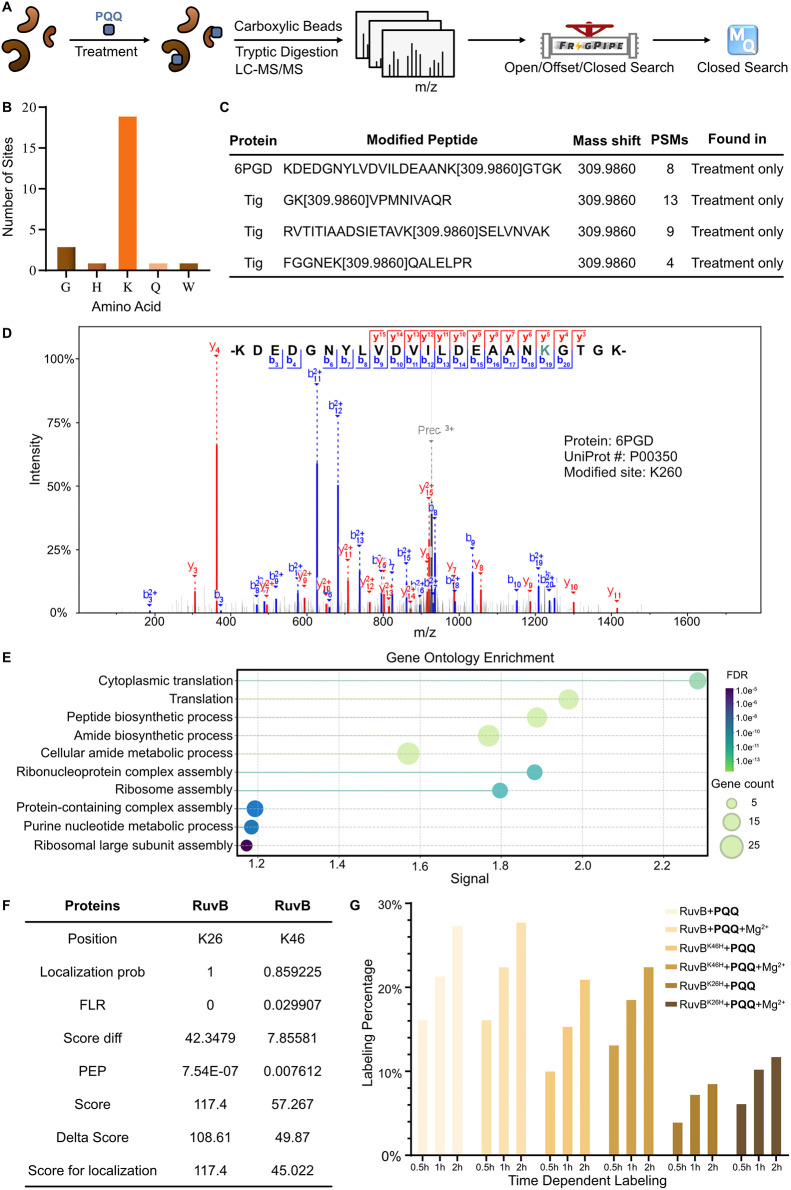
General MS/MS workflow and **PQQ** covalent binding proteins analysis. (A) General MS/MS workflow. Intact proteins were treated with **PQQ**, followed by cell lysis, cleanup with carboxylated beads, digestion by trypsin and analysis by mass spectrometry, which was then further analyzed with Fragpipe and Maxquant. (B) Amino acid selectivity of **PQQ** in *E. coli* K-12 proteome from offset search. *E. coli* K-12 lysate of 3.5 mg/mL was treated with 2000 μM **PQQ** and 2000 μM CaCl_2_ at 37 °C for 4 h. (C) Table of representative **PQQ** covalent binding proteins from closed search. The experimental details are shown in Figure 7B. (D) MS/MS spectrum of 6GPD peptide (-KDEDGNYLVDVILDEAANKGTGK-) identified by Fragpipe PDV.^71^ (E) Gene ontology analysis of biological process with **PQQ** covalent binding proteins from closed search in the above MS/MS workflow. This was analyzed via String with “Group Similarity” ≥ 0.8 and highest confidence ≥ 0.9. (F) Table of RuvB-binding site identification from **PQQ** treatment analyzed by Maxquant. 100 μM RuvB was treated with 20-fold molar excess **PQQ** and incubated at 30 °C for 1 h. (G) Time-dependent labeling of intact RuvB/RuvB^k46H^/RuvB^K26H^ treated with **PQQ**. 100 μM RuvB/RuvB^k46H^/RuvB^K26H^ was treated with 20-fold molar excess of **PQQ** and 20-fold molar excess of MgCl_2_ and incubated at 30 °C for time-dependent labeling.

To elucidate a selected covalent binding site in more detail, we performed MS/MS sequencing with purified RuvB, a AAA+ ATPase involved in DNA remodeling. Modified sites of RuvB were regarded as true positives if they exhibit a localization probability > 0.85, false localization rate (FLR) < 0.05, and Posterior Error Probability (PEP) < 0.01 from Maxquant analysis. Two lysine residues (K26 and K46, Figures 7F and S25A) exhibited the characteristic 310 Da mass shift and were consistently identified in two independent experiments (RuvB treated with **PQQ** only; RuvB treated with **PQQ** and MgCl_2_). To experimentally validate these binding sites, we prepared the corresponding RuvB mutant proteins RuvB^K26H^ and RuvB^K46H^, which were incubated with **PQQ** and analyzed by intact protein MS (Figures 7G and S25B–G). While a characteristic mass shift as well as protein labeling was observed in the case of RuvB^K46H^, RuvB^K26H^ showed significantly decreased modification with the cofactor, confirming K26 as the predominant covalent binding site. These changes of intensity were corroborated by **PQQ4** labeling, click to rhodamine azide and subsequent fluorescent gel-based analysis with the mutants and wt enzyme (Figure S25H). Additionally, we used several databases to predict **PQQ**-dependent enzymes or **PQQ**-binding proteins to further substantiate the validity and comprehensive coverage of our developed method. Collectively, four **PQQ**-associated proteins (YliI, PedH, Gcd, and BamB) were detected in this study out of six known/predicted **PQQ**-dependent enzymes (YliI, PedH, Gcd, BamB, PedE, and QuiA) in *E. coli* K-12 and *P. putida* KT2440 (Table S26; for **PQQ**-binding proteins, refer to Table S27).

## Conclusion

**PQQ** is a versatile cofactor involved in enzymatic redox reactions. While **PQQ**-dependent alcohol and sugar dehydrogenases have been described in bacteria, the full complement of the quinoproteome remains unknown. Our diverse set of functionalized **PQQ** probes confirmed binding, reconstitution of catalysis, as well as labeling of the known **PQQ** enzymes YliI and PedH, which we used for tool validation prior to in-depth studies in living bacterial cells. We identified a confined suite of putative **PQQ**-binding proteins in *E. coli* and *P. putida*, which we validated for cofactor binding via complementary methods. Satisfyingly, our diverse probe design turned out to match the different binding sites, which would not have been possible with just one molecule. Intriguingly, we observed that **PQQ** binding to some proteins occurred via a covalent lysine modification. This notion was confirmed via labeling of the RuvB protein, where K26 was identified as the binding site. However, given the overall lower enrichment of quinoproteins in the absence of UV irradiation, we conclude that irreversible binding is a trait of only some proteins. While we can only speculate about the physiological impact of covalency, it is conceivable that the general reactivity of **PQQ** is most likely conserved in other organisms, including mammalian cells.^62^ Given the use of **PQQ** as a dietary supplement, irreversible binding to proteins is of particular interest in order to study the associated nutritional and health effects. Thus, our tools provide a basis for these and other studies investigating the inventory of quinoproteins in diverse organisms.

## Supplementary Material















## Data Availability

Mass spectrometry proteomics data have been deposited to the ProteomeXchange Consortium^72^ via the PRIDE^73^ partner repository with the data set identifier PXD069310 and PXD075565.

## Notes

Additional experimental details, including methods, procedures, figures, characterization, and spectra are attached in the Supporting Information (PDF)
Identification of PQQ covalent binding proteins in E. coli K-12 (XLSX)
Identification of RuvB covalent binding site from PQQ treatment (XLSX)
Target identification by chemical proteomics in E. coli BL21 (tuner) cells (XLSX)
Target identification by chemical proteomics in E. coli K-12 cells (XLSX)
Target identification by chemical proteomics in HepG2 cells (XLSX)
Target identification by chemical proteomics in P. putida KT2440 cells (XLSX)
