# Supplementary material for: Chemical Proteomics Reveal the Inventory of Pyrroloquinoline Quinone Binding Proteins in Bacteria
Source: J Am Chem Soc. 2026 Apr 2;148(14):15306–19. doi: 10.1021/jacs.6c03427 (PMC13088180; doi:10.1021/jacs.6c03427)
Supplement: Supplementary file 3 [file ja6c03427_si_003.pdf]

Chemical proteomics reveal the inventory of pyrroloquinoline quinone binding proteins in bacteria

Tao Wang, Rahel Mühlhofer, E Lei, Wei Ding, Andreas S. Klein, Cathleen Zeymer*, and Stephan A. Sieber*

Center for Functional Protein Assemblies (CPA), Department of Bioscience, TUM School of Natural Sciences, Technical University of Munich (TUM), Ernst-Otto-Fischer-Straße 8, 85748 Garching, Germany

Table of Content

1. Chemistry Methods
   - 1.1. General Information
   - 1.2. Chemical Synthesis and Analytical Data
2. Biological Methods
   - 2.1. Bacterial Culture Conditions
   - 2.2. Bacterial Media Preparation
   - 2.3. HepG2 Cell Culture Conditions
   - 2.4. Gel-Based Fluorescent in situ Labeling and Lysate Labeling
   - 2.5. Preparative Labeling Analysis
   - 2.6. Full Proteome Analysis
   - 2.7. MS/MS Workflow
   - 2.8. LC-MS/MS Measurements on TimsTOF Pro
   - 2.9. Preparative Labeling and Full Proteome LC-MS/MS Data Analysis
   - 2.10. MS/MS Data Analysis
   - 2.11. Molecular Cloning
   - 2.12. DNA and Protein Sequences of All Constructs
   - 2.13. Recombinant Protein Expression and Purification
   - 2.14. UV/vis Absorption-Based Binding Assay for PQQ and PQQ-Derivative Probes
   - 2.15. Coupled Colorimetric Enzyme Activity Assay for PQQ-Dependent Sugar and Alcohol Dehydrogenases
   - 2.16. Intact Protein Mass Spectrometry (IPMS) Experiments
   - 2.17. Computational Modelling of PQQ Probe Binding to YliI and PedH
3. Supplementary Figures and Tables
4. NMR Spectra
5. References

1. Chemistry Methods

1.1. General Information

Chemicals were purchased from commercial suppliers (Sigma-Aldrich, Enamine and TCI) and used as delivered. Anhydrous solvents, deuterated solvents and high-performance liquid chromatography (HPLC) grade solvents were bought from Sigma-Aldrich, VWR chemicals, Fisher chemicals, abcr and BLDpharm. Technical grade solvents were distilled for flash column chromatography. Unless otherwise stated, all reactions and manipulations were carried out with *Schlenk* technique under an ambient atmosphere in new reaction flasks. Room temperature (RT) is defined as 25 ^{o}C. Other **PQQ** probes were not used in this research, such as **PQQ3**, **PQQ5** and **PQQ7**.

NMR Spectra were recorded on a Bruker Avance-III-400, Bruker Avance-III-500, Bruker Avance-III-600. Chemical shifts are reported in ppm with the solvent resonance as the internal standard. For ^{1}H NMR: $CDCl_3$, 7.26; DMSO-d_6, 2.50; $CDCN_3$, 1.94; CD_3OD, 3.31; DMF-d_7, 2,75, 2.92, 8.03; For ^{13}C NMR: $CDCl_3$, 77.16; DMSO-d_6, 39.52; $CDCN_3$, 1.32, 118.26; CD_3OD, 49.00; DMF-d_7, 29.76, 34.89, 163.15. Data is reported as follows: s = singlet, d = doublet, t = triplet, q = quartet, td =triplet of doublets, m = multiplet, br = broad singlet, coupling constants in Hz; integration.

Mass Spectra (MS) and High-Resolution Mass Spectrometry (HRMS) were determined in the Center for Functional Protein Assemblies of the Technical University of Munich. Electrospray Ionization Low-Resolution Mass Spectrometry (ESI-LRMS) were measured on a MSQ plus (Thermo Fisher Scientific Inc.) coupled with a Dionex Ultimate 3000 HPLC. For Electrospray Ionization High-Resolution Mass Spectrometry (ESI-HRMS), an LTQ-FT Ultra (Thermo Fisher Scientific Inc.) or Q Exactive plus (Thermo Fisher Scientific Inc.) coupled to a Dionex Ultimate 3000 HPLC was applied.

Flash Column Chromatography was accomplished using silica gel 60 (0.04 – 0.063 mm / 230 – 400 mesh ASTM), which was purchased from Merck KGaA.

Analytical / Preparative thin-layer chromatography (TLC) was carried out on pre-coated aluminum sheets provided by Merck silica gel 60 F254 plates. Components were visualized by treatment with aqueous phosphomolybdic solution or by irradiation under UV light (254 nm or 365 nm).

Analytical / Preparative High-Performance Liquid Chromatography (HPLC) was conducted with Waters 2695 quaternary gradient module coupled to a Waters PDA 2998 or Waters 2545 quaternary gradient module coupled to a Waters PDA 2998. For analytical purpose, a Waters XBridge reverse phase C_{18} column (3.5 μm, 4.6 × 100 mm, flow: 1.2 mL / min) or XBride BEH amide (5 μm, 4.6 × 100 mm, flow: 1.2 mL / min) was installed on Waters 2695; for preparative purpose, a waters XBridge reverse phase C_{18} column (10 μm, 30 × 150 mm, flow: 50 mL / min) or XBride BEH amide (5 μm, 10 × 250 mm, flow: 50 mL / min) was installed on Waters 2545. All the final **PQQ** probes were purified with preparative HPLC to achieve high purities to minimize the impurities background in biological experiments

UV/vis Spectroscopy were recorded on a *Jasco* V-750 photometer (*JASCO* Deutschland GmbH) at room temperature (data interval: 1 nm: bandwidth: 0.2 nm; response: 0.24 s, path length: 1 cm) or Varioskan Lux Multimode Microplate Reader (Thermo Fisher Scientific Inc) at room temperature (data interval: 1 nm). Background absorption was corrected by recording a blank spectrum in advance.

1.2. Chemical Synthesis and Analytical Data

Scheme S1. Retrosynthesis of PQQ probes

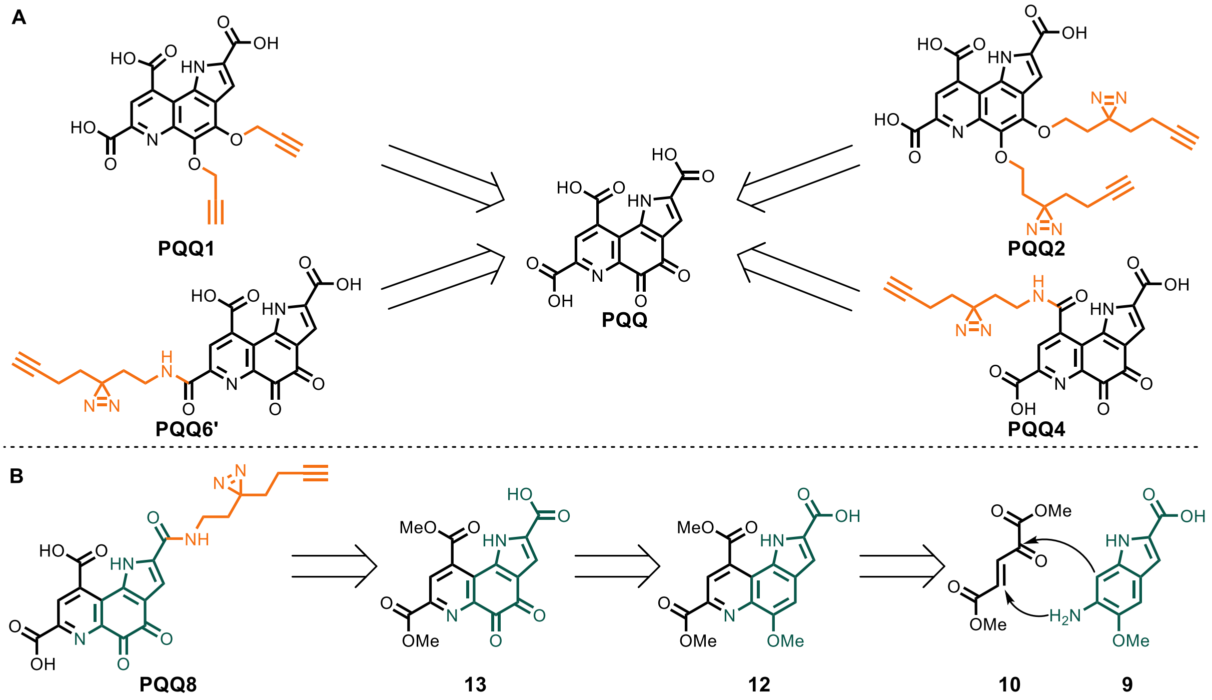

Trimethyl 4,5-dioxo-4,5-dihydro-1H-pyrrolo[2,3-f]quinoline-2,7,9-tricarboxylate (**1**) The synthesis and spectroscopic data of the compound were in accordance with the reported literature[1]

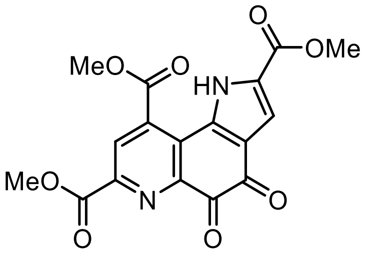

To a mixture of pyrroloquinoline quinone (**PQQ**, 1.98 g, 6 mmol) and potassium carbonate (7.46 g, 54 mmol) in 17 mL anhydrous DMF, dimethyl sulfate was dropwise added (17.0 mL, 180 mmol) at room temperature, then the reaction was stirred at room temperature for 2 days. The reaction was quenched with 40 mL H_2O, then stirred at room temperature for 2 h, when yellow precipitate developed. The resulting precipitate was filtered, washed with 20 mL H_2O and lyophilized to afford the crude product as **PQQ** cannot totally convert to the product even with prolonged reaction time. The crude product reacted with dimethyl sulfate (17.0 mL, 180 mmol) and potassium carbonate (7.46 g, 54 mmol) in 17 mL anhydrous DMF at room temperature for another 2 days. Then the reaction was quenched with 40 mL H_2O, stirred for 2 h to precipitate the product and the resulting precipitate was filtered, washed with 20 mL H_2O and lyophilized to afford trimethyl 4,5-dioxo-4,5-dihydro-1H-pyrrolo[2,3-f]quinoline-2,7,9-tricarboxylate (**1**, 1.72 g, 77%) as yellow solid.

^{1}H NMR (400 MHz, DMSO-d_6) δ 12.51 (s, 1H), 8.56 (s, 1H), 7.28 (d, J = 2.1 Hz, 1H), 4.05 (s, 3H), 3.96 (s, 3H), 3.89 (s, 3H). ^{13}C NMR (100 MHz, DMSO-d_6) δ 177.04, 173.30, 166.60, 163.82, 159.86, 148.94, 145.77, 134.16, 133.55, 128.53, 126.60, 126.52, 124.98, 113.87, 54.25, 52.98, 52.39.

HRMS (ESI) m/z: [M-1]$^-$ calcd. 371.0520, found 371.0526.

Trimethyl 4,5-dihydroxy-1H-pyrrolo[2,3-f]quinoline-2,7,9-tricarboxylate (2) The synthesis was prepared according to a published literature procedure[2]

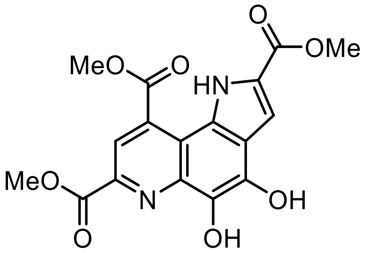

To a mixture of trimethyl 4,5-dioxo-4,5-dihydro-1H-pyrrolo[2,3-f]quinoline-2,7,9-tricarboxylate (**1**, 112 mg, 0.3 mmol) in 200 mL anhydrous MeOH, phenylhydrazine hydrochloride (216 mg, 1.5 mmol) was added under argon atmosphere at room temperature. Next, the reaction was heated up to 50 ^{o}C and stirred for 1 h. The reaction mixture was cooled down to room temperature and filtered, washed with 10 mL MeOH, further dried under vacuum to afford the trimethyl 4,5-dihydroxy-1H-pyrrolo[2,3-f]quinoline-2,7,9-tricarboxylate (**2**, 110 mg, 98%) as yellow solid.

^{1}H NMR (500 MHz, DMF-d_7) δ 12.29 (s, 1H), 10.72 (s, 1H), 8.82 (br, 1H), 8.63 (s, 1H), 7.50 (d, *J* = 2.4 Hz, 1H), 4.21 (s, 3H), 4.06 (s, 3H), 4.01 (s, 3H). ^{13}C NMR (100 MHz, DMF-d_7) δ 168.89, 165.16, 161.45, 143.77, 142.44, 138.81, 132.56, 131.26, 127.30, 124.17, 123.38, 120.32, 111.07, 106.02, 54.04, 52.74, 52.13.

HRMS (ESI) m/z: $[M+1]^{+}$ calcd. 375.0823, found 375.0822.

1-(Prop-2-yn-1-yl)-4,5-bis(prop-2-yn-1-yloxy)-1H-pyrrolo[2,3-f]quinoline-2,7,9-tricarboxylic acid (PQQ1)

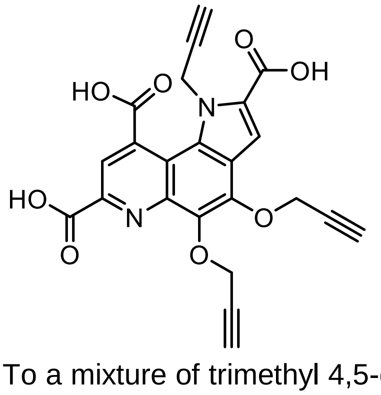

To a mixture of trimethyl 4,5-dihydroxy-1H-pyrrolo[2,3-f]quinoline-2,7,9-tricarboxylate (**1**, 52.0 mg, 0.14 mmol) and cesium carbonate (101.0 mg, 0.31 mmol) in 1 mL anhydrous DMF, propargyl bromide (60 μL, 0.7 mmol) was added under argon atmosphere, then the reaction was stirred at room temperature for 2 days. The reaction mixture was purified with preparative TLC (DCM: MeOH, 50: 1) to afford the intermediate trimethyl 1-(prop-2-yn-1-yl)-4,5-bis(prop-2-yn-1-yloxy)-1H-pyrrolo[2,3-f]quinoline-2,7,9-tricarboxylate.

To trimethyl 1-(prop-2-yn-1-yl)-4,5-bis(prop-2-yn-1-yloxy)-1H-pyrrolo[2,3-f]quinoline-2,7,9-tricarboxylate dissolved in 1 mL THF and 1 mL H_2O solution, lithium hydroxide (23.5 mg, 0.98 mmol) was added under argon atmosphere, and the reaction was stirred at room temperature for 6 h. The reaction was quenched with 0.5 mL of 6 M HCl aq., and further purified with preparative HPLC (C_{18}, from 10: 90 to 90: 10, 0.1% TFA in CH_3CN: 0.1% TFA in H_2O) to afford the 1-(prop-2-yn-1-yl)-4,5-bis(prop-2-yn-1-yloxy)-1H-pyrrolo[2,3-f]quinoline-2,7,9-tricarboxylic acid (**PQQ1**) as a 2,2,2-trifluoroacetate salt (6.1 mg, 8% yield for two steps) as yellow solid.

^{1}H NMR (400 MHz, MeOD) δ 8.48 (s, 1H), 7.63 (s, 1H), 5.37 (d, *J* = 2.4 Hz, 2H), 5.30 (d, *J* = 2.4 Hz, 2H), 5.19 (d, *J* = 2.4 Hz, 2H), 2.94 (t, *J* = 2.4 Hz, 1H), 2.92 (t, *J* = 2.4 Hz, 1H), 2.49 (t, *J* = 2.4 Hz, 1H). ^{13}C NMR (100 MHz, MeOD) δ 170.25, 167.38, 163.73, 147.15, 146.36, 138.51, 134.67, 132.53, 125.73, 120.23, 114.52, 113.51, 80.13, 79.93, 79.16, 77.70, 77.38, 74.72, 62.94, 61.79, 41.12.
HRMS (ESI) m/z: $[M-1]^-$ calcd. 445.0677, found 445.0672.

3-(But-3-yn-1-yl)-3-(2-iodoethyl)-3H-diazirine (**3**) The synthesis and spectroscopic data of the compound were in accordance with the reported literature[3]

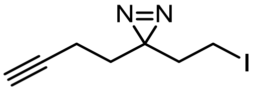

To a solution of triphenylphosphine (205 mg, 0.78 mmol), imidazole (147 mg, 2.16 mmol) in 5 mL DCM, iodine (219 mg, 0.86 mmol) was added at 0 ºC, and the reaction was stirred at 0 ºC for 10 min. Next, 2-(3-(but-3-yn-1-yl)-3H-diazirin-3-yl)ethan-1-ol (98 mg, 0.72 mmol) in 1 mL DCM solution was dropwise added to the reaction, and the reaction was stirred at 0 ºC for 5 h. After the reaction was completed, it was quenched with 1 mL sat. sodium sulfite solution, extracted with DCM (3 ×10 mL), and the combined organic phase was washed with 20 mL sat. brine, concentrated under vacuum. The residue mixture was purified with silica gel chromatography (hexane: ethyl acetate, 50: 1) to afford 3-(but-3-yn-1-yl)-3-(2-iodoethyl)-3H-diazirine (**3**, 142 mg, 80%) as colorless oil.
^{1}H NMR (400 MHz, $CDCl_3$) δ 2.89 (t, *J* = 7.6 Hz, 2H), 2.12 (t, *J* = 7.6 Hz, 2H), 2.06 – 1.99 (m, 3H), 1.69 (t, *J* = 7.1 Hz, 2H). ^{13}C NMR (100 MHz, $CDCl_3$) δ 82.58, 69.58, 37.67, 31.98, 28.76, 13.40, -3.90.

4,5-Bis(2-(3-(but-3-yn-1-yl)-3H-diazirin-3-yl)ethoxy)-1H-pyrrolo[2,3-f]quinoline-2,7,9-tricarboxylic acid (PQQ2)

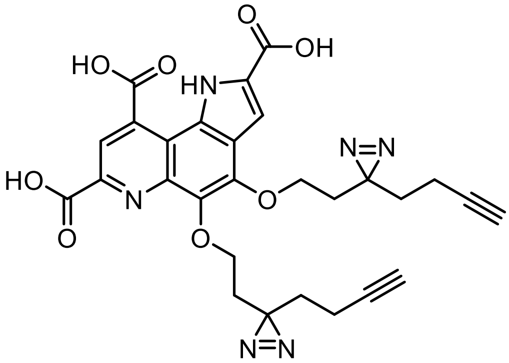

To a mixture of trimethyl 4,5-dihydroxy-1H-pyrrolo[2,3-f]quinoline-2,7,9-tricarboxylate (**2**, 37 mg, 0.1 mmol) and cesium carbonate (78 mg, 0.3 mmol) in 0.5 mL anhydrous DMF, 3-(but-3-yn-1-yl)-3-(2-iodoethyl)-3H-diazirine (**3**, 75 mg, 0.3 mmol) was added under argon atmosphere, then the reaction was stirred at room temperature for 4 days. After the reaction was completed, it was purified with preparative TLC (DCM: MeOH, 30: 1) to get the intermediate trimethyl 4,5-bis(2-(3-(but-3-yn-1-yl)-3H-diazirin-3-yl)ethoxy)-1H-pyrrolo[2,3-f]quinoline-2,7,9-tricarboxylate. To the above solution of trimethyl 4,5-bis(2-(3-(but-3-yn-1-yl)-3H-diazirin-3-yl)ethoxy)-1H-pyrrolo[2,3-f]quinoline-2,7,9-tricarboxylate in 0.5 mL THF and 0.5 mL H_2O lithium hydroxide (48 mg, 2.0 mmol) was added under argon atmosphere, and the reaction was stirred at room temperature for 6 h. The reaction was quenched with 0.5 mL of 6 M HCl aq., and further purified with preparative HPLC (C_{18}, from 10: 90 to 90: 10, 0.1% TFA in CH_3CN: 0.1% TFA in H_2O) to afford the 4,5-bis(2-(3-(but-3-yn-1-yl)-3H-diazirin-3-yl)ethoxy)-1H-pyrrolo[2,3-f]quinoline-2,7,9-

tricarboxylic acid (**PQQ2**) as a 2,2,2-trifluoroacetate salt (7.8 mg, 11% yield for two steps) as brown solid.
^{1}H NMR (500 MHz, DMSO-d_6) δ 13.40 (br, 4H), 8.59 (s, 1H), 7.42 (s, 1H), 4.31 (s, 2H), 4.10 (s, 2H), 2.83 (s, 1H), 2.80 (s, 1H), 2.08 (s, 4H), 2.03 (s, 4H), 1.82 (s, 2H), 1.74 (s, 2H). ^{13}C NMR (125 MHz, DMSO-d_6) δ 169.42, 166.26, 161.94, 145.40, 145.12, 144.78, 137.70, 127.68, 126.80, 121.75, 121.66, 113.95, 106.18, 83.38, 83.20, 71.86, 71.73, 69.56, 67.69, 33.38, 32.89, 31.78, 31.43, 27.25, 27.18, 12.85, 12.76.
HRMS (ESI) m/z: $[M-1]^-$ calcd. 571.1582, found 571.1576.

2,9-Bis(methoxycarbonyl)-4,5-dioxo-4,5-dihydro-1H-pyrrolo[2,3-f]quinoline-7-carboxylic acid (4) The synthesis and spectroscopic data of the compound were in accordance with the reported literature[1]

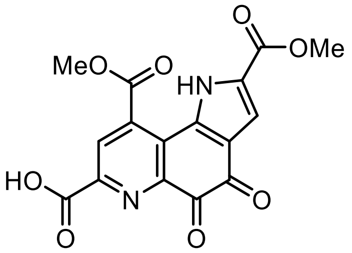

Trimethyl 4,5-dioxo-4,5-dihydro-1H-pyrrolo[2,3-f]quinoline-2,7,9-tricarboxylate (**1**, 372 mg, 1.0 mmol) dissolved in 10 mL TFA and 5 mL H_2O solution was stirred at 60 ºC overnight. After the reaction was completed, it was cooled down to room temperature and 20 mL H_2O was added and stirred at room temperature for 2 h to precipitate the product. The resulting precipitate was filtered, washed with 10 mL H_2O and dried under vacuum to afford the 2,9-bis(methoxycarbonyl)-4,5-dioxo-4,5-dihydro-1H-pyrrolo[2,3-f]quinoline-7-carboxylic acid (**4**, 358 mg, 100%) as yellow solid.
^{1}H NMR (400 MHz, DMSO-d_6) δ 12.52 (br, 1H), 8.56 (s, 1H), 7.28 (s, 1H), 4.05 (s, 3H), 3.89 (s, 3H). ^{13}C NMR (100 MHz, DMSO-d_6) δ 177.28, 173.36, 166.77, 164.82, 159.89, 148.90, 147.02, 134.09, 133.76, 128.51, 126.40, 126.31, 124.84, 113.87, 54.21, 52.38.
HRMS (ESI) m/z: $[M+1]^+$ calcd, 359.0510, found 359.0507.

7-((2-(3-(But-3-yn-1-yl)-3H-diazirin-3-yl)ethyl)carbamoyl)-4,5-dioxo-4,5-dihydro-1H-pyrrolo[2,3-f]quinoline-2,9-dicarboxylic acid (PQQ4)

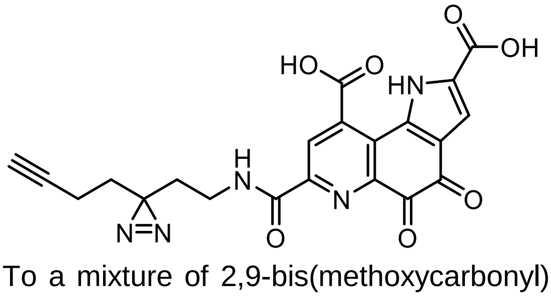

To a mixture of 2,9-bis(methoxycarbonyl)-4,5-dioxo-4,5-dihydro-1H-pyrrolo[2,3-f]quinoline-7-carboxylic acid (**4**, 36 mg, 0.1 mmol), PyBOP (57 mg, 0.11 mmol) and DIPEA (27 µL, 0.2 mmol) in 0.5 mL anhydrous DMF, 2-(3-(but-3-yn-1-yl)-3H-diazirin-3-yl)ethan-1-amine (15 mg, 0.11 mmol) was added under argon atmosphere, then the reaction was stirred at room temperature overnight. The reaction was quenched with 0.5 mL of 6 M HCl aq., and purified with preparative HPLC (C_{18}, from 10: 90 to 90: 10, CH_3CN: H_2O) to afford the intermediate dimethyl 7-((2-(3-(but-3-yn-1-yl)-3H-diazirin-3-yl)ethyl)carbamoyl)-4,5-dioxo-4,5-dihydro-1H-pyrrolo[2,3-f]quinoline-2,9-dicarboxylate.

To the above dimethyl 7-((2-(3-(but-3-yn-1-yl)-3H-diazirin-3-yl)ethyl)carbamoyl)-4,5-dioxo-4,5-dihydro-1H-pyrrolo[2,3-f]quinoline-2,9-dicarboxylate in 0.5 mL THF and 0.5 mL H_2O, lithium hydroxide (27 mg, 1.15 mmol) was added under argon atmosphere, and the reaction was stirred at room temperature for 6 h. The reaction was quenched with 0.5 mL of 6 M HCl aq., and further purified with preparative HPLC (BEH amide, HILIC column, from 95: 5 to 60: 40, CH_3CN: 10 mM $HCOONH_4$ in H_2O) to afford the 7-((2-(3-(but-3-yn-1-yl)-3H-diazirin-3-yl)ethyl)carbamoyl)-4,5-dioxo-4,5-dihydro-1H-pyrrolo[2,3-f]quinoline-2,9-dicarboxylic acid (**PQQ4**) as a formate salt (1.9 mg, 4 % yield for two steps) as yellow solid.
^{1}H NMR (500 MHz, DMSO-d_6) δ 13.49 (br, 1H), 9.33 (s, 1H), 8.56 (s, 1H), 7.12 (s, 1H), 3.29 – 3.24 (m, 2H), 2.84 (q, *J* = 2.7, 2.2 Hz, 1H), 2.03 (td, *J* = 7.4, 2.7 Hz, 2H), 1.71 (t, *J* = 7.4 Hz, 2H), 1.65 (t, *J* = 7.4 Hz, 2H). ^{13}C NMR (125 MHz, DMSO-d_6) δ 177.64, 167.17, 163.51, 162.98, 147.32, 146.07, 144.40, 126.78, 123.14, 121.42, 83.63, 72.35, 34.58, 32.73, 31.75, 27.77, 13.18.
HRMS (ESI) m/z: $[M+1]^+$ calcd 450.1045, found 450.1039.

7-(Methoxycarbonyl)-4,5-dioxo-4,5-dihydro-1H-pyrrolo[2,3-f]quinoline-2,9-dicarboxylic acid (6) The synthesis was prepared according to a published literature procedure[4]

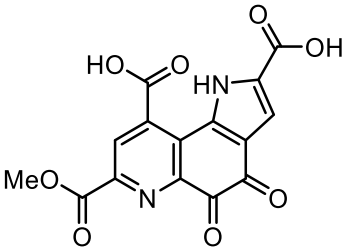

To a mixture of trimethyl 4,5-dioxo-4,5-dihydro-1H-pyrrolo[2,3-f]quinoline-2,7,9-tricarboxylate (**1**, 186 mg, 0.5 mmol) in 12.5 mL CH_3CN, 6.25 mL of 0.8 M potassium carbonate solution was added, and the reaction was stirred at 60 ºC for 3.5 h. The reaction was cooled down to room temperature, and adjusted pH 1.0 with 6 M HCl aq., further continued to stir at room temperature for 2 h to precipitate the product. The resulting precipitate was filtered, washed with 10 mL CH_3CN and 10 mL H_2O and dried under vacuum to afford the 7-(methoxycarbonyl)-4,5-dioxo-4,5-dihydro-1H-pyrrolo[2,3-f]quinoline-2,9-dicarboxylic acid (**6**, 154 mg, 82 %) as brown solid.
^{1}H NMR (400 MHz, DMSO-d_6) δ 13.38 (br, 1H), 8.60 (s, 1H), 7.20 (d, *J* = 2.2 Hz, 1H), 3.95 (s, 3H). ^{13}C NMR (100 MHz, DMSO-d_6) δ 178.20, 173.80, 169.03, 164.46, 161.36, 149.28, 146.06, 136.80, 134.57, 129.69, 128.19, 127.20, 125.11, 114.01, 53.35.
HRMS (ESI) m/z: $[M+1]^+$ calcd, 345.054, found 345.0352.

2,7-Bis(methoxycarbonyl)-4,5-dioxo-4,5-dihydro-1H-pyrrolo[2,3-f]quinoline-9-carboxylic acid (7) The synthesis was prepared according to a published literature procedure[4]

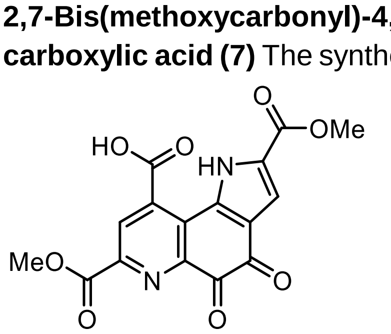

To a mixture of 7-(methoxycarbonyl)-4,5-dioxo-4,5-dihydro-1H-pyrrolo[2,3-f]quinoline-2,9-dicarboxylic acid (**6**, 206 mg, 0. 6 mmol) in 15 mL MeOH, two drops of concentrated sulfuric acid were added, and the reaction was refluxed for 2 h. The reaction was cooled down to room

temperature and 5 mL water was added followed by stirring at room temperature for another 2 h to precipitate the product. Then the resulting precipitate was filtered, washed with 8 mL MeOH and 2 mL H_2O and dried under vacuum to afford the 2,7-bis(methoxycarbonyl)-4,5-dioxo-4,5-dihydro-1H-pyrrolo[2,3-f]quinoline-9-carboxylic acid (**7**, 201 mg, 94%) as yellow solid.
^{1}H NMR (500 MHz, DMSO-d_6) δ 13.58 (s, 1H), 8.60 (s, 1H), 7.26 (d, J = 2.2 Hz, 1H), 3.96 (s, 3H), 3.87 (s, 3H). ^{13}C NMR (100 MHz, DMSO-d_6) δ 177.60, 173.25, 168.52, 163.99, 159.92, 148.87, 145.75, 136.62, 134.59, 129.27, 126.62, 126.23, 124.57, 113.92, 52.89, 52.29.
HRMS (ESI) m/z: $[M+1]^+$ calcd. 359.0510, found 359.0510.

Dimethyl 9-((2-(3-(but-3-yn-1-yl)-3H-diazirin-3-yl)ethyl)carbamoyl)-4,5-dioxo-4,5-dihydro-1H-pyrrolo[2,3-f]quinoline-2,7-dicarboxylate (PQQ6)

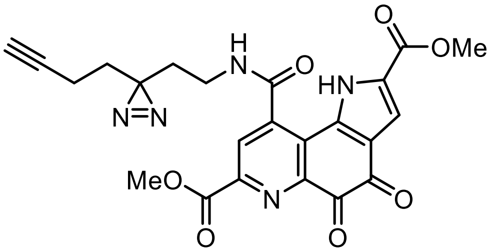

To 2,7-bis(methoxycarbonyl)-4,5-dioxo-4,5-dihydro-1H-pyrrolo[2,3-f]quinoline-9-carboxylic acid (**7**, 18 mg, 0.05 mmol), PyBOP (31 mg, 0.06 mmol) and DIPEA (8 µL, 0.06 mmol) in 0.5 mL anhydrous DMF mixture, 2-(3-(but-3-yn-1-yl)-3H-diazirin-3-yl)ethan-1-amine (9 mg, 0.06 mmol) was added under argon atmosphere, then the reaction was stirred at room temperature overnight. The reaction was purified with preparative TLC with DCM / MeOH (20: 1) and further purified with preparative HPLC (C_{18}, from 10: 90 to 90: 10, CH_3CN: H_2O) to afford dimethyl 9-((2-(3-(but-3-yn-1-yl)-3H-diazirin-3-yl)ethyl)carbamoyl)-4,5-dioxo-4,5-dihydro-1H-pyrrolo[2,3-f]quinoline-2,7-dicarboxylate (**PQQ6**, 2.0 mg, 8% yield) as yellow solid.
^{1}H NMR (500 MHz, CD_3CN) δ 8.42 (s, 1H), 7.31 (s, 1H), 3.99 (s, 3H), 3.90 (s, 3H), 3.39 – 3.48 (m, 2H), 2.20 (t, *J* = 2.7 Hz, 1H), 2.05 (td, *J* = 7.4, 2.7 Hz, 2H), 1.81 (t, *J* = 6.9 Hz, 2H), 1.71 (d, *J* = 7.4 Hz, 2H). ^{13}C NMR (125 MHz, MeOD) δ 188.14, 169.95, 166.17, 161.67, 159.52, 146.02, 140.53, 135.54, 128.35, 126.13, 122.35, 122.28, 114.20, 94.57, 83.55, 70.49, 53.86, 52.80, 36.66, 33.08, 32.95, 28.04, 13.85.
HRMS (ESI) m/z: $[M+1]^+$ calcd, 478.1358, found 478.1357.

2-Carboxy-5-methoxy-1H-indol-6-aminium chloride (9)

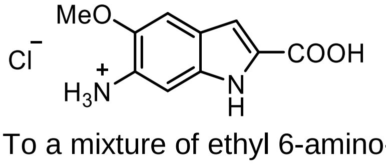

To a mixture of ethyl 6-amino-5-methoxy-1H-indole-2-carboxylate (**8**, 4.511 g, 19.26 mmol) in 20 mL THF and 20 mL H_2O, LiOH (2.121 g, 88.5 mmol) was added at room temperature, then the reaction was refluxed overnight. The reaction was cooled down to room temperature and adjusted pH 1.0 with 6 M HCl aq. to precipitate the product. The resulting precipitate was filtered, washed with 10 mL THF, and dried under vacuum to afford the 2-carboxy-5-methoxy-1H-indol-6-aminium chloride (**9**, 4.568 g, 98%) as light brown solid.
^{1}H NMR (400 MHz, DMSO-d_6) δ 11.88 (s, 1H), 10.28 (br, 2H), 7.67 (s, 1H), 7.33 (s, 1H), 7.05 (d, *J* = 1.4 Hz, 1H), 3.87 (s, 3H).^{13}C NMR (100 MHz, DMSO-d_6) δ 162.42, 147.08, 131.07, 129.75, 126.15, 119.68, 108.11, 107.03, 103.02, 56.19.
HRMS (ESI) m/z: $[M+1]^+$ calcd. 207.0765, found 207.0763.

Dimethyl (E)-4-oxopent-2-enedioate (10) The synthesis and spectroscopic data of the compound were in accordance with the reported literature[5]

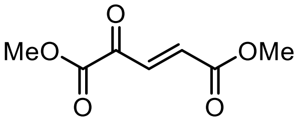

To a solution of dimethyl 2-oxopentanedioate (1.40 g, 8 mmol) in 30 mL DCM, bromine (0.62 mL, 12 mmol) was added at room temperature, and then the reaction was refluxed for 4 h. The reaction was cooled down to room temperature and concentrated under vacuum to afford the intermediate oil.

To the above oil dissolved in 30 mL Et_2O solution, Et_3N (0.95 mL, 6.8 mmol) was added, and the reaction was stirred at room temperature for 1 h. The reaction was quenched with 10 mL H_2O, extracted with ethyl acetate (3 × 30 mL), washed with 50 mL sat. brine and dried over Na_2SO_4, and concentrated to afford the reaction residue. The residue mixture was purified with silica gel chromatograph with hexane: ethyl acetate (4: 1) to afford the dimethyl (E)-4-oxopent-2-enedioate (**10**, 903 mg, 66%) as yellow solid.

^{1}H NMR (400 MHz, $CDCl_3$) δ 7.62 (d, *J* = 16.0 Hz, 1H), 6.97 (d, *J* = 16.0 Hz, 1H), 3.93 (s, 3H), 3.84 (s, 3H). ^{13}C NMR (100 MHz, $CDCl_3$) δ 182.31, 165.24, 161.11, 135.59, 134.25, 53.51, 52.74.

5-Methoxy-7,9-bis(methoxycarbonyl)-1H-pyrrolo[2,3-f]quinoline-2-carboxylic acid (12) The synthesis was prepared according to a modified literature procedure[6]

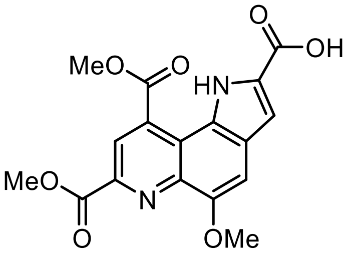

To a mixture of 2-carboxy-5-methoxy-1H-indol-6-aminium chloride (**9**, 485 mg, 2.0 mmol) and dimethyl (E)-4-oxopent-2-enedioate (**10**, 378 mg, 2.2 mmol) in 10 mL DCM, DIPEA (0.27 μL, 2.0 mmol) was added at room temperature, and then the reaction was stirred at room temperature overnight to generate the cyclization intermediate **11**. To the above reaction 10 mL dioxane and $Cu(OAc)_2$ (18 mg, 0.1 mmol) were added, then the reaction was bubbled with HCl gas at room temperature for 4 h and then bubbled with air for another 2 h for aromatization. During this time, a precipitate developed. After the reaction was completed, the resulting precipitate was filtered, washed with 5 mL DCM and 5 mL dioxane, dried under vacuum to afford 25-methoxy-7,9-bis(methoxycarbonyl)-1H-pyrrolo[2,3-f]quinoline-2-carboxylic acid (**12**, 526 mg, 68%) as yellow solid.

^{1}H NMR (500 MHz, DMSO-*d*$_6$) δ 13.44 (br, 1H), 11.93 (s, 1H), 8.70 (s, 1H), 7.59 (s, 1H), 7.28 (d, *J* = 2.2 Hz, 1H), 4.10 (s, 3H), 4.01 (s, 3H), 3.99 (s, 3H). ^{13}C NMR (125 MHz, DMSO-*d*$_6$) δ 168.20, 164.81, 162.00, 150.40, 143.41, 141.24, 132.42, 128.74, 127.20, 123.38, 123.18, 116.21, 108.23, 103.83, 55.94, 54.08, 52.84.

HRMS (ESI) m/z: $[M+1]^+$ calcd 359.0874, found 359.0873.

7,9-Bis(methoxycarbonyl)-4,5-dioxo-4,5-dihydro-1H-pyrrolo[2,3-f]quinoline-2-carboxylic acid (13) The synthesis was prepared according to a published literature procedure[7]

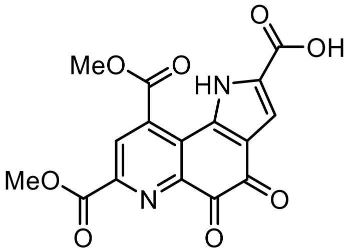

To a mixture of 5-methoxy-7,9-bis(methoxycarbonyl)-1H-pyrrolo[2,3-f]quinoline-2-carboxylic acid (**12**, 358 mg, 1.0 mmol) in 16 mL CH_3CN and 4 mL H_2O, CAN (2.49 g, 4.55 mmol) was added portion wise at 0 ºC. The reaction was stirred at 0 ºC for 1 h and adjusted pH 1.0 with 6 M HCl aq., followed by stirring at 0 ºC for 1 h. During this time, a precipitate developed. The resulting precipitate was filtered, washed with 10 mL CH_3CN, dried under vacuum to afford the 7,9-bis(methoxycarbonyl)-4,5-dioxo-4,5-dihydro-1H-pyrrolo[2,3-f]quinoline-2-carboxylic acid (**13**, 209 mg, 58%) as yellow solid.

^{1}H NMR (500 MHz, DMSO-d_6) δ 12.40 (br, 1H), 8.55 (s, 1H), 7.21 (s, 1H), 4.04 (s, 3H), 3.96 (s, 3H). ^{13}C NMR (125 MHz, DMSO-d_6) δ 177.28, 173.43, 166.79, 163.92, 160.99, 148.95, 145.58, 134.07, 133.24, 128.63, 128.17, 126.87, 125.09, 113.56, 54.31, 53.04.

HRMS (ESI) m/z: $[M+1]^+$ calcd. 359.0510, found 359.0511.

2-((2-(3-(but-3-yn-1-yl)-3H-diazirin-3-yl)ethyl)carbamoyl)-4,5-dioxo-4,5-dihydro-1H-pyrrolo[2,3-f]quinoline-7,9-dicarboxylic acid (PQQ8)

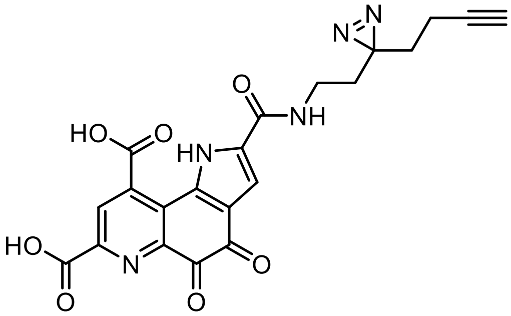

To a mixture of 7,9-bis(methoxycarbonyl)-4,5-dioxo-4,5-dihydro-1H-pyrrolo[2,3-f]quinoline-2-carboxylic acid (**13**, 39 mg, 0.1 mmol), PyBOP (52 mg, 0.1 mmol) and DIPEA (42 μL, 0.24 mmol) in 0.5 mL anhydrous DMF, 2-(3-(but-3-yn-1-yl)-3H-diazirin-3-yl)ethan-1-amine (14 mg, 0.1 mmol) was added under argon atmosphere, then the reaction was stirred at room temperature overnight. The reaction was quenched with 0.5 mL of 6 M HCl aq., and purified with preparative HPLC (C_{18}, from 10: 90 to 90: 10, 0.1% TFA in CH_3CN: 0.1% TFA in H_2O) and lyophilized to afford the dimethyl 2-((2-(3-(but-3-yn-1-yl)-3H-diazirin-3-yl)ethyl)carbamoyl)-4,5-dioxo-4,5-dihydro-1H-pyrrolo[2,3-f]quinoline-7,9-dicarboxylate.

To the above solution of dimethyl 2-((2-(3-(but-3-yn-1-yl)-3H-diazirin-3-yl)ethyl)carbamoyl)-4,5-dioxo-4,5-dihydro-1H-pyrrolo[2,3-f]quinoline-7,9-dicarboxylate in 0.5 mL THF and 0.5 mL H_2O, lithium hydroxide (27 mg, 1.15 mmol) was added under argon atmosphere, and the reaction was stirred at room temperature for 6 h. The reaction was quenched with 0.5 mL 6 M HCl aq., and further purified with preparative HPLC (BEH amide, HILIC column, from 95: 5 to 60: 40, CH_3CN: 10 mM $HCOONH_4$ in H_2O) and lyophilized to afford the 2-((2-(3-(but-3-yn-1-yl)-3H-diazirin-3-yl)ethyl)carbamoyl)-4,5-dioxo-4,5-dihydro-1H-pyrrolo[2,3-f]quinoline-7,9-dicarboxylic acid (**PQQ8**, 1.3 mg, 3 % yield for two steps) as yellow solid.

^{1}H NMR (500 MHz, DMSO-d_6) δ 8.54 (s, 1H), 8.40 (t, J = 5.6 Hz, 1H), 7.27 (s, 1H), 3.12 - 3.18 (m, 2H), 2.82 (t, J = 2.7 Hz, 1H), 2.01 (td, J = 7.4, 2.7 Hz, 2H), 1.61 - 1.66 (m, 4H). ^{13}C NMR (125 MHz, DMSO-d_6) δ 180.40, 173.70, 167.07, 165.97, 159.67, 147.25, 145.07, 136.79,

130.66, 130.48, 129.78, 125.53, 122.70, 109.83, 83.32, 71.95, 34.02, 32.14, 31.40, 27.41, 12.81.
HRMS (ESI) m/z: $[M-1]^+$ calcd 448.0898, found 448.0898.

2-(3-(But-3-yn-1-yl)-3H-diazirin-3-yl)acetic acid (PhotoX) The synthesis and spectroscopic data of the compound were in accordance with the reported literature[8]

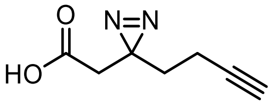

To a solution of 2-(3-(but-3-yn-1-yl)-3H-diazirin-3-yl)ethan-1-ol (100 mg, 0.72 mmol) in 6 mL acetone, 2 M Jones reagent (1.45 mL, 2.89 mmaol) was dropwise added at 0 ºC, and then the reaction was warmed up to room temperature and stirred at room temperature for 2 h. After the reaction was completed, it was quenched with 10 mL of 70% isopropanol, filtered with celite, washed with 10 mL acetone, and concentrated under vacuum. The residue mixture was purified with silica gel chromatography with hexane: ethyl acetate (4: 1) to afford the 2-(3-(but-3-yn-1-yl)-3H-diazirin-3-yl)acetic acid (**PhotoX**, 95 mg, 89%) as colorless oil.
^{1}H NMR (400 MHz, $CDCl_3$) δ 2.41 (s, 2H), 2.07 (td, J = 7.3, 2.6 Hz, 2H), 2.01 (t, J = 2.6 Hz, 1H), 1.80 (t, J = 7.3 Hz, 2H). ^{13}C NMR (100 MHz, $CDCl_3$) δ 175.36, 82.38, 69.53, 39.44, 31.88, 25.17, 13.17.
HRMS (ESI) m/z: $[M-1]^-$ calcd. 151.0513, found 151.052.

2. Biological Methods

2.1. Bacterial Culture Conditions

For pre-cultures, 5 mL LB media (Lysogeny broth, 10 g / L casein peptone, 5 g / L NaCl, 5 g / L yeast extract, pH 7.5) were inoculated with 50 µL bacterial glycerol stock and the culture grew at 37 ºC, 200 rpm for 10 – 12 h. 500 µL pre-cultures were used to inoculate in 50 mL LB media in 500 mL flaks and then the cultures grew at 37 ºC, 200 rpm for 10 – 12 h. Minimal medium[9] and Mineral salts medium (MSM)[10] were used for *E. coli* and *P. putida* KT2440 labeling, respectively. The preparation of those two media is depicted below (Table S1 – S4 for *E. coli* and Table S5 – S6 for *P. putida* KT440).

2.2. Bacterial Media Preparation

Table S1. Minimal medium for *E. coli* K-12, *E. coli* BL21 and *E. coli* BL21 (Tuner)

minimal medium content	
5% aspartate, pH7.5	25 mL
25× 18 amino acids mix	20 mL
50× M salts	10 mL
Leucine (4 mg/mL), pH7.5	5 mL
1M $MgSO_4$	1 mL
40% glucose	6.25 mL
Trace metals	0.1 mL

Fill it up to 500 mL with ddH_2O, and sterile filtration before use.

Table S2. 25× 18 amino acids mix

25 × 18 amino acids preparation		
Glutamic acid sodium salt	Aspartic acid	Lysine-HCl
Arginine-HCl	Histine-HCl-H_2O	Alanine
Proline	Glycine	Threonine
Serine	Glutamine	Asparagine-H_2O
Valine	Leucine	Isoleucine
Phenylalanine	Tryptophan	Methionine

5 g for each amino acid, then fill up to 1 L with ddH_2O.

Table S3. 50 × M salts

50 × mineral salts preparation	
Na_2HPO_4 (1.25M)	88.73 g
KH_2PO_4 (1.25 M)	85.1 g
NH_4Cl (2.5 M)	66.9 g
Na_2SO_4 (0.25 M)	17.8 g

Fill up to 500 mL with ddH_2O.

Table S4. 5000× trace metal

5000 × trace metal preparation	
$CaCl_2·2H_2O$	2.94 g (20 mM)
$MnCl_2·4H_2O$	1.98 g (10 mM)
$ZnSO_4·7H_2O$	2.87 g (10 mM)
$CoCl_2·6H_2O$	476 mg (2 mM)

$CuCl_2$	269 mg (2 mM)
$NiCl_2$	259 mg (2 mM)
$NaMoO_4 \cdot 2H_2O$	484 mg (2 mM)
Na_2SeO_3	346 mg (2 mM)
H_3BO_3	124 mg (2 mM)
$FeCl_3$	8.11 g (50 mM)

Fill up to 1000 mL with ddH_2O.

Mineral Salts Medium (MSM) for *P. putida* KT2440

100 mL 10×MSM bulk medium and 2 mL 1×trace metals were combined, which was then filled up to 1000 mL with dd H_2O and sterilized by filtration. The medium was completed by adding 20 mM sterile glucose.

Table S5. MSM bulk medium

10 × MSM bulk medium preparation	
K_2HPO_4	22.7g (0.130 mM)
KH_2PO_4	9.5g (0.0698 mM)
$(NH_4)_2SO_4$	6.7g (0.0507 mM)

Fill up to 1000 mL with ddH_2O, and adjust PH 7.

Table S6. Trace metals for *P. putida* KT2440

1 × trace metals preparation	
$Na_2EDTA \cdot 2H_2O$	637 mg (0.0171 mM)
$MgSO_4 \cdot 7H_2O$	6.0 g (0.2435 mM)
$FeSO_4 \cdot 7H_2O$	250 mg (0.009 mM)
$ZnSO_4 \cdot 7H_2O$	100 mg (0.00348 mM)
$CaCl_2 \cdot 2H_2O$	50 mg (0.00340 mM)
$NaMoO_4 \cdot 2H_2O$	10 mg (0.000413 mM)
$CuSO_4 \cdot 5H_2O$	10 mg (0.0004 mM)
$CoCl_2 \cdot 6H_2O$	20 mg (0.000841 mM)
$MnSO_4 \cdot H_2O$	52 mg (0.00308 mM)

Fill up to 100 mL with ddH_2O.

2.3. HepG2 Cell Culture Condition

HepG2 cells were cultured in Gibco Dulbecco's Modified Eagle Medium (DMEM, containing 1g / L D-Glucose and L-glutamine) supplemented with 10% fetal bovine serum (FBS, Sigma Aldrich) at 37 ºC in a CO_2 incubator. Accutase (Merk) was used for cell detachment. Cell numbers were determined by mixing cell suspension with trypan blue (1: 1), then counted in a *Neubauer* chamber.

2.4. Gel-Based Fluorescent in situ Labeling and Lysate Labeling

Gel-based fluorescent in situ labeling of *E. coli* and *P. putida*

Pre-cultures of bacteria (*E. coli* or *P. putida*) were inoculated in 50 mL LB media and then the cultures grew at 37 ºC, 200 rpm for 10 h or 12 h to reach the early stationary phase. The cells were harvested with centrifugation (6000 g, 10 min, 4 ºC) and washed with minimal medium / mineral salts medium (2 × 50 mL, 6000 g, 10 min, 4 ºC). The pellets were resuspended in 50 mL minimal medium and the resuspension was incubated at 37 ºC, 200 rpm for 4 h starvation,

followed by cells harvest with centrifugation (6000 g, 10 min, 4 ^{o}C) and wash with minimal medium (2 × 50 mL, 6000 g, 10 min, 4 ^{o}C) (The starvation step was only implemented in *E. coli*, but can be omitted depending on the purpose of specific *E. coli* strain and *P. putida*). The pellets were resuspended in minimal medium / mineral salts medium to obtain an OD_{600} = 40 cell suspension. The 200 µL bacterial culture of OD_{600} = 40 was treated with 2 µL DMSO, **PQQ** and **PQQ** probes (final concentration: 50 µM) at 37 ^{o}C, 200 rpm for 2 h or 1 h, for *E. coli* and *P. putida* respectively. The cells were irradiated under UV 365 nm for 5 min with cooling underneath; while non-UV samples underwent the same procedure without UV irradiation. The cells were harvested with centrifugation (6000 g, 10 min, 4 ^{o}C) and washed with cold PBS (2 × 400 µL, 6000 g, 10 min, 4 ^{o}C). The cell pellets were resuspended in 200 µL SDS (0.5% in PBS), and lysed with sonication (70% intensity, 30 s). The clear supernatant was clarified with centrifugation (21000 g, r.t., 30 min) and the protein concentration was determined with BCA assay (Roti Quant, Roth). After the protein concentration was adjusted as 2.23 mg / mL, 50 µL sample was subjected to a click reaction using 5-TAMRA-Azide (1 µL, 10 mM in DMSO), tris(benzyltriazolylmethyl)amine (TBTA, 3 µL, 1.667 mM in ^{t}BuOH: DMSO = 4: 1), tris(2-carboxyethyl)phosphine (TCEP, 1 µL, 100 mM in H_2O) and $CuSO_4$ (1 µL, 50 mM in H_2O) at room temperature for 1 h. The reaction was quenched with 56 µL 2 × Laemmli buffer (63 mM Tris-HCl, 10% glycerol, 139 mM SDS, 0.0025% bromophenol blue, 5% 2-mercaptoethanol) and heated at 95 ^{o}C for 5 min. The samples were loaded on an SDS-PAGE gel (12.5% or 15% acrylamide, each lane with 20 µL sample). Fluorescence was recorded with Fujifilm Las-400 luminescent image analyzer equipped with a Fujinon VRF43LMD3 lens and a 575DF20 filter. Coomassie Brilliant Blue was used to monitor the total protein content.

Summary of labeling conditions for all strains

E. coli K-12:

Pre-cultures of *E. coli* K-12 were inoculated in 50 mL LB media and then the culture grew at 37 ^{o}C, 200 rpm for 10 h to reach the early stationary phase. The cells were harvested with centrifugation (6000 g, 10 min, 4 ^{o}C) and washed with minimal medium (2 × 50 mL, 6000 g, 10 min, 4 ^{o}C). The pellets were resuspended in 50 mL minimal medium and the resuspension was incubated at 37 ^{o}C, 200 rpm for 4 h starvation, followed by cells harvest with centrifugation (6000 g, 10 min, 4 ^{o}C) and wash with minimal medium (2 × 50 mL, 6000 g, 10 min, 4 ^{o}C) (The starvation step can be omitted depending on the experimental purpose). The pellets were resuspended in minimal medium to obtain an OD_{600} = 40 cell suspension. The 200 µL bacterial culture of OD_{600} = 40 was treated with DMSO, **PQQ** and **PQQ** probes (final concentration: 50 µM) at 37 ^{o}C, 200 rpm for 2 h.

E. coli BL21 (Tuner_YliI or Tuner_PedH):

Pre-cultures of *E. coli* BL21 were inoculated in 50 mL LB media containing antibiotics (ampicillin for Tuner_YliI and kanamycin for Tuner_PedH) and then the culture grew at 37 ^{o}C, 200 rpm for 10 h or 12 h to reach the early stationary phase. The cells were harvested with centrifugation (6000 g, 10 min, 4 ^{o}C) and washed with minimal medium (2 × 50 mL, 6000 g, 10 min, 4 ^{o}C). The pellets were resuspended in minimal medium to obtain a OD_{600} = 40 cell suspension. The 200 µL bacterial culture of OD_{600} = 40 was treated with DMSO, **PQQ** and **PQQ** probes (final concentration: 50 µM) at 37 ^{o}C, 200 rpm for 2 h.

P. putida KT2440

Pre-cultures of *P. putida* KT2440 were inoculated in 50 mL LB media and then the culture grew at 37 ^{o}C, 200 rpm for 12 h to reach the early stationary phase. The cells were harvested with

centrifugation (6000 g, 10 min, 4ºC) and washed with mineral salts medium (2 × 50 mL, 6000 g, 10 min, 4 ºC). The pellets were resuspended in mineral salts medium to obtain a OD_{600} = 40 cell suspension. The 200 µL bacterial culture of OD_{600} = 40 was treated with DMSO, **PQQ** and **PQQ** probes (final concentration: 50 µM) at 37 ºC, 200 rpm for 1 h.

Gel-based fluorescent *E. coli* K-12 lysate labeling (with spiked proteins)
Pre-culture of *E. coli* K-12 was inoculated in 50 mL LB media and then the culture grew at 37ºC, 200 rpm for 10 h to reach the early stationary phase. The cells were harvested with centrifugation (6000 g, 10 min, 4ºC) and washed with cold PBS (2 × 50 mL, 6000 g, 10 min, 4ºC). The pellets were resuspended in cold HEPES buffer (25 mM HEPES, 150 mM NaCl) to obtain an $OD6_{00}$ = 20 cell suspension. The cell pellets were lysed with sonication (70% intensity, 10 × 30 s, 4ºC). The clear supernatant was clarified with centrifugation (21000 g, 30 min, 4ºC) and the protein concentration was determined with BCA assay (Roti Quant, Roth). After the K-12 lysate protein concentration was adjusted as 2.23 mg / mL, the purified recombinant proteins were spiked in. 200 µL K-12 lysate and the samples were treated with 2 µL DMSO, **PQQ**, **PQQ4** (final concentration: 50 µM), or a competition condition (50 µM **PQQ4** + 1000 µM **PQQ**), then the samples were incubated at room temperature for 1 h. The cells were irradiated for 5 min under UV 365 nm with cooling underneath. Non-UV samples underwent the same procedure without UV irradiation. The 50 µL sample was subjected to a click reaction using 5-TAMRA-Azide (1 µL, 10 mM in DMSO), tris(benzyltriazolylmethyl)amine (TBTA, 3 µL, 1.667 mM in ^{t}BuOH: DMSO = 4: 1), tris(2-carboxyethyl)phosphine (TCEP, 1 µL, 100 mM in H_2O) and $CuSO_4$ (1 µL, 50 mM in H_2O) at room temperature for 1 h. The reaction was quenched with 56 µL 2 × Laemmli buffer (63 mM Tris-HCl, 10% glycerol, 139 mM SDS, 0.0025% bromophenol blue, 5% 2-mercaptoethanol) and heated at 95 ºC for 5 min. The samples were loaded on an SDS-PAGE gel (12.5% or 15% acrylamide, each lane with 20 µL sample). Fluorescence was recorded with Fujifilm Las-400 luminescent image analyzer equipped with a Fujinon VRF43LMD3 lens and a 575DF20 filter. Coomassie Brilliant Blue was used to monitor the total protein content.

Gel-based fluorescent in situ labeling of HepG2
For preculture, 1 mL of 2 million HepG2 cells were cultivated in 5 mL DMEM medium supplemented with 10% FCS in a T25 flask at 37 ºC in a CO_2 incubator. The culture was changed medium twice per week and the cells were harvested with centrifugation (500 g, 5 min, r.t.) when they reached up to 80% confluence. The cell pellets were resuspended in 10 mL DMEM medium supplemented with 10% FCS in a T75 flask and the cells were harvested with centrifugation (500 g, 5 min, r.t.) when they reached up to 80% confluence. The cells were resuspended in 20 mL DMEM medium supplemented with 10% FCS in a T175 cell culture flask. The cells were harvested with centrifugation (500 g, 5 min, r.t.) when they reached up to 80% confluence, which were then used for in situ labeling. The 2.5 million HepG2 cells were seeded in 5 mL DMEM medium into 6-cm dishes and cultivated at 37 ºC in a CO_2 incubator till the 80% confluence. The medium was aspirated, and the cells were washed with 5 mL warm PBS, followed by 5 mL serum-free DMEM medium. The HepG2 cells in 2 mL serum-free medium were treated with 5 µL DMSO, **PQQ**, **PQQ** probes and **PhotoX** (**2-(3-(But-3-yn-1-yl)-3H-diazirin-3-yl)acetic acid**, final concentration: 50 µM, see the synthesis part, and used for exploring the photo-cross-linker off-targets binding) at 37 ºC for 16 h in a CO_2 incubator. The cells were irradiated for 40 min under UV 365 nm with cooling underneath. The medium was aspirated and the cells were washed with cold PBS (2 × 2 mL). The cells were added 0.15 mL RIPA buffer (25 mM Tris HCl, 150 mM NaCl, 1% NP-40, 1% sodium deoxycholate, 0.1% SDS,

PH7.6), and lysed for 10 min on ice, followed by sonication (30% intensity, 30 s, r.t.). The clear supernatant was clarified with centrifugation (21000 g, r.t., 30 min) and the protein concentration was determined with BCA assay (Roti Quant, Roth). After the protein concentration was adjusted as 2.23 mg / mL, 50 μL sample was subjected to a click reaction using 5-TAMRA-Azide (1 μL, 10 mM in DMSO), tris(benzyltriazolylmethyl)amine (TBTA, 3 μL, 1.667 mM in ^{t}BuOH: DMSO = 4: 1), tris(2-carboxyethyl)phosphine (TCEP, 1 μL, 100 mM in H_2O) and $CuSO_4$ (1 μL, 50 mM in H_2O) at room temperature for 1 h. The reaction was quenched with 56 μL 2 × LaemmLi buffer (63 mM Tris-HCl, 10% glycerol, 139 mM SDS, 0.0025% bromophenol blue, 5% 2-mercaptoethanol) and heated at 95 ºC for 5 min. The samples were loaded on an SDS-PAGE gel (12.5% or 15% acrylamide, each lane with 20 μL sample). Fluorescence was recorded with Fujifilm Las-400 luminescent image analyzer equipped with a Fujinon VRF43LMD3 lens and a 575DF20 filter. Coomassie Brilliant Blue was used to monitor the total protein content.

2.5. Preparative Labeling Analysis

In situ labeling of *E. coli* K-12 and cell lysis

For pre-cultures, 5 mL LB media were inoculated with 50 μL *E. coli* K-12 glycerol stock and the culture grew at 37 ºC, 200 rpm for 10 h. For each biological replicate, 500 μL pre-culture of *E. coli* K-12 was inoculated in 50 mL LB media and then the culture grew at 37 ºC, 200 rpm for 10 h to reach the early stationary phase. The cells were harvested with centrifugation (6000 g, 10 min, 4 ºC) and washed with minimal medium (2 × 50 mL, 6000 g, 10 min, 4 ºC). The pellets were resuspended in 50 mL minimal medium and the resuspension was incubated at 37 ºC, 200 rpm for 4 h starvation, followed by cells harvest with centrifugation (6000 g, 10 min, 4 ºC) and wash with minimal medium (2 × 50 mL, 6000 g, 10 min, 4 ºC) (The starvation step can be omitted depending on the experimental purpose). The pellets were resuspended in minimal medium to obtain an OD_{600} = 40 cell suspension. The 200 μL bacterial culture of OD_{600} = 40 was treated with 2 μL DMSO, **PQQ** and **PQQ** probes (final concentration: 50 μM) at 37 ºC, 200 rpm for 2 h. Regarding on the in situ competition experiment, 200 μL bacterial culture of OD_{600} = 40 was treated with DMSO and **PQQ** (for competition samples) and pre-incubated at 37 ºC, 200 rpm for 30 min, which were subsequently treated with **PQQ** (for negative control) and **PQQ4** (final concentration: 50 μM **PQQ**, 50 μM **PQQ4**, 50 μM **PQQ4** + 500 μM **PQQ**, and 50 μM **PQQ4** + 750 μM **PQQ**) at 37 ºC, 200 rpm for 2 h. The cells were irradiated for 5 min under UV 365 nm with cooling underneath, while non-UV samples underwent the same procedure without UV irradiation. The cells were harvested with centrifugation (6000 g, 10 min, 4 ºC) and washed with cold PBS (2 × 400 μL, 6000 g, 10 min, 4 ºC). The cell pellets were resuspended in 200 μL SDS (0.5% in PBS), and lysed with sonication (70% intensity, 30 s). The clear supernatant was clarified with centrifugation (21000 g, r.t., 30 min) and the protein concentration was determined with BCA assay (Roti Quant, Roth). This in situ labeling of *E. coli* K-12 was performed in n = 3 or 4 biological replicates.

In situ labeling of *E. coli* BL21 (Tuner_YliI and Tuner_PedH) and cell lysis

For pre-cultures, 5 mL LB media containing antibiotics were inoculated with 50 μL *E. coli* BL21 (ampicillin for Tuner_YliI, kanamycin for Tuner_PedH) glycerol stock and the culture grew at 37 ºC, 200 rpm for 10 h. For each biological replicate, 500 μL pre-culture of *E. coli* BL21 (Tuner_YliI and Tuner_PedH) was inoculated in 50 mL LB media containing antibiotics and then the culture grew at 37 ºC, 200 rpm for 10 h to reach the early stationary phase. The cells were harvested with centrifugation (6000 g, 10 min, 4 ºC) and washed with minimal medium containing antibiotics (2 × 50 mL, 6000 g, 10 min, 4 ºC). The pellets were resuspended in

minimal medium containing antibiotics to obtain an OD_{600} = 40 cell suspension. The 200 µL bacterial culture of OD_{600} = 40 was treated with 2 µL DMSO, **PQQ** and **PQQ** probes (final concentration of 50 µM) at 37 ºC, 200 rpm for 2 h. The cells were irradiated for 5 min under UV 365 nm with cooling underneath. The cells were harvested with centrifugation (6000 g, 10 min, 4 ºC) and washed with cold PBS (2 × 400 µL, 6000 g, 10 min, 4 ºC). The cell pellets were resuspended in 200 µL SDS (0.5% in PBS), and lysed with sonication (70% intensity, 30 s). The clear supernatant was clarified with centrifugation (21000 g, r.t., 30 min) and the protein concentration was determined with BCA assay (Roti Quant, Roth). This in situ labeling of *E. coli* BL21 (Tuner) was performed in n = 4 biological replicates.

In situ labeling of *P. putida* KT2440

For pre-cultures, 5 mL LB media were inoculated with 50 µL *P. putida* KT2440 glycerol stock and the culture grew at 37 ºC, 200 rpm for 12 h. For each biological replicate, 500 µL pre-culture of *P. putida* KT2440 was inoculated in 50 mL LB media and then the culture grew at 37 ºC, 200 rpm for 12 h to reach the early stationary phase. The cells were harvested with centrifugation (6000 g, 10 min, 4 ºC) and washed with mineral salts medium (2 × 50 mL, 6000 g, 10 min, 4 ºC). The pellets were resuspended in mineral salts medium to obtain an OD_{600} = 40 cell suspension. The 200 µL bacterial culture of OD_{600} = 40 was treated with 2 µL DMSO, **PQQ** and **PQQ** probes (final concentration: 50 µM) at 37 ºC, 200 rpm for 2 h. The cells were irradiated for 5 min under UV 365 nm with cooling underneath. The cells were harvested with centrifugation (6000 g, 10 min, 4ºC) and washed with cold PBS (2 × 400 µL, 6000 g, 10 min, 4 ºC). The cell pellets were resuspended in 200 µL SDS (0.5% in PBS), and lysed with sonication (70% intensity, 30 s). The clear supernatant was clarified with centrifugation (21000 g, 30 min, r.t.) and the protein concentration was determined with BCA assay (Roti Quant, Roth). This in situ labeling of *P. putida* KT2440 was performed in n = 4 biological replicates.

In situ labeling of HepG2 cells

For preculture, 1 mL of 2 million HepG2 cells were cultivated in 5 mL DMEM medium supplemented with 10% FCS in a T25 flask at 37 ºC in a CO_2 incubator. The culture was changed medium twice per week and the cells were harvested with centrifugation (500 g, 5 min, r.t.) when they reached up to 80% confluence. The cell pellets were resuspended in 10 mL DMEM medium supplemented with 10% FCS in a T75 flask and the cells were harvested with centrifugation (500 g, 5 min, r.t.) when they reached up to 80% confluence. The cells were resuspended in 20 mL DMEM medium supplemented with 10% FCS in a T175 cell culture flask. The cells were harvested with centrifugation (500 g, 5 min, r.t.) when they reached up to 80% confluence, which were then used for in situ labeling. For each biological replicate, 2.5 million HepG2 cells were seeded in 5 mL DMEM medium into 6-cm dishes and cultivated at 37 ºC in a CO_2 incubator till the 80% confluence. The medium was aspirated, and the cells were washed with warm PBS (2 × 5 mL), followed by serum-free DMEM medium (2 × 5 mL). The HepG2 cells in 2 mL serum-free medium were treated with 5 µL DMSO, **PQQ**, **PQQ** probes and **PhotoX** (final concentration: 50 µM; **PhotoX** see the synthesis part, and used for exploring the photo-cross-linker off-targets binding) at 37 ºC for 16 h in a CO_2 incubator. The cells were irradiated for 40 min under UV 365 nm with cooling underneath. The medium was aspirated and the cells were washed with cold PBS (2 × 2 mL). The cells were added 0.15 mL RIPA buffer (25 mM Tris HCl, 150 mM NaCl, 1% NP-40, 1% sodium deoxycholate, 0.1% SDS, PH7.6), and lysed for 10 min on ice, followed by sonication (30% intensity, 30 s, r.t.). The clear supernatant was clarified with centrifugation (21000 g, 30 min, r.t.) and the protein

concentration was determined with BCA assay (Roti Quant, Roth). This in situ labeling of HepG2 was performed in n = 4 biological replicates.

Click, enrichment and digestion

All the solvents used were LC-MS grade. The protein concentration from above was adjusted as 2.23 mg / mL, then 45 µL of each sample was transferred to a 96-well plate (Greiner, round-bottom). A click reaction mixture was prepared by mixing 0.6 µL biotin azide (20 mM in DMSO), 0.25 µL tris(benzyltriazolylmethyl)amine (TBTA, 16.67 mM in ^{t}BuOH: DMSO = 1:1), 0.6 µL tris(2-carboxyethyl)phosphine (TCEP, 100 mM in H_2O) and 1.2 µL $CuSO_4$ (50 mM in H_2O). Each sample was added 2.65 µL click mixture, and incubated at room temperature and 950 rpm for 90 min in an Eppendorf ThermoMixer C. The reaction was quenched with 65 µL urea (8 M in H_2O) containing TCEP (10 mM in H_2O, for reduction) and iodoacetamide (IAA, 20 mM in H_2O, for alkylation) and incubated at room temperature and 950 rpm for 15 min. Then 2 µL dithiothreitol (DTT, 500 mM in H_2O) was added to quench the excessive IAA. 10 µL washed hydrophobic / hydrophilic carboxylate-coated magnetic beads (1: 1 mixture, Cytiva, washed with 3 × 1 mL H_2O) were added to each sample. Then 175 µL EtOH was added to each sample to precipitate the proteins onto the beads. The following steps were performed using an automated robot (Hamilton Microlab Prep, Hamilton). The plate was incubated at room temperature and 500 rpm for 5 min. Then the plate was placed on a 96-well ring magnet (Alpaqua, *Manum FLX*), and the supernatant was aspirated without removing any beads. The samples were treated with 180 µL EtOH (80%), shaken at room temperature and 800 rpm for 1 min, placed onto a magnet, and the supernatant was aspirated. The 80% EtOH wash step was repeated twice. Then the samples were added 180 µL acetonitrile, shaken at room temperature and 800 rpm for 1 min, placed onto a magnet, and the supernatant was aspirated. The proteins bound to the carboxylate beads were eluted by adding 75 µL SDS (0.2% in PBS), and then incubated at 40 ^{o}C and 800 rpm for 5 min. The plate was placed onto the magnet, followed by transferring supernatant into new wells. The elution and transfer step were repeated once. Next, 50 µL washed streptavidin magnetic beads (New England Biolabs, washed with 3 × 0.2% SDS in PBS) were added to each sample. The plate was sealed with plastic foil and incubated at room temperature and 800 rpm for 1 h in an Eppendorf ThermoMixer C to enrich the labeled proteins onto the beads. Afterwards, the plate was carried out with Hamilton robot. The plate was placed onto a magnet, and the supernatant was aspirated. The samples were added 180 µL NP-40 (0.1% in PBS), incubated at room temperature and 800 rpm for 1 min. The plate was placed onto a magnet, and the supernatant was aspirated. This NP-40 wash step was repeated twice. The samples were added 180 µL urea (6 M in H_2O), incubated at room temperature and 800 rpm for 1 min. The plate was placed onto a magnet, and the supernatant was aspirated. The Urea wash step was repeated once. The samples were added 200 µL water, incubated at room temperature and 800 rpm for 1 min. The plate was placed onto a magnet, and the supernatant was aspirated. The water wash step was repeated twice. The enriched proteins were added 100 µL triethylammonium bicarbonate (TEAB, 50 mM in H_2O) containing 1.5 µL trypsin (0.5 µg / µL, Progema). The plate was sealed with an aluminum foil and proteins were digested at 37 ^{o}C at 900 rpm for at least 16 h in an Eppendorf ThermoMixer C. Next, the plate was handled with Hamilton robot for sample transfer. The plate was placed onto the magnet and the supernatants were transferred to new wells. The samples were added 50 µL FA (3%), incubated at 40 ^{o}C and 800 rpm for 5 min. The plate was placed onto a magnet and the supernatants were transferred to the above related vials for following desalting. The desalting step was underwent using handmade STAGE (STop And Go Extraction) tips containing 2-layer styrenedivinylbenzene-reverse phase sulfonate

(SDB-RPS, Empore). The STAGE tips were first equilibrated with 170 µL wash buffer 1 (1% TFA in ^{i}PrOH) and centrifuged at 800 g for 10 min. The digested peptides were loaded onto the tips and centrifuged at 500 g for 10 min. The wash step with buffer 1 was repeated twice. Then the peptides were washed with 170 µL wash buffer 2 (0.2% TFA in H_2O) and centrifuge at 800 g for 10 min. Finally, the peptides were eluted with 50 µL elution buffer (1% ammonia in 80% CH_3CN) by centrifugation at 800 g for 10 min. The eluted peptides were collected with LoBind eppis (Eppendorf) and dried using a concentrator plus (Eppendorf) at 40 ºC. Then the peptides were reconstituted in 30 µL FA (1%) and 5 µL of each sample was injected on a timsTOF pro mass spectrometer (Bruker) in data-independent acquisition (DIA) mode for LC-MS/MS measurements. The bacterial samples (*E. coli* and *P. putida*) were applied with 60 min gradient method (Table S7), and human samples of HepG2 were measured with 90 min gradient method (Table S8). The MS samples were performed in n = 3 or 4 biological replicates.

2.6. Full Proteome Analysis

E. coli K-12_labled at inoculation
For pre-cultures, 5 mL LB media were inoculated with 50 µL *E. coli* K-12 glycerol stock and the culture grew at 37 ºC, 200 rpm for 10 h. For each biological replicate, 50 µL pre-culture of *E. coli* K-12 was inoculated in 5 mL minimal medium, treated with 5 µL DMSO, **PQQ** (final concentration: 0.5 µM and 50 µM) and the culture grew at 37 ºC, 200 rpm for 10 h. The cells were harvested with centrifugation (6000 g, 10 min, 4 ºC) and washed with cold PBS (2 × 5 mL, 6000 g, 10 min, 4 ºC). The cell pellets were resuspended in 500 µL SDS (0.5% in PBS), and lysed with sonication (70% intensity, 30 s). The clear supernatant was clarified with centrifugation (21000 g, r.t., 30 min) and the protein concentration was determined with BCA assay (Roti Quant, Roth). This full proteome experiments were performed in n = 4 biological replicates.

P. putida KT2440_labeled at inoculation
For pre-cultures, 5 mL LB media were inoculated with 50 µL *P. putida* KT2440 glycerol stock and the culture grew at 37 ºC, 200 rpm for 12 h. For each biological replicate, 50 µL pre-culture of *P. putida* KT2440 was inoculated in 5 mL mineral salts medium, treated with 5 µL DMSO, **PQQ** (final concentration: 0.5 µM and 50 µM) and the culture grew at 37 ºC, 200 rpm for 12 h. The cells were harvested with centrifugation (6000 g, 10 min, 4 ºC) and washed with cold PBS (2 × 5 mL, 6000 g, 10 min, 4 ºC). The cell pellets were resuspended in 500 µL SDS (0.5% in PBS), and lysed with sonication (70% intensity, 30 s). The clear supernatant was clarified with centrifugation (21000 g, r.t., 30 min) and the protein concentration was determined with BCA assay (Roti Quant, Roth). This full proteome experiments were performed in n = 4 biological replicates.

Cleanup and digestion
All the solvents used were LC-MS grade. The protein concentration from above was adjusted as 0.4 mg/mL and then 50 µL of each sample was transferred to a 96-well plate (Greiner, round-bottom). The 3 µL mixture of TCEP (500 mM in H_2O): 500 mM IAA (500 mM in H_2O) = 1: 1 was added to each sample for reduction and alkylation. The plate was incubated at room temperature and 950 rpm for 15 min in an Eppendorf ThermoMixer C. And 2 µL DTT (500 mM in H_2O) was added to quench excessive IAA and incubate at room temperature and 950 rpm for 5 min in an Eppendorf ThermoMixer C. 10 µL washed hydrophobic / hydrophilic carboxylate-coated magnetic beads (1: 1 mixture, Cytiva, washed with 3 × 1 mL H_2O) were added to each sample. Then 150 µL EtOH was added to each sample to precipitate the

proteins onto the beads. The following steps were performed using an automated robot (Hamilton Microlab Prep, Hamilton). The plate was incubated at room temperature and 500 rpm for 5 min. Then the plate was placed on a 96-well ring magnet (Alpaqua, *Manum FLX*), and the supernatant was aspirated without removing any beads. The samples were treated with 180 µL EtOH (80%), shaken at room temperature and 800 rpm for 1 min, placed onto a magnet, and the supernatant was aspirated. The 80% EtOH wash step was repeated twice. Then the samples were added 180 µL acetonitrile, shaken at room temperature and 800 rpm for 1 min, placed onto a magnet, and the supernatant was aspirated. The proteins on beads were added 100 µL triethylammonium bicarbonate (TEAB, 50 mM in H_2O) containing 0.4 µL trypsin (0.5 µg / µL, Progema). The plate was sealed with an aluminum foil and the samples were digested at 37 ºC at 900 rpm for at least 16 h in an Eppendorf ThermoMixer C. Next, the plate was handled with Hamilton robot for sample transfer. The plate was placed onto the magnet and the supernatants were transferred to new wells. The samples were added 50 µL FA (3%), incubated at 40 ºC and 800 rpm for 5 min. The plate was placed onto a magnet and the supernatants were transferred to the above related vials for desalting. The desalting step was underwent using handmade STAGE tips containing 2-layer styrenedivinylbenzene-reverse phase sulfonate (SDB-RPS, Empore). The STAGE tips were first equilibrated with 170 µL wash buffer 1 (1% TFA in ^{i}PrOH) and centrifuged at 800 g for 10 min. The digested peptides were loaded onto the tips and centrifuged at 500 g for 10 min. The wash step with buffer 1 was repeated twice. Then the peptides were washed with 170 µL wash buffer 2 (0.2% TFA in H_2O) and centrifuge at 800 g for 10 min. Finally, the peptides were eluted with 50 µL elution buffer (1% ammonia in 80% CH_3CN) by centrifugation at 800 g for 10 min. The eluted peptides were collected with LoBind eppis (Eppendorf) and dried using a concentrator plus (Eppendorf) at 40 ºC. Then the peptides were reconstituted in 120 µL FA (1%) and 2 µL of each sample was injected on a timsTOF pro mass spectrometer (Bruker) in data-independent acquisition (DIA) mode for LC-MS/MS measurements. and measured with 90 min gradient method (Table S8) on LC-MS. The MS samples were performed in n = 4 biological replicates.

2.7. MS/MS Workflow

E. coli K-12 lysate

For pre-cultures, 5 mL LB media were inoculated with 50 µL *E. coli* K-12 glycerol stock and the culture grew at 37 ºC, 200 rpm for 10 h. The 500 µL pre-culture of *E. coli* K-12 was inoculated 50 mL LB media and then the culture grew at 37 ºC, 200 rpm for 10 h. The cells were harvested with centrifugation (6000 g, 10 min, 4 ºC) and washed with cold PBS (2 × 50 mL, 6000 g, 10 min, 4 ºC). The pellets were resuspended in cold HEPES buffer (25 mM HEPES, 150 mM NaCl) to obtain an $OD6_{00}$ = 20 cell suspension. The cell pellets were lysed with sonication (70% intensity, 10 × 30 s, 4 ºC) and the clear supernatant was clarified with centrifugation (21000 g, 4 ºC, 30 min). The protein concentration was determined with BCA assay (Roti Quant, Roth). The *E. coli* K-12 lysate protein concentration was adjusted as 4.01 mg / mL. For each biological replicate, 87.3 µL *E. coli* K-12 lysate (final concentration: 100 µM, calculated based on the average size of *E. coli* K-12 proteome) was treated with 20-fold molar excess **PQQ** and 20-fold molar excess $CaCl_2$ (final concentration of both **PQQ** and $CaCl_2$: 2000 µM) as treatments; *E. coli* K-12 lysate was treated with $CaCl_2$ as negative controls. Then all the samples (final volume: 100 µL) were incubated at 37 ºC for 4 h in an Eppendorf ThermoMixer. In the experiment, the high concentration of **PQQ** was saturated in the reaction. The MS/MS samples were performed in n = 4 biological replicates.

Recombinant RuvB

For each biological replicate, 20.1 µL purified recombinant RuvB (final concentration: 100 µM in 25 mM HEPES and 150 mM NaCl, pH 7.5) was treated with 20-fold molar excess **PQQ** (final concentration: 2000 µM) or 20-fold molar excess **PQQ** and 20-fold molar excess $MgCl_2$ (final concentration of both: 2000 µM) as treatments; RuvB was treated with 20-fold molar excess $MgCl_2$ (final concentration: 2000 µM) as controls. Then all the samples (final volume: 50 µL) were incubated at 30 ºC for 1 h in an Eppendorf ThermoMixer. The MS samples were performed in n = 4 biological replicates.

Cleanup and digestion
All the solvents used were LC-MS grade. The protein concentration from above was adjusted as 0.4 mg / mL and then 50 µL of each sample was transferred to a 96-well plate (Greiner, round-bottom). The 3 µL mixture of TCEP (500 mM in H_2O): IAA (500 mM in H_2O) = 1: 1 was added to each sample for reduction and alkylation. The plate was incubated at room temperature and 950 rpm for 15 min in an Eppendorf ThermoMixer C. And 2 µL DTT (500 mM in H_2O) was added to quench excessive IAA and incubate at room temperature and 950 rpm for 5 min. 10 µL washed hydrophobic / hydrophilic carboxylate-coated magnetic beads (1: 1 mixture, Cytiva, washed with 3 × 1 mL H_2O) were added to each sample. Then 150 µL EtOH was added to each sample to precipitate the proteins onto the beads. The following steps were performed using an automated robot (Hamilton Microlab Prep, Hamilton). The plate was incubated at room temperature and 500 rpm for 5 min. Then the plate was placed on a 96-well ring magnet (Alpaqua, *Manum FLX*), and the supernatant was aspirated without removing any beads. The samples were treated with 180 µL EtOH (80%), shaken at room temperature and 800 rpm for 1 min, placed onto a magnet, and the supernatant was aspirated. The 80% EtOH wash step was repeated twice. Then the samples were added 180 µL acetonitrile, shaken at room temperature and 800 rpm for 1 min, placed onto a magnet, and the supernatant was aspirated. The proteins on beads were added 100 µL triethylammonium bicarbonate (TEAB, 50 mM in H_2O) containing 0.4 µL trypsin (0.5 µg / µL, Progema). The plate was sealed with an aluminum foil and proteins were digested at 37 ºC at 900 rpm for at least 16 h in an Eppendorf ThermoMixer C. Next, the plate was handled with Hamilton robot for sample transfer. The plate was placed onto the magnet and the supernatants were transferred to new wells. The samples were added 50 µL FA (3%), incubated at 40 ºC and 800 rpm for 5 min. The plate was placed onto a magnet and the supernatants were transferred to the above related vails for desalting. The desalting step was underwent using handmade STAGE tips containing 2-layer styrenedivinylbenzene-reverse phase sulfonate (SDB-RPS, Empore). The STAGE tips were first equilibrated with 170 µL wash buffer 1 (1% TFA in ^{i}PrOH) and centrifuged at 800 g for 10 min. The digested peptides were loaded onto the tips and centrifuged at 500 g for 10 min. The wash step with buffer 1 was repeated twice. Then the peptides were washed with 170 µL wash buffer 2 (0.2% TFA in H_2O) and centrifuge at 800 g for 10 min. Finally, the peptides were eluted with 50 µL elution buffer (1% ammonia in 80% CH_3CN) by centrifugation at 800 g for 10 min. The eluted peptides were collected with LoBind eppis (Eppendorf) and dried using a concentrator plus (Eppendorf) at 40 ºC. Then the peptides were reconstituted in 120 µL FA (1%) and 2 µL of each sample was injected on a timsTOF pro mass spectrometer (Bruker) in data-independent acquisition (DIA) mode for LC-MS/MS measurements. All the MSMS samples were measured with 100 min gradient method (see Table S9). The MS samples were performed in n = 4 biological replicates.

2.8. LC-MS/MS Measurements on TimsTOF Pro

Peptides were analyzed and separated online using a Bruker timsTOF Pro mass spectrometer coupled to a Dionex UltiMate 3000 nano HPLC system with a Captive Spray nano-electrospray ion source and Sonation column oven. Peptides were initially loaded onto the trap column (Acclaim PepMap100 C18, 75 µm ID x 2 cm, 3 µm particle size, Thermo Fisher Scientific), and washed with mobile phase A (0.1% formic acid in H_2O) for 7 min at a flow rate of 5 µL/min. They were then transferred to the separation column (Aurora C18 column, 25 cm × 75 µm, 1.7 µm, IonOpticks) and separated using a gradient method according to the tables below. Mobile phase A is 0.1% (v/v) formic acid in water, while mobile phase B is 0.1% (v/v) formic acid in acetonitrile.

Table S7. 60 min gradients method for LC-MS/MS measurements

Time (min)	Flow (µL/min)	0.1% FA in H_2O (A)	0.1% FA in CH_3CN (B)
0	0.4	95%	5%
7.0	0.4	95%	5%
38.0	0.4	72%	28%
44.0	0.4	60%	40%
44.1	0.4	5%	95%
50.0	0.4	5%	95%
50.1	0.4	95%	5%
60.0	0.4	95%	5%

Table S8. 90 min gradients method for LC-MS/MS measurements

Time (min)	Flow (µL/min)	0.1% FA in H_2O (A)	0.1% FA in CH_3CN (B)
0	0.4	95%	5%
7.0	0.4	95%	5%
67.0	0.4	60%	40%
72.0	0.4	40%	60%
72.1	0.4	10%	90%
82.0	0.4	10%	90%
82.1	0.4	95%	5%
90.0	0.4	95%	5%

Table S9. 100 min gradients method for LC-MS/MS measurements

Time (min)	Flow (µL/min)	0.1% FA in H_2O (A)	0.1% FA in CH_3CN (B)
0	0.4	95%	5%
7.0	0.4	95%	5%
67.0	0.4	72%	28%
80.0	0.4	60%	40%
80.1	0.4	5%	95%
90.0	0.4	5%	95%
90.1	0.4	95%	5%
100.0	0.4	95%	5%

DIA-PASEF settings

The timsTOF Pro was operated in data-independent dia-PASEF mode with the dual TIMS analyzer operating at equal accumulation and ramp times of 100 ms each and ion mobility coefficients (1/K0) range from 0.60 – 1.60 V × s/cm² for MS1 scans. Dia-PASEF included a mass range of 400 – 1201 m/z and an ion mobility range of 0.60 – 1.43 V × s/cm² for

fragmentation of MS2 scans. Each scan comprised two ion mobility isolation windows of 25 m/z widths. With 32 isolation windows and 1 m/z overlaps, the setup covered the mass range, resulting in 16 dia-PASEF scans per MS1 scan and a total cycle time of approximately 1.80 s. The collision energy was ramped linearly as a function of mobility from 59 eV at 1/K0 = 1.3 V × s/cm^2 to 20 eV at 1/K0 = 0.85 V × s/cm^2. TIMS voltages were calibrated linearly to obtain the reduced 1/K0 using three Agilent ESI-L Tuning Mix ions (m/z 622, 922, and 1222) spiked into the Captive Spray Source inlet filter.

DDA-PASEF settings
The timsTOF Pro was operated in data-dependent PASEF mode with the dual TIMS analyzer operating at equal accumulation and ramp times of 100 ms each with a set 1/K0 ion mobility range from 0.85 – 1.40 V × s/cm^2 for MS1 scans. DDA-PASEF included a mass range of 100 – 1700 m/z with the capillary voltage of the Captive Spray source set to 1500 V for fragmentation of MS2 scans. TOP 10 dda-PASEF scans per topN acquisition cycle were performed, resulting in a total cycle time of 1.17 s. Only precursors reaching an intensity threshold of 1750 arbitrary units were considered for 675 fragmentations, and precursors reaching a target intensity of 14500 arbitrary units were dynamically excluded for 0.4 min. The quadrupole isolation width was set to 2 Th for m/z < 700 and to 3 Th for m/z > 800. The collision energy was ramped linearly as a function of mobility from 59 eV at 1/K0 = 1.6 V × s/cm^2 to 20 eV at 0.6 V × s/cm^2. TIMS voltages were calibrated linearly to obtain the reduced 1/K0 using three ESI-L Tuning Mix ions (Agilent, mz 622, 922 and 1222) spiked on the Captive Spray Source inlet filter.

2.9. Preparative Labeling and Full Proteome LC-MS/MS Data Analysis

MS data was analyzed using DIA-NN (version 1.8.1)[11, 12] in library-free mode. The in situ library generation was searched against the Uniprot reference proteome: *E. coli* K-12 (ID: UP000000625, retrieved on 2023.05.02), *E. coli* BL21 (ID: UP000002032, retrieved on 2024.01.13, with YliI or PedH FASTA combined), *P. putida* KT2440 (ID: UP000000556, retrieved on 2024.01.14) and HepG2 (ID: UP000005640, only Swiss-Prot reviewed gene retrieved on 2024.01.14, with Frankenfield's universal contaminants[13] and reverse FASTA combined).
The DIA-NN was operated with the following parameters: under "precursor ion generation," the "FASTA digest for library-free search / library generation" and "Deep-learning-based spectra, RTs and IMs prediction" were enabled, the "Protease" was set to Trypsin / P, the "Missed cleavages" was set to 2, the "Maximum number of variable modifications" was set to 0, the "N-term M excision" and "C-carbamidomethylation" were enabled, the "Peptide length range" was set to 7 – 30, the "Precursor charge range" was set to 2 – 4, the "Precursor m/z range" was set to 300 –1800, and the "Fragment ion m/z range" was set to 200 – 1800; under "Output," the "Generate spectral library," "Quantities matrices" and "Generate PDF report" were enabled, both "Precursor FDR (%)" and "Log level" were set to 1; under "Algorithm," the "Mass accuracy," "MS1 accuracy" and "Scan window" were all set to 0, the " Use isotopologues," "MBR," "Heuristic protein inference" and "No shared spectra" were enabled, the "Protein inference" mode was set to Genes, the "Neural network classifier" was operated in a single-pass mode, the "Quantification strategy" was proceeded with Robust LC (high precision), the "Cross-run normalization" was based on RT-dependent, the "Library generation" mode was set to smart profiling, the "Speed and RAM usage" was employed with optimal results. After DIA-NN analysis, LFQ for all protein groups were analyzed using Perseus (version 2.0.10).[14, 15] The following procedures were used to proceed with data: 1). Rename columns, 2). Add

annotations, 3). Log_2 Transform of LFQ intensities, 4). Categorical annotations of rows, 5). Remove contaminants, 6). Remove reverse, 7). Filter rows based on valid values (2 for three replicates, 3 for four replicates) with at least in one group, 8). Impute the missing values from normal distribution over the total matrix, 9). Two-sample Student's t-test with 5% FDR. Step 5 and step 6 were only used for HepG2 proteomics data analysis to filter out the reverse and containments. The fold change values and statistical significance were determined by Perseus scatter plots, which were replotted using GraphPad Prism 10.1.0.

2.10. MS/MS Data Analysis

The MS/MS data was analyzed with Fragpipe (version 20.0, for *E. coli* K-12 data analysis) and Maxquant (version 2.6.4, for RuvB binding site identification).
Fragpipe search was performed against the Uniprot reference proteome: *E. coli* K-12 (ID: UP000000625, retrieved on 2023.05.02, with Fragpipe default contaminants combined). For further data analysis, the FragPipe build-in MSFragger (version 3.8),[16] Philosopher (version: 5.0.0),[17] IonQuant (version 1.9.8),[18] Python (version 3.9.13) and EasyPQP (version 0.1.50) were used. The MS/MS spectra from Fragpipe were visualized by PDV 1.1.1.[19]
Open search[20] analysis with FragPipe
To identify the unannotated mass shift of **PQQ** covalent binding against *E. coli* K-12 proteome, an open search was performed. The "MS/MS type" was selected IM-MS. The negative controls were set as "Control" and the **PQQ**-treated experiments were set "Experiment." The "DIA-Umpire SE (Signal Extraction)" was disabled. The "Run MSFragger" was enabled with the following parameters: under "Peak Matching" of "Common Options," the "Precursor mass tolerance" was set to -150 to +150 Da, the "fragment mass tolerance" was set to 20 ppm, the "Calibration and Optimization" mode was employed with Mass calibration and parameter optimization, the "Isotope Error" were set to 0; under "Protein Digestion" of "Common Options," the "cleavage" was set to enzymatic, the "Clip N-term M" was enabled, the "enzyme name 1" was set to strict trypsin, the "Cuts 1" was set to after KR, the "missed cleavages 1" was set to 2, the "Sense 1" was set to C, the "peptide length" was set to 7 – 50, the "peptide mass range" was set to 500 – 5000 and the "Split database" was set to 1; under "Modification," the "Max variable mods on a peptide" was set to 3 and "Max combinations" was set to 5000 for "Variable modifications," and no "fixed modifications" were enabled; under "Advanced Options," the "Mass offsets" was set to 0 and the "Restrict delta mass" was set to all; the "Glyco/Labile Mods" was disabled; under "Spectral Processing" of "Advanced Options," the "Activation Type Filter" was set to all, the "Precursor mass mode" was set to corrected, the "Check spectra files" and the "Require precursor" were enabled, the "Min peaks" was set to 15, the "Use top N peaks" was set to 150, the "Min ratio" was set to 0.01, the "Clear m/z range" was set to 0 – 0, the "Intensity transform" was set to None, the "Remove precursor peak" mode was proceed with only peak with precursor charge and the "removal m/z range" was set to -1.5 – +1.5; under the "Open Search Options," the "Report mass shift as a variable mod" was set to No, the "Track zero top N" was set to 0, the "Add top N complementary" was set to 0, the "Zero bin accept expect" was set to 0, the "Zero bin multiply expect" was set to 1; the "Delta mass exclude rang" was set to (-1.5, 3.5), and the "Localize mass shift (LOS)" was enabled; under "Advanced Output Options," the "Report top N for DDA" was set to 1, the "Report alternative proteins" was enabled, the "Output format" mode was based on PEPXML_PIN, the "Output max expect" was set to 50, the "Report top N for DIA" was set to 5, the "Group variable" was set to None and the "Report top N for GPF-DIA" was set to 3; under "advanced Peak Matching Options," the "Min frags modeling" was set to 2, the "Min matched frags" was set to 4, the "Max fragment charge" was set to 2, the "Deisotope" was set to Yes, the "Fragment ion series" set to b, y, the

“Deneutraloss” was set to Yes and the “Precursor true tolerance” was set to 20 ppm. The “Validation Tools” option were set as the following parameters: the “Crystal-C” and the “Rescoring using deep learning prediction” were disabled; under “PSM validation,” the “Run PeptideProhet” was operated with the following command: --nonparam –exectscore –decoyprobs –masswidth 1000.0 -clevel -2 with “Single combined pepxml file per experiment / group enabled; under “PTM Site localization,” the “PTMProphet” was disabled; under “Protein inference,” the “ProteinProhet” was operated with the following command: --maxppmdiff 2000000; under “FDR filter and Report,” the “Generate reports” was operated with the following command: --sequential --prot 0.01 –mapmods and the “Generate protein-level summary” was enabled. The “Run PTM-Shepherd” option was enabled with the following parameters: under “PTM Profiling,” the “Smoothing factor” was set to 2, the “Precursor tolerance” was set to 0.01 Da, the “Prominence ratio” was set to 0.3, the “Peak picking width” was set to 0.002 Da, the “Peak minimum PSMs” was set to 10, the “Max fragment charge” was set to 1 and the “Fragment mass tolerance” was set to 20 ppm, the “Normalize data to” was normalized to PSMs; under “Annotation,” the “Annotation tolerance” was set to 0.01 Da, a custom reduced mass-list file was used as the “Custom annotation file”; under “Localization,” the ions were set to b and y with “localization background” set to 4; the “Diagnostic Feature Discovery” and the “Diagnostic Feature Extraction” were disabled. The “MS1 Quantification,” “Isobaric Labeling-Based quantification” and “Spectral library generation” options were disabled.
For downstream data analysis, the unannotated mass shift of 309.9860 was retrieved from ‘global.modsummary.tsv’ file, in which the psm was 0 for controls and the psm was positive for **PQQ** treatments. And the mass of 309.9860 was in line with the IPMS results. Thus, 309.9860 is the characteristic mass of the **PQQ** covalent binding against protein.

Offset search with Fragpipe
To identify the **PQQ** covalent binding amino acid preference against *E. coli* K-12 proteome, an offset search was performed. The “MS/MS type” was selected IM-MS. Here, **PQQ**-treated experiments only were uploaded for data analysis. The “DIA-Umpire SE (Signal Extraction)” was disabled. The “Run MS Fragger” was enabled with the following parameters: under “Peak Matching” of “Common Options,” the “Precursor mass tolerance” was set to -20 to +20 ppm, the “fragment mass tolerance” was set to 20 ppm, the “Calibration and Optimization” mode was employed with Mass calibration and parameter optimization, the “Isotope Error” were set to 0/1/2; under “Protein Digestion” of “Common Options,” the “cleavage” was set to enzymatic, the “Clip N-term M” was enabled, the “enzyme name 1” was set to trypsin, the “Cuts 1” was set to after KR but “No cuts 1” after P, the “missed cleavages 1” was set to 2, the “Sense 1” was set to C, the “peptide length” was set to 6 – 50, the “peptide mass range” was set to 500 – 5000 and the “Split database” was set to 1; under “Modification,” the “Max variable mods on a peptide” was set to 3, “Max combinations” was set to 5000 and “Use all mods in the first search” was enabled for “Variable modifications,” and no “fixed modifications” were enabled; under “Advanced Options,” the “Mass offsets” was set to 0/309.9860 and the “Restrict delta mass” was set to all, the “Glyco/Labile Mods” was disabled; under “Spectral Processing” of “Advanced Options,” the “Activation Type Filter” was set to all, the “Precursor mass mode” was set to selected, the “Check spectra files” and the “Require precursor” were enabled, the “Min peaks” was set to 15, the “Use top N peaks” was set to 150, the “Min ratio” was set to 0.01, the “Clear m/z range” was set to 0 – 0, the “Intensity transform” was set to square root, the “Remove precursor peak” mode was proceeded with peaks with all charge states and the “removal m/z range” was set to -1.5 – +1.5; under “Open Search Options,” the “Report mass shift as a variable mod” was set to Yes, remove delta mass, the “Track zero top N” was set to

0, the “Add top N complementary” was set to 0, the “Zero bin accept expect” was set to 0, the “Zero bin multiply expect” was set to 1, the “Delta mass exclude rang” was set to (-1.5, 3.5), and the “Localize mass shift (LOS)” was enabled; under “Advanced Output Options,” the “Report top N for DDA” was set to 1, the “Output format” mode was based on TSV_PEPXML_PIN, the “Output max expect” was set to 50, the “Report top N for DIA” was set to 5, the “Group variable” was set to None and the “Report top N for GPF-DIA” was set to 3; under “advanced Peak Matching Options,” the “Min frags modeling” was set to 2, the “Min matched frags” was set to 4, the “Max fragment charge” was set to 2, the “Deisotope” was set to Yes, the “Fragment ion series” set to b, y, the “Deneutraloss” was set to Yes and the “Precursor true tolerance” was set to 20 ppm. The “Validation Tools” option were set as the following parameters: the “Crystal-C” and the “Rescoring using deep learning prediction” was disabled; under “PSM validation,” the “Run PeptideProhet” was operated with the following command: --nonparam –exectscore –decoyprobs –masswidth 1000.0 -clevel -2 with “Single combined pepxml file per experiment / group” enabled; under “PTM Site localization,” the “PTMProphet” was disabled; under “protein inference,” the “ProteinProhet” was operated with the following command: --maxppmdiff 2000000; under “FDR filter and Report,” the “Generate reports” was operated with the following command: --sequential --mapmods --prot 0.01 and the “Generate protein-level summary” was enabled. The “Run PTM-Shepherd” option was enabled with the following parameters: under “PTM Profiling,” the “Smoothing factor” was set to 2, the “Precursor tolerance” was set to 20 ppm, the “Prominence ratio” was set to 0.3, the “Peak picking width” was set to 20 ppm, the “Peak minimum PSMs” was set to 10, the “Max fragment charge” was set to 2 and the “Fragment mass tolerance” was set to 20 ppm, the “Normalize data to” was normalized to PSMs; under “Annotation,” the “Annotation tolerance” was set to 0.01 Da; a custom reduced mass-list file was used as the “Custom annotation file”; under “Localization,” the ions were set to b and y with “localization background” set to 4; under “Diagnostic Feature Discovery,” the “Mine for diagnostic ions and fragments” was enable, the “Min. peptide ions per MS1 delta mass peak” of 25 and the “Min. intensity fold change” of 3 were set to all “Diagnostic ions,” “Peptide ions” and “Fragment ions,” the “Min. fragment ions per spec” of 2 and the “Min. fragment propensity” of 12.5 were set to “Fragment ions;” the “Diagnostic Feature Extraction” mode was set to Extract known diagnostic ions from spectra. The “MS1 Quantification,” “Isobaric Labeling-Based quantification” and “Spectral library generation” options were disabled.

For downstream analysis,[21] the “tsv” files for the four experiments were individually processed. They were filtered to retain only entries that are present in the “psm.tsv” file, which contains the PSMs filtered by 1% PSM- and protein-level FDR. The column “best locs” indicates the possible residues modified by the mass offset. MSFragger puts the mass offset on each residue one-by-one and calculates hyperscores. The residues with the highest hyperscore were indicated by lower-case letters. Only entries were retained that were localized to a unique residue as seen by containing one lower-case letter (If there was no lower-case letter, the score for the unmodified peptide was higher than that for the best modified peptide and therefore no localization was performed). Next, the entries were filtered for a delta score > 1 (delta score was defined as the difference of the highest hyperscore and the second highest hyperscore during the localization). For each entry, the UniProt Code was isolated from the column “Protein” and the full protein sequence was linked into the table. Based on this information, all peptide sequences that did not occur exactly once in the identified protein were excluded and the residue number of the modified residue was determined. If the N-terminus (modification at amino acid 1 or amino acid 2 and if amino acid 1 was not present in the peptide (clipping of N-terminal methionine)) or the C-terminus (last amino acid of the protein) were

modified, this was only counted and labelled as modification of the terminus and not of the respective amino acid at that position. For each entry, an identifier was generated in the format "UniProtCode"_X_"residue number," where X is the one letter code of the modified amino acid or "N-terminal" or "C-terminal" for terminal modifications. Duplicates of entries with the same identifier were retained only once. The data of four experiments was then combined and only residues were counted in the final analysis that were present in all the replicates. The fraction of all sites that were modified at each amino acid and the terminal was reported.

Closed Search Analysis with Fragpipe

To identify the binding proteins of **PQQ** against *E. coli* K-12 proteome, a closed search was performed. The "MS/MS type" was selected IM-MS. Here, the **PQQ**-treated experiments only were uploaded for data analysis. The "DIA-Umpire SE (Signal Extraction)" was disabled. The "Run MS Fragger" was enabled with the following parameters: under "Peak Matching" of "Common Options," the "Precursor mass tolerance" was set to -20 to +20 ppm, the "fragment mass tolerance" was set to 20 ppm, the "Calibration and Optimization" mode was employed with Mass calibration and parameter optimization, the "Isotope Error" were set to 0/1/2/3; under "Protein Digestion" of "Common Options," the "cleavage" was set to enzymatic, the "Clip N-term M" was enabled, the "enzyme name 1" was set to strict trypsin, the "Cuts 1" was set to after KR, the "missed cleavages 1" was set to 2, the "Sense 1" was set to C, the "peptide length" was set to 7 – 50, the "peptide mass range" was set to 500 – 5000 and the "Split database" was set to 1; under "Variable modifications" of "Modification," the "Max variable mods on a peptide" was set to 3 and "Max combinations" was set to 5000, the "Variable modifications" was set to 309.9860 from Open Search, the "Site" was set to K with "Max occurrences" of 2, no "fixed modifications" under "Modification" was enabled; under "Advanced Options," the "Mass offsets" was set to 0 and the "Restrict delta mass" was set to all; the "Glyco/Labile Mods" was disabled; under "Spectral Processing" of "Advanced Options," the "Activation Type Filter" was set to all, the "Precursor mass mode" was set to selected, the "Check spectra files" and the "Require precursor" were enabled, the "Min peaks" was set to 15, the "Use top N peaks" was set to 150, the "Min ratio" was set to 0.01, the "Clear m/z range" was set to 0 – 0, the "Intensity transform" was set to None, the "Remove precursor peak" mode was proceeded with only peak with precursor charge and the "removal m/z range" was set to -1.5 – +1.5; the "Open Search Options" was disabled; under "Advanced Output Options," the "Report top N for DDA" was set to 1, the "Report alternative proteins" was enabled, the "Output format" mode was based on PEPXML_PIN, the "Output max expect" was set to 50, the "Report top N for DIA" was set to 5, the "Group variable" was set to None and the "Report top N for GPF-DIA" was set to 3; under "advanced Peak Matching Options," the "Min frags modeling" was set to 2, the "Min matched frags" was set to 4, the "Max fragment charge" was set to 2, the "Deisotope" was set to Yes, the "Fragment ion series" set to b, y, the "Deneutraloss" was set to Yes and the "Precursor true tolerance" was set to 20 ppm. The "Validation Tools" option were set as the following parameters: the "Crystal-C" was not enabled; under "Rescoring using deep learning prediction," the "Run MSBooster," "Predict RT" and "Predict spectra" were enabled; under "PSM validation," the "Percolator" was operated with the following command: --only-psms --no-terminate --post-processing-tdc; under "PTM Site localization," the "PTMProphet" was disabled; under "Protein inference," the "ProteinProhet" was operated with the following command: --maxppmdiff 2000000; under "FDR filter and Report," the "Generate reports" was operated with the following command: --sequential --picked --prot 0.01 and the "Generate protein-level summary" was enabled. The "Run PTM-Shepherd" option was disabled. The "Run MS1 quant" and "IonQuant" for "MS1 Quantification" were enabled with the following

parameters: under “Basic options,” the “LFQ” and “Add MaxLFQ” were enabled, and the “MaxLFQ min ions” was set to 2; under “common,” the “Match between runs (MBR)” was enabled, the “MBR ion FDR” was set to 0.01, “Normalize intensity across runs” was enabled, the “Peptide-protein uniqueness” mode was set to unique+razor; under “Feature detection and peak tracing” of “Advance options,” the “Min scans” was set to 3, the “Min isotopes” was set to 2, the “m/z tolerance (ppm)” was set to 10, the “RT tolerance (minutes)” was set to 0.4, and the “IM tolerance (1/k0)” was set to 0.05; under “Match between runs (MBR)” of “Advanced Options,” the “MBR RT tolerance (minutes)” was set to 1, the “MBR IM tolerance (1/k0)” was set to 0.05, the “MBR peptide FDR” was set to 1, the “MBR min correlation” was set to 0, the “MBR top runs” was set to 10 and the “MBR protein FDR” was set to 1; under “Intensity” of “Advanced options,” the “Top N ions” was set to 0 and the “Min freq” was set to 0; under “Other,” the “Min site localization probability” was set to 0.75; the “FreeQuant (alternative tool)” was disabled. The “Isobaric labeling-based quantification” and “Spectral library generation” options were disabled.

For downstream data analysis, the “ion.tsv” files of the four replicates and the ‘’combined modified peptides’’ file were analyzed. For each entry, the “Modified peptide” was generated based on the entry with **PQQ** modified mass of 309.9860 from open search. The masses of probe modification in the “Modified Peptide” were replaced by an “*,” and the masses of carbamidomethylation ([57.0215]), methionine oxidation ([15.9949]) and N-terminal acetylation ([42.0106]) in this entry were deleted, if present. Merge the ‘’ion.tsv’’ files and ‘’combined modified peptides’’ into one file based on the **PQQ** modified peptides, charges and spectral count. the UniProt Code was isolated from the column “Protein” and the full protein sequences were linked into the table. Based on this information, all peptide sequences that did not occur exactly once in the same protein were excluded and the residue number of the modified residue was determined. The “Identifier” was generated in the format “UniProtCode”_X_”residue number,” where X is the one letter code of the modified amino acid or “N-term” or “C-term” for terminal modifications. First the ions were aggregated within the same replicate. If several different “Modified peptides” were detected for the same “Identifier” in the same replicate, the “Modified Peptide” and “Peptide Sequence” with the shortest sequence were kept. Then this “identifier” was deleted if the total number of MS/MS and MBR as the match type from all replicates were less than 2. And this “identifier” was deleted as well if the total PSMs for each “identifier” from all replicates was less than 2. In addition, the “identifier” was kept and considered to be as true positive only if the Peptide prophet Probability (median value of all replicates) > 0.75. Last, for each “Identifier,” the standard deviation of the “Log_{10} intensity” from all replicates was determined as calculated by log_{10} transformed peptide mass intensity. The value was kept if the standard deviation of Log_{10} Intensity was less than 0.5 for all ions of the same “Identifier.” If different “Modified peptides” were detected for the same “Identifier” across the different replicates, the “Modified Peptide” and “Peptide Sequence” with the shortest sequence were kept. Finally, the filtered modified peptides were linked to the related proteins. And those proteins were **PQQ** modified proteins in *E. coli* K-12 proteome.

Closed search with Maxquant

MaxQuant[22, 23] search was performed against the Uniprot reference proteome: *E. coli* K-12 (ID: UP000000625, retrieved on 2023.05.02, with Maxquant default contaminants combined). “**PQQ** Modification” of $C_{14}H_4N_2O_7$ (309.9862) was added to the configuration table The mass data was analyze based on the label-free quantification with Andromeda.[24] Under “Raw data,” the “PTM” of negative controls were set false and experiments set as true, the “Fraction” was set empty. The “group-specific parameters” was adopted as below: the “Type” mode was set

to “TIMS-DDA”; under “modification,” the “variable modifications” were set to oxidation (methionine) and acetylation (N-terminus), the “fixed modifications” was set to carbamidomethylation (cysteine) and **PQQ** modification of 309.9860; under “label-free quantification,” the mode was set to LFQ; under “Misc.,” the “Re-quantify” was enabled; under “Digestion,” the “Digestion mode” was set to specific and Trypsin/P as enzyme with “max. missed cleavage” as 2; under “Instrument,” the “Instrument type” was set to Brucker TIMS; the “cross links” and “first search” were disabled. The “Global parameters” was adopted as below: under “sequence,” the “include contaminants” was enabled; under “identification,” the “Match between runs” was enabled; under “protein quantification,” oxidation (methionine), acetylation (N-terminus) and **PQQ** modification were set for “Modifications used in protein quantification”; the “Tables,” “MS/MS analyzer,” “Advanced,” “Folder location” and “MS/MS fragment” settings were set as default.
For the downstream analysis, the “PQQ Modification Sites” text file was analyzed. For every independent experiment, the modified sites of RuvB were regarded as true positives if they exhibit a localization probability > 0.85, false localization rate (FLR) < 0.05 and Posterior Error Probability (PEP) < 0.01. And those modified sites of RuvB were kept only if they were identified by two different experiments (treated with **PQQ** only; treated with **PQQ** and $MgCl_2$).

2.11. Molecular Cloning

Genomic DNA of *E. coli* K-12 and *P. putida* KT2440 were isolated with bacterial DNA isolation kit (VWR). All primers (Table S12) were ordered from Sigma Aldrich, and all PCR reactions were performed with the respective primers as described in Table S10, S11. Amplification of the fragments and vectors were confirmed by agarose gel electrophoresis. The methylated templated DNA was removed by DpnI digestion at 37 ºC for 1 h. The PCR products were further purified with Gel Extraction Kit (Omega) after agarose gel electrophoresis.

The constructed plasmids were achieved by using Gateway cloning[25] and Gibson Assembly[26] (protocols see below), which were then transformed into chemical competent *E. coli* TOP10 or NEB 10β or *E. coli* BL21 (DE3) cells by heat shock at 42 ºC for 1 min. Then the cells were added 500 µL SOC medium (yeast extract 1 g, tryptone 4 g, NaCl 120 mg, KCl, 40 mg, $MgSO_4$ 400 mg, pH 6.8 – 7, 20 mM $MgCl_2 \cdot 6H_2O$ and 1 M $MgSO_4 \cdot 7H_2O$ and 20 mM glucose) and incubated at 37 ºC at 200 rpm for 2 h. Afterwards, transformed cells were cultivated overnight at 37 ºC with LB agar plates containing antibiotics (gentamicin or ampicillin or kanamycin). The single colonies were picked and incubated in LB medium containing antibiotic at 37 ºC, 200 rpm overnight. The constructs were isolated with peqGOLD Plasmid MiniPrep Kit I (VWR) and confirmed with sanger sequencing (Genewiz Germany GmbH). The PedH-C-His (PET29b_PedH_F412V_W561A) and N-His-YliI (PET16b_YliI) constructs were purchased from TWIST Bioscience, which were subsequently transformed into *E. coli* BL21 (DE3) gold for protein expression.

Gateway cloning
Gateway cloning was performed with pDonor207 as donor vector and pET300 / pET301 as destination vectors according to the protocols of Invitrogen. Final constructs were transformed into *E. coli* TOP10 or NEB 10β or *E. coli* BL21 (DE3) and confirmed with sanger sequencing (Genewiz Germany GmbH). In this research, the technique was applied onto Tig, HtpG, Gsk and FklB plasmid constructions.

Gibson Assembly

Gibson Assembly was performed with pET300 / pET29b as subclone vectors. All vectors were assembled using an isothermal Gibson Assembly (1 h, 50 ºC) for PCR fragments. Final constructs were transformed into NEB 10β or *E. coli* BL21 (DE3), except for native YliI (pET29b_YliI) and native PedH (pET29b_PedH), which were transformed into *E. coli* BL21_Tuner for proteomics. Then they were confirmed with sanger sequencing (Genewiz Germany GmbH). In this research, the technique was applied to MBP, YliI, SurA, PedH, PpiD, RuvB, $RuvB^{K26H}$ and $RuvB^{K46H}$ plasmid constructions.

Table S10. Conditions used for the PCR reaction

Component	Amount
Autoclaved ddH_2O	31 µL
5 x Phusion GC buffer	10 µL
10 mM dNTPs	1 µL
10 µM forward primer	2.5 µL
10 µM reverse primer	2.5 µL
Template DNA (30 – 100 ng/ µL)	1 µL
DMSO	1.5 µL
Phusion DNA polymerase	0.5 µL
Total	50 µL

Table S11. General program used for the thermocycler of the PCR conditions

Step	Temp.	Duration	Cycle
Initial denaturation	98 ºC	30 s	
Denaturation	98 ºC	7 s	
Annealing	Primer dep.	7 s	40X
Extension	72 ºC	20 – 30 s/Kbp	
Final extension	72 ºC	10 min	
Hold	4 ºC	∞	

Table S12. Primers used for cloning

Primer	Organism/ plasmid	Sequence	Comment / Construct
Tig_for	*E. coli* K-12	ggggacaagtttgtacaaaaaagcaggctttCAAGTTTCAGTTGAAACCACTCAAG	PET300_Tig
Tig_rev	*E. coli* K-12	ggggaccactttgtacaagaaagctgggtgTTACGCCTGCTGGTTCATC	PET300_Tig
HtpG_for	*E. coli* K-12	ggggacaagtttgtacaaaaaagcaggctttAAAGGACAAGAAACTCGTGG	PET300_HtpG
HtpG_rev	*E. coli* K-12	ggggaccactttgtacaagaaagctgggtgTCAGGAAACCAGCAGC	PET300_HtpG
Gsk_for	*E. coli* K-12	ggggacaagtttgtacaaaaaagcaggctttAAATTTCCCGGTAAACGTAAATCC	PET300_Gsk

Gsk_rev	*E. coli* K-12	ggggaccactttgtacaagaaagctgggtgTTAACGATCCCAGTAAGACTCTTC	PET300_Gsk
FklB_for	*E. coli* K-12	ggggacaagtttgtacaaaaaagcaggctttgaaggagatagaaccATGACCACCCCAACTTTTGAC	PET301_FklB
FklB_rev	*E. coli* K-12	ggggaccactttgtacaagaaagctgggtgGAGGATTTCCAGCAGTTCGAC	PET301_FklB
MBP_frag_for	*E. coli* K-12	TGTACAAAAAAGCAGGCTTTAAAATCGAAGAAGGTAAACTGG	PET300_MBP
MBP_frag_rev	*E. coli* K-12	TTGTACAAGAAAGCTGGGTGTTACTTGGTGATACGAGTCTG	PET300_MBP
PET300_for	PET300_Tig	CACCCAGCTTTCTTGTACAAAG	PET300 backbond for surface display construct
PET300_rev	PET300_Tig	AAAGCCTGCTTTTTTGTACAAACTTG	PET300 backbond for surface display construct
YliI_frag_for	*E. coli* K-12	TTTAAGAAGGAGATATACATATGCATCGACAATCCTTTTTC	PET29b_YliI
YliI_frag_rev	*E. coli* K-12	CAATGGTGGTGATGATGATGCTAATTGCGTGGGCTAAC	PET29b_YliI
PET29b_for	PET29b_PedH_F412V_W561A	CATCATCATCACCACCATTGACT	PET29b backbond for surface display construct
PET29b_rev	PET29b_PedH_F412V_W561A	ATGTATATCTCCTTCTTAAAGTTAAACAAAATTATTTCTAG	PET29b backbond for surface display construct
SurA_frag_for	*P. putida* KT2440	TGTACAAAAAAGCAGGCTTTCTGGGCGTCGCTTTGC	PET300_SurA
SurA_frag_rev	*P. putida* KT2440	TTGTACAAGAAAGCTGGGTGTCACTGGGCGGCCTG	PET300_SurA
PpiD_frag_for	*P. putida* KT2440	TGTACAAAAAAGCAGGCTTTGCCGCCACTCATAGC	PET300_PpiD
PpiD_frag_rev	*P. putida* KT2440	TTGTACAAGAAAGCTGGGTGTTAGTAACGGGTGATGTCTGC	PET300_PpiD
PedH_frag_for	*P. putida* KT2440	TTTAAGAAGGAGATATACATATGACCCGATCCCCAC	PET29b_PedH

PedH_frag_rev	*P. putida* KT2440	CAATGGTGGTGATGATGATG TTATGGCTTGACGCTTGC	PET29b_PedH
RuvB_frag_for	*E. coli* K-12	TGTACAAAAAAGCAGGCTTTA TTGAAGCAGACCGTC	PET300_ RuvB
RuvB_frag_rev	*E. coli* K-12	TTGTACAAGAAAGCTGGGTG TTACGGCATTTCTGGC	PET300_ RuvB
$RuvB^{K26H}$_frag_for	PET300_RuvB	TGTACAAAAAAGCAGGCTTTA TTGAAGCAGACCGTCTGATTT CTGCCGGTACCACTTTGCCG GAAGATGTAGCAGATCGCGC CATTCGCCCCCATTTACTGGA AGAG	PET300_ $RuvB^{K26H}$
$RuvB^{K26H}$_frag_rev	PET300_RuvB	TTGTACAAGAAAGCTGGGTG TTACGGCATTTCTGGC	PET300_ $RuvB^{K26H}$
$RuvB^{K46H}$_frag_for1	PET300_RuvB	TGTACAAAAAAGCAGGCTTTA TTGAAGCAGACCGTC	PET300_ $RuvB^{K46H}$
$RuvB^{K46H}$_frag_for2	PET300_RuvB	GGAGATTTTCATCCATGCAG CGAAACTGCGCGG	PET300_ $RuvB^{K46H}$
$RuvB^{K46H}$_frag_rev1	PET300_RuvB	TTGTACAAGAAAGCTGGGTG TTACGGCATTTCTGGC	PET300_ $RuvB^{K46H}$
$RuvB^{K46H}$_frag_rev2	PET300_RuvB	CCGCGCAGTTTCGCTGCATG GATGAAAATCTCC	PET300_ $RuvB^{K46H}$

2.12. DNA and Protein Sequences of All Constructs

Molecular weight (MW) and extinction coefficients (ε280) were calculated using the ProtParam tool on the Expasy Sever.

Trigger factor (Tig)
PET300_Tig (Tig_frag_for + Tig_frag_rev + pDonor207 + PET300)
MW: 50125 Da, ε: 18910 M^{-1} cm^{-1}
Gene sequence
ATGCATCATCATCATCATCACATCACAAGTTTGTACAAAAAAGCAGGCTTTCAAGTTTCA
GTTGAAACCACTCAAGGCCTTGGCCGCCGTGTAACGATTACTATCGCTGCTGACAGCAT
CGAGACCGCTGTTAAAAGCGAGCTGGTCAACGTTGCGAAAAAAGTACGTATTGACGGC
TTCCGCAAAGGCAAAGTGCCAATGAATATCGTTGCTCAGCGTTATGGCGCGTCTGTACG
CCAGGACGTTCTGGGTGACCTGATGAGCCGTAACTTCATTGACGCCATCATTAAAGAAA
AAATCAATCCGGCTGGCGCACCGACTTATGTTCCGGGCGAATACAAGCTGGGTGAAGA
CTTCACTTACTCTGTAGAGTTTGAAGTTTATCCGGAAGTTGAACTGCAGGGTCTGGAAG
CGATCGAAGTTGAAAAACCGATCGTTGAAGTGACCGACGCTGACGTTGACGGCATGCT
GGATACTCTGCGTAAACAGCAGGCGACCTGGAAAGAAAAAGACGGCGCTGTTGAAGCA
GAAGACCGCGTAACCATCGACTTCACCGGTTCTGTAGACGGCGAAGAGTTCGAAGGCG
GTAAAGCGTCTGATTTCGTACTGGCGATGGGCCAGGGTCGTATGATCCCGGGCTTTGA
AGACGGTATCAAAGGCCACAAAGCTGGCGAAGAGTTCACCATCGACGTGACCTTCCCG
GAAGAATACCACGCAGAAAACCTGAAAGGTAAAGCAGCGAAATTCGCTATCAACCTGAA
GAAAGTTGAAGAGCGTGAACTGCCGGAACTGACTGCAGAATTCATCAAACGTTTCGGC
GTTGAAGATGGTTCCGTAGAAGGTCTGCGCGCTGAAGTGCGTAAAAACATGGAGCGCG
AGCTGAAGAGCGCCATCCGTAACCGCGTTAAGTCTCAGGCGATCGAAGGTCTGGTAAA

AGCTAACGACATCGACGTACCGGCTGCGCTGATCGACAGCGAAATCGACGTTCTGCGT
CGCCAGGCTGCACAGCGTTTCGGTGGCAACGAAAAACAAGCTCTGGAACTGCCGCGC
GAACTGTTCGAAGAACAGGCTAAACGCCGCGTAGTTGTTGGCCTGCTGCTGGGCGAAG
TTATCCGCACCAACGAGCTGAAAGCTGACGAAGAGCGCGTGAAAGGCCTGATCGAAGA
GATGGCTTCTGCGTACGAAGATCCGAAAGAAGTTATCGAGTTCTACAGCAAAAACAAAG
AACTGATGGACAACATGCGCAATGTTGCTCTGGAAGAACAGGCTGTTGAAGCTGTACTG
GCGAAAGCGAAAGTGACTGAAAAAGAAACCACTTTCAACGAGCTGATGAACCAGCAGG
CGTAA

Protein sequence
MHHHHHHITSLYKKAGFQVSVETTQGLGRRVTITIAADSIETAVKSELVNVAKKVRIDGFRKG
KVPMNIVAQRYGASVRQDVLGDLMSRNFIDAIIKEKINPAGAPTYVPGEYKLGEDFTYSVEFE
VYPEVELQGLEAIEVEKPIVEVTDADVDGMLDTLRKQQATWKEKDGAVEAEDRVTIDFTGSV
DGEEFEGGKASDFVLAMGQGRMIPGFEDGIKGHKAGEEFTIDVTFPEEYHAENLKGKAAKF
AINLKKVEERELPELTAEFIKRFGVEDGSVEGLRAEVRKNMERELKSAIRNRVKSQAIEGLVK
ANDIDVPAALIDSEIDVLRRQAAQRFGGNEKQALELPRELFEEQAKRRVVVGLLLGEVIRTNE
LKADEERVKGLIEEMASAYEDPKEVIEFYSKNKELMDNMRNVALEEQAVEAVLAKAKVTEKE
TTFNELMNQQA

Chaperone protein HtpG (HtpG)
PET300_HtpG (HtpG_frag_for + HtpG_frag_rev + pDonor207 + PET300)
MW: 73355 Da, ε: 83310 M^{-1} cm^{-1}
Gene sequence
ATGCATCATCATCATCATCACATCACAAGTTTGTACAAAAAAGCAGGCTTTAAAGGACAA
GAAACTCGTGGTTTTCAGTCAGAAGTGAAACAGCTTCTGCACCTGATGATCCATTCTCTC
TATTCCAATAAAGAAATCTTCCTGCGTGAGCTTATCTCTAACGCCTCCGATGCGGCGGA
CAAGCTGCGTTTCCGTGCGCTCTCTAACCCGGACCTGTACGAAGGTGATGGCGAACTA
CGCGTTCGTGTCTCTTTCGATAAAGACAAGCGTACGCTGACCATCTCCGATAACGGCGT
GGGGATGACCCGCGACGAAGTGATTGACCATCTGGGGACTATCGCTAAATCCGGTACC
AAATCATTCCTCGAATCCCTGGGTTCTGACCAGGCGAAAGACAGCCAGCTGATCGGTCA
GTTTGGTGTTGGTTTCTACTCTGCGTTTATCGTGGCCGACAAAGTGACCGTGCGTACTC
GCGCGGCAGGCGAAAAACCAGAAAATGGCGTCTTCTGGGAATCGGCTGGCGAAGGTG
AATACACCGTTGCCGACATCACCAAAGAAGATCGTGGTACTGAAATCACCCTGCATCTG
CGTGAAGGCGAAGACGAGTTCCTCGATGACTGGCGCGTGCGTTCCATCATCAGCAAAT
ACTCCGACCATATCGCGCTGCCGGTAGAGATCGAAAAACGCGAAGAGAAAGACGGCGA
AACCGTTATCTCCTGGGAGAAAATCAACAAAGCGCAGGCGCTGTGGACTCGTAACAAGT
CGGAAATCACCGATGAAGAGTACAAAGAGTTCTACAAACACATCGCCCACGACTTTAAT
GATCCGCTGACCTGGAGCCACAACCGTGTTGAAGGTAAGCAGGAGTACACCAGCCTGC
TGTACATCCCGTCCCAGGCTCCGTGGGATATGTGGAACCGCGATCATAAACACGGCCT
GAAACTGTATGTTCAGCGTGTGTTCATCATGGACGACGCAGAACAGTTCATGCCGAACT
ATCTGCGCTTCGTGCGTGGTCTGATTGACTCCAGCGATCTGCCGCTGAACGTTTCCCGT
GAAATCCTCCAGGACAGCACGGTAACGCGTAACCTGCGCAATGCGCTGACCAAGCGTG
TGCTGCAAATGCTGGAAAAACTGGCGAAAGACGACGCGGAAAAATACCAGACCTTCTG
GCAACAGTTTGGCCTGGTACTGAAAGAAGGTCCGGCGGAAGATTTCGCTAACCAGGAA
GCGATCGCCAAACTGCTGCGTTTTGCTTCTACCCATACCGATTCTTCTGCGCAGACCGT
ATCTCTGGAAGACTACGTTTCCCGCATGAAAGAAGGGCAGGAGAAAATCTACTACATCA
CCGCAGACAGCTATGCGGCAGCGAAGAGCAGCCCGCACCTGGAACTGCTGCGTAAGA
AAGGCATCGAAGTTCTGCTGCTTTCCGACCGCATCGATGAGTGGATGATGAACTATCTG

ACTGAGTTCGACGGTAAACCGTTCCAGTCGGTGTCTAAAGTTGACGAGTCGCTTGAAAA
ACTGGCTGACGAAGTTGATGAGAGCGCGAAAGAAGCGGAGAAAGCACTGACTCCGTTC
ATCGACCGTGTGAAAGCCCTGCTCGGCGAGCGCGTGAAAGATGTCCGTCTGACTCACC
GTCTGACCGATACGCCAGCGATCGTTTCGACCGACGCGGACGAAATGAGCACTCAGAT
GGCGAAACTGTTCGCTGCGGCGGGCCAGAAAGTGCCAGAAGTGAAATACATCTTCGAA
CTGAACCCGGATCACGTACTGGTGAAACGTGCGGCAGATACTGAAGATGAAGCGAAGT
TCAGCGAGTGGGTAGAACTGCTGCTGGATCAGGCGCTGCTGGCAGAACGCGGCACGC
TGGAAGATCCGAACCTGTTTATTCGTCGTATGAACCAGCTGCTGGTTTCCTGA

Protein sequence
MHHHHHHITSLYKKAGFKGQETRGFQSEVKQLLHLMIHSLYSNKEIFLRELISNASDAADKLR
FRALSNPDLYEGDGELRVRVSFDKDKRTLTISDNGVGMTRDEVIDHLGTIAKSGTKSFLESL
GSDQAKDSQLIGQFGVGFYSAFIVADKVTVRTRAAGEKPENGVFWESAGEGEYTVADITKE
DRGTEITLHLREGEDEFLDDWRVRSIISKYSDHIALPVEIEKREEKDGETVISWEKINKAQAL
WTRNKSEITDEEYKEFYKHIAHDFNDPLTWSHNRVEGKQEYTSLLYIPSQAPWDMWNRDH
KHGLKLYVQRVFIMDDAEQFMPNYLRFVRGLIDSSDLPLNVSREILQDSTVTRNLRNALTKR
VLQMLEKLAKDDAEKYQTFWQQFGLVLKEGPAEDFANQEAIAKLLRFASTHTDSSAQTVSL
EDYVSRMKEGQEKIYYITADSYAAAKSSPHLELLRKKGIEVLLLSDRIDEWMMNYLTEFDGK
PFQSVSKVDESLEKLADEVDESAKEAEKALTPFIDRVKALLGERVKDVRLTHRLTDTPAIVST
DADEMSTQMAKLFAAAGQKVPEVKYIFELNPDHVLVKRAADTEDEAKFSEWVELLLDQALL
AERGTLEDPNLFIRRMNQLLVS

Guanosine-inosine kinase (Gsk)
PET300_Gsk (Gsk_frag_for + Gsk_frag_rev + pDonor207 + PET300)
MW: 50381 Da, ε: 64665 M^{-1} cm^{-1} (assuming all pairs of Cys residues form cystines)
Gene sequence
ATGCATCATCATCATCATCACATCACAAGTTTGTACAAAAAAGCAGGCTTTAAATTTCCC
GGTAAACGTAAATCCAAACATTACTTCCCCGTAAACGCACGCGATCCGCTGCTTCAGCA
ATTCCAGCCAGAAAACGAAACCAGCGCTGCCTGGGTAGTGGGTATCGATCAAACGCTG
GTCGATATTGAAGCGAAAGTGGATGATGAATTTATTGAGCGTTATGGATTAAGCGCCGG
GCATTCACTGGTGATTGAGGATGATGTAGCCGAAGCGCTTTATCAGGAACTAAAACAGA
AAAACCTGATTACCCATCAGTTTGCGGGTGGCACCATTGGTAACACCATGCACAACTAC
TCGGTGCTCGCGGACGACCGTTCGGTGCTGCTGGGCGTCATGTGCAGCAATATTGAAA
TTGGCAGTTATGCCTATCGTTACCTGTGTAACACTTCCAGCCGTACCGATCTTAACTATC
TACAAGGCGTGGATGGCCCGATTGGTCGTTGCTTTACGCTGATTGGCGAGTCCGGGGA
ACGTACCTTTGCTATCAGTCCAGGCCACATGAACCAGCTGCGGGCTGAAAGCATTCCG
GAAGATGTGATTGCCGGAGCCTCGGCACTGGTTCTCACCTCATATCTGGTGCGTTGCAA
GCCGGGTGAACCCATGCCGGAAGCAACCATGAAAGCCATTGAGTACGCGAAGAAATAT
AACGTACCGGTGGTGCTGACGCTGGGCACCAAGTTTGTCATTGCCGAGAATCCGCAGT
GGTGGCAGCAATTCCTCAAAGATCACGTCTCTATCCTTGCGATGAACGAAGATGAAGCC
GAAGCGTTGACCGGAGAAAGCGATCCGTTGTTGGCATCTGACAAGGCGCTGGACTGGG
TAGATCTGGTGCTGTGCACCGCCGGGCCAATCGGCTTGTATATGGCGGGCTTTACCGA
AGACGAAGCGAAACGTAAAACCCAGCATCCGCTGCTGCCGGGCGCTATAGCGGAATTC
AACCAGTATGAGTTTAGCCGCGCCATGCGCCACAAGGATTGCCAGAATCCGCTGCGTG
TATATTCGCACATTGCGCCGTACATGGGCGGGCCGGAAAAAATCATGAACACTAATGGA
GCGGGGGATGGCGCATTGGCAGCGTTGCTGCATGACATTACCGCCAACAGCTACCATC
GTAGCAACGTACCAAACTCCAGCAAACATAAATTCACCTGGTTAACTTATTCATCGTTAG
CGCAGGTGTGTAAATATGCTAACCGTGTGAGCTATCAGGTACTGAACCAGCATTCACCT

CGTTTAACGCGCGGCTTGCCGGAGCGTGAAGACAGCCTGGAAGAGTCTTACTGGGATC
GTTAA

Protein sequence
MHHHHHHITSLYKKAGFKFPGKRKSKHYFPVNARDPLLQQFQPENETSAAWVVGIDQTLVD
IEAKVDDEFIERYGLSAGHSLVIEDDVAEALYQELKQKNLITHQFAGGTIGNTMHNYSVLADD
RSVLLGVMCSNIEIGSYAYRYLCNTSSRTDLNYLQGVDGPIGRCFTLIGESGERTFAISPGHM
NQLRAESIPEDVIAGASALVLTSYLVRCKPGEPMPEATMKAIEYAKKYNVPVVLTLGTKFVIA
ENPQWWQQFLKDHVSILAMNEDEAEALTGESDPLLASDKALDWVDLVLCTAGPIGLYMAGF
TEDEAKRKTQHPLLPGAIAEFNQYEFSRAMRHKDCQNPLRVYSHIAPYMGGPEKIMNTNGA
GDGALAALLHDITANSYHRSNVPNSSKHKFTWLTYSSLAQVCKYANRVSYQVLNQHSPRLT
RGLPEREDSLEESYWDR

FKBP-type 22 kDa peptidyl-prolyl cis-trans isomerase (FklB)
PET301_FklB (FklB_frag_for + FklB_frag_rev + pDonor207 + PET301)
MW: 24339 Da, ε: 18450 M^{-1} cm^{-1}
Gene sequence
ATGACCACCCCAACTTTTGACACCATCGAAGCGCAAGCAAGCTACGGCATTGGTTTGCA
GGTAGGGCAACAACTGAGTGAATCTGGCCTGGAAGGGCTGCTGCCAGAAGCACTGGTT
GCAGGTATTGCCGATGCGCTGGAAGGCAAACATCCGGCTGTTCCGGTTGATGTGGTGC
ATCGCGCGCTGCGTGAAATCCACGAGCGCGCCGATGCCGTTCGTCGTCAGCGTTTCCA
GGCGATGGCTGCTGAAGGTGTGAAATACCTGGAAGAAAACGCCAAAAAAGAAGGTGTG
AATAGCACCGAATCTGGCCTGCAATTCCGCGTGATCAACCAGGGTGAAGGCGCAATTC
CGGCACGTACCGACCGCGTTCGTGTTCATTACACCGGTAAACTGATCGACGGCACCGT
GTTTGACAGCTCCGTTGCTCGTGGTGAACCCGCTGAATTCCCGGTTAATGGCGTGATCC
CTGGCTGGATTGAAGCACTGACTCTGATGCCGGTAGGTTCTAAATGGGAACTGACTATC
CCGCAGGAACTGGCATATGGCGAGCGCGGCGCAGGCGCATCCATCCCTCCGTTCAGC
ACCCTGGTGTTTGAAGTCGAACTGCTGGAAATCCTCCACCCAGCTTTCTTGTACAAAGT
GGTGATTATGCATCATCATCATCATCAC

Protein sequence
MTTPTFDTIEAQASYGIGLQVGQQLSESGLEGLLPEALVAGIADALEGKHPAVPVDVVHRAL
REIHERADAVRRQRFQAMAAEGVKYLEENAKKEGVNSTESGLQFRVINQGEGAIPARTDRV
RVHYTGKLIDGTVFDSSVARGEPAEFPVNGVIPGWIEALTLMPVGSKWELTIPQELAYGERG
AGASIPPFSTLVFEVELLEILHPAFLYKVVIMHHHHHH

Maltose/maltodextrin-binding periplasmic protein (MBP)
PET300_MBP (MBP_frag_for + MBP_frag_rev + PET300_for + PET300_rev)
MW: 42771 Da, ε: 67840 M^{-1} cm^{-1}
Gene sequence
ATGCATCATCATCATCATCACATCACAAGTTTGTACAAAAAAGCAGGCTTTAAAATCGAA
GAAGGTAAACTGGTAATCTGGATTAACGGCGATAAAGGCTATAACGGTCTCGCTGAAGT
CGGTAAGAAATTCGAGAAAGATACCGGAATTAAAGTCACCGTTGAGCATCCGGATAAAC
TGGAAGAGAAATTCCCACAGGTTGCGGCAACTGGCGATGGCCCTGACATTATCTTCTG
GGCACACGACCGCTTTGGTGGCTACGCTCAATCTGGCCTGTTGGCTGAAATCACCCCG
GACAAAGCGTTCCAGGACAAGCTGTATCCGTTTACCTGGGATGCCGTACGTTACAACG
GCAAGCTGATTGCTTACCCGATCGCTGTTGAAGCGTTATCGCTGATTTATAACAAAGATC
TGCTGCCGAACCCGCCAAAAACCTGGGAAGAGATCCCGGCGCTGGATAAAGAACTGAA

AGCGAAAGGTAAGAGCGCGCTGATGTTCAACCTGCAAGAACCGTACTTCACCTGGCCG
CTGATTGCTGCTGACGGGGGTTATGCGTTCAAGTATGAAAACGGCAAGTACGACATTAA
AGACGTGGGCGTGGATAACGCTGGCGCGAAAGCGGGTCTGACCTTCCTGGTTGACCT
GATTAAAAACAAACACATGAATGCAGACACCGATTACTCCATCGCAGAAGCTGCCTTTAA
TAAAGGCGAAACAGCGATGACCATCAACGGCCCGTGGGCATGGTCCAACATCGACACC
AGCAAAGTGAATTATGGTGTAACGGTACTGCCGACCTTCAAGGGTCAACCATCCAAACC
GTTCGTTGGCGTGCTGAGCGCAGGTATTAACGCCGCCAGTCCGAACAAAGAGCTGGCG
AAAGAGTTCCTCGAAAACTATCTGCTGACTGATGAAGGTCTGGAAGCGGTTAATAAAGA
CAAACCGCTGGGTGCCGTAGCGCTGAAGTCTTACGAGGAAGAGTTGGCGAAAGATCCA
CGTATTGCCGCCACCATGGAAAACGCCCAGAAAGGTGAAATCATGCCGAACATCCCGC
AGATGTCCGCTTTCTGGTATGCCGTGCGTACTGCGGTGATCAACGCCGCCAGCGGTCG
TCAGACTGTCGATGAAGCCCTGAAAGACGCGCAGACTCGTATCACCAAGTAA

Protein sequence
MHHHHHHITSLYKKAGFKIEEGKLVIWINGDKGYNGLAEVGKKFEKDTGIKVTVEHPDKLEE
KFPQVAATGDGPDIIFWAHDRFGGYAQSGLLAEITPDKAFQDKLYPFTWDAVRYNGKLIAYP
IAVEALSLIYNKDLLPNPPKTWEEIPALDKELKAKGKSALMFNLQEPYFTWPLIAADGGYAFK
YENGKYDIKDVGVDNAGAKAGLTFLVDLIKNKHMNADTDYSIAEAAFNKGETAMTINGPWA
WSNIDTSKVNYGVTVLPTFKGQPSKPFVGVLSAGINAASPNKELAKEFLENYLLTDEGLEAV
NKDKPLGAVALKSYEEELAKDPRIAATMENAQKGEIMPNIPQMSAFWYAVRTAVINAASGR
QTVDEALKDAQTRITK

Aldose sugar dehydrogenase (YliI) without leader peptide sequence

PET16b_YliI from TWIST Bioscience

This N-His_YliI variant, referred to "YliI," was used for spiking of recombinantly expressed protein into *E. coli* K-12 lysate as well as in vitro binding and activity studies.

MW: 39764 Da, ε: 75400 M^{-1} cm^{-1}

Gene sequence
ATGGGCCACCATCATCATCACCATGCTCCTGCAACGGTAAATGTCGAAGTACTGCAAGA
CAAACTCGACCATCCCTGGGCACTGGCCTTTTTACCCGATAATCACGGTATGTTAATCA
CTCTGCGCGGCGGCGAGTTGCGTCACTGGCAAGCAGGAAAAGGATTATCTGCGCCGCT
TTCCGGAGTTCCGGACGTTTGGGCGCACGGGCAGGGCGGCCTGCTGGACGTGGTTTT
AGCGCCTGATTTTGCTCAGTCTCGCCGCATCTGGTTAAGTTATTCCGAAGTTGGCGATG
ATGGCAAAGCCGGAACTGCTGTGGGTTATGGCCGCTTAAGTGATGATCTCTCAAAAGTG
ACCGACTTCCGCACCGTCTTTCGCCAGATGCCAAAACTGTCTACCGGCAACCATTTTGG
CGGGCGGCTGGTATTCGACGGTAAAGGTTATCTTTTTATTGCTCTGGGCGAAAACAATC
AGCGCCCGACGGCGCAGGATCTGGATAAATTACAGGGCAAACTGGTGCGTCTGACCGA
CCAGGGCGAAATCCCGGATGATAATCCTTTTATAAAGGAATCCGGTGCGCGCGCCGAG
ATCTGGTCTTATGGCATTCGTAATCCGCAAGGAATGGCGATGAATCCGTGGAGTAATGC
ACTGTGGCTGAATGAACATGGCCCGCGCGGTGGTGATGAAATTAATATCCCGCAAAAA
GGCAAAAACTACGGCTGGCCGCTGGCAACCTGGGGAATCAACTATTCAGGCTTTAAGA
TACCGGAAGCGAAAGGGGAGATCGTCGCCGGGACCGAGCAACCTGTTTTTTACTGGAA
AGATTCGCCCGCTGTGAGCGGCATGGCCTTCTATAACAGCGATAAATTCCCCCAGTGG
CAGCAAAAATTATTTATTGGCGCGCTGAAAGATAAAGATGTCATTGTGATGAGCGTCAAC
GGCGACAAAGTGACAGAAGATGGCCGTATTTTAACGGACAGAGGGCAGCGAATTCGTG
ATGTTCGCACTGGACCCGACGGTTATTTATACGTTCTCACCGACGAGTCCAGTGGGGAA
TTACTTAAAGTTAGCCCACGCAATTAG

Protein sequence
MGHHHHHHAPATVNVEVLQDKLDHPWALAFLPDNHGMLITLRGGELRHWQAGKGLSAPLS
GVPDVWAHGQGGLLDVVLAPDFAQSRRIWLSYSEVGDDGKAGTAVGYGRLSDDLSKVTDF
RTVFRQMPKLSTGNHFGGRLVFDGKGYLFIALGENNQRPTAQDLDKLQGKLVRLTDQGEIP
DDNPFIKESGARAEIWSYGIRNPQGMAMNPWSNALWLNEHGPRGGDEINIPQKGKNYGWP
LATWGINYSGFKIPEAKGEIVAGTEQPVFYWKDSPAVSGMAFYNSDKFPQWQQKLFIGALK
DKDVIVMSVNGDKVTEDGRILTDRGQRIRDVRTGPDGYLYVLTDESSGELLKVSPRN

Aldose sugar dehydrogenase (YliI) with leader peptide sequence
PET29b_YliI (YliI_frag_for + YliI_frag_rev + PET29b_for + PET29b_rev)
Native YliI was used for all proteomics experiments.
MW: 41054 Da, ε: 80900 M^{-1} cm^{-1}
Gene sequence
ATGCATCGACAATCCTTTTTCCTTGTGCCCCTTATTTGTCTTTCTTCCGCTCTCTGGGCG
GCTCCTGCAACGGTAAATGTCGAAGTACTGCAAGACAAACTCGACCATCCCTGGGCACT
GGCCTTTTTACCCGATAATCACGGTATGTTAATCACTCTGCGCGGCGGCGAGTTGCGTC
ACTGGCAAGCAGGAAAAGGATTATCTGCGCCGCTTTCCGGAGTTCCGGACGTTTGGGC
GCACGGGCAGGGCGGCCTGCTGGACGTGGTTTTAGCGCCTGATTTTGCTCAGTCTCGC
CGCATCTGGTTAAGTTATTCCGAAGTTGGCGATGATGGCAAAGCCGGAACTGCTGTGG
GTTATGGCCGCTTAAGTGATGATCTCTCAAAAGTGACCGACTTCCGCACCGTCTTTCGC
CAGATGCCAAAACTGTCTACCGGCAACCATTTTGGCGGGCGGCTGGTATTCGACGGTA
AAGGTTATCTTTTTATTGCTCTGGGCGAAAACAATCAGCGCCCGACGGCGCAGGATCTG
GATAAATTACAGGGCAAACTGGTGCGTCTGACCGACCAGGGCGAAATCCCGGATGATA
ATCCTTTTATAAAGGAATCCGGTGCGCGCGCCGAGATCTGGTCTTATGGCATTCGTAAT
CCGCAAGGAATGGCGATGAATCCGTGGAGTAATGCACTGTGGCTGAATGAACATGGCC
CGCGCGGTGGTGATGAAATTAATATCCCGCAAAAAGGCAAAAACTACGGCTGGCCGCT
GGCAACCTGGGGAATCAACTATTCAGGCTTTAAGATACCGGAAGCGAAAGGGGAGATC
GTCGCCGGGACCGAGCAACCTGTTTTTTACTGGAAAGATTCGCCCGCTGTGAGCGGCA
TGGCCTTCTATAACAGCGATAAATTCCCCCAGTGGCAGCAAAAATTATTTATTGGCGCG
CTGAAAGATAAAGATGTCATTGTGATGAGCGTCAACGGCGACAAAGTGACAGAAGATGG
CCGTATTTTAACGGACAGAGGGCAGCGAATTCGTGATGTTCGCACTGGACCCGACGGT
TATTTATACGTTCTCACCGACGAGTCCAGTGGGGAATTACTTAAAGTTAGCCCACGCAAT
TAG

Protein sequence
MHRQSFFLVPLICLSSALWAAPATVNVEVLQDKLDHPWALAFLPDNHGMLITLRGGELRHW
QAGKGLSAPLSGVPDVWAHGQGGLLDVVLAPDFAQSRRIWLSYSEVGDDGKAGTAVGYG
RLSDDLSKVTDFRTVFRQMPKLSTGNHFGGRLVFDGKGYLFIALGENNQRPTAQDLDKLQG
KLVRLTDQGEIPDDNPFIKESGARAEIWSYGIRNPQGMAMNPWSNALWLNEHGPRGGDEIN
IPQKGKNYGWPLATWGINYSGFKIPEAKGEIVAGTEQPVFYWKDSPAVSGMAFYNSDKFPQ
WQQKLFIGALKDKDVIVMSVNGDKVTEDGRILTDRGQRIRDVRTGPDGYLYVLTDESSGELL
KVSPRN

Chaperone SurA (SurA)
PET300_SurA (SurA_frag_for + SurA_frag_rev + PET300_for + PET300_rev)
MW: 49581 Da, ε: 32430 M^{-1} cm^{-1}
Gene sequence

ATGCATCATCATCATCATCACATCACAAGTTTGTACAAAAAAGCAGGCTTTCTGGGCGTC
GCTTTGCTGAGTGGCGCGGTGCATGCCGCGGTGCAACCTCTTGATCGCGTGGTGGCTA
TCGTCGACAACGACGTGGTCATGCAAAGCCAGCTGGACCAGCGTGTCCATGAGGTCCA
GCAAACCATCGCCAAGCGCGGCGGCGGCGTGCCGCCGACCAGCGCCCTGGAACAGC
AGGTCCTGGAACGCCTGATCGTCGAGAACCTGCAGCTGCAGATCGGCGAGCGCTCTG
GTATCCGCATCACCGACGAAGAGCTGAACCAGGCCATTGGCACCATTGCCCAGCGCAA
TGGCATGTCGCTGGACCAGTTCCGCGCGGCGCTGGCCCGTGACGGCCTGTCGTTCGA
CGACGCTCGCGAGCAGGTCAAGCGCGAGATGATCATCAGCCGCGTGCGCCAACGCCG
CGTTGCCGAGCGCATTCAGGTGTCCGAGCAGGAAGTGAAGAACTTCCTGGCCTCGGAC
CTGGGCAAGATGCAGATGTCGGAAGAGTACCGCCTGGCCAACATCCTCATCCCGACCC
CGGAAGCCGCCAACTCGGACGATATCCAGAAGGCTGCGCGCAAGGTCGGTGACGTGT
ACCAGCAACTGCGCCAGGGTGCCGACTTCGGCCAGATGGCGATTGCCAACTCGGCCA
GCGAAAACGCCCTGGAAGGCGGCGAGATGGGCTGGCGTAAAGCCGGTCAGCTGCCAC
CCGACTTCGCCAAGATGCTCAGCAGCATGCCAGTGGGCGAAATTACCCAGCCTATTCG
CATCCCCAACGGCTTCATCATCCTCAAGCTCGAGGAGAAGCGCGGCGGCAGCGAGAAC
GTGCTGCGCGACGAAGTGCATGTACGCCACATCCTGATCAAGCCAAGCGAGATCCGCA
GCGAGGCGGCCACCGAGCAGCTGGCCGAGCGCCTTTATGATCGGATCAAGAACGGCG
AAGACTTCGGCGAACTGGCCAAGAGCTTCTCGGAAGACCCGGGTTCGGCACTCAACGG
CGGCGACCTCAACTGGGTCGATCCGAACAGCCTGGTACCGGAGTTCCGTGAACAGATG
GCCAATGCCCAGCAAGGCGTAGTGACCAAACCGTTCAAGACCCAGTACGGCTGGCACG
TTCTGGAAGTGCTGGGCCGCCGCGCCACCGACAGCACCGAGCAAGCTCGCGAACAGC
AGGCCCTGAGCGTACTGCGCAACCGCAAGTATGACGAAGAGCTGCAAACCTGGCTGCG
CCAGATCCGCGACGAAGCCTACGTTGAAATCAAGCTGCCTGGCGCTGACCAGGCCGCC
CAGTGA

Protein sequence
MHHHHHHITSLYKKAGFLGVALLSGAVHAAVQPLDRVVAIVDNDVVMQSQLDQRVHEVQQ
TIAKRGGGVPPTSALEQQVLERLIVENLQLQIGERSGIRITDEELNQAIGTIAQRNGMSLDQF
RAALARDGLSFDDAREQVKREMIISRVRQRRVAERIQVSEQEVKNFLASDLGKMQMSEEYR
LANILIPTPEAANSDDIQKAARKVGDVYQQLRQGADFGQMAIANSASENALEGGEMGWRKA
GQLPPDFAKMLSSMPVGEITQPIRIPNGFIILKLEEKRGGSENVLRDEVHVRHILIKPSEIRSEA
ATEQLAERLYDRIKNGEDFGELAKSFSEDPGSALNGGDLNWVDPNSLVPEFREQMANAQQ
GVVTKPFKTQYGWHVLEVLGRRATDSTEQAREQQALSVLRNRKYDEELQTWLRQIRDEAY
VEIKLPGADQAAQ

Periplasmic chaperone PpiD (PpiD)
PET300_PpiD (PpiD_frag_for + PpiD_frag_rev + PET300_for + PET300_rev)
MW: 66756 Da, ε: 27850 M^{-1} cm^{-1}
Gene sequence
ATGCATCATCATCATCATCACATCACAAGTTTGTACAAAAAAGCAGGCTTTGCCGCCACT
CATAGCCAGGACGCCGCCAAGGTGAACGGCCAGACCATCAGCCAGAACGAACTGAGC
CAGGCGGCCGACATGCAGCGCCGTCAACTGATGCAACAGTTGGGCAAGGATTTCGACC
CGGCGCTGTTGGATGACAAGTTGCTGCGAGAGGAGGCACTCAAGGGGCTGATCAGCC
GCAAGCTGCTGCTGCAAGGTGCTGAGGATGCCAAGTTCGCATTCTCCGAGGCCGCGCT
GGATCAGGTGATCCTGCAGACCCCTGAGTTCCAGGTGGACGGCAAGTTCAGCGCTGAG
CGTTTCGACCAGGTGATCCGCCAGATGGGCTACGGTCGCATGCAGTTCCGCGAGATGC
TCGGCGAGGAAATGCTCATCGGCCAACTGCGCACCGGCCTGGCCGGCAGCAGCTTCG
TCACCGATCAACAGGTCGATGCCTTCGCTCGCCTGGAGAAGCAGACCCGCGACTTCGC

CTCCCTGACGTTCAAGGCCGACCCGGCCGCAGTCAAGGTCAGCGACGAAGAGGTCAA
GGCGCACTACGACCAGCACGCCAAGGAGTTCATGTCGCCTGACCAGGTCGTGATCGAC
TACATCGAGCTGAAGAAGTCGGCTTTCTTCGATCAGGTCAAAGTGACTGACGAAGAGCT
CAAGGCCCAGTACGAGAAGGAAATCGCCAACCTGGCCGAGCAGCGCCATGCCGCGCA
CATCCTCATCGAGGTCAACGACAAGGTCACCGACGCCCAGGCCAAGGCCCGCGCCGA
AGAGATCGAGCAGCGTCTGGCCAAGGGTGAGGACTTTGCCGCTTTGGCCAAGGAGTTC
TCTCAGGACCCAGGCTCCGCCAACACCGGTGGTGACCTCGGCTTTGCCGGCCCGGGT
GTATACGACCCGGCCTTCGAAGAGGCGCTGTACAAGCTGCAGGATGGGCAGGTATCTG
CACCGGTGCGCACCGAGTTTGGCTACCATCTGATCAAGCTGCTGGGCGTCCAGGCGCC
AGAAGTACCGAGCTTCGCCAGCCTGAAGGACAAACTGACCCGCGATCTGAAGATGCCG
CTGGTCGAGCAGCGTTATGTCGACGCCAGCAAGCAACTGCAGGATGCTGCCTACGAGG
CTTCCGACCTGGCCCAGCCGGCCCAGGACCTGAACCTCAAGGTGCACACCTCCGCCG
CCTTCGGCCGCGAGGGCGGTGAAGGCATTACCGCCAACCGTGCGGTGGTGCAGGCTG
CGTTCTCTGAAGAAGTGCTGGATGAGGGTGCCAACAGCACCGCCATCGAGCTCGACCC
TGAGACCACTGTGGTGCTGCGCGTCAAGGAACACCGCAAGCCAGAGCAACTGCCGCT
GGACGCTGTGGCCAAGAACATCAGGGAGCACCTGGCCAAAGAGAAGGCGACTGCCGA
ACTCAAGGCCAAGGCAGACAAGCTGATTGGCGGCCTGCGTGACGGTTCCATCGCAGCA
GGCAGCGTGCACGAAGGCCAAGGCTGGAAAGCCTATGAAGCCGTCACCCGTGGTGAG
GACGGTATTGACCCGGCCGAACTGCAGGCACTGTTCCGTCTGGGCAAGCCGCAAGCC
AAGGACAAGCCAGTGTATGGCAGCGTCGTGCTGCGTGACGGTAGCCTGGTGGTGTTGC
AGCTCAAGGGCGTGAACGAAGGCGCTGCCGCCACCGACGAAGAGAAGCAGCAGATTC
GCCGCTACCTCGCGTCACGTGCTGGCCAGCAGGACTTTGCCGCTTACCGCAAGCAGCT
GGAAGCCAATGCAGACATCACCCGTTACTAA

Protein sequence
MHHHHHHITSLYKKAGFAATHSQDAAKVNGQTISQNELSQAADMQRRQLMQQLGKDFDPA
LLDDKLLREEALKGLISRKLLLQGAEDAKFAFSEAALDQVILQTPEFQVDGKFSAERFDQVIR
QMGYGRMQFREMLGEEMLIGQLRTGLAGSSFVTDQQVDAFARLEKQTRDFASLTFKADPA
AVKVSDEEVKAHYDQHAKEFMSPDQVVIDYIELKKSAFFDQVKVTDEELKAQYEKEIANLAE
QRHAAHILIEVNDKVTDAQAKARAEEIEQRLAKGEDFAALAKEFSQDPGSANTGGDLGFAG
PGVYDPAFEEALYKLQDGQVSAPVRTEFGYHLIKLLGVQAPEVPSFASLKDKLTRDLKMPLV
EQRYVDASKQLQDAAYEASDLAQPAQDLNLKVHTSAAFGREGGEGITANRAVVQAAFSEE
VLDEGANSTAIELDPETTVVLRVKEHRKPEQLPLDAVAKNIREHLAKEKATAELKAKADKLIG
GLRDGSIAAGSVHEGQGWKAYEAVTRGEDGIDPAELQALFRLGKPQAKDKPVYGSVVLRD
GSLVVLQLKGVNEGAAATDEEKQQIRRYLASRAGQQDFAAYRKQLEANADITRY

Alcohol dehydrogenase (PedH) without leader peptide sequence

PET29b_PedH_F412V_W561A from TWIST Bioscience

PedH double mutant (F412V+W561A) has been engineered previously to widen the substrate entry tunnel.[27] This variant was used for spiking of recombinantly expressed protein into *E. coli* K-12 lysate as well as in vitro binding and activity studies.

MW: 63097 Da, ε: 141415 M^{-1} cm^{-1} (assuming all pairs of Cys residues form cysteine)

Gene sequence
ATGGCAGTGTCTAACGAGGAGATTCTGCAAGATCCAAAGAATCCACAACAAATTGTAAC
GAACGGATTAGGAGTACAAGGTCAACGTTATTCGCCTTTGGATTTGCTGAACGTTAACA
ATGTGAAAGAATTGCGTCCTGTATGGGCTTTCTCGTTTGGTGGCGAGAAACAACGTGGT
CAACAAGCGCAACCATTAATTAAAGATGGCGTTATGTATCTCACGGGAAGCTATAGCCG
CGTATTTGCGGTTGACGCACGTACTGGTAAGAAGTTGTGGCAGTATGACGCCCGTCTC

CCCGACGATATTCGTCCATGTTGTGATGTTATTAATCGTGGGGTGGCCCTTTATGGTAAT
TTAGTATTCTTTGGAACTCTCGATGCGAAACTTGTAGCGCTTAATAAAGATACGGGTAAA
GTAGTTTGGTCAAAGAAAGTAGCGGATCATAAAGAGGGATATTCGATTAGTGCTGCCCC
CATGATTGTGAACGGAAAATTGATTACCGGTGTAGCAGGTGGTGAATTTGGGGTTGTCG
GGAAAATTCAAGCATATAATCCCGAAAATGGTGAGTTATTGTGGATGCGTCCTACAGTC
GAGGGCCACATGGGTTACGTATATAAAGACGGTAAAGCTATTGAAAATGGCATCAGTGG
CGGCGAAGCCGGGAAAACGTGGCCAGGGGATCTCTGGAAAACTGGTGGTGCGGCCCC
CTGGTTAGGCGGCTATTATGATCCGGAGACGAATTTGATTTTATTCGGGACGGGGAATC
CTGCACCTTGGAATAGCCATTTGCGTCCAGGCGATAATCTTTATAGCTCGTCACGTCTT
GCCCTTAATCCAGATGATGGTACGATTAAATGGCATTTTCAATCGACACCCCACGATGG
TTGGGATTTTGATGGTGTGAATGAACTCATTAGTTTTAATTATAAAGATGGTGGAAAAGA
AGTGAAAGCAGCAGCTACCGCGGATCGTAATGGCTTCTTCTATGTATTAGATCGTACGA
ATGGTAAATTTATTCGTGGTTTTCCTTTTGTAGATAAAATTACTTGGGCGACCGGTCTGG
ATAAAGATGGGCGCCCCATTTATAATGATGCAAGTCGTCCTGGGGCCCCGGGGTCGGA
AGCGAAAGGGTCATCCGTATTTGTGGCCCCAGCGGTACTGGGTGCGAAGAATTGGATG
CCAATGGCGTATAATAAAGATACGGGCTTGTTTTATGTACCAAGTAATGAATGGGGAAT
GGATATTTGGAATGAGGGAATTGCATACAAGAAGGGCGCCGCCTTTCTGGGCGCGGGG
TTTACTATTAAACCACTTAACGAGGATTATATTGGTGTTCTTCGTGCAATTGATCCCGTTT
CCGGGAAAGAGGTCTGGCGTCATAAGAATTACGCCCCATTATGGGGTGGCGTTCTTAC
TACGAAAGGGAATTTAGTGTTTACTGGAACTCCTGAAGGGTTTCTTCAAGCGTTTAATGC
CAAAACTGGGGATAAAGTTTGGGAGTTTCAAACTGGTAGCGGTGTATTGGGGTCACCG
GTGACGTGGGAGATGGATGGTGAACAGTATGTCTCCGTGGTGTCGGGTTGGGGTGGT
GCAGTCCCTTTAGCGGGTGGAGAGGTAGCGAAGCGTGTGAAAGATTTTAATCAAGGTG
GAATGTTGTGGACATTTAAACTGCCGAAACAACTGCAACAGACCGCCTCGGTTAAACCG
CTCGAGCACCACCACCACCACCACTGA

Protein sequence
MAVSNEEILQDPKNPQQIVTNGLGVQGQRYSPLDLLNVNNVKELRPVWAFSFGGEKQRGQ
QAQPLIKDGVMYLTGSYSRVFAVDARTGKKLWQYDARLPDDIRPCCDVINRGVALYGNLVF
FGTLDAKLVALNKDTGKVVWSKKVADHKEGYSISAAPMIVNGKLITGVAGGEFGVVGKIQAY
NPENGELLWMRPTVEGHMGYVYKDGKAIENGISGGEAGKTWPGDLWKTGGAAPWLGGY
YDPETNLILFGTGNPAPWNSHLRPGDNLYSSSRLALNPDDGTIKWHFQSTPHDGWDFDGV
NELISFNYKDGGKEVKAAATADRNGFFYVLDRTNGKFIRGFPFVDKITWATGLDKDGRPIYN
DASRPGAPGSEAKGSSVFVAPAVLGAKNWMPMAYNKDTGLFYVPSNEWGMDIWNEGIAY
KKGAAFLGAGFTIKPLNEDYIGVLRAIDPVSGKEVWRHKNYAPLWGGVLTTKGNLVFTGTPE
GFLQAFNAKTGDKVWEFQTGSGVLGSPVTWEMDGEQYVSVVSGWGGAVPLAGGEVAKR
VKDFNQGGMLWTFKLPKQLQQTASVKPLEHHHHHH

Alcohol dehydrogenase (PedH) with leader peptide sequence
PET29b_PedH (PedH_frag_for + PedH_frag_rev + PET29b_for + PET29b_rev)
Native PedH was used for all proteomics experiments.
MW: 64884 Da, ε: 146915 M^{-1} cm^{-1} (assuming all pairs of Cys residues form cysteine)
Gene sequence
ATGACCCGATCCCCACGTCGCCCCTTGTTCGCCGTGAGCCTGGTGCTCAGCGCCATGC
TGCTTGCCGGCGCGGCTCACGCCGCTGTCAGCAATGAAGAAATCCTCCAGGACCCGAA
GAACCCGCAGCAGATCGTGACCAATGGCCTGGGCGTGCAGGGCCAGCGCTACAGCCC
GCTGGACCTGCTCAATGTCAATAACGTCAAGGAGCTGCGCCCGGTCTGGGCGTTCTCC
TTCGGCGGGGAGAAGCAGCGCGGCCAGCAGGCCCAGCCGCTGATCAAGGACGGGGT

GATGTACCTGACCGGCTCCTACTCGCGGGTGTTCGCCGTGGATGCCCGCACCGGCAA
GAAACTGTGGCAATACGATGCACGTCTGCCGGATGACATCCGCCCCTGCTGCGACGTA
ATCAACCGCGGCGTCGCGCTGTACGGCAACCTGGTGTTCTTCGGCACGCTGGACGCCA
AGCTGGTGGCCCTGAACAAGGACACCGGCAAGGTGGTCTGGAGCAAGAAGGTCGCCG
ACCACAAAGAAGGCTACTCCATCAGCGCCGCGCCGATGATCGTCAATGGCAAGCTGAT
CACAGGCGTTGCCGGCGGCGAGTTCGGCGTGGTGGGCAAGATCCAGGCGTACAACCC
GGAGAACGGCGAACTGCTGTGGATGCGCCCCACCGTGGAAGGGCACATGGGCTATGT
GTACAAGGATGGCAAGGCGATCGAGAACGGTATTTCCGGCGGTGAGGCGGGCAAGAC
CTGGCCTGGCGACCTGTGGAAGACCGGCGGCGCCGCGCCGTGGCTGGGGGGTTACT
ACGACCCTGAAACCAACCTGATCCTGTTTGGTACCGGTAACCCGGCGCCGTGGAACTC
GCACCTGCGCCCCGGTGACAACCTGTACTCCTCCTCACGCCTGGCACTGAACCCGGAC
GACGGCACCATCAAGTGGCACTTCCAGAGCACGCCGCATGACGGCTGGGACTTCGAC
GGCGTCAACGAGCTGATCTCGTTCAACTACAAGGACGGCGGCAAGGAGGTCAAGGCTG
CCGCCACGGCAGACCGCAACGGTTTCTTCTACGTGCTCGACCGCACCAACGGCAAGTT
CATCCGCGGCTTCCCCTTCGTGGACAAGATCACCTGGGCCACTGGCCTGGACAAGGAC
GGCCGGCCGATCTACAACGACGCCAGCCGCCCGGGCGCACCCGGCAGCGAGGCCAA
GGGCAGCTCGGTGTTCGTCGCGCCGGCCTTCCTCGGCGCCAAGAACTGGATGCCGAT
GGCCTACAACAAGGACACAGGGCTGTTCTACGTGCCGTCCAACGAGTGGGGCATGGAC
ATCTGGAACGAAGGCATCGCCTATAAGAAAGGTGCGGCGTTCCTCGGTGCCGGCTTCA
CCATCAAGCCGCTCAATGAAGACTACATCGGCGTGCTGCGCGCCATCGACCCGGTCAG
CGGCAAGGAAGTGTGGCGCCACAAGAACTATGCGCCGCTGTGGGGCGGTGTGCTGAC
CACCAAGGGCAACCTGGTGTTCACGGGCACGCCAGAGGGCTTCCTGCAGGCATTCAAC
GCCAAGACCGGCGACAAGGTCTGGGAATTCCAGACCGGCTCGGGCGTGCTCGGCTCG
CCCGTCACCTGGGAAATGGACGGCGAGCAATACGTTTCGGTAGTCTCCGGCTGGGGC
GGCGCGGTGCCGCTGTGGGGCGGCGAAGTGGCCAAACGGGTCAAGGACTTCAACCAG
GGCGGCATGCTCTGGACCTTCAAGTTGCCCAAGCAGTTGCAGCAAACGGCAAGCGTCA
AGCCATAA

Protein sequence
MTRSPRRPLFAVSLVLSAMLLAGAAHAAVSNEEILQDPKNPQQIVTNGLGVQGQRYSPLDLL
NVNNVKELRPVWAFSFGGEKQRGQQAQPLIKDGVMYLTGSYSRVFAVDARTGKKLWQYD
ARLPDDIRPCCDVINRGVALYGNLVFFGTLDAKLVALNKDTGKVVWSKKVADHKEGYSISAA
PMIVNGKLITGVAGGEFGVVGKIQAYNPENGELLWMRPTVEGHMGYVYKDGKAIENGISGG
EAGKTWPGDLWKTGGAAPWLGGYYDPETNLILFGTGNPAPWNSHLRPGDNLYSSSRLALN
PDDGTIKWHFQSTPHDGWDFDGVNELISFNYKDGGKEVKAAATADRNGFFYVLDRTNGKFI
RGFPFVDKITWATGLDKDGRPIYNDASRPGAPGSEAKGSSVFVAPAFLGAKNWMPMAYNK
DTGLFYVPSNEWGMDIWNEGIAYKKGAAFLGAGFTIKPLNEDYIGVLRAIDPVSGKEVWRHK
NYAPLWGGVLTTKGNLVFTGTPEGFLQAFNAKTGDKVWEFQTGSGVLGSPVTWEMDGEQ
YVSVVSGWGGAVPLWGGEVAKRVKDFNQGGMLWTFKLPKQLQQTASVKP

Holliday junction branch migration complex subunit RuvB
PET300_RuvB (RuvB_frag_for + RuvB_frag_rev + PET300_for + PET300_rev)
MW: 39106 Da, ε: 17420 M^{-1} cm^{-1}
Gene sequence
ATGCATCATCATCATCATCACATCACAAGTTTGTACAAAAAAGCAGGCTTTATTGAAGCA
GACCGTCTGATTTCTGCCGGTACCACTTTGCCGGAAGATGTAGCAGATCGCGCCATTC
GCCCCAAATTACTGGAAGAGTATGTTGGTCAGCCGCAGGTTCGTTCACAGATGGAGATT
TTCATCAAAGCAGCGAAACTGCGCGGCGATGCCCTCGATCATTTGTTGATTTTTGGTCC

TCCGGGGTTGGGTAAAACTACGCTTGCCAACATTGTCGCCAATGAAATGGGCGTTAATT
TACGCACGACTTCTGGTCCGGTGCTGGAAAAGGCGGGCGATTTGGCTGCGATGCTCAC
TAACCTTGAACCGCATGACGTGCTGTTTATTGATGAGATCCACCGTCTATCGCCAGTTG
TTGAAGAAGTGCTGTACCCGGCAATGGAAGACTACCAACTGGATATCATGATTGGTGAA
GGTCCGGCGGCACGCTCCATTAAAATTGATTTGCCGCCGTTTACCCTGATTGGTGCAAC
CACGCGCGCAGGTTCGCTGACATCACCGTTGCGCGACCGTTTTGGTATTGTGCAACGT
CTGGAGTTTTATCAGGTGCCGGATCTGCAATATATCGTCAGTCGCAGCGCACGCTTTAT
GGGGCTTGAGATGAGTGATGACGGCGCGCTGGAAGTTGCTCGTCGCGCTCGCGGTAC
GCCGCGCATTGCCAACCGTCTGCTGCGTCGAGTGCGTGATTTCGCCGAAGTGAAGCAC
GATGGCACCATCTCGGCAGATATCGCTGCTCAGGCGCTGGATATGTTGAATGTCGATG
CTGAAGGTTTCGATTATATGGACCGCAAATTGTTGCTGGCGGTAATCGATAAGTTCTTTG
GTGGACCTGTAGGTCTGGATAACCTGGCGGCAGCCATTGGCGAAGAACGTGAAACCAT
TGAGGATGTGCTGGAACCTTATTTGATTCAGCAAGGCTTTTTGCAGCGTACACCGCGTG
GGCGTATGGCGACGACGCGGGCGTGGAATCACTTTGGCATAACGCCGCCAGAAATGC
CGTAA

Protein sequence
MHHHHHHITSLYKKAGFIEADRLISAGTTLPEDVADRAIRPKLLEEYVGQPQVRSQMEIFIKA
AKLRGDALDHLLIFGPPGLGKTTLANIVANEMGVNLRTTSGPVLEKAGDLAAMLTNLEPHDV
LFIDEIHRLSPVVEEVLYPAMEDYQLDIMIGEGPAARSIKIDLPPFTLIGATTRAGSLTSPLRDR
FGIVQRLEFYQVPDLQYIVSRSARFMGLEMSDDGALEVARRARGTPRIANRLLRRVRDFAE
VKHDGTISADIAAQALDMLNVDAEGFDYMDRKLLLAVIDKFFGGPVGLDNLAAAIGEERETIE
DVLEPYLIQQGFLQRTPRGRMATTRAWNHFGITPPEMP

Holliday junction branch migration complex subunit RuvB mutant (RuvB^{K26H})

PET300_ RuvB^{K26H} (RuvB^{K26H}_frag_for + RuvB^{K26H}_frag_rev + PET300_for + PET300_rev)
MW: 39115 Da, ε: 17420 M^{-1} cm^{-1}
Gene sequence
ATGCATCATCATCATCATCACATCACAAGTTTGTACAAAAAAGCAGGCTTTATTGAAGCA
GACCGTCTGATTTCTGCCGGTACCACTTTGCCGGAAGATGTAGCAGATCGCGCCATTC
GCCCCCATTTACTGGAAGAGTATGTTGGTCAGCCGCAGGTTCGTTCACAGATGGAGATT
TTCATCAAAGCAGCGAAACTGCGCGGCGATGCCCTCGATCATTTGTTGATTTTTGGTCC
TCCGGGGTTGGGTAAAACTACGCTTGCCAACATTGTCGCCAATGAAATGGGCGTTAATT
TACGCACGACTTCTGGTCCGGTGCTGGAAAAGGCGGGCGATTTGGCTGCGATGCTCAC
TAACCTTGAACCGCATGACGTGCTGTTTATTGATGAGATCCACCGTCTATCGCCAGTTG
TTGAAGAAGTGCTGTACCCGGCAATGGAAGACTACCAACTGGATATCATGATTGGTGAA
GGTCCGGCGGCACGCTCCATTAAAATTGATTTGCCGCCGTTTACCCTGATTGGTGCAAC
CACGCGCGCAGGTTCGCTGACATCACCGTTGCGCGACCGTTTTGGTATTGTGCAACGT
CTGGAGTTTTATCAGGTGCCGGATCTGCAATATATCGTCAGTCGCAGCGCACGCTTTAT
GGGGCTTGAGATGAGTGATGACGGCGCGCTGGAAGTTGCTCGTCGCGCTCGCGGTAC
GCCGCGCATTGCCAACCGTCTGCTGCGTCGAGTGCGTGATTTCGCCGAAGTGAAGCAC
GATGGCACCATCTCGGCAGATATCGCTGCTCAGGCGCTGGATATGTTGAATGTCGATG
CTGAAGGTTTCGATTATATGGACCGCAAATTGTTGCTGGCGGTAATCGATAAGTTCTTTG
GTGGACCTGTAGGTCTGGATAACCTGGCGGCAGCCATTGGCGAAGAACGTGAAACCAT
TGAGGATGTGCTGGAACCTTATTTGATTCAGCAAGGCTTTTTGCAGCGTACACCGCGTG
GGCGTATGGCGACGACGCGGGCGTGGAATCACTTTGGCATAACGCCGCCAGAAATGC
CGTAA

Protein sequence
MHHHHHHITSLYKKAGFIEADRLISAGTTLPEDVADRAIRPHLLEEYVGQPQVRSQMEIFIKA
AKLRGDALDHLLIFGPPGLGKTTLANIVANEMGVNLRTTSGPVLEKAGDLAAMLTNLEPHDV
LFIDEIHRLSPVVEEVLYPAMEDYQLDIMIGEGPAARSIKIDLPPFTLIGATTRAGSLTSPLRDR
FGIVQRLEFYQVPDLQYIVSRSARFMGLEMSDDGALEVARRARGTPRIANRLLRRVRDFAE
VKHDGTISADIAAQALDMLNVDAEGFDYMDRKLLLAVIDKFFGGPVGLDNLAAAIGEERETIE
DVLEPYLIQQGFLQRTPRGRMATTRAWNHFGITPPEMP

Holliday junction branch migration complex subunit RuvB mutant (RuvB^{K46H})
PET300_ RuvB^{K46H} (RuvB^{K46H}_frag_for1 + RuvB^{K46H}_frag_rev1 + RuvB^{K46H}_frag_for2 + RuvB^{K46H}_frag_rev2 + PET300_for + PET300_rev)
MW: 39115 Da, ε: 17420 M^{-1} cm^{-1}
Gene sequence
ATGCATCATCATCATCATCACATCACAAGTTTGTACAAAAAAGCAGGCTTTATTGAAGCA
GACCGTCTGATTTCTGCCGGTACCACTTTGCCGGAAGATGTAGCAGATCGCGCCATTC
GCCCCAAATTACTGGAAGAGTATGTTGGTCAGCCGCAGGTTCGTTCACAGATGGAGATT
TTCATCCATGCAGCGAAACTGCGCGGCGATGCCCTCGATCATTTGTTGATTTTTGGTCC
TCCGGGGTTGGGTAAAACTACGCTTGCCAACATTGTCGCCAATGAAATGGGCGTTAATT
TACGCACGACTTCTGGTCCGGTGCTGGAAAAGGCGGGCGATTTGGCTGCGATGCTCAC
TAACCTTGAACCGCATGACGTGCTGTTTATTGATGAGATCCACCGTCTATCGCCAGTTG
TTGAAGAAGTGCTGTACCCGGCAATGGAAGACTACCAACTGGATATCATGATTGGTGAA
GGTCCGGCGGCACGCTCCATTAAAATTGATTTGCCGCCGTTTACCCTGATTGGTGCAAC
CACGCGCGCAGGTTCGCTGACATCACCGTTGCGCGACCGTTTTGGTATTGTGCAACGT
CTGGAGTTTTATCAGGTGCCGGATCTGCAATATATCGTCAGTCGCAGCGCACGCTTTAT
GGGGCTTGAGATGAGTGATGACGGCGCGCTGGAAGTTGCTCGTCGCGCTCGCGGTAC
GCCGCGCATTGCCAACCGTCTGCTGCGTCGAGTGCGTGATTTCGCCGAAGTGAAGCAC
GATGGCACCATCTCGGCAGATATCGCTGCTCAGGCGCTGGATATGTTGAATGTCGATG
CTGAAGGTTTCGATTATATGGACCGCAAATTGTTGCTGGCGGTAATCGATAAGTTCTTTG
GTGGACCTGTAGGTCTGGATAACCTGGCGGCAGCCATTGGCGAAGAACGTGAAACCAT
TGAGGATGTGCTGGAACCTTATTTGATTCAGCAAGGCTTTTTGCAGCGTACACCGCGTG
GGCGTATGGCGACGACGCGGGCGTGGAATCACTTTGGCATAACGCCGCCAGAAATGC
CGTAA

Protein sequence
MHHHHHHITSLYKKAGFIEADRLISAGTTLPEDVADRAIRPKLLEEYVGQPQVRSQMEIFIHA
AKLRGDALDHLLIFGPPGLGKTTLANIVANEMGVNLRTTSGPVLEKAGDLAAMLTNLEPHDV
LFIDEIHRLSPVVEEVLYPAMEDYQLDIMIGEGPAARSIKIDLPPFTLIGATTRAGSLTSPLRDR
FGIVQRLEFYQVPDLQYIVSRSARFMGLEMSDDGALEVARRARGTPRIANRLLRRVRDFAE
VKHDGTISADIAAQALDMLNVDAEGFDYMDRKLLLAVIDKFFGGPVGLDNLAAAIGEERETIE
DVLEPYLIQQGFLQRTPRGRMATTRAWNHFGITPPEMP

2.13. Recombinant Protein Expression and Purification

For pre-cultures, 5 mL LB media containing antibiotic were inoculated with 50 µL *E. coli* BL21 (DE3) glycerol stock containing the respective construct and the culture grew at 37 ºC, 200 rpm for 12 h. 2 L LB media supplemented with antibiotic was inoculated with pre-culture (1:100). Then it was incubated at 37 ºC, 200 rpm until OD_{600} reached 0.6 – 0.8, and protein overexpression was induced by adding isopropyl β-D-thiogalactopyranoside (IPTG, final concentration: 0.5 mM). The expression was carried out at 18 ºC, 200 rpm for 16 h. Cell pellets were harvest by centrifugation (6000 g, 10 min, 4 ºC), washed with cold PBS, and stored at -20 ºC for further processing.

The frozen cell pellets were resuspended in 40 mL lysis buffer (25 mM HEPES, 150 mM NaCl, 10 mM imidazole, pH 7.5) supplemented with DNAse I (0.05 mg/mL) and lysozyme (0.5 mg/mL) at 4 ºC, and the suspension was incubated for 30 min on ice. The cells were lysed with sonification (4 × 1.5 min, 70% intensity) and clear supernatant was clarified with centrifugation (18000 g, 30 min, 4 ºC). The clear supernatant was performed on a Ni-NTA affinity column (His Trap HP column) installed on ÄKTA pure 25 FPLC protein purification system. The column was washed with 25 mL lysis buffer (25 mM HEPES, 150 mM NaCl, 10 mM imidazole, pH 7.5), 25 mL wash buffer 1 (25 mM HEPES, 1 M NaCl, 10 mM imidazole, pH 7.5), 25 mL wash buffer 2 (25 mM HEPES, 150 mM NaCl, 40 mM imidazole, pH 7.5), which was final eluted with 25 mL elution buffer (25 mM HEPES, 150 mM NaCl, 500 mM imidazole, pH 7.5). The fractions containing protein were combined and dialyzed with a 3 kDa dialysis membrane in storage buffer (25 mM HEPES, 150 mM NaCl, pH 7.5) overnight at 4 ºC, in which the storage buffer was changed once in between. The combined dialysis protein was concentrated with Amicon centrifuge filter (10 KDa cutoff, 4000 g, 4 ºC). If the purity of proteins were not high enough, further purification with size-exclusion chromatography (SEC) column of HiLoad 16 / 600 Superdex 200 increase can be performed. The Superdex 200 increase installed on ÄKTA pure 25 FPLC was equilibrated with 30 mL storage buffer, then loaded with concentrated protein fractions, finally eluted with 30 mL storage buffer. Fractions were analyzed by FPLC chromatography and SDS-PAGE. The combined fractions of proteins of interest were concentrated with Amicon centrifuge filter (10 KDa cutoff, 4000 g, 4 ºC). The concentration of the proteins was determined by Nanodrop, and then frozen in liquid nitrogen and storage at -80 ºC for further use.

2.14. UV/vis Absorption-Based Binding Assay for PQQ and PQQ-Derived Probes

<u>Procedure for the known **PQQ** enzymes YliI and PedH:</u> 50 µM cofactor-free protein in HEPES buffer (25 mM HEPES, 150 mM NaCl, pH 7.5) was incubated with 3-fold molar excess **PQQ** or **PQQ**-derived probes and 3-fold molar excess $CaCl_2$ or $LaCl_3$ (total volume 500 µL) at 4 ºC overnight or 25 ºC for 2 h, respectively. Unbound **PQQ** or **PQQ**-derived probe was removed using a desalting column (PD MiniTrap G-25), eluting with 1 mL buffer, thus leading to a final protein concentration of ca. 25 µM. Negative controls included protein without **PQQ** (**PQQ**-derived probes) and free **PQQ** (**PQQ**-derived probes) without protein. These samples were also treated with 3-fold molar excess $CaCl_2$ or $LaCl_3$, and free metal was removed using the desalting column.

Absorption spectra of all samples were recorded on a *Jasco* V-750 photometer (*JASCO* Deutschland GmbH) at room temperature (data interval: 1 nm, bandwidth: 0.2 nm, response: 0.24 s, path length: 1 cm). Background absorption was corrected by recording a blank spectrum in advance.

<u>Validation of **PQQ** binding to putative quinoproteins identified from chemoprotemic profiling:</u> 200 µM of the recombinantly expressed and purified proteins in HEPES buffer (25 mM HEPES, 150 mM NaCl, pH 7.5) were incubated with 3-fold molar excess **PQQ** and 3-fold molar excess $CaCl_2$ or $MgCl_2$ (total volume 500 µL) at 4 ºC overnight. Unbound **PQQ** was removed using a desalting column (PD MiniTrap G-25), eluting with 1 mL buffer, thus leading to a final protein concentration of ca. 100 µM. Negative controls included protein without **PQQ** and free **PQQ** without protein. These samples were also treated with 3-fold molar excess $CaCl_2$ or $MgCl_2$, and free metal was removed using the desalting column.
Absorption spectra of all samples were recorded on a Varioskan Lux Multimode Microplate Reader (Thermo Fisher Scientific Inc) in a cycloolefin cellview 96 well microplate (Greiner BIO-ONE) at room temperature (data interval: 1 nm). Background absorption was corrected by recording a buffer spectrum in advance.

2.15. Coupled Colorimetric Enzyme Activity Assay for PQQ-dependent Sugar and Alcohol Dehydrogenases

YliI and PedH samples were reconstituted with **PQQ** (or **PQQ**-derived probe) and $CaCl_2$ or $LaCl_3$ as described in **2.14**, respectively. The enzymatic activity was measured in a coupled colorimetric assay using 2,6-dichloro-phenolindophenol (DCPIP) and phenazine ethosulfate (PES).[28, 29] The 2x assay buffer consisted of 200 µM DCPIP and 2 mM PES in HEPES buffer (25 mM HEPES, 150 mM NaCl, pH 7.5).
For the spectroscopic measurements, 250 µL 2x assay buffer and 250 µL substrate stock solution (2.5 M glucose or 100 mM EtOH) in HEPES buffer were added to a Quartz cuvette (d = 1cm), followed by the addition of 2 µL of 25 µM reconstituted enzyme. Glucose was used as the substrate for YliI and EtOH for PedH. The reactions were mixed quickly and the decrease in absorption at 600 nm was measured for 900 s using a *Jasco* V-750 photometer.

Scheme S2. Coupled Colorimetric Enzyme Activity Assay

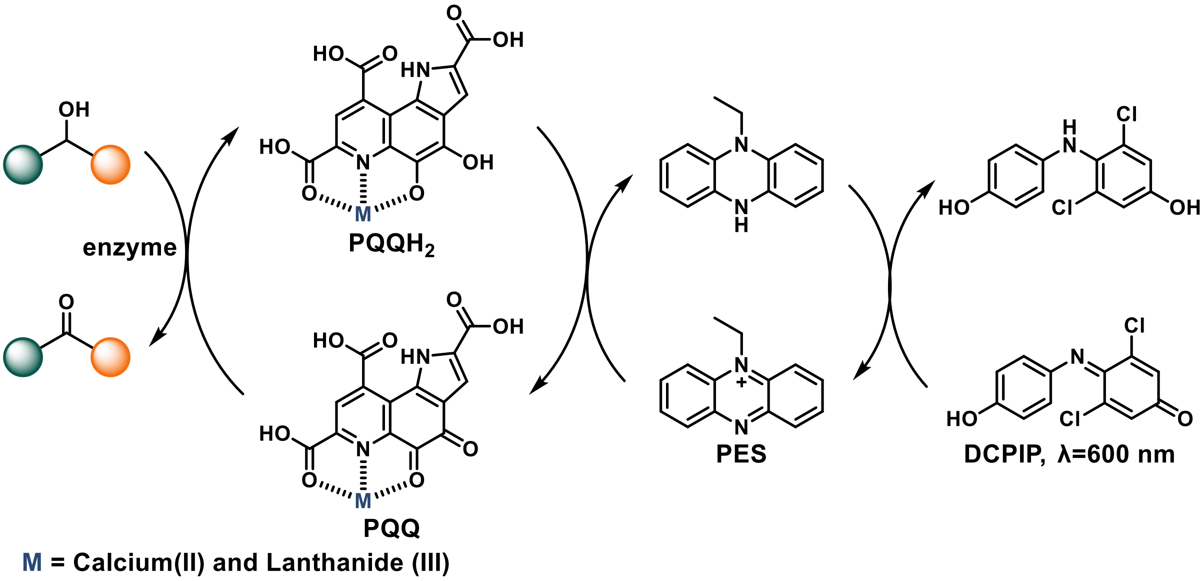

2.16. Intact Protein Mass Spectrometry (IPMS) Experiments

High-resolution IPMS was performed to validate the recombinantly expressed proteins and potential covalent modifications by **PQQ**. In order to measure the degree of covalent modification by **PQQ**, several conditions were applied here: 1) low concentration: 100 µM

protein in HEPES buffer (25 mM HEPES, 150 mM NaCl, pH 7.5) was treated with 3-fold molar excess **PQQ** and 3-fold molar excess $CaCl_2$ or $MgCl_2$ or $LaCl_3$, and then the samples were incubated at 4 ºC for 14 h. 2) high concentration: 100 μM protein in HEPES buffer (25 mM HEPES, 150 mM NaCl, pH 7.5) was treated with 20-fold molar excess **PQQ** and 20-fold molar excess $CaCl_2$ or $MgCl_2$ or $LaCl_3$ in HEPES buffer, and then the samples were incubated at 37 ºC or 30 ºC for 2 h (30 ºC used for RuvB and PedH). All samples (final volume: 50 μL) were measured on an LTQ-FT Ultra (Thermo Fisher Scientific Inc.) coupled to a Dionex Ultimate 3000 HPLC with an electrospray ionization source (spray voltage 4.0 kV, tube lens 110 V, capillary voltage 48 V, sheath gas 60 a.u., aux gas 10 a.u., sweep gas 0.2 a.u). The mass spectrometer was operated in positive ion mode collecting full scans at high resolution (R = 200, 000) in a range of m/z = 600 – 2000. The protein spectra were deconvoluted using UniDec 7.0.1.[30]

2.17. Computational Modelling of PQQ Probe Binding to YliI and PedH

Computational models of the PQQ-derived probes binding in the active sites of YliI and PedH were generated using AI-based protein structure prediction tools, locally installed Boltz-1[31] and Boltz-2.[32] Binding of **PQQ4** and **PQQ8** with Ca(II) in YliI or La(III) in PedH was assessed. For comparison, the binding mode of native **PQQ** was superimposed for both proteins in Pymol. For PedH, a **PQQ**-bound crystal structure is available (PDB ID: 6ZCV). For YliI, **PQQ** was also modeled into the active site, as the available crystal structure (PDB ID: 2G8S) lacks the cofactor. However, as there are many other **PQQ**-bound structures of closely related and structurally similar sugar dehydrogenases available in the PDB, the native binding mode was unambiguously determined.

3. Supplementary Figures and Tables

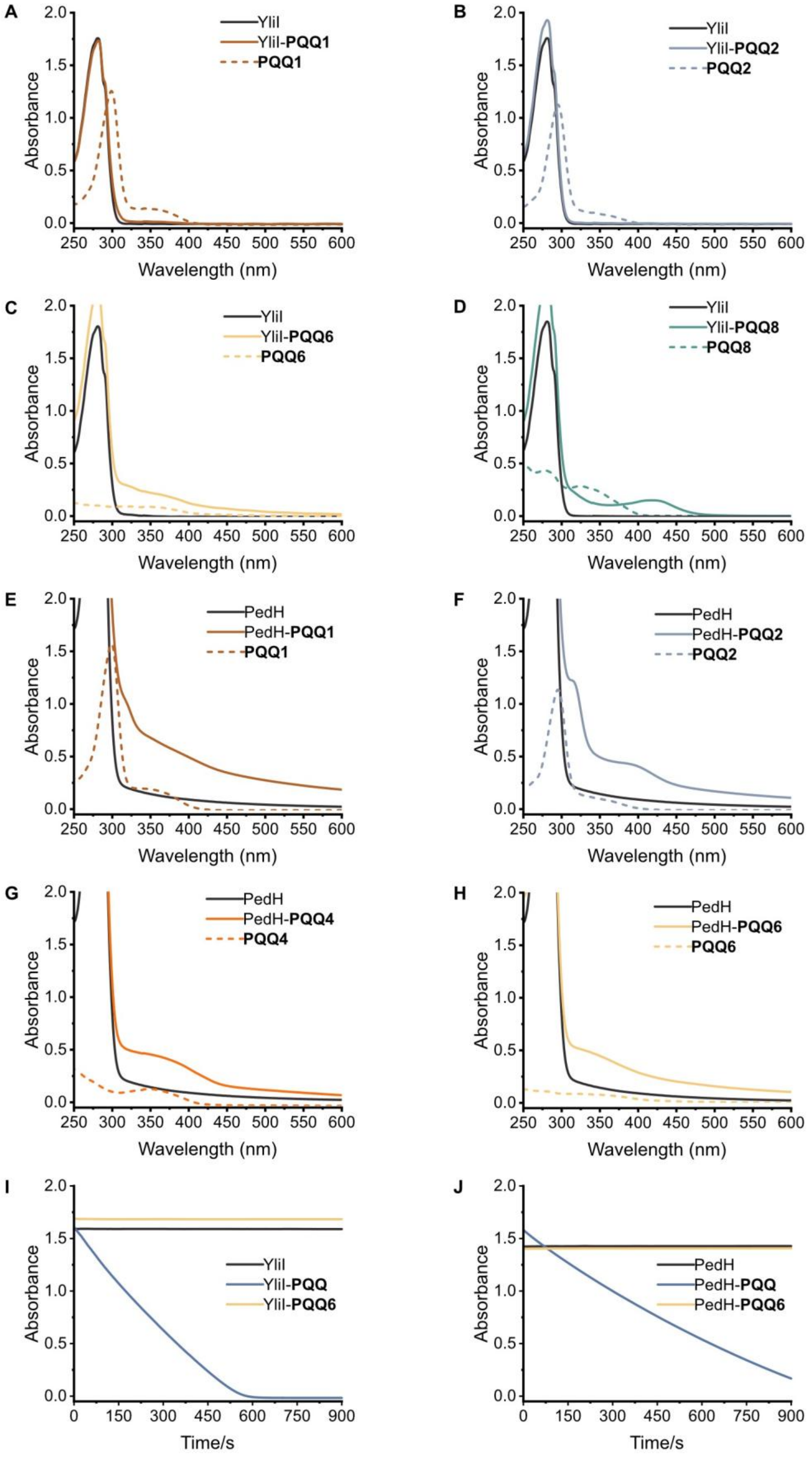

Figure S1. PQQ probes binding to known dehydrogenases. (A) Absorption spectra of apo YliI, YliI reconstituted with **PQQ1**, and free **PQQ1**. (B) Absorption spectra of apo YliI, YliI reconstituted with **PQQ2**, and free **PQQ2**. (C) Absorption spectra of apo YliI, YliI reconstituted with **PQQ6**, and free **PQQ6**. (D) Absorption spectra of apo YliI, YliI reconstituted with **PQQ8**, and free **PQQ8**. (E) Absorption spectra

of apo PedH, PedH reconstituted with **PQQ1**, and free **PQQ1**. (F) Absorption spectra of apo PedH, PedH reconstituted with **PQQ2**, and free **PQQ2**. (G) Absorption spectra of apo PedH, PedH reconstituted with **PQQ4**, and free **PQQ4**. (H) Absorption spectra of apo PedH, PedH reconstituted with **PQQ6**, and free **PQQ6**. (I) Glucose dehydrogenase activity of YliI reconstituted with **PQQ** or **PQQ6** was measured in a coupled colorimetric assay using 100 nM reconstituted enzyme and 1.25 M glucose. (J) Ethanol dehydrogenase activity of PedH reconstituted with **PQQ** or **PQQ6** was measured in a coupled colorimetric assay using 100 nM reconstituted enzyme and 50 mM ethanol.

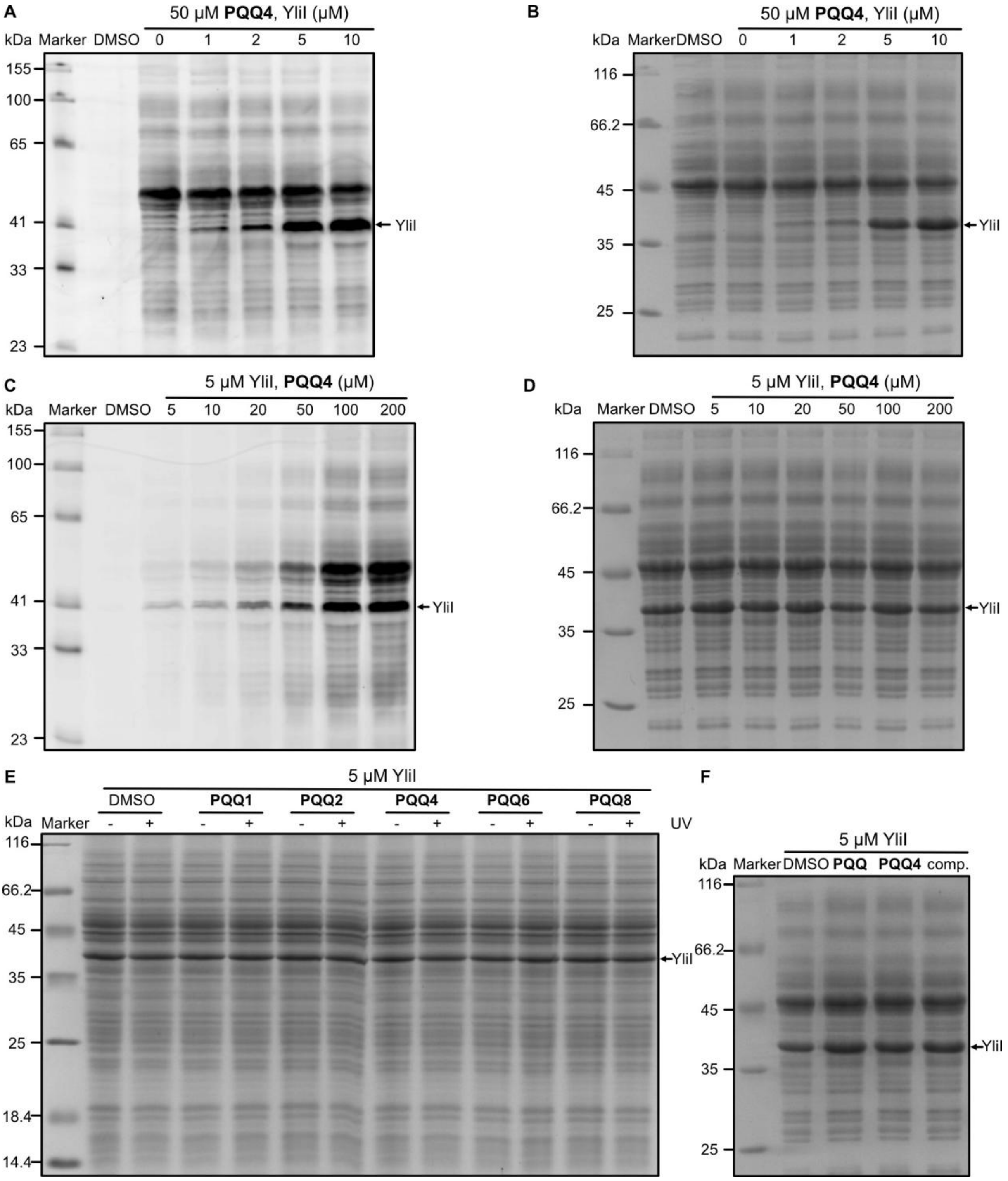

Figure S2. Enrichment of **PQQ**-dependent proteins by different **PQQ** probes analyzed with SDS-PAGE. (A) Fluorescent SDS-PAGE of *E. coli* K-12 lysate, with different concentrations of spiked YliI, labeled by 50 µM **PQQ4** compared to DMSO. (B) Coomassie SDS-PAGE of *E. coli* K-12 lysate, with different concentrations of spiked YliI, labeled by 50 µM **PQQ4** compared to DMSO. (C) Fluorescent SDS-PAGE of *E. coli* K-12 lysate, with 5 µM spiked YliI, labeled by different concentrations of **PQQ4** compared to

DMSO. (D) Coomassie SDS-PAGE of *E. coli* K-12 lysate, with 5 µM spiked YliI, labeled by different concentrations of **PQQ4** compared to DMSO. (E) Coomassie SDS-PAGE of *E. coli* K-12 lysate, with 5 µM spiked YliI, labeled by 50 µM **PQQ** probes compared to DMSO. (F) Coomassie SDS-PAGE of *E. coli* K-12 lysate, with 5 µM spiked YliI, labeled by 50 µM **PQQ4** compared to DMSO, 50 µM **PQQ** and competition (50 µM **PQQ4** + 1000 µM **PQQ**).

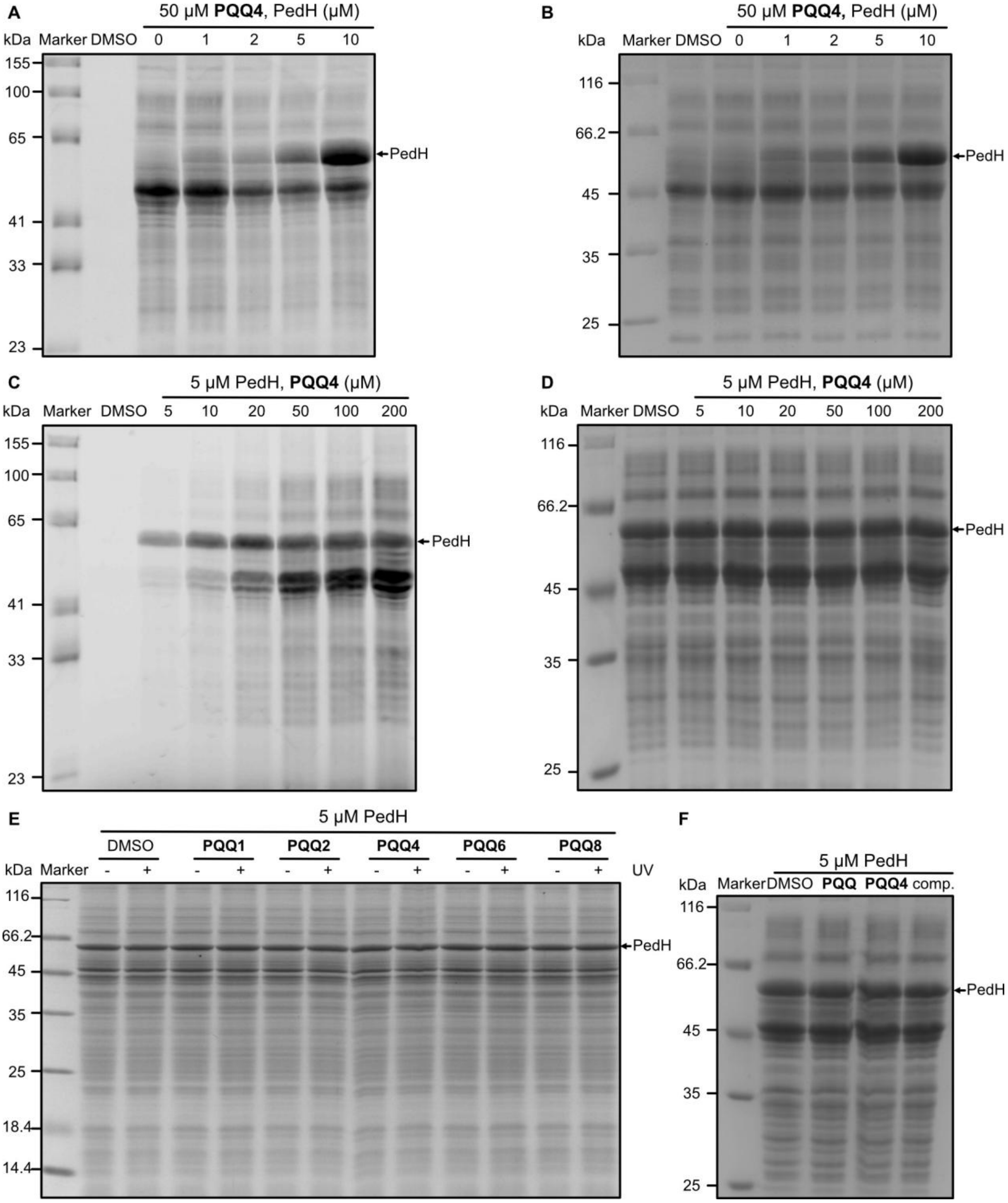

Figure S3. Enrichment of **PQQ**-dependent proteins by different **PQQ** probes with SDS-PAGE. (A) Fluorescent SDS-PAGE of *E. coli* K-12 lysate, with different concentrations of spiked PedH, labeled by 50 µM **PQQ4** compared to DMSO. (B) Coomassie SDS-PAGE of *E. coli* K-12 lysate, with different concentrations of spiked PedH, labeled by 50 µM **PQQ4** compared to DMSO. (C) Fluorescent SDS-PAGE of *E. coli* K-12 lysate, with 5 µM spiked PedH, labeled by different concentrations of **PQQ4** compared to DMSO. (D) Coomassie SDS-PAGE of *E. coli* K-12 lysate, with 5 µM spiked PedH, labeled

by different concentrations of **PQQ4** compared to DMSO. (E) Coomassie SDS-PAGE of *E. coli* K-12 lysate, with 5 µM spiked PedH, labeled by 50 µM **PQQ** probes compared to DMSO. (F) Coomassie SDS-PAGE of *E. coli* K-12 lysate, with 5 µM spiked PedH, labeled by 50 µM **PQQ4** compared to DMSO, 50 µM **PQQ** and competition (50 µM **PQQ4** + 1000 µM **PQQ**).

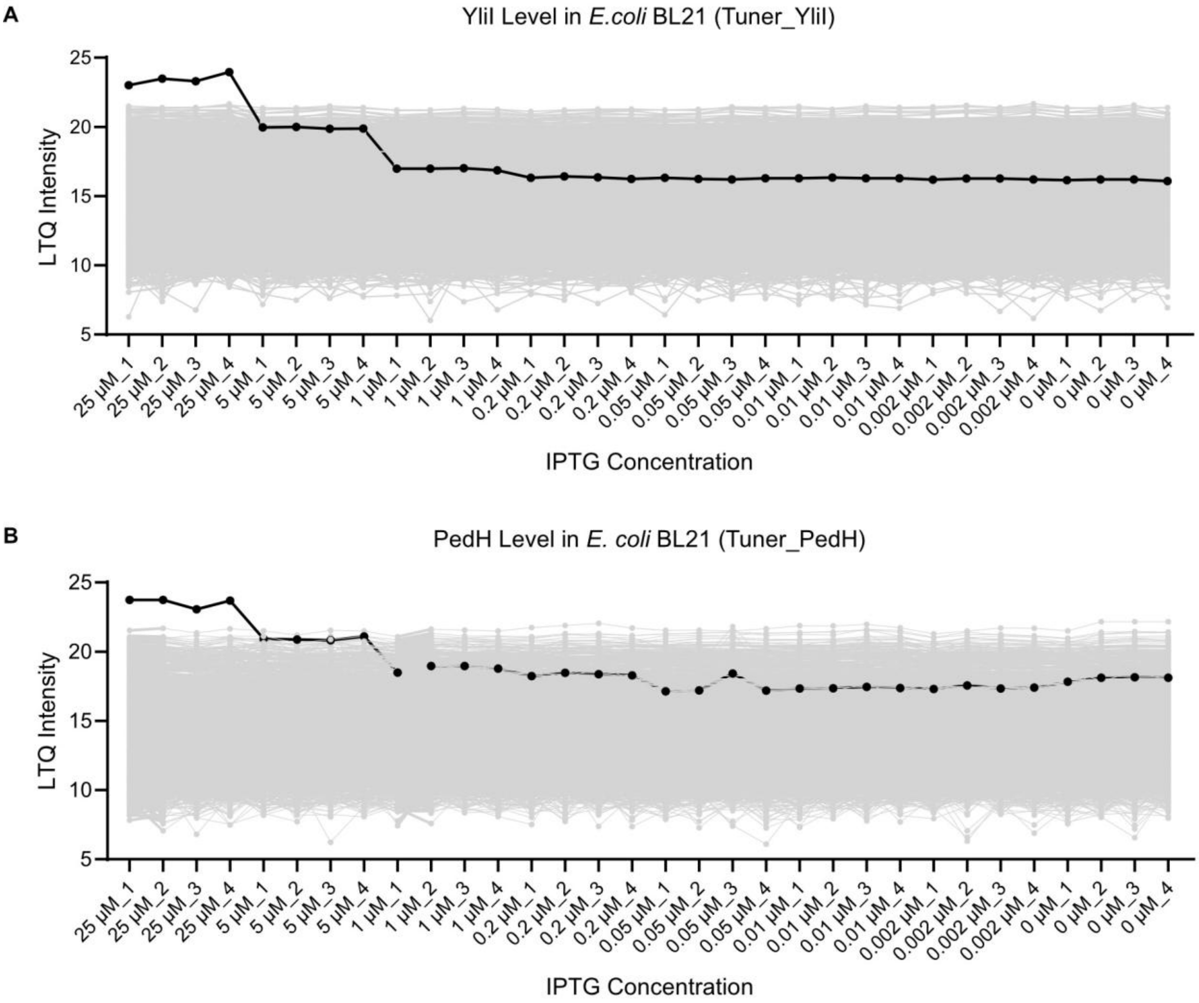

Figure S4. Profile plot of *E. coli* BL 21 (Tuner) cells with different IPTG concentration. (A) Profile plot of *E. coli* BL 21 (Tuner_YliI) with different IPTG concentrations. The experiment was conducted in 4 biological replicates. The dark dots and solid lines represent the label-free quantification (LTQ) intensity of YliI under different IPTG. (B) Profile plot of *E. coli* BL 21 (Tuner_PedH) with different IPTG concentrations. The experiment was conducted in 4 biological replicates. The dark dots and solid lines represent the label-free quantification (LTQ) intensity of PedH under different IPTG.

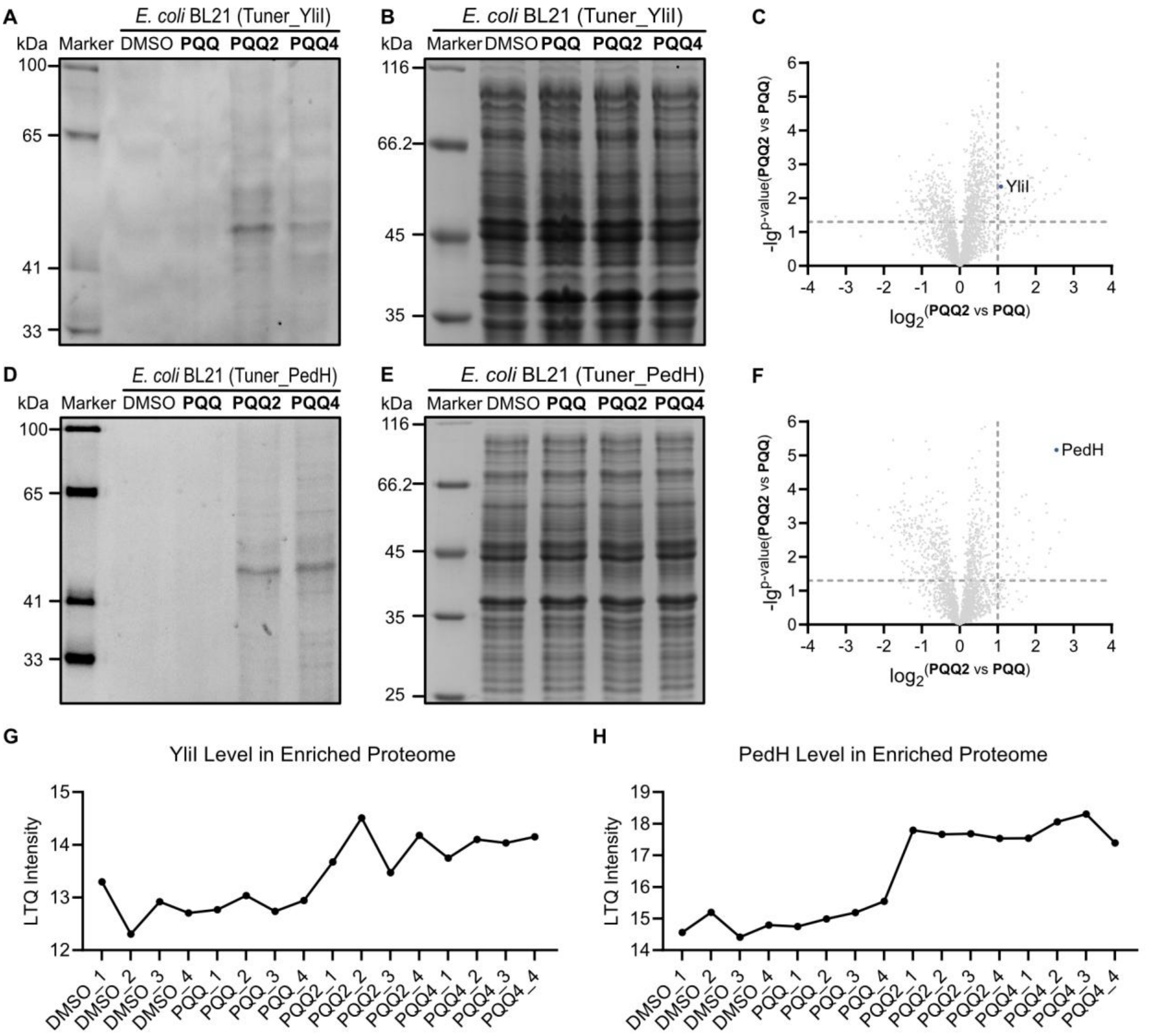

Figure S5. Enrichment of **PQQ**-dependent proteins by chemical proteomics in *E. coli* BL 21 (Tuner) cells. (A) Fluorescent SDS-PAGE of *E. coli* BL21 (Tuner_YliI) in situ labeled by 50 μM **PQQ** probes compared to DMSO and 50 μM **PQQ**. (B) Coomassie SDS-PAGE of *E. coli* BL21 (Tuner_YliI) in situ labeled by 50 μM **PQQ** probes compared to DMSO and 50 μM **PQQ**. (C) Volcano plot of *E. coli* BL21 (Tuner_YliI) treated with 50 μM **PQQ2** compared to 50 μM **PQQ**. The experiment was conducted in 4 biological replicates. The vertical and horizontal dashed lines represent a $\log_2$-fold change of 1 and a -$\log_{10}$ p-value of 1.3, respectively. (D) Fluorescent SDS-PAGE of *E. coli* BL21 (Tuner_PedH) in situ labeled by 50 μM **PQQ** probes compared to DMSO and 50 μM **PQQ**. (E) Coomassie SDS-PAGE of *E. coli* BL21 (Tuner_PedH) in situ labeled by 50 μM **PQQ** probes compared to DMSO and 50 μM **PQQ**. (F) Volcano plot of *E coli* BL21 (Tuner_PedH) treated with 50 μM **PQQ2** compared to 50 μM **PQQ**. The experimental details and cutoff criteria for volcano plot are as in Figure S5C. (G) Profile plot of YliI labeled by 50 μM different **PQQ** probes in *E. coli* BL21 (Tuner_YliI) compared to DMSO and 50 μM **PQQ**. The experiment was conducted in 4 biological replicates. The dark dots and solid lines represent the LFQ intensity of YliII labeled by different **PQQ** probes. (H) Profile plot of PedH labeled by 50 μM different **PQQ** probes in *E. coli* BL21 (Tuner_PedH) compared to DMSO and 50 μM **PQQ**. The experimental details and representation of dark dots and solid lines for profile plot are as in Figure S5G.

Table S13. YliI and PedH enriched by PQQ2 and PQQ4 in *E. coli* BL21 (Tuner_YliI, Tuner_PedH respectively)

Genes	UniProt ID	Protein	Entry	Fold Change	$-lg^{p\text{-value}}$	q-value	t-value
yliI	P75804	YliI	**PQQ2** vs **PQQ**	1.09	2.34	0.014c	4.40
yliI	P75804	YliI	**PQQ4** vs **PQQ**	1.14	4.21	0	9.88

pedH	Q88JH0	PedH	**PQQ2** vs **PQQ**	2.55	5.16	0	14.45
pedH	Q88JH0	PedH	**PQQ4** vs **PQQ**	2.70	4.21	0	9.90

YliI was enriched by **PQQ2** and **PQQ4** in *E. coli* BL21 (Tuner_YliI); PedH was enriched by **PQQ2** and **PQQ4** in *E. coli* BL21 (Tuner_PedH)

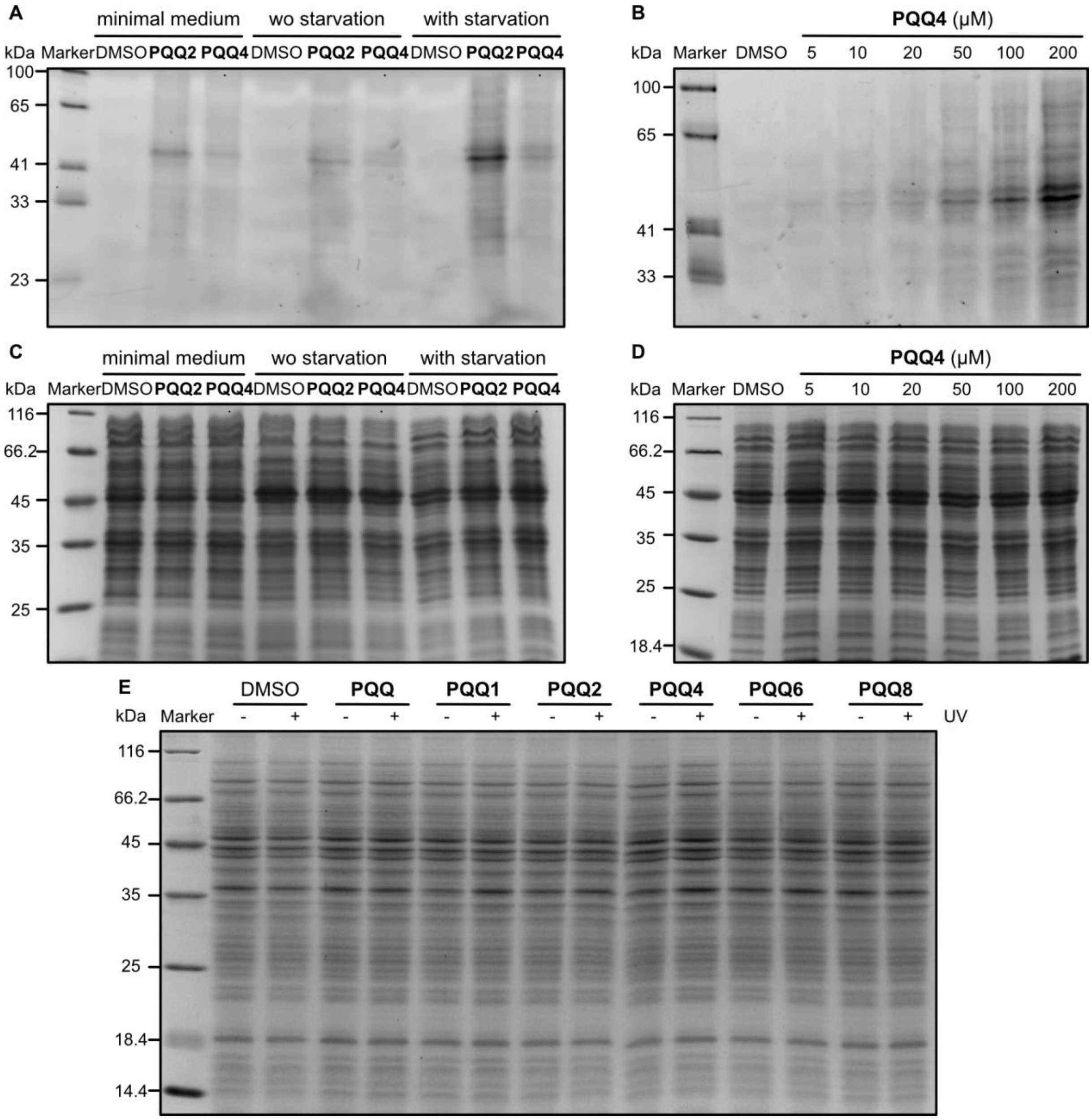

Figure S6. SDS-PAGE of *E. coli* K-12 in situ labeled by different **PQQ** probes. (A) Fluorescent SDS-PAGE of *E. coli* K-12 in situ labeled in different media by 50 μM **PQQ** probes compared to DMSO. (B) Fluorescent SDS-PAGE of *E. coli* K-12 in situ labeled by different concentrations of **PQQ4** compared to DMSO. (C) Coomassie SDS-PAGE of *E. coli* K-12 in situ labeled in different media by 50 μM **PQQ** probes compared to DMSO. (D) Coomassie SDS-PAGE of *E. coli* K-12 in situ labeled by different concentrations of **PQQ4** compared to DMSO. (E) Coomassie SDS-PAGE of *E. coli* K-12 in situ labeled by 50 μM **PQQ** probes with +/- UV treatment compared to DMSO and 50 μM **PQQ**.

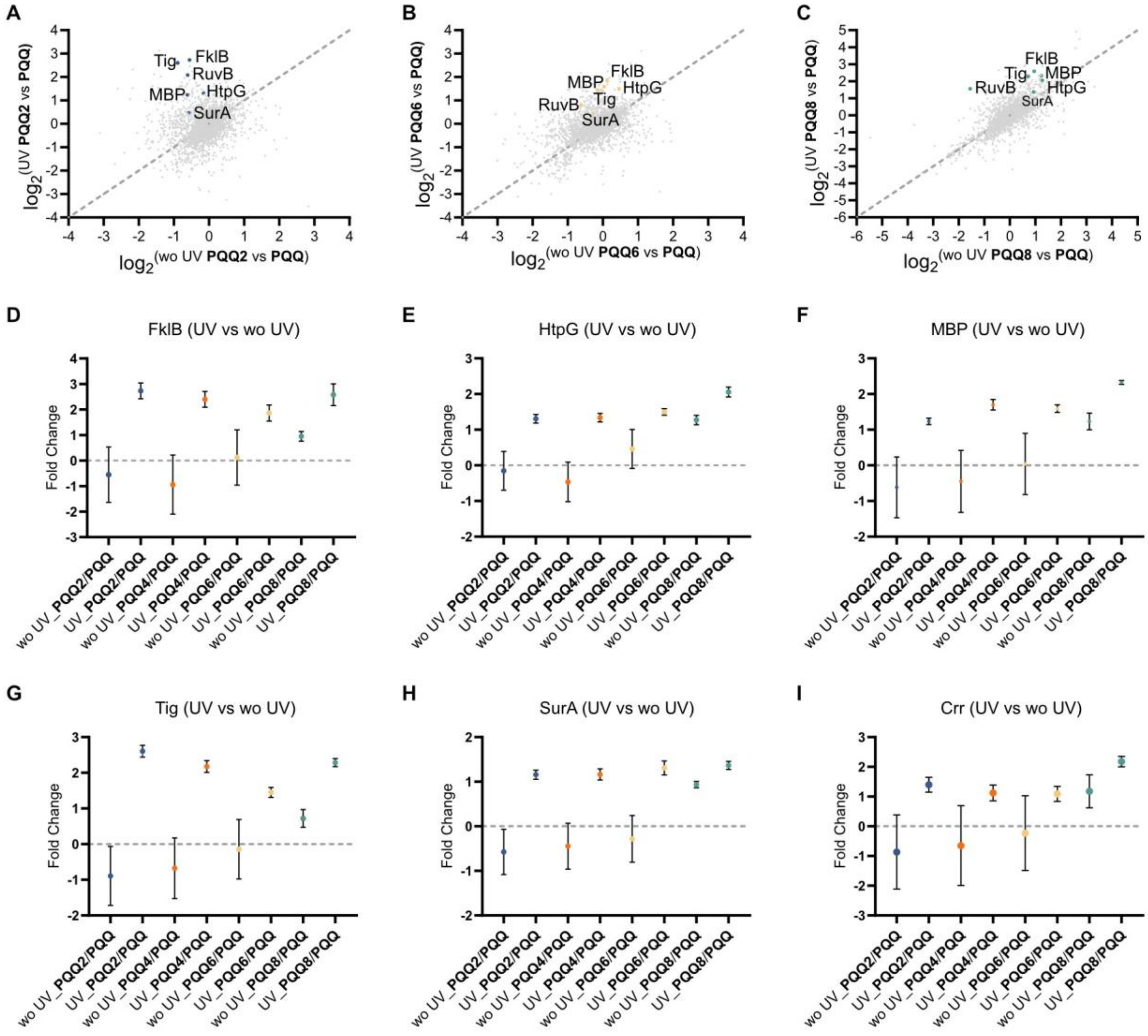

Figure S7. Enrichment of target proteins in *E. coli* K-12 with +/- UV treatment by different **PQQ** probes via proteomics. (A) Scatter plot of *E. coli* K-12 treated with 50 µM **PQQ2** with +/- UV treatment compared to 50 µM **PQQ**. The experiment was conducted in 3 biological replicates. Proteins above the dashed line exhibit higher enrichment upon UV-irradiation which is the case for all significant hits with p-value < 0.05 and q-value < 0.05. (B) Scatter plot of *E. coli* K-12 treated with 50 µM **PQQ6** with +/- UV treatment compared to 50 µM **PQQ**. The experimental details and cutoff criteria for scatter plot are as in Figure S7A. (C) Scatter plot of *E. coli* K-12 treated with 50 µM **PQQ8** with +/- UV treatment compared to 50 µM **PQQ.** The experimental details and cutoff criteria for scatter plot are as in Figure S7A. (D) FklB labeled by different **PQQ** probes under +/- UV conditions. The experiment was conducted with 3 biological replicates. The horizontal dash line represents a $\log_2$-fold change of 0 and the data represents standard error of mean (SEM) of averaged triplicates of n=3 biologically independent experiments, same applies for Figure S7E-I. (E) HtpG labeled by different **PQQ** probes under +/- UV conditions. (F) MBP labeled by different **PQQ** probes under +/- UV conditions. (G) Tig labeled by different **PQQ** probes under +/- UV conditions. (H) SurA labeled by different **PQQ** probes under +/- UV conditions. (I) Crr labeled by different **PQQ** probes under +/- UV conditions.

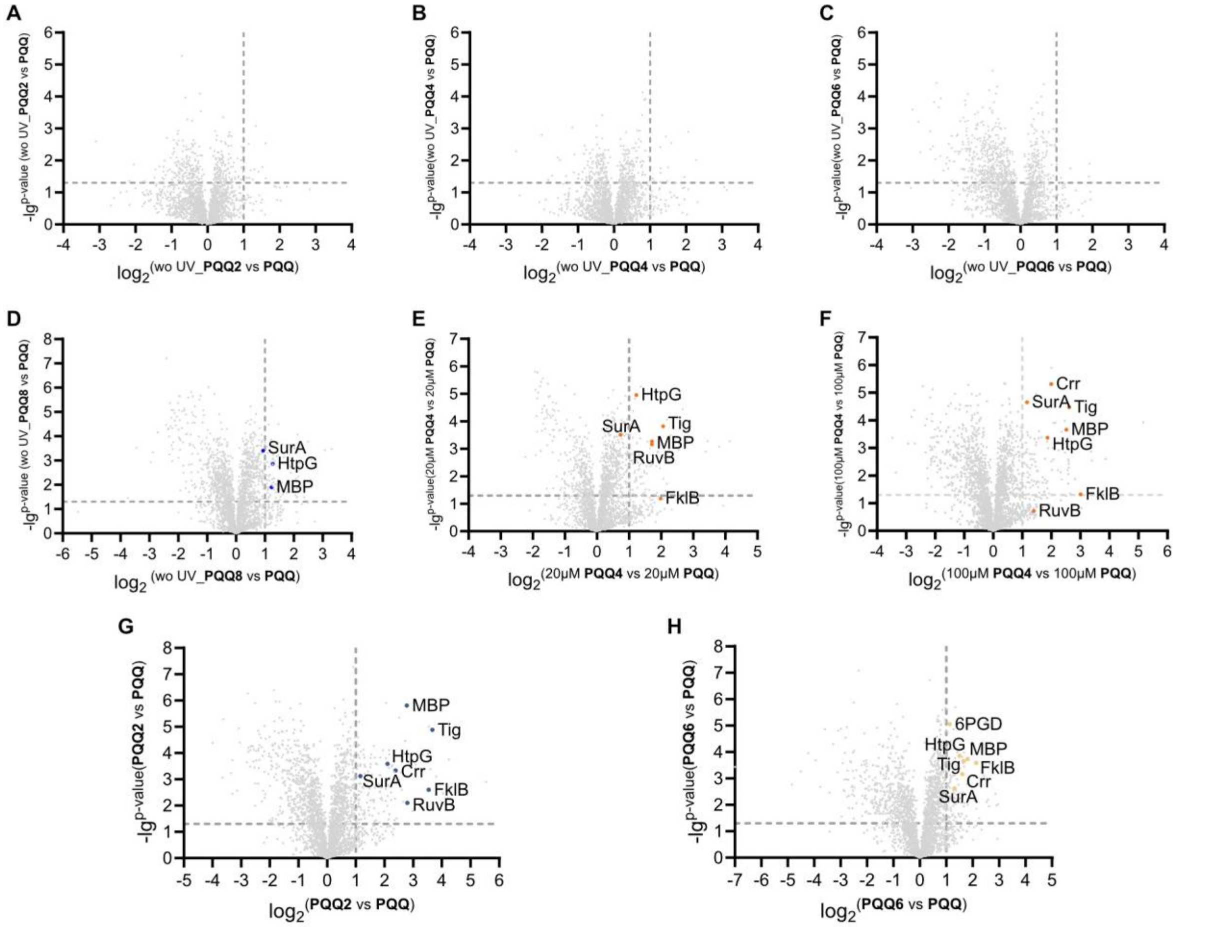

Figure S8. Enrichment of target proteins in *E. coli* K-12 labeled by different **PQQ** probes with +/- UV treatment via proteomics. (A) Volcano plot of *E coli* K-12 treated with 50 µM **PQQ2** without UV irradiation compared to 50 µM **PQQ**. The experiment was conducted in 3 biological replicates. The vertical and horizontal dashed lines represent a log_2-fold change of 1 and a -log_{10} p-value of 1.3, respectively. (B) Volcano plot of *E coli* K-12 treated with 50 µM **PQQ4** without UV irradiation compared to 50 µM **PQQ**. The experimental details and cutoff criteria for volcano plot are as in Figure S8A. (C) Volcano plot of *E coli* K-12 treated with 50 µM **PQQ6** without UV irradiation compared to 50 µM **PQQ**. The experimental details and cutoff criteria for volcano plot are as in Figure S8A. (D) Volcano plot of *E coli* K-12 treated with 50 µM **PQQ8** without UV irradiation compared to 50 µM **PQQ**. The experimental details and cutoff criteria for volcano plot are as in Figure S8A. (E) Volcano plot of *E coli* K-12 treated with 20 µM **PQQ4** with UV irradiation compared to 20 µM **PQQ**. The experimental details and cutoff criteria for volcano plot are as in Figure S8A. (F) Volcano plot of *E coli* K-12 treated with 100 µM **PQQ4** with UV irradiation compared to 100 µM **PQQ**. The experimental details and cutoff criteria for volcano plot are as in Figure S8A. (G) Volcano plot of *E coli* K-12 treated with 50 µM **PQQ2** with UV irradiation compared to 50 µM **PQQ**. The experimental details and cutoff criteria for volcano plot are as in Figure S8A. (H) Volcano plot of *E coli* K-12 treated with 50 µM **PQQ6** with UV irradiation compared to 50 µM **PQQ**. The experimental details and cutoff criteria for volcano plot are as in Figure S8A.

Table S14. Representative protein hits enriched by PQQ8 in *E. coli* K-12 without UV treatment

Genes	UniProt ID	Protein	Entry	Fold Change	-$lg^{p\text{-}value}$	q-value	t-value
surA	P0ABZ6	SurA	wo UV_**PQQ8** vs **PQQ**	0.94	3.40	0	10.94
htpG	P0A6Z3	HtpG	wo UV_**PQQ8** vs **PQQ**	1.27	2.87	0.003	7.94
malE	P0AEX9	MBP	wo UV_**PQQ8** vs **PQQ**	1.23	1.90	0.010	4.31

Table S15. Representative protein hits enriched by PQQ probes in *E. coli* K-12 with UV treatment

Genes	UniProt ID	Protein	Entry	Fold Change	-lg$^{p\text{-value}}$	q-value	t-value
surA	P0ABZ6	SurA	**PQQ2** vs **PQQ**	1.16	3.12	0.001	9.28
htpG	P0A6Z3	HtpG	**PQQ2** vs **PQQ**	2.11	3.59	0.001	12.22
malE	P0AEX9	MBP	**PQQ2** vs **PQQ**	2.78	5.81	0	44.28
tig	P0A850	Tig	**PQQ2** vs **PQQ**	3.67	4.88	0	25.97
crr	P69783	Crr	**PQQ2** vs **PQQ**	2.39	3.34	0.001	10.52
fklB	P0A9L3	FklB	**PQQ2** vs **PQQ**	3.54	2.61	0.003	6.78
ruvB	P0A812	RuvB	**PQQ2** vs **PQQ**	2.79	2.10	0.005	4.92
surA	P0ABZ6	SurA	20 µM **PQQ4** vs 20 µM **PQQ**	0.73	3.51	0.003	11.65
htpG	P0A6Z3	HtpG	20 µM **PQQ4** vs 20 µM **PQQ**	1.23	4.96	0	27.08
malE	P0AEX9	MBP	20 µM **PQQ4** vs 20 µM **PQQ**	1.71	3.27	0.004	10.12
tig	P0A850	Tig	20 µM **PQQ4** vs 20 µM **PQQ**	2.06	3.81	0.004	13.97
fklB	P0A9L3	FklB	20 µM **PQQ4** vs 20 µM **PQQ**	1.98	1.19	0.086	2.53
ruvB	P0A812	RuvB	20 µM **PQQ4** vs 20 µM **PQQ**	1.71	3.16	0.005	9.48
surA	P0ABZ6	SurA	50 µM **PQQ4** vs 50 µM **PQQ**	1.16	2.77	0.004	7.51
htpG	P0A6Z3	HtpG	50 µM **PQQ4** vs 50 µM **PQQ**	1.68	3.14	0.003	9.37
malE	P0AEX9	MBP	50 µM **PQQ4** vs 50 µM **PQQ**	2.33	4.41	0.007	19.74
tig	P0A850	Tig	50 µM **PQQ4** vs 50 µM **PQQ**	2.32	3.98	0.003	15.33
crr	P69783	Crr	50 µM **PQQ4** vs 50 µM **PQQ**	1.45	2.46	0.006	6.19
fklB	P0A9L3	FklB	50 µM **PQQ4** vs 50 µM **PQQ**	2.35	1.97	0.015	4.52
ruvB	P0A812	RuvB	50 µM **PQQ4** vs 50 µM **PQQ**	1.67	1.37	0.034	2.95
surA	P0ABZ6	SurA	100 µM **PQQ4** vs 100 µM **PQQ**	1.16	4.65	0	22.67
htpG	P0A6Z3	HtpG	100 µM **PQQ4** vs 100 µM **PQQ**	1.87	3.37	0.003	10.74
malE	P0AEX9	MBP	100 µM **PQQ4** vs 100 µM **PQQ**	2.52	3.66	0.003	12.75
tig	P0A850	Tig	100 µM **PQQ4** vs 100 µM **PQQ**	2.61	4.47	0.004	20.47
crr	P69783	Crr	100 µM **PQQ4** vs 100 µM **PQQ**	2.00	5.31	0	33.30

fklB	P0A9L3	FklB	100 µM **PQQ4** vs 100 µM **PQQ**	3.01	1.32	0.046	2.83
ruvB	P0A812	RuvB	100 µM **PQQ4** vs 100 µM **PQQ**	1.39	0.71	0.195	1.56
surA	P0ABZ6	SurA	**PQQ6** vs **PQQ**	1.31	2.62	0.003	6.84
htpG	P0A6Z3	HtpG	**PQQ6** vs **PQQ**	1.50	3.86	0	14.32
malE	P0AEX9	MBP	**PQQ6** vs **PQQ**	1.81	3.74	0	13.35
tig	P0A850	Tig	**PQQ6** vs **PQQ**	1.66	3.65	0	12.66
crr	P69783	Crr	**PQQ6** vs **PQQ**	1.61	3.16	0.001	9.49
fklB	P0A9L3	FklB	**PQQ6** vs **PQQ**	2.14	3.59	0	12.24
gnd	P00350	6PGD	**PQQ6** vs **PQQ**	1.13	5.05	0	28.65
surA	P0ABZ6	SurA	**PQQ8** vs **PQQ**	1.36	3.61	0	12.35
htpG	P0A6Z3	HtpG	**PQQ8** vs **PQQ**	2.06	3.61	0	12.34
malE	P0AEX9	MBP	**PQQ8** vs **PQQ**	2.33	5.40	0	34.91
tig	P0A850	Tig	**PQQ8** vs **PQQ**	2.28	4.11	0	16.61
crr	P69783	Crr	**PQQ8** vs **PQQ**	2.18	3.28	0	10.19
fklB	P0A9L3	FklB	**PQQ8** vs **PQQ**	2.58	2.11	0.004	4.95
ruvB	P0A812	RuvB	**PQQ8** vs **PQQ**	1.56	1.27	0.031	2.70

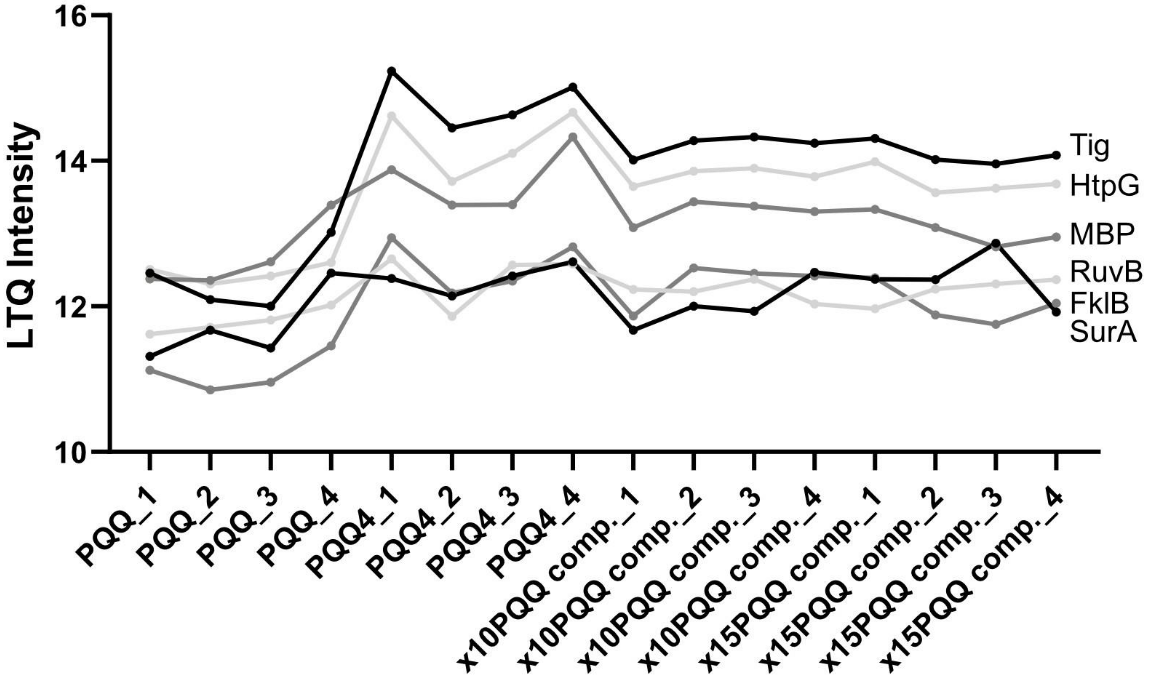

Figure S9. Profile plot of selected hit proteins in *E. coli* K-12 competition experiment. *E. coli* K-12 was in situ labeled by 50 µM **PQQ4** as well as competition with **PQQ** (50 µM **PQQ4** + 500 µM **PQQ**, and 50 µM **PQQ4** + 750 µM **PQQ**) compared to **PQQ**. The experiment was conducted in 4 biological replicates. The dots and solid lines represent the LFQ intensity of selected hit proteins (Tig, HtpG, MBP, RuvB, FklB and SurA) labeled by 50 µM **PQQ4**.

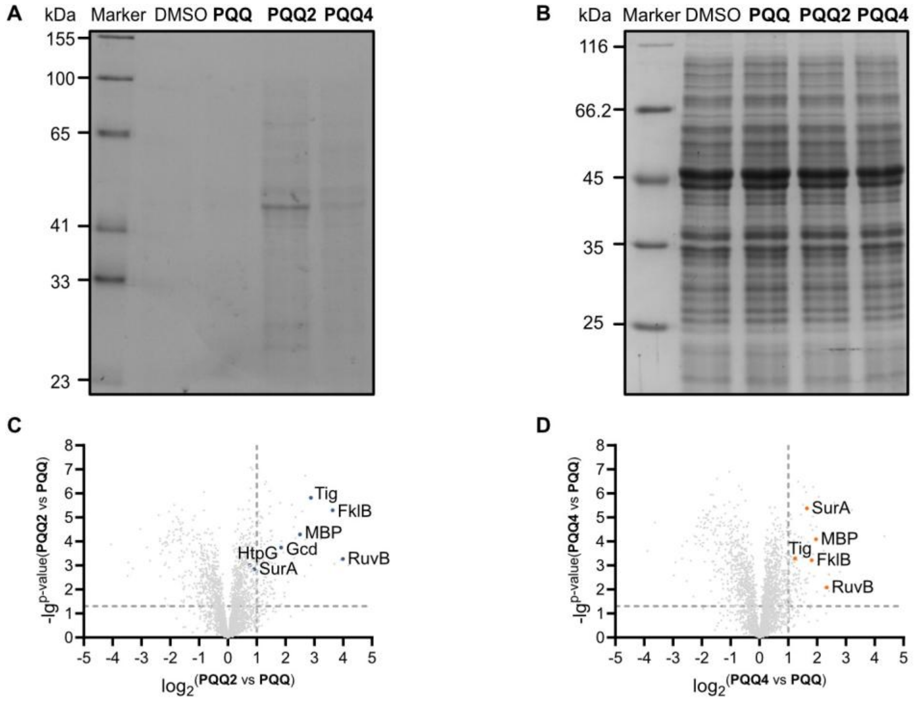

Figure S10. Target identification by chemical proteomics in *E. coli* K-12 cells without starvation. (A) Fluorescent SDS-PAGE of *E. coli* K-12 in situ labeled by 50 µM **PQQ** probes compared to DMSO and 50 µM **PQQ**. (B) Coomassie SDS-PAGE of *E. coli* K-12 in situ labeled by 50 µM **PQQ** probes compared to DMSO and 50 µM **PQQ**. (C) Volcano plot of *E coli* K-12 without starvation treated with 50 µM **PQQ2** compared to 50 µM **PQQ**. The experiment was conducted in 4 biological replicates. The vertical and horizontal dashed lines represent a log_2-fold change of 1 and a $-log_{10}$ p-value of 1.3, respectively. (D) Volcano plot of *E coli* K-12 without starvation treated with 50 µM **PQQ4** compared to 50 µM **PQQ**. The experimental details and cutoff criteria for volcano plot are as in Figure S10C.

Table S16. Representative protein hits enriched by PQQ2 and PQQ4 in *E. coli* K-12 without starvation condition

Genes	UniProt ID	Protein	Entry	Fold Change	$-lg^{p\text{-value}}$	q-value	t-value
surA	P0ABZ6	SurA	**PQQ2** vs **PQQ**	0.91	2.84	0.002	5.56
htpG	P0A6Z3	HtpG	**PQQ2** vs **PQQ**	0.76	3.00	0.002	5.99
malE	P0AEX9	MBP	**PQQ2** vs **PQQ**	2.50	4.29	0	10.22
tig	P0A850	Tig	**PQQ2** vs **PQQ**	2.88	5.82	0	18.67
fklB	P0A9L3	FklB	**PQQ2** vs **PQQ**	3.63	5.29	0	15.22
ruvB	P0A812	RuvB	**PQQ2** vs **PQQ**	3.99	3.27	0.001	6.70
gcd	P15877	Gcd	**PQQ2** vs **PQQ**	1.84	3.74	0.001	8.17
surA	P0ABZ6	SurA	**PQQ4** vs **PQQ**	1.65	5.38	0	15.72
malE	P0AEX9	MBP	**PQQ4** vs **PQQ**	1.97	4.09	0	9.41
tig	P0A850	Tig	**PQQ4** vs **PQQ**	1.24	3.29	0	6.75
fklB	P0A9L3	FklB	**PQQ4** vs **PQQ**	1.81	3.22	0	6.54
ruvB	P0A812	RuvB	**PQQ4** vs **PQQ**	2.34	2.08	0.005	3.87

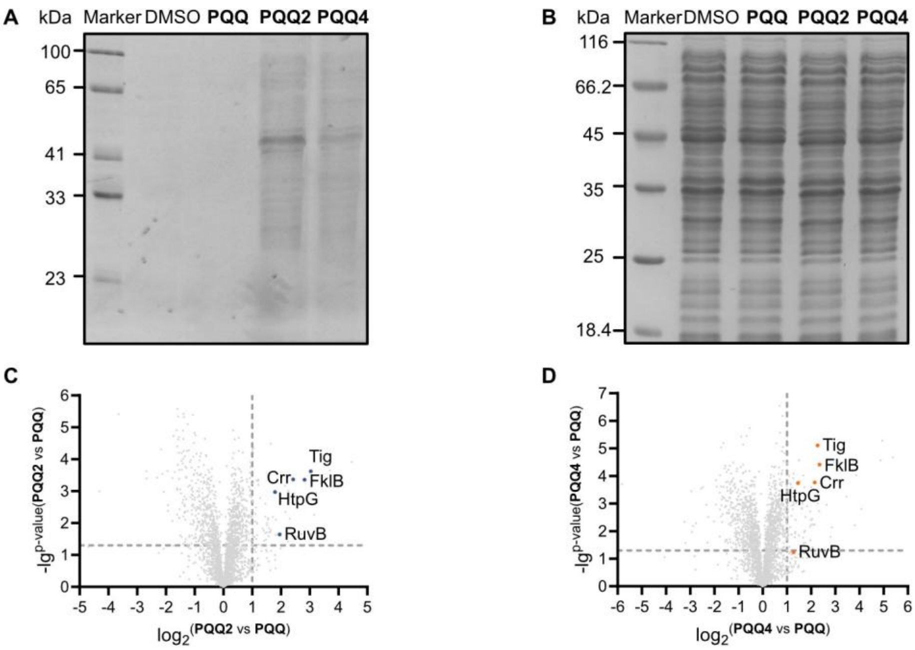

Figure S11. Target identification by chemical proteomics in *E. coli* K-12 cells grown in minimal medium. (A) Fluorescent SDS-PAGE of *E. coli* K-12 in situ labeled by 50 µM **PQQ** probes in minimal medium compared to DMSO and 50 µM **PQQ**. (B) Coomassie SDS-PAGE of *E. coli* K-12 in situ labeled by 50 µM **PQQ** probes in minimal medium compared to DMSO and 50 µM **PQQ**. (C) Volcano plot of *E coli* K-12 in minimal medium treated with 50 µM **PQQ2** compared to 50 µM **PQQ**. The experiment was conducted in 4 biological replicates. The vertical and horizontal dashed lines represent a $\log_2$-fold change of 1 and a -$\log_{10}$ p-value of 1.3, respectively. (D) Volcano plot of *E coli* K-12 in minimal medium treated with 50 µM **PQQ4** compared to 50 µM **PQQ**. The experimental details and cutoff criteria for volcano plot are as in Figure S11C.

Table S17. Representative protein hits enriched by PQQ2 and PQQ4 in *E. coli* K-12 grown in minimal medium

Genes	UniProt ID	Protein	Entry	Fold Change	-$lg^{p\text{-value}}$	q-value	t-value
htpG	P0A6Z3	HtpG	**PQQ2** vs **PQQ**	1.79	2.97	0.006	8.47
tig	P0A850	Tig	**PQQ2** vs **PQQ**	3.03	3.62	0.003	12.47
fklB	P0A9L3	FklB	**PQQ2** vs **PQQ**	2.82	3.36	0.005	10.65
ruvB	P0A812	RuvB	**PQQ2** vs **PQQ**	1.95	1.64	0.032	3.60
crr	P69783	Crr	**PQQ2** vs **PQQ**	2.43	3.37	0.005	10.73
htpG	P0A6Z3	HtpG	**PQQ4** vs **PQQ**	1.45	3.74	0.002	13.35
tig	P0A850	Tig	**PQQ4** vs **PQQ**	2.27	5.11	0	29.58
fklB	P0A9L3	FklB	**PQQ4** vs **PQQ**	2.35	4.42	0.005	19.79
ruvB	P0A812	RuvB	**PQQ4** vs **PQQ**	1.27	1.24	0.039	2.63
crr	P69783	Crr	**PQQ4** vs **PQQ**	2.15	3.76	0.002	13.55

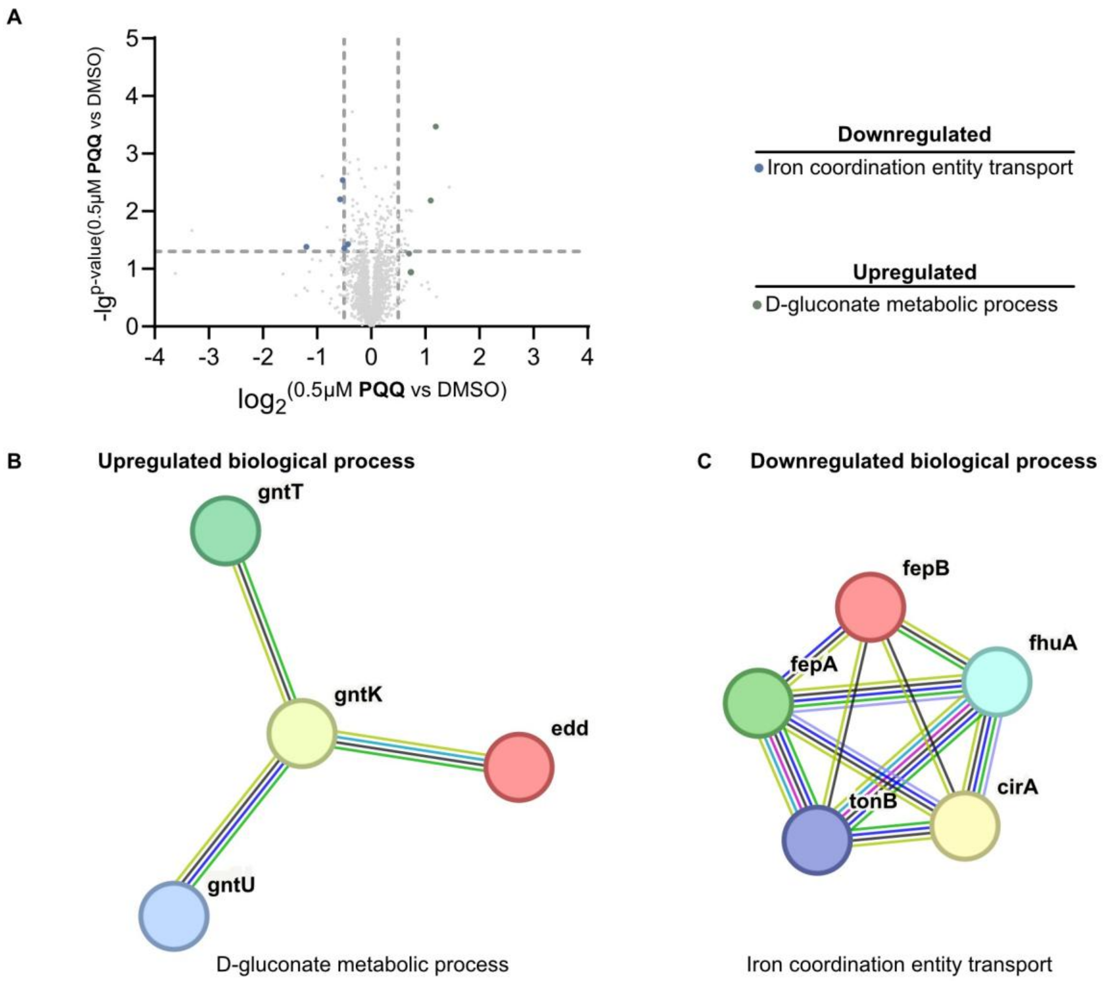

Figure S12. Full proteome analysis of *E. coli* K-12 treated by 0.5 μM **PQQ** compared to DMSO. (A) Volcano plot of *E. coli* K-12 treated with 0.5 μM **PQQ** compared to DMSO. The experiment was conducted in 4 biological replicates. The vertical and horizontal dashed lines represent a log_2-fold change of 1 and a -log_{10} p-value of 1.3, respectively. Colored dots (log_2-fold change > 0.5 or < -0.5, p-value < 0.05, t-value > 2 or < -2) show functional upregulated and downregulated proteins. (B) Upregulated biological process analyzed with String 12.0 database with “Group Similarity” > = 0.8 and high confidence > = 0.7. (C) Downregulated biological process analyzed with String database with “Group Similarity” > = 0.8 and high confidence > = 0.7.

Table S18. Upregulated protein hits in *E. coli* K-12 treated with 0.5 μM PQQ vs DMSO

Genes	UniProt ID	Protein	Entry	Fold Change	-lg$^{p\text{-value}}$	q-value	t-value
edd	P0ADF6	EDD	0.5 μM_**PQQ** vs DMSO	1.19	3.47	0.224	7.28
gntK	P46859	GntK	0.5 μM_**PQQ** vs DMSO	1.10	2.19	0.529	4.08
gntT	P39835	GntT	0.5 μM_**PQQ** vs DMSO	0.70	1.26	0.702	2.38
gntU	P0AC96	GntU	0.5 μM_**PQQ** vs DMSO	0.70	0.98	0.705	1.89

Table S19. Downregulated protein hits in *E. coli* K-12 treated with 0.5 μM PQQ vs DMSO

Genes	UniProt ID	Protein	Entry	Fold Change	-lg$^{p\text{-value}}$	q-value	t-value
fepA	P05825	FepA	0.5 μM_**PQQ** vs DMSO	-0.50	1.36	0.740	-2.54
fepB	P0AEL6	FepB	0.5 μM_**PQQ** vs DMSO	-0.43	1.43	0.718	-2.66
fhuA	P06971	FhuA	0.5 μM_**PQQ** vs DMSO	-0.53	2.54	0.454	-4.84
cirA	P17315	CirA	0.5 μM_**PQQ** vs DMSO	-0.57	2.21	0.579	-4.12
tonB	P02929	TonB	0.5 μM_**PQQ** vs DMSO	-1.20	1.38	0.731	-2.58

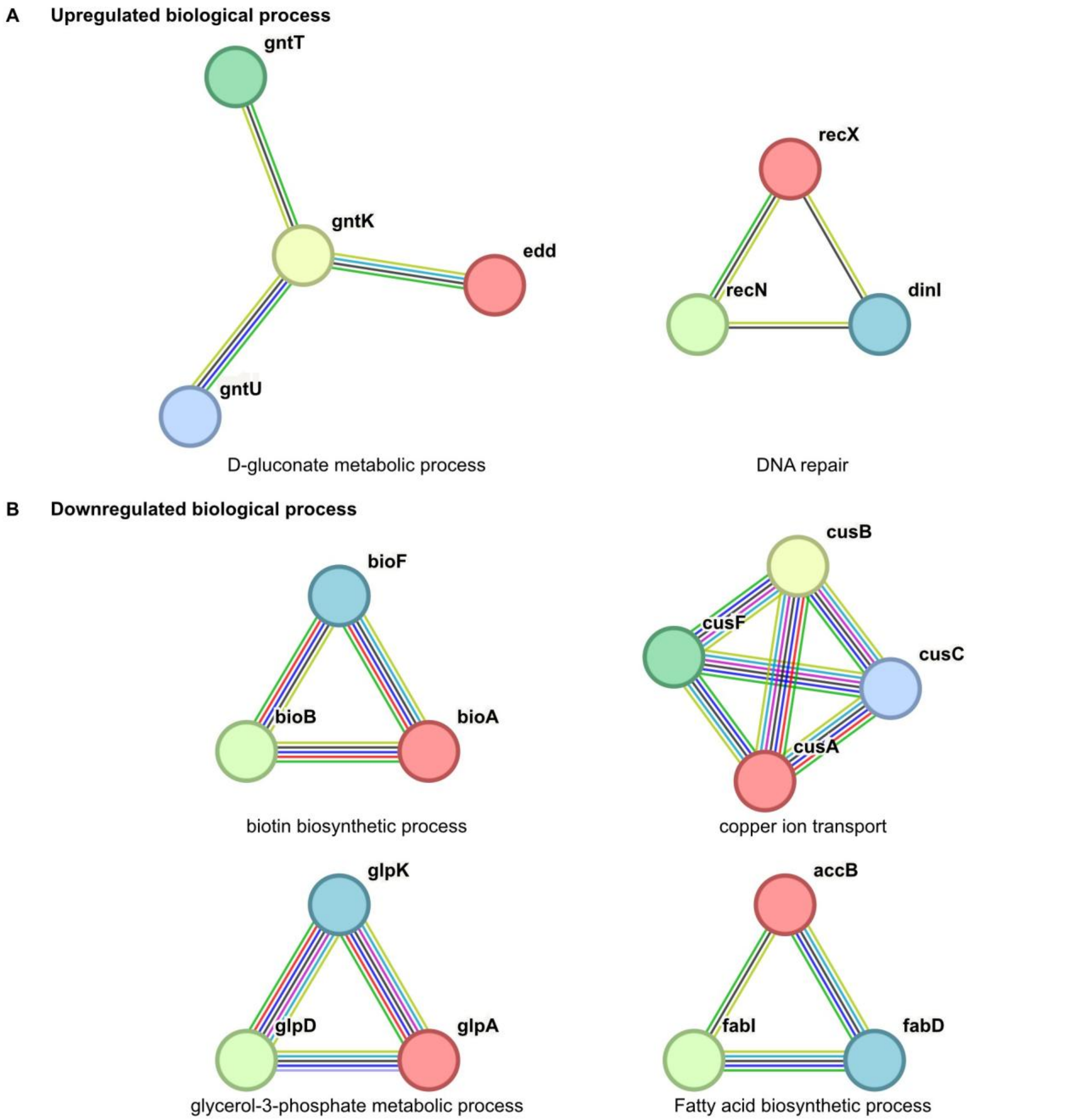

Figure S13. Full proteome analysis of *E. coli* K-12 treated by 50 µM **PQQ** compared to DMSO. (A) Upregulated biological process analyzed with String database with "Group Similarity" > = 0.8 and high confidence > = 0.7. (B) Downregulated biological process analyzed with String 12.0 database with "Group Similarity" > = 0.8 and high confidence > = 0.7.

Table S20. Upregulated protein hits in *E. coli* K-12 treated with 50 µM PQQ vs DMSO

**Genes**	**UniProt ID**	**Protein**	**Entry**	**Fold Change**	**-lg$^{p\text{-}value}$**	**q-value**	**t-value**
edd	P0ADF6	EDD	50 µM_**PQQ** vs DMSO	1.25	4.07	0.022	9.35
gntK	P46859	GntK	50 µM_**PQQ** vs DMSO	1.46	2.22	0.090	4.15
gntT	P39835	GntT	50 µM_**PQQ** vs DMSO	0.86	1.37	0.183	2.57
gntU	P0AC96	GntU	50 µM_**PQQ** vs DMSO	1.10	1.70	0.134	3.15
recX	P33596	RecX	50 µM_**PQQ** vs DMSO	1.11	1.81	0.127	3.34
recN	P05824	RecN	50 µM_**PQQ** vs DMSO	1.07	3.59	0.025	7.68
dinI	P0ABR1	DinI	50 µM_**PQQ** vs DMSO	1.09	4.14	0.028	9.61

Table S21. Downregulated protein hits in *E. coli* K-12 treated with 50 μM PQQ vs DMSO

Genes	UniProt ID	Protein	Entry	Fold Change	$-lg^{p\text{-}value}$	q-value	t-value
bioA	P12995	BioA	50 μM_**PQQ** vs DMSO	-0.57	1.54	0.151	-2.85
bioB	P12996	BioB	50 μM_**PQQ** vs DMSO	-0.50	3.56	0.021	-7.58
bioF	P12998	BioF	50 μM_**PQQ** vs DMSO	-0.57	1.65	0.136	-3.06
cusA	P38054	CusA	50 μM_**PQQ** vs DMSO	-0.61	2.17	0.090	-4.04
cusB	P77239	CusB	50 μM_**PQQ** vs DMSO	-0.79	1.82	0.128	-3.37
cusC	P77211	CusC	50 μM_**PQQ** vs DMSO	-0.57	1.29	0.194	-2.43
cusF	P77214	CusF	50 μM_**PQQ** vs DMSO	-0.68	1.97	0.114	-3.64
glpA	P0A9C0	GlpA	50 μM_**PQQ** vs DMSO	-0.74	1.27	0.200	-2.40
glpD	P13035	GlpD	50 μM_**PQQ** vs DMSO	-0.59	2.24	0.089	-4.20
glpK	P0A6F3	GlpK	50 μM_**PQQ** vs DMSO	-0.61	2.66	0.054	-5.11
accB	P0ABD8	BCCP	50 μM_**PQQ** vs DMSO	-0.50	1.79	0.127	-3.31
fabI	P0AEK4	FabI	50 μM_**PQQ** vs DMSO	-0.51	1.44	0.167	-2.69
fabD	P0AAI9	FabD	50 μM_**PQQ** vs DMSO	-0.39	2.47	0.059	-4.69

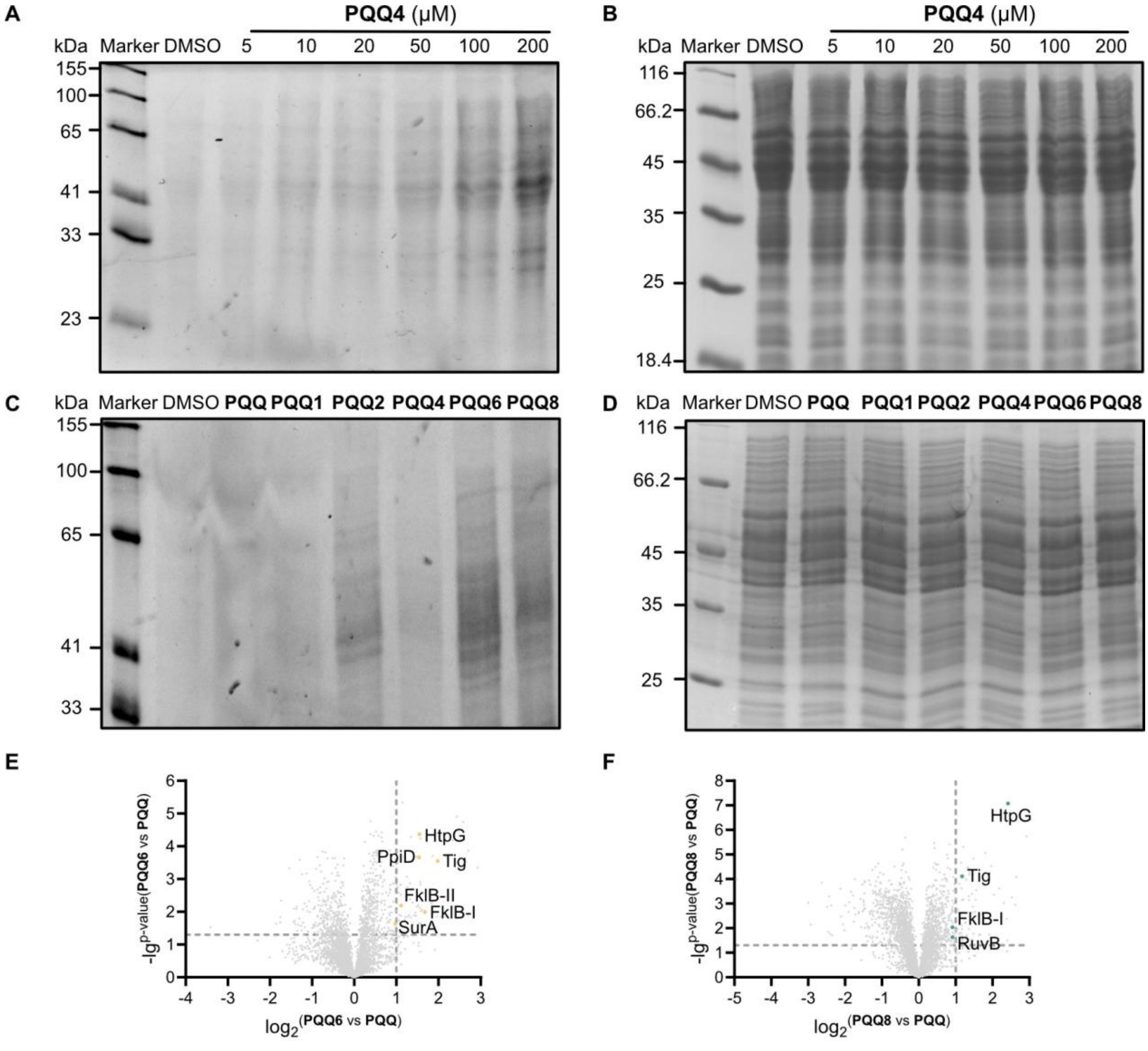

Figure S14. Target identification by chemical proteomics in *P. putida* KT2440 cells. (A) Fluorescent SDS-PAGE of *P. putida* KT2440 in situ labeled by different concentrations of **PQQ4** compared to DMSO. (B) Coomassie SDS-PAGE of *P. putida* KT2440 in situ labeled by different concentrations of **PQQ4** compared to DMSO. (C) Fluorescent SDS-PAGE of *P. putida* KT2440 in situ labeled by different **PQQ** probes compared to DMSO and 50 µM **PQQ**. (D) Coomassie SDS-PAGE of *P. putida* KT2440 in situ labeled by different **PQQ** probes compared to DMSO and 50 µM **PQQ**. (E) Volcano plot of *P. putida* KT2440 treated with 50 µM **PQQ6** compared to 50 µM **PQQ**. The experiment was conducted in 4 biological replicates. The vertical and horizontal dashed lines represent a log_2-fold change of 1 and a -log_{10} p-value of 1.3, respectively. (F) Volcano plot of *P. putida* KT2440 treated with 50 µM **PQQ8** compared to 50 µM **PQQ**. The experimental details and cutoff criteria for volcano plot are as in Figure S14E.

Table S22. Representative protein hits enriched by PQQ probes in *P. putida* KT2440.

Genes	UniProt ID	Protein	Entry	Fold Change	-lg$^{p\text{-value}}$	q-value	t-value
surA	Q88QT4	SurA	**PQQ2** vs **PQQ**	3.41	4.27	0	18.16
htpG	Q88FB9	HtpG	**PQQ2** vs **PQQ**	3.55	4.15	0	16.92
tig	Q88KJ1	Tig	**PQQ2** vs **PQQ**	3.92	3.57	0.002	12.06
fklB-I	Q88Q14	FklB-I	**PQQ2** vs **PQQ**	3.34	1.92	0.018	4.35
fklB-II	Q88M60	FklB-II	**PQQ2** vs **PQQ**	3.24	4.07	0	16.15
ruvB	Q88NJ0	RuvB	**PQQ2** vs **PQQ**	1.63	1.23	0.055	2.61
PP_2304	Q88KI6	PpiD	**PQQ2** vs **PQQ**	3.38	4.50	0	20.83
bamB	Q88PJ4	BamB	**PQQ2** vs **PQQ**	2.04	4.51	0	20.97
pqqC	Q88QV6	PqqC	**PQQ2** vs **PQQ**	0.92	3.49	0.003	11.51
surA	Q88QT4	SurA	**PQQ4** vs **PQQ**	2.71	3.27	0.008	10.12
htpG	Q88FB9	HtpG	**PQQ4** vs **PQQ**	2.71	3.52	0.012	11.73
tig	Q88KJ1	Tig	**PQQ4** vs **PQQ**	2.88	3.01	0.008	8.67
fklB-I	Q88Q14	FklB-I	**PQQ4** vs **PQQ**	2.53	1.53	0.065	3.30
fklB-II	Q88M60	FklB-II	**PQQ4** vs **PQQ**	2.21	3.13	0.008	9.33
ruvB	Q88NJ0	RuvB	**PQQ4** vs **PQQ**	1.16	0.87	0.191	1.87
PP_2304	Q88KI6	PpiD	**PQQ4** vs **PQQ**	2.22	3.22	0.007	9.84
bamB	Q88PJ4	BamB	**PQQ4** vs **PQQ**	0.77	1.18	0.113	2.51
surA	Q88QT4	SurA	**PQQ6** vs **PQQ**	1.29	2.48	0.033	6.28
htpG	Q88FB9	HtpG	**PQQ6** vs **PQQ**	1.56	2.79	0.029	7.56
tig	Q88KJ1	Tig	**PQQ6** vs **PQQ**	1.83	2.35	0.034	5.77
fklB-I	Q88Q14	FklB-I	**PQQ6** vs **PQQ**	1.67	1.04	0.188	2.20
fklB-II	Q88M60	FklB-II	**PQQ6** vs **PQQ**	1.36	2.49	0.034	6.31
PP_2304	Q88KI6	PpiD	**PQQ6** vs **PQQ**	1.60	3.20	0.026	9.71
htpG	Q88FB9	HtpG	**PQQ8** vs **PQQ**	2.42	7.08	0	30.41
tig	Q88KJ1	Tig	**PQQ8** vs **PQQ**	1.18	4.11	0.003	9.50
fklB-I	Q88Q14	FklB-I	**PQQ8** vs **PQQ**	0.92	2.03	0.013	3.77
ruvB	Q88NJ0	RuvB	**PQQ8** vs **PQQ**	0.92	1.63	0.025	3.02

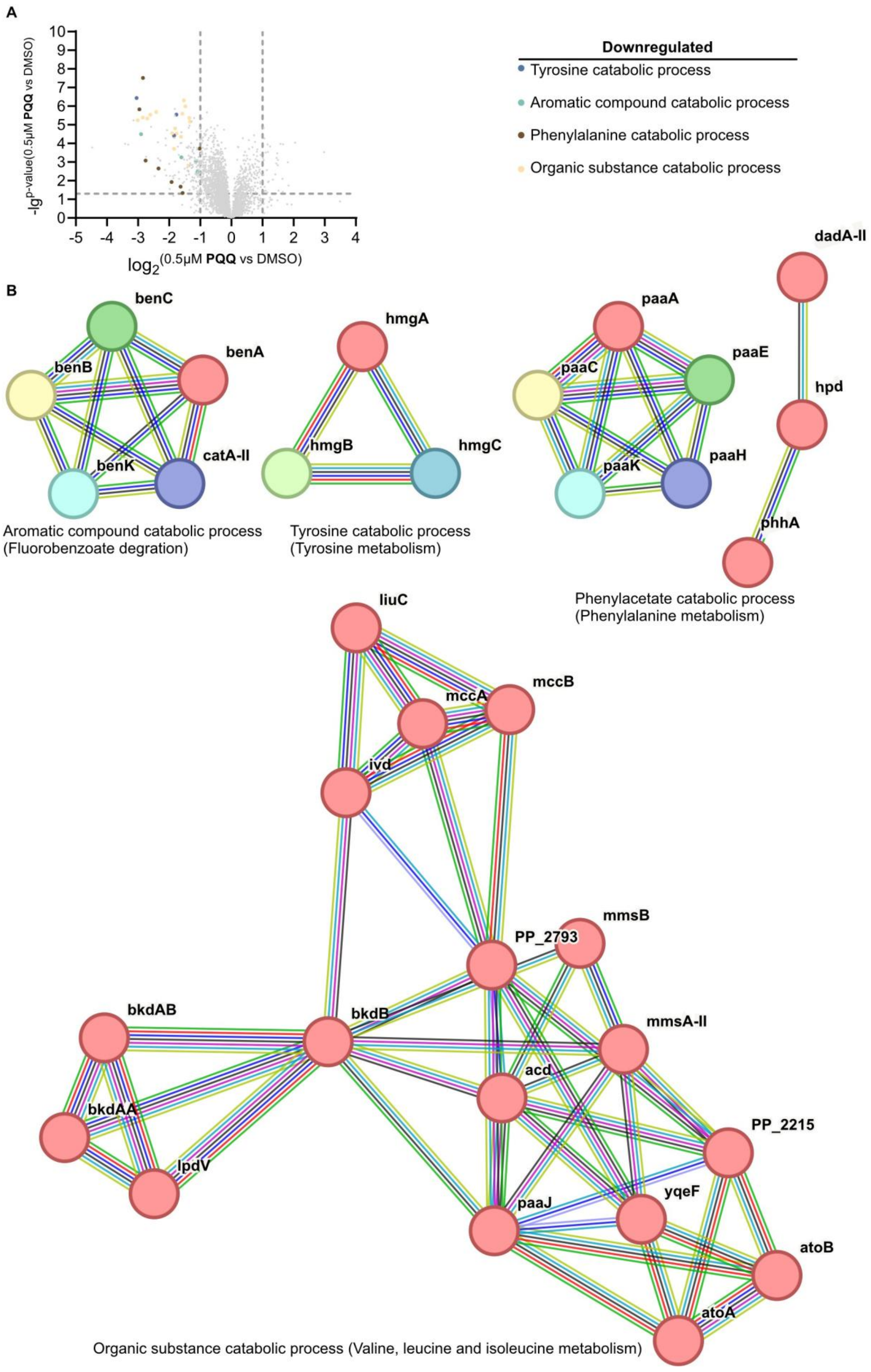
A
-lg p-value(0.5μM PQQ vs DMSO)
log2 (0.5μM PQQ vs DMSO)
Downregulated
Tyrosine catabolic process
Aromatic compound catabolic process
Phenylalanine catabolic process
Organic substance catabolic process
B
benC
benA
benB
benK
catA-II
Aromatic compound catabolic process
(Fluorobenzoate degration)
hmgA
hmgB
hmgC
Tyrosine catabolic process
(Tyrosine metabolism)
paaA
paaE
paaC
paaK
paaH
dadA-II
hpd
phhA
Phenylacetate catabolic process
(Phenylalanine metabolism)
liuC
mccB
mccA
ivd
mmsB
PP_2793
bkdAB
bkdB
mmsA-II
acd
bkdAA
PP_2215
lpdV
paaJ
yqeF
atoB
atoA
Organic substance catabolic process (Valine, leucine and isoleucine metabolism)

Figure S15. Full proteome analysis of *P. putida* KT2440 treated by 0.5 µM **PQQ** compared to DMSO. (A) Volcano plot of *P. putida* KT2440 treated with 0.5 µM **PQQ** at the start of inoculation compared to DMSO. The experiment was conducted in 4 biological replicates. The vertical and horizontal dashed lines represent a log_2-fold change of 1 and a -log_{10} p-value of 1.3, respectively. Colored dots (log_2-fold change < -1, p-value < 0.05, t-value < -2) show functional downregulated proteins. (B) Downregulated biological process analyzed with String 12.0 database with "Group Similarity" > = 0.8 and high confidence > = 0.7.

Table S23. Downregulated protein hits in *P. putida* KT2440 treated with 0.5 µM PQQ vs DMSO.

Genes	UniProt ID	Protein	Entry	Fold Change	-lg$^{p\text{-}value}$	q-value	t-value
benA	Q88I40	BenA	0.5 µM_**PQQ** vs DMSO	-1.07	2.53	0.005	-4.82
benB	Q88I39	BenB	0.5 µM_**PQQ** vs DMSO	-1.14	3.05	0.002	-6.08
benC	Q88I38	BenC	0.5 µM_**PQQ** vs DMSO	-2.90	4.50	0	-11.11
benK	Q88I36	BenK	0.5 µM_**PQQ** vs DMSO	-1.08	2.52	0.005	-4.80
catA-II	Q88I35	CatA2	0.5 µM_**PQQ** vs DMSO	-1.61	3.25	0.001	-6.65
hmgA	Q88E47	HmgA	0.5 µM_**PQQ** vs DMSO	-3.05	6.44	0	-23.74
hmgB	Q88E48	HmgB	0.5 µM_**PQQ** vs DMSO	-1.77	5.55	0	-16.81
hmgC	Q88E49	HmgC	0.5 µM_**PQQ** vs DMSO	-1.84	4.42	0	-10.75
paaA	Q88HS5	PaaA	0.5 µM_**PQQ** vs DMSO	-2.76	3.08	0.002	-6.16
paaC	Q88HS7	PaaC	0.5 µM_**PQQ** vs DMSO	-1.92	1.93	0.020	-3.57
paaE	Q88HS9	PaaE	0.5 µM_**PQQ** vs DMSO	-1.63	1.68	0.033	-3.10
paaK	Q88HS4	PaaK	0.5 µM_**PQQ** vs DMSO	-2.34	2.65	0.004	-5.10
paaH	Q88HS1	PaaH	0.5 µM_**PQQ** vs DMSO	-1.57	1.34	0.063	-2.51
dadA2	Q88CB1	DadA2	0.5 µM_**PQQ** vs DMSO	-1.02	3.72	0	-8.10
hpd	Q88HC7	HpD	0.5 µM_**PQQ** vs DMSO	-2.84	7.51	0	-36.00
phhA	Q88EH3	PhhA	0.5 µM_**PQQ** vs DMSO	-2.96	5.83	0	-18.76
liuC	Q88FM3	LiuC	0.5 µM_**PQQ** vs DMSO	-1.84	3.71	-1.84	-8.06
mccA	Q88FM2	MccA	0.5 µM_**PQQ** vs DMSO	-1.35	5.37	0	-15.70
mccB	Q88FM4	MccB	0.5 µM_**PQQ** vs DMSO	-1.53	6.30	0	-22.54

ivd	Q88FM5	IvD	0.5 µM_**PQQ** vs DMSO	-1.58	5.59	0	-17.11
mmsB	Q88E02	MmsB	0.5 µM_**PQQ** vs DMSO	-1.90	4.55	0	-11.33
mmsA-II	Q88E01	MmsA-II	0.5 µM_**PQQ** vs DMSO	-3.01	5.24	0	-14.92
PP_2793	Q88J56	PP_2793	0.5 µM_**PQQ** vs DMSO	-2.85	5.39	0	-15.78
acd	Q88KS3	AcD	0.5 µM_**PQQ** vs DMSO	-1.48	5.99	0	-19.97
bkdB	Q88EQ0	BkdB	0.5 µM_**PQQ** vs DMSO	-1.76	4.53	0	-11.23
bkdAB	Q88EQ1	BkdAB	0.5 µM_**PQQ** vs DMSO	-1.85	4.35	0	-10.47
bkdAA	Q88EQ2	BkdAA	0.5 µM_**PQQ** vs DMSO	-2.61	5.54	0	-16.75
lpdV	Q88EP9	LpdV	0.5 µM_**PQQ** vs DMSO	-1.62	4.35	0	-10.47
paaJ	Q88HS3	PaaJ	0.5 µM_**PQQ** vs DMSO	-1.37	2.80	0.003	-5.45
yqeF	Q88E32	YqeF	0.5 µM_**PQQ** vs DMSO	-1.81	4.80	0	-12.54
PP_2215	Q88KS4	PP_2215	0.5 µM_**PQQ** vs DMSO	-1.33	5.16	0	-14.44
atoA	Q88I79	AtoA	0.5 µM_**PQQ** vs DMSO	-2.70	5.34	0	-15.50
atoB	Q88I78	AtoB	0.5 µM_**PQQ** vs DMSO	-2.42	5.69	0	-17.74

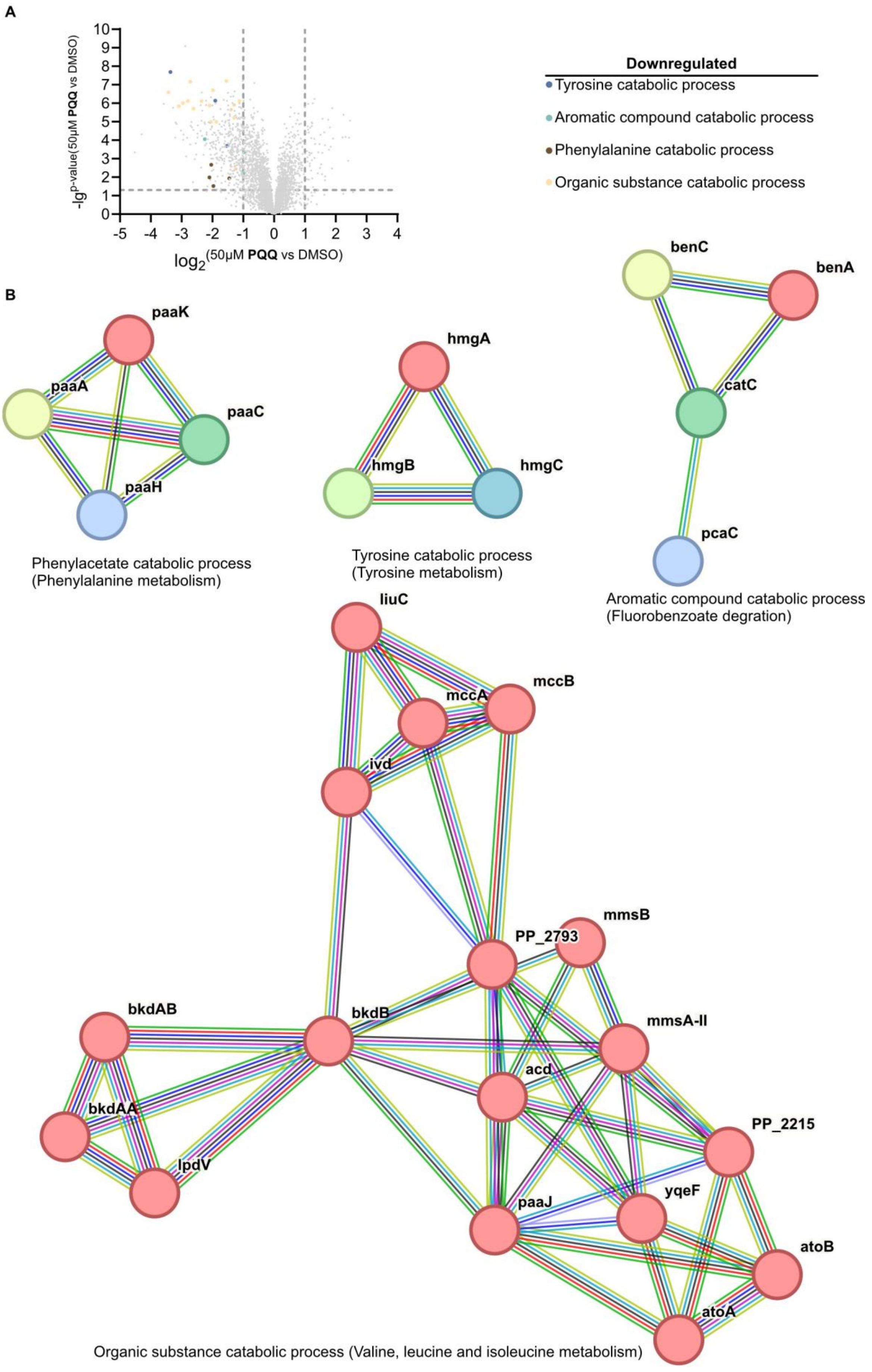
A
-lg p-value(50µM PQQ vs DMSO)
log2(50µM PQQ vs DMSO)
Downregulated
Tyrosine catabolic process
Aromatic compound catabolic process
Phenylalanine catabolic process
Organic substance catabolic process
B
paaK
paaA
paaC
paaH
Phenylacetate catabolic process
(Phenylalanine metabolism)
hmgA
hmgB
hmgC
Tyrosine catabolic process
(Tyrosine metabolism)
benC
benA
catC
pcaC
Aromatic compound catabolic process
(Fluorobenzoate degration)
liuC
mccA
mccB
ivd
PP_2793
mmsB
bkdAB
bkdB
mmsA-II
acd
bkdAA
lpdV
PP_2215
paaJ
yqeF
atoB
atoA
Organic substance catabolic process (Valine, leucine and isoleucine metabolism)

Figure S16. Full proteome analysis of *P. putida* KT2440 treated by 50 µM **PQQ** compared to DMSO. (A) Volcano plot of *P. putida* KT2440 treated with 50 µM **PQQ** at the start of inoculation compared to DMSO. The experiment was conducted in 4 biological replicates. The vertical and horizontal dashed lines represent a log_2-fold change of 1 and a $-log_{10}$ p-value of 1.3, respectively. Colored dots (log_2-fold change < -1, p-value < 0.05, t-value < -2) show functional downregulated proteins. (B) Downregulated biological process analyzed with String 12.0 database with “Group Similarity” > = 0.8 and high confidence > = 0.7.

Table S24. Downregulated protein hits in *P. putida* KT2440 treated with 50 µM PQQ vs DMSO

Genes	UniProt ID	Protein	Entry	Fold Change	$-lg^{p\text{-}value}$	q-value	t-value
benA	Q88I40	BenA	50 µM_**PQQ** vs DMSO	-1.02	2.28	0.006	-4.27
benC	Q88I38	BenC	50 µM_**PQQ** vs DMSO	-2.25	4.05	0	-9.29
catC	Q88GK7	catC	50 µM_**PQQ** vs DMSO	-0.99	2.22	0.007	-4.14
pcaC	Q88HS7	PcaC	50 µM_**PQQ** vs DMSO	-0.96	3.36	0.001	-6.96
hmgA	Q88E47	HmgA	50 µM_**PQQ** vs DMSO	-3.36	7.69	0	-38.52
hmgB	Q88E48	HmgB	50 µM_**PQQ** vs DMSO	-1.91	6.14	0	-21.13
hmgC	Q88E49	HmgC	50 µM_**PQQ** vs DMSO	-1.53	3.69	0	-7.99
paaA	Q88HS5	PaaA	50 µM_**PQQ** vs DMSO	-2.10	1.99	0.012	-3.69
paaC	Q88HS7	PaaC	50 µM_**PQQ** vs DMSO	-1.97	1.52	0.030	-2.83
paaK	Q88HS4	PaaK	50 µM_**PQQ** vs DMSO	-0.51	1.22	0.056	-2.31
paaH	Q88HS1	PaaH	50 µM_**PQQ** vs DMSO	-1.45	1.93	0.013	-3.58
liuC	Q88FM3	LiuC	50 µM_**PQQ** vs DMSO	-2.35	6.11	0	-20.92
mccA	Q88FM2	MccA	50 µM_**PQQ** vs DMSO	-1.30	5.90	0	-19.31
mccB	Q88FM4	MccB	50 µM_**PQQ** vs DMSO	-1.55	7.20	0	-31.90
ivd	Q88FM5	IvD	50 µM_**PQQ** vs DMSO	-1.40	5.64	0	-17.45
mmsB	Q88E02	MmsB	50 µM_**PQQ** vs DMSO	-1.87	4.98	0	-13.47
mmsA-II	Q88E01	MmsA-II	50 µM_**PQQ** vs DMSO	-3.08	5.84	0	-18.84
PP_2793	Q88J56	PP_2793	50 µM_**PQQ** vs DMSO	-2.95	6.00	0	-20.05

acd	Q88KS3	AcD	50 µM_**PQQ** vs DMSO	-1.12	6.12	0	-21.01
bkdB	Q88EQ0	BkdB	50 µM_**PQQ** vs DMSO	-2.05	5.00	0	-13.56
bkdAB	Q88EQ1	BkdAB	50 µM_**PQQ** vs DMSO	-2.80	6.12	0	-21.02
bkdAA	Q88EQ2	BkdAA	50 µM_**PQQ** vs DMSO	-3.43	6.59	0	-25.21
lpdV	Q88EP9	LpdV	50 µM_**PQQ** vs DMSO	-2.09	5.88	0	-19.11
paaJ	Q88HS3	PaaJ	50 µM_**PQQ** vs DMSO	-1.24	2.45	0.005	-4.63
yqeF	Q88E32	YqeF	50 µM_**PQQ** vs DMSO	-1.98	6.70	0	-26.33
PP_2215	Q88KS4	PP_2215	50 µM_**PQQ** vs DMSO	-1.30	5.20	0	-14.69
atoA	Q88I79	AtoA	50 µM_**PQQ** vs DMSO	-2.72	7.16	0	-31.43
atoB	Q88I78	AtoB	50 µM_**PQQ** vs DMSO	-2.61	5.71	0	-17.89

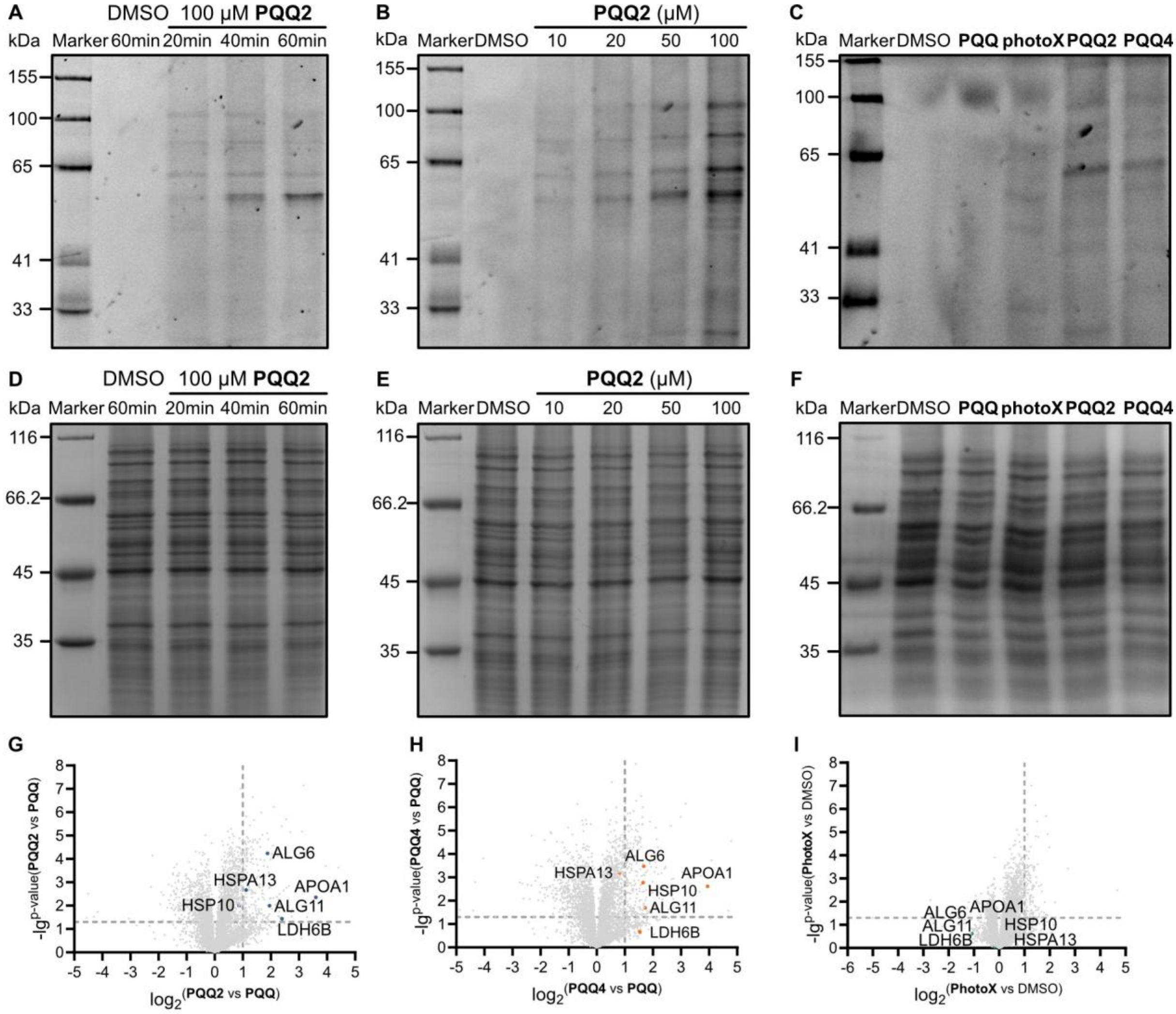

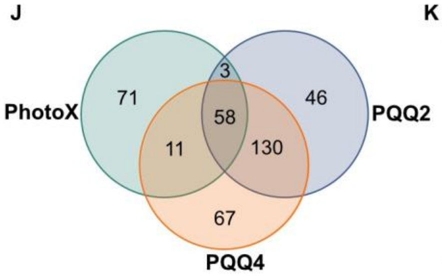

K

Protein	Protein Name	Function	Enriched by
ALG6	Dolichyl pyrophosphate Man9GlcNAc2 alpha-1,3-glucosyltransferase	Protein glycosylation	**PQQ2** and **PQQ4**
ALG11	GDP-Man:Man(3)GlcNAc(2)-PP-Dol alpha-1,2-mannosyltransferase	Protein glycosylation	**PQQ2** and **PQQ4**
APOA1	Apolipoprotein A-I	As cofactor for LCTA	**PQQ2** and **PQQ4**
HSPA13	Heat shock 70 kDa protein 13	ATPase activity	**PQQ2** and **PQQ4**
HSP10	10 kDa heat shock protein, mitochondrial	Protein fold/transport	**PQQ2** and **PQQ4**
LDH6B	L-lactate dehydrogenase A-like 6B	PQQ binding protein	**PQQ2**

LCTA indicates lecithin cholesterol acyltransferase

Figure S17. Target identification by chemical proteomics in HepG2 cells. (A) Fluorescent SDS-PAGE of HepG2 in situ labeled by 100 µM **PQQ2** with different UV irradiation time compared to DMSO. (B) Fluorescent SDS-PAGE of HepG2 in situ labeled by different concentrations of **PQQ2** compared to DMSO. (C) Fluorescent SDS-PAGE of HepG2 in situ labeled by different **PQQ** probes compared to DMSO, **PQQ** and **Photox** (**2-(3-(But-3-yn-1-yl)-3H-diazirin-3-yl)acetic acid**, see the synthesis part). (D) Coomassie SDS-PAGE of HepG2 in situ labeled by 100 µM **PQQ2** with different UV irradiation time compared to DMSO. (E) Coomassie SDS-PAGE of HepG2 in situ labeled by different concentrations of **PQQ2** compared to DMSO. (F) Coomassie SDS-PAGE of HepG2 in situ labeled by different **PQQ** probes compared to DMSO, **PQQ** and **PhotoX**. (G) Volcano plot of HepG2 treated with 50 µM **PQQ2** compared to 50 µM **PQQ**. The experiment was conducted in 4 biological replicates. The vertical and horizontal dashed lines represent a $\log_2$-fold change of 1 and a $-\log_{10}$ p-value of 1.3, respectively. (H) Volcano plot of HepG2 treated with 50 µM **PQQ4** compared to 50 µM **PQQ**. The experimental details and cutoff criteria for volcano plot are as in Figure S17G. (I) Volcano plot of HepG2 treated with 50 µM **PhotoX** compared to DMSO. The experimental details and cutoff criteria for volcano plot are as in Figure S17G. (J) Venn diagram of enriched HepG2 hits by different **PQQ** probes and **PhotoX**. The enriched hits mean the enriched genes in volcano plot with $\log_2$-fold change > 1, p-value < 0.05 and q-value < 0.05. In this experiment, **PhotoX** was used to explore the photo-cross-linker off-target binding proteins

in HepG2. Thus, the real targets are the overlap hits between **PQQ2** and **PQQ4** deducing the off-target hits by **PhotoX**. (K) Table of representative HepG2 proteins found in all AfBPP experiments.

Table S25. Representative protein hits enriched by PQQ2 and PQQ4, and off-targets binding by photoX in HepG2 cells

Genes	UniProt ID	Protein	Entry	Fold Change	-lg$^{p\text{-}}$ value	q-value	t-value
ALG6	Q9Y672	ALG6	**PQQ2** vs **PQQ**	1.88	4.24	0.001	10.01
ALG11	Q2TAA5	ALG11	**PQQ2** vs **PQQ**	1.95	2.00	0.022	3.70
APOA1	P02647	APOA1	**PQQ2** vs **PQQ**	3.60	2.35	0.013	4.43
HSPA13	P48723	HSPA13	**PQQ2** vs **PQQ**	1.12	2.67	0.008	5.14
HSPE1	P61604	HSP10	**PQQ2** vs **PQQ**	0.86	2.00	0.022	3.70
LDHAL6B	Q9BYZ2	LDH6B	**PQQ2** vs **PQQ**	2.37	1.39	0.057	2.61
ALG6	Q9Y672	ALG6	**PQQ4** vs **PQQ**	1.67	3.48	0.001	7.31
ALG11	Q2TAA5	ALG11	**PQQ4** vs **PQQ**	1.73	1.70	0.027	3.14
APOA1	P02647	APOA1	**PQQ4** vs **PQQ**	3.95	2.62	0.006	5.01
HSPA13	P48723	HSPA13	**PQQ4** vs **PQQ**	0.82	3.19	0.002	6.47
HSPE1	P61604	HSP10	**PQQ4** vs **PQQ**	1.65	2.78	0.004	5.40
LDHAL6B	Q9BYZ2	LDH6B	**PQQ4** vs **PQQ**	1.52	0.65	0.241	1.36
ALG6	Q9Y672	ALG6	**photoX** vs **DMSO**	-1.08	0.62	0.332	-1.30
ALG11	Q2TAA5	ALG11	**photoX** vs **DMSO**	-0.15	0.06	0.905	-0.18
APOA1	P02647	APOA1	**photoX** vs **DMSO**	0.74	2.12	0.046	3.94
HSPA13	P48723	HSPA13	**photoX** vs **DMSO**	0.13	0.21	0.715	0.52
HSPE1	P61604	HSP10	**photoX** vs **DMSO**	0.38	0.51	0.404	1.11
LDHAL6B	Q9BYZ2	LDH6B	**photoX** vs **DMSO**	-0.28	0.06	0.915	-0.16

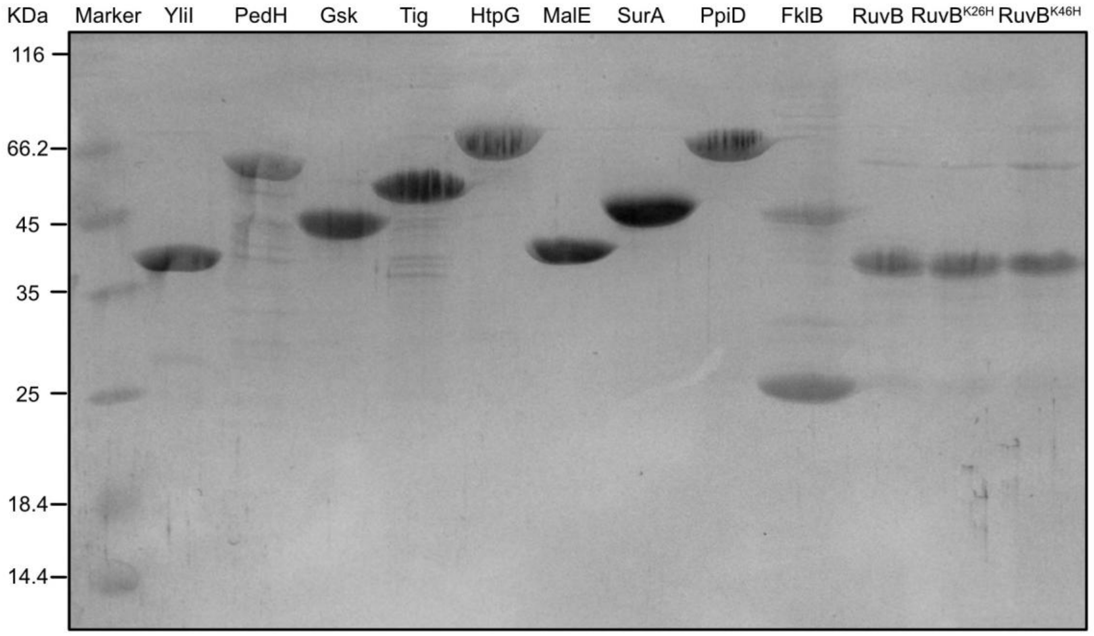

Figure S18. SDS-PAGE of purified recombinant proteins

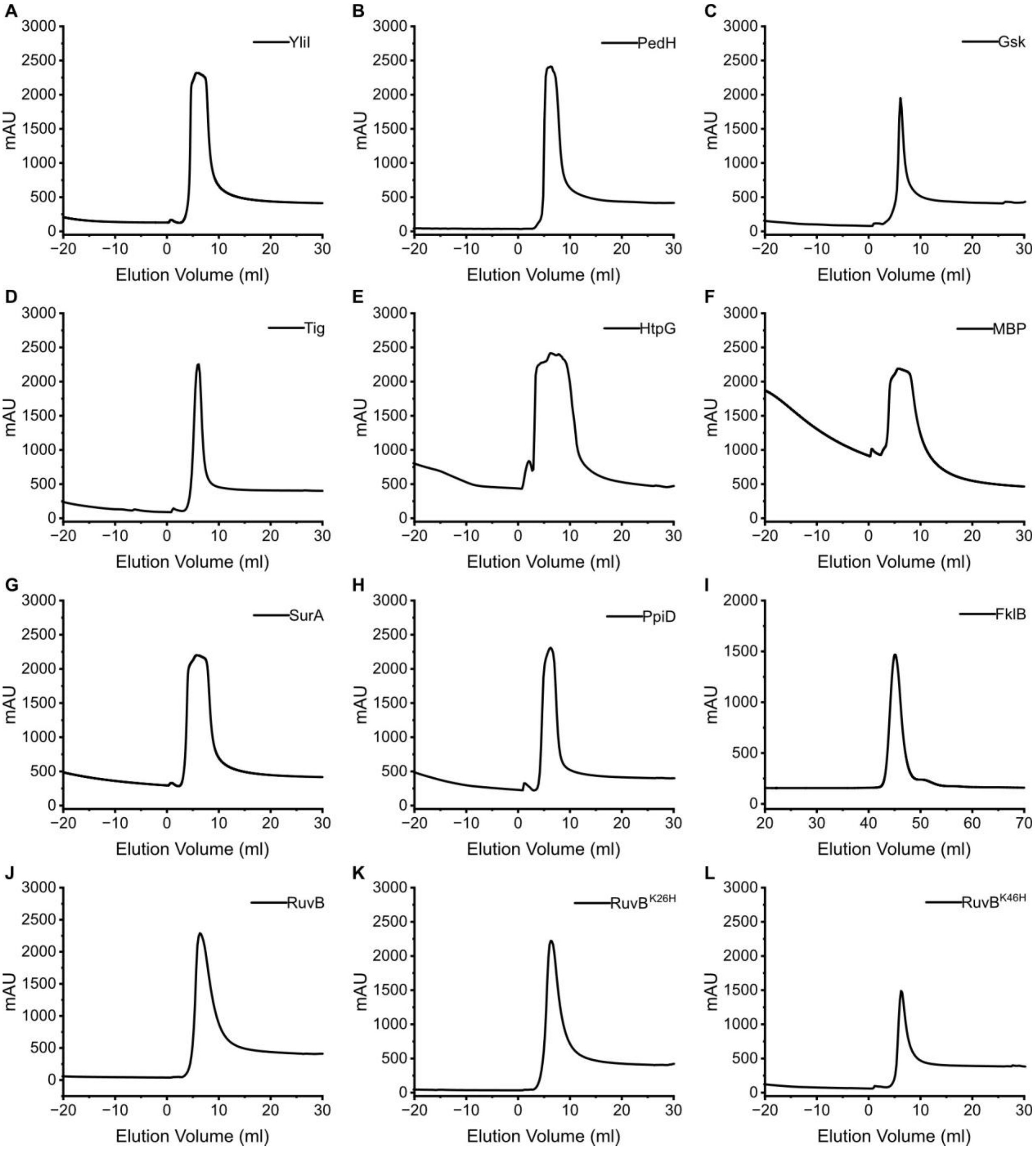

Figure S19. Chromatography of purified recombinant proteins. (A) Chromatography of purified recombinant YliI. (B) PedH. (C) Gsk. (D) Tig. (E) HtpG. (F) MBP. (G) SurA. (H) PpiD. (I) FklB. (J) RuvB. (K) RuvB^{k26H}. (L) RuvB^{K46H}.

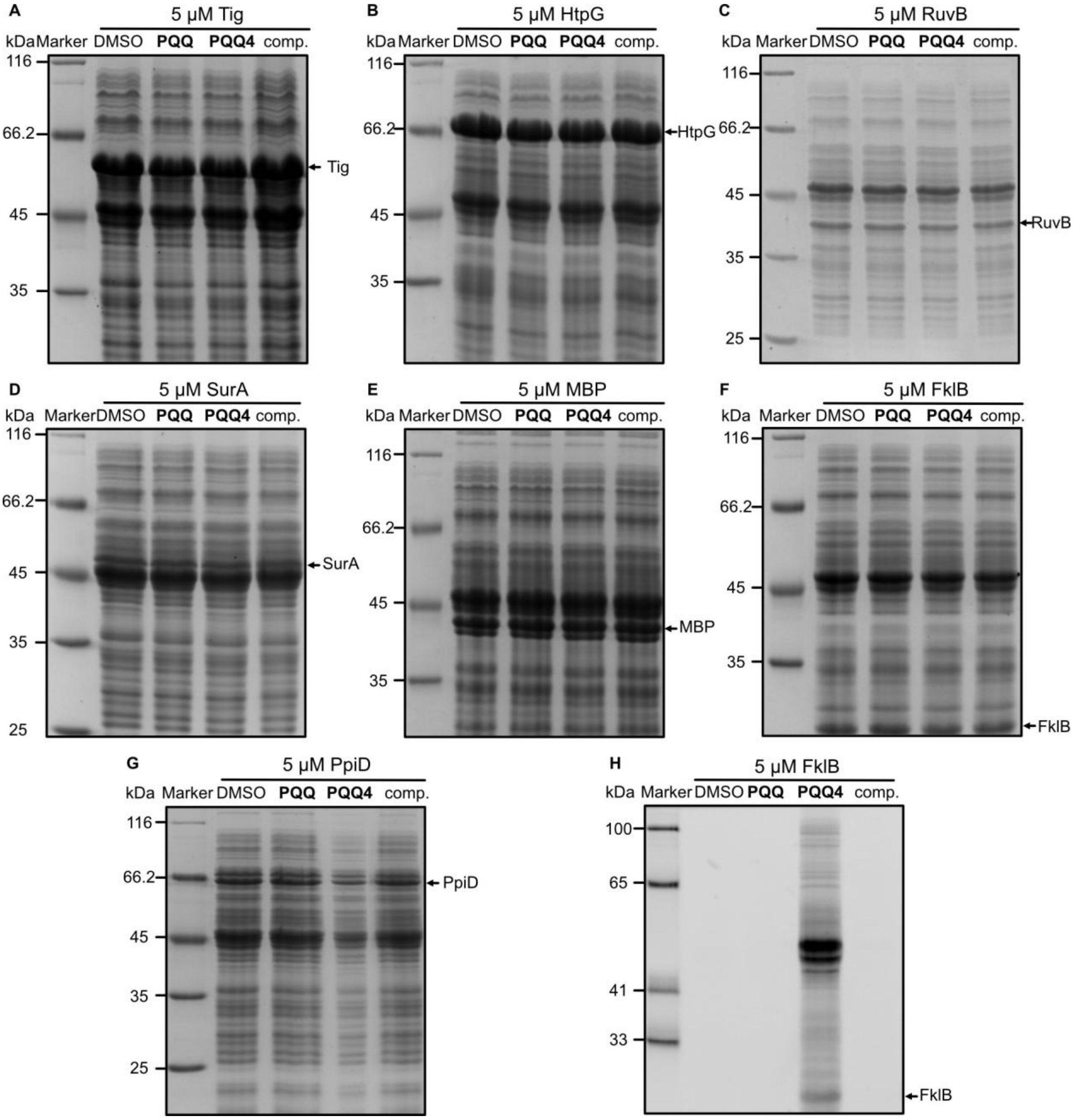

Figure S20. SDS-PAGE of *E. coli* K-12 lysate labeled by **PQQ4** with spiked purified recombinant proteins. (A) Coomassie SDS-PAGE of *E. coli* K-12 lysate, with 5 µM spiked Tig, labeled by 50 µM **PQQ4** compared to DMSO, 50 µM **PQQ** and competition condition (50 µM **PQQ4** + 1000 µM **PQQ**). (B) Coomassie SDS-PAGE as described in Figure S20A with 5 µM spiked HtpG. (C) Coomassie SDS-PAGE as described in Figure S20A with 5 µM spiked RuvB. (D) Coomassie SDS-PAGE as described in Figure S20A with 5 µM spiked SurA. (E) Coomassie SDS-PAGE as described in Figure S20A with 5 µM spiked MBP. (F) Coomassie SDS-PAGE as described in Figure S20A with 5 µM spiked FklB. (G) Coomassie SDS-PAGE as described in Figure S20A with 5 µM spiked PpiD. (H) Fluorescent SDS-PAGE as described in Figure S20A with 5 µM spiked FklB.

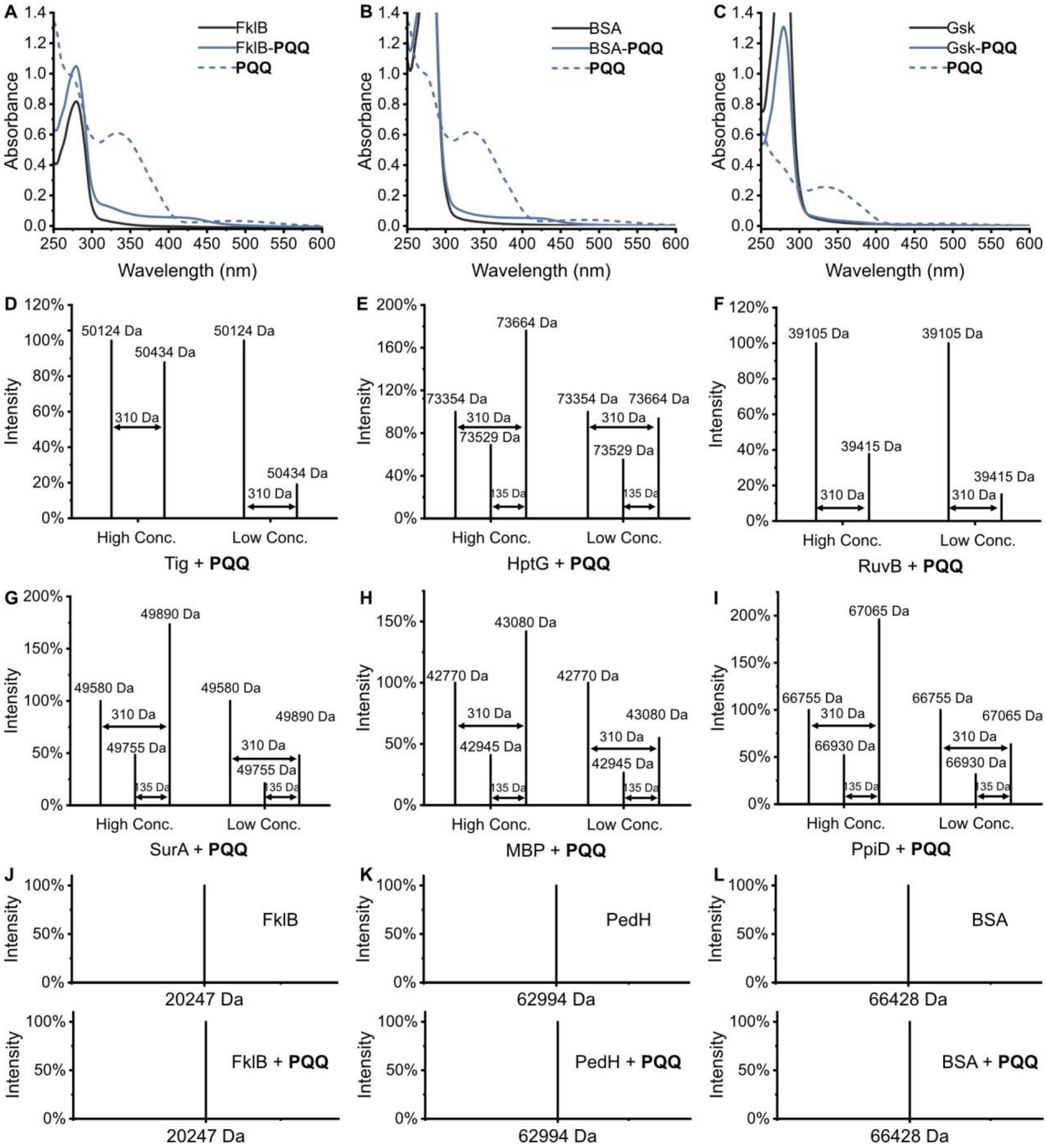

Figure S21. Identification of covalent binding proteins by UV-vis spectrometry and IPMS. (A) Absorption spectra of 100 µM FklB, 100 µM FklB-**PQQ** and 100 µM **PQQ**. (B) Absorption spectra of 100 µM BSA, 100 µM BSA-**PQQ** and 100 µM **PQQ**. (C) Absorption spectra of 50 µM Gsk, 50 µM Gsk-**PQQ** and 50 µM **PQQ**. (D) IPMS of Tig treated with low **PQQ** concentration (100 µM Tig treated with 3-fold molar excess **PQQ** and 3-fold molar excess $CaCl_2$ at 4 ºC for 12 h), and high **PQQ** concentration (100 µM Tig treated with 20-fold molar excess **PQQ** and 20-fold molar excess $CaCl_2$ at 37 ºC for 2 h). Here the intensity indicates the deconvoluted mass intensity by UniDec, same applies for Figure S21E-L. (E) IPMS of 100 µM HtpG treated with **PQQ**. Here the experimental details are as in Figure S21D. (F) IPMS of 100 µM RuvB treated with low **PQQ** concentration (100 µM RuvB treated with 3-fold molar excess **PQQ** and 3-fold molar excess $MgCl_2$ at 4 ºC for 12 h), and high **PQQ** concentration (100 µM RuvB treated with 20-fold molar excess **PQQ** and 20-fold molar excess $MgCl_2$ at 30 ºC for 2 h). (G) IPMS of 100 µM SurA treated with **PQQ**. Here the experimental details are as in Figure S21D. (H) IPMS of 100 µM MBP treated with **PQQ**. Here the experimental details are as in Figure S21D. (I) IPMS of 100 µM PpiD treated with **PQQ**. Here the experimental details are as in Figure S21D. (J) IPMS of 100 µM FklB treated with 20-fold molar excess **PQQ** and 20-fold molar excess $CaCl_2$ at 37 ºC for 2 h. (K) IPMS of 100 µM PedH treated with 20-fold molar excess **PQQ** and 20-fold molar excess $LaCl_3$ at 30 ºC for 2 h. (L) IPMS of 100 µM BSA treated with 20-fold molar excess **PQQ** and 20-fold molar excess $CaCl_2$ for at 37 ºC 2 h.

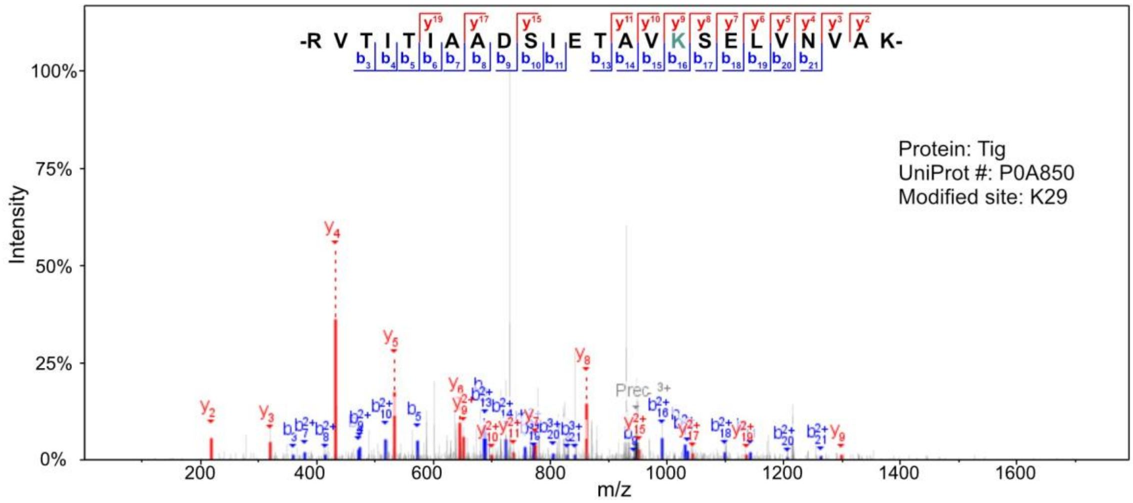

Figure S22. MS/MS spectrum of Tig peptide (-RVTITIAADSIETAVKSELVNVAK-) identified by Fragpipe PDV

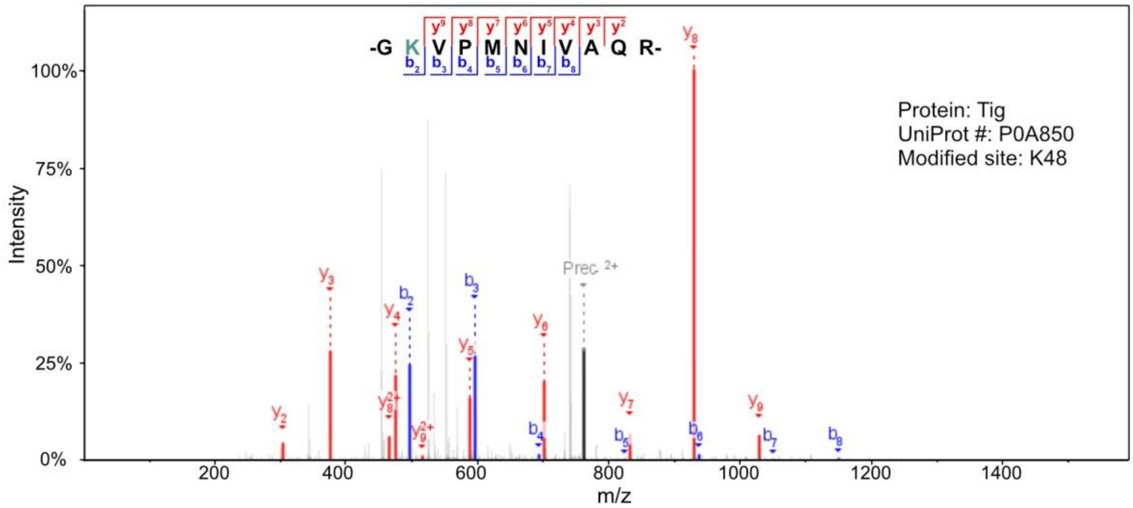

Figure S23. MS/MS spectrum of Tig peptide (-GKVPMNIVAQR-) identified by Fragpipe PDV

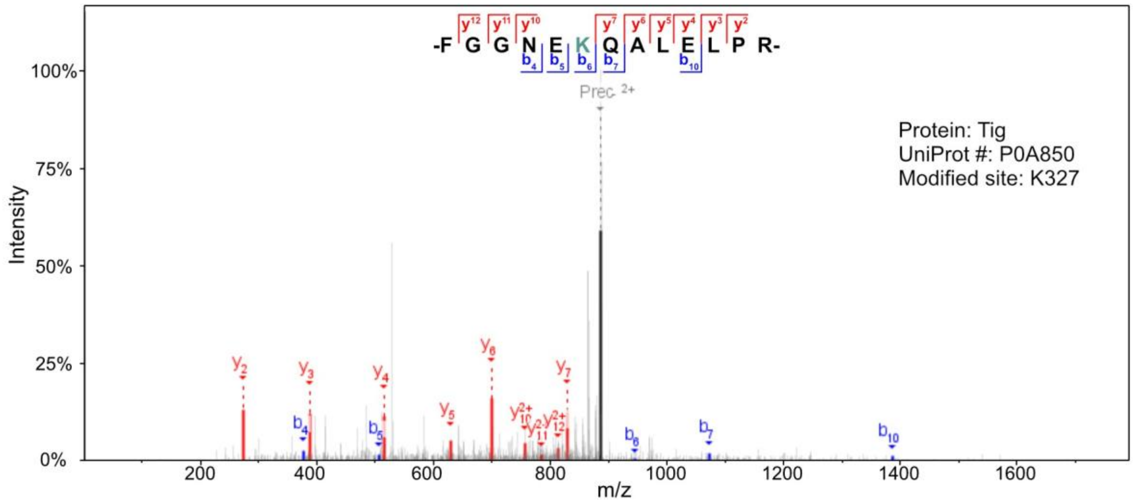

Figure S24. MS/MS spectrum of Tig peptide (-FGGNEKQALELPR-) identified by Fragpipe PDV

A

Proteins	Position	Localization prob	FLR	Score diff	PEP	Score	Delta score	Score for localization
RuvB	K26	1	0	58.093	6.21E-12	129.05	115.63	124.43
RuvB	K267	0.996666	0.000476	24.7555	0.005913	68.735	38.165	67.563
RuvB	K46	0.85621	0.023806	7.74852	0.000717	80.755	73.549	56.632

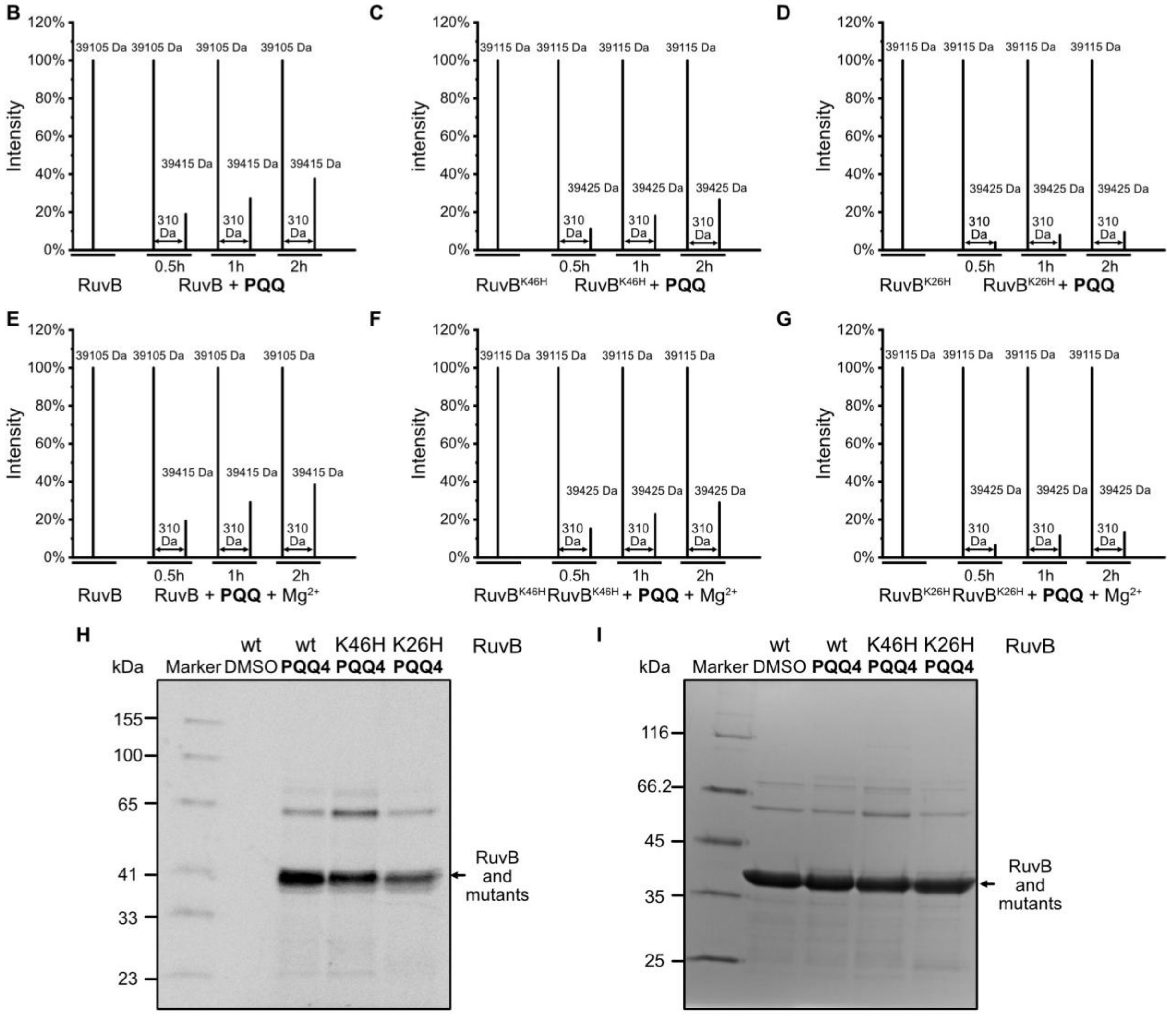

Figure S25. Binding site identification of RuvB by MS/MS and IPMS. (A) Table of RuvB binding site identification from **PQQ** and $MgCl_2$ treatment analyzed by Maxquant. 100 μM RuvB was treated with 20-fold molar excess **PQQ** and 20-fold molar excess $MgCl_2$, and incubated at 30 ºC for 1 h. (B) Time-dependent labeling of intact RuvB treated with **PQQ**. 100 μM RuvB treated with 20-fold molar excess **PQQ** was incubated at 30 ºC for time-dependent labeling, and here the intensity indicates the deconvoluted protein intensity from UniDec, same applies for Figure S25C,D. (C) Time-dependent labeling of intact $RuvB^{k46H}$ treated with **PQQ**. (D) Time-dependent labeling of intact $RuvB^{k26H}$ treated with **PQQ**. (E) Time-dependent labeling of intact RuvB treated with **PQQ** and $MgCl_2$. 100 μM RuvB treated with 20-fold molar excess **PQQ** and 20-fold molar excess $MgCl_2$, was incubated at 30 ºC for time-dependent labeling, and here the intensity indicates the deconvoluted protein intensity from UniDec, same applies for Figure S25F,G. (F) Time-dependent labeling of intact $RuvB^{k46H}$ treated with **PQQ**. (G) Time-dependent labeling of intact $RuvB^{k26H}$ treated with **PQQ**. (H) Fluorescent SDS-PAGE of RuvB and mutants labeled by **PQQ4** compared to DMSO.10 μM RuvB and mutants were treated with 5 μM **PQQ4** at room temperature for 2 h, which were then directly performed click reaction (no UV irradiation), subsequent loaded with same amount of protein for each lane of SDS gel, same applies for Figure S25I. (I) Coomassie SDS-PAGE of RuvB and mutants labeled by **PQQ4** compared to DMSO.

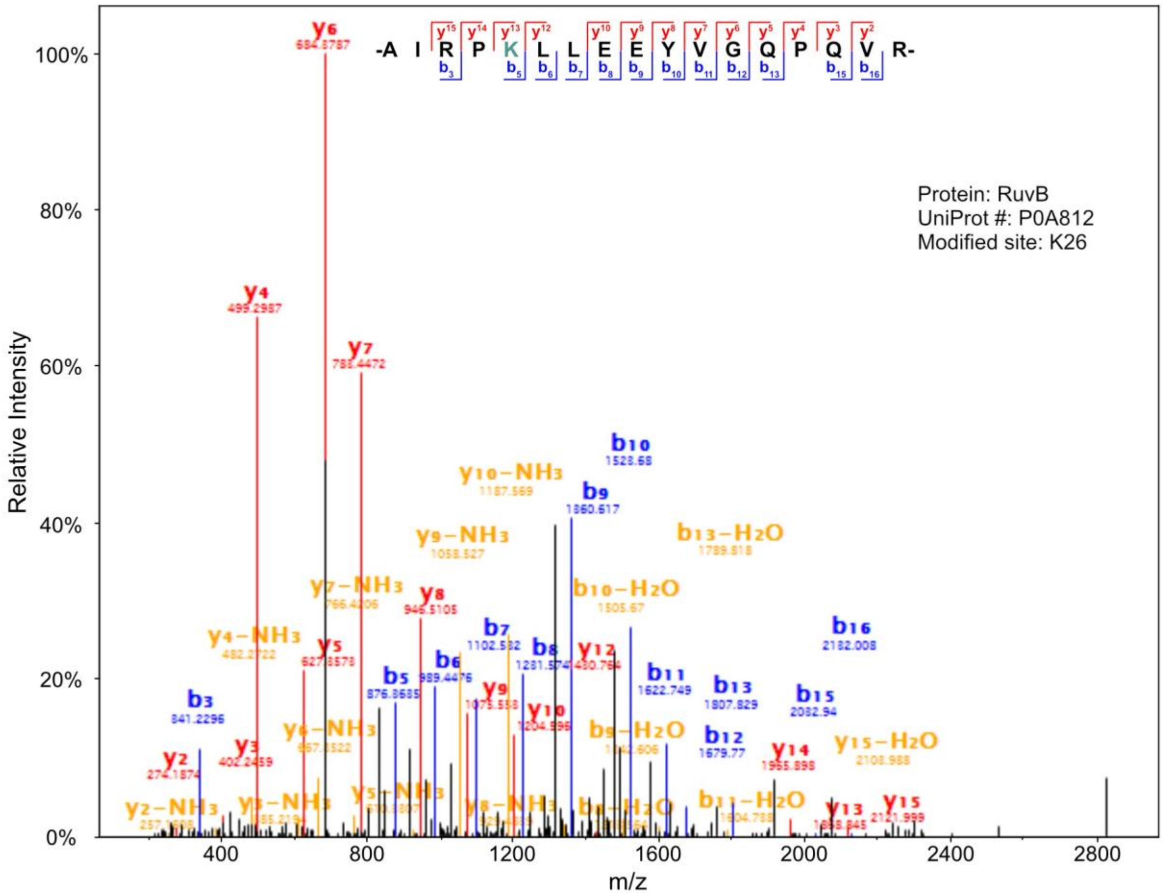

Figure S26. MS/MS spectrum of RuvB peptide (-AIRPKLLEEYVGQPQVR-) identified by Maxquant.

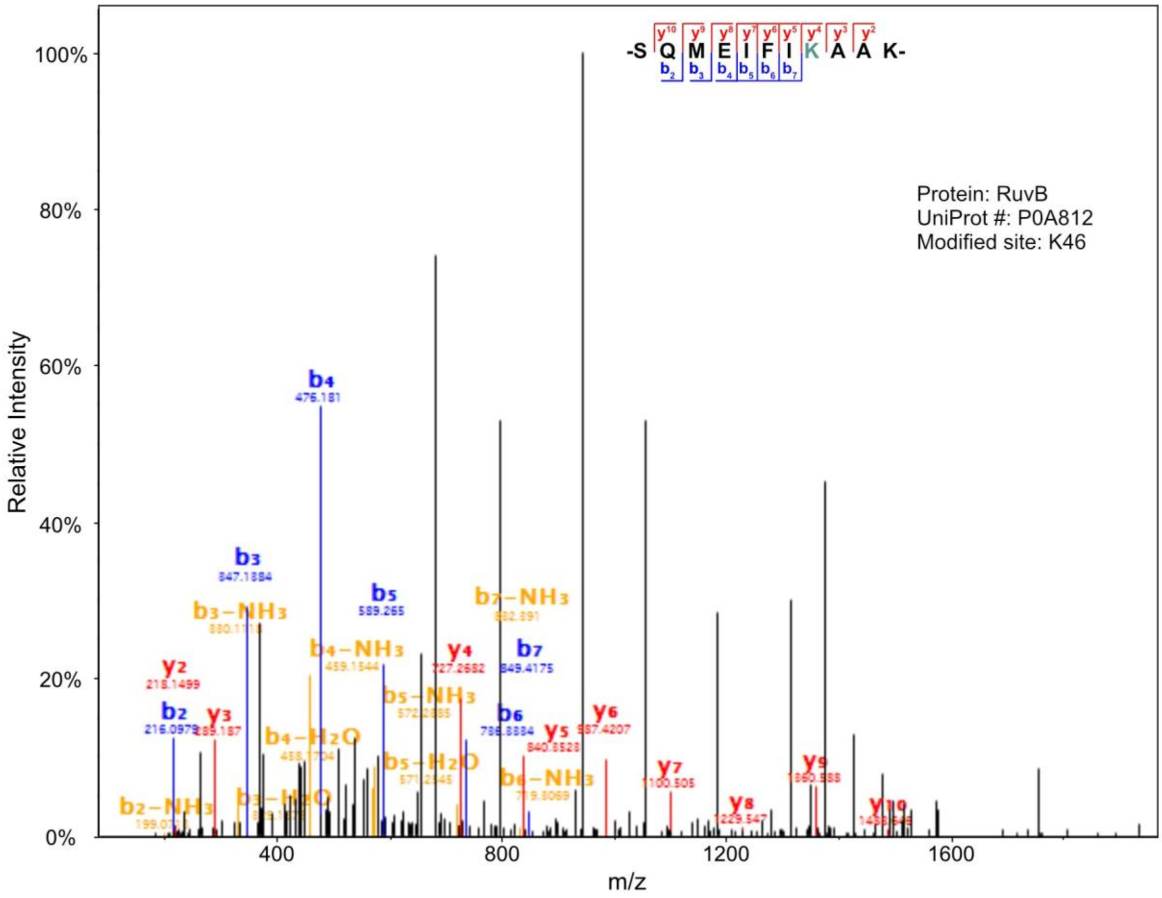

Figure S27. MS/MS spectrum of RuvB peptide (-SQMEIFIKAAK-) identified by Maxquant.

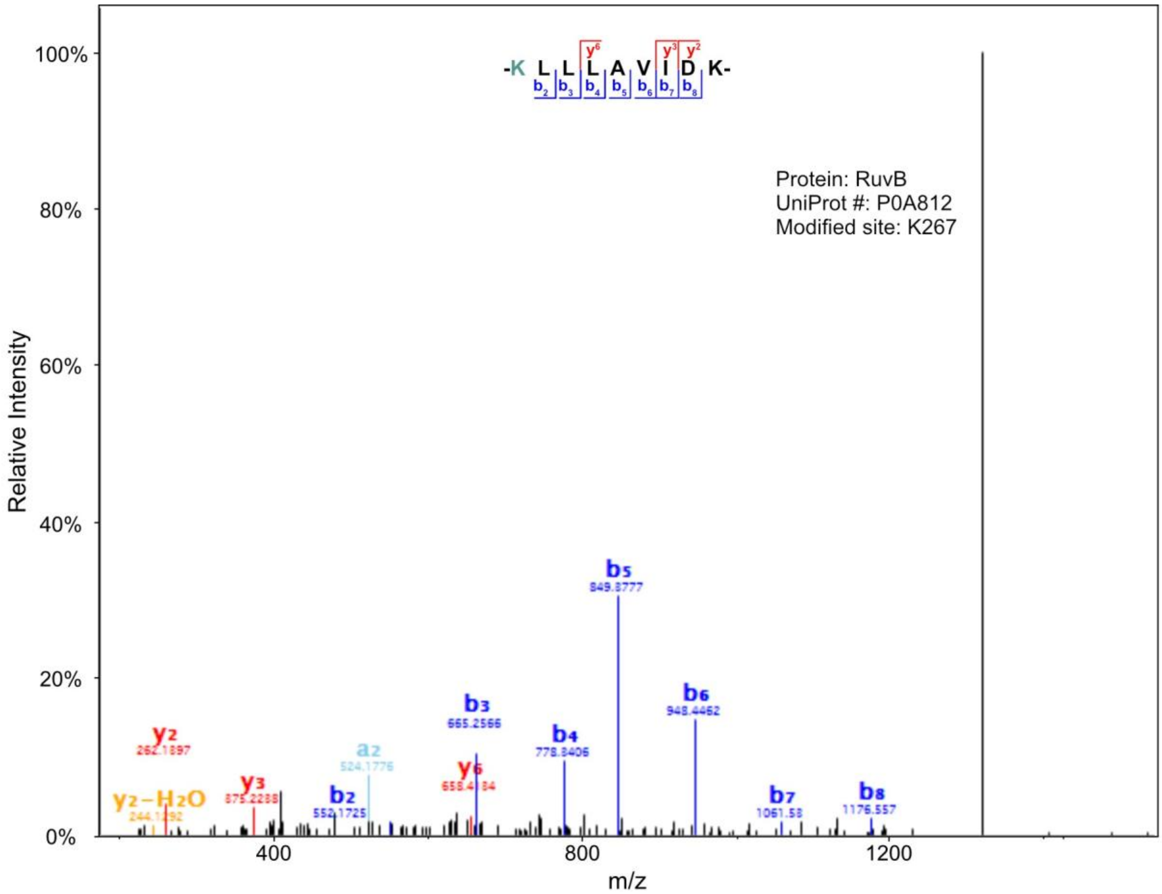

Figure S28. MS/MS spectrum of RuvB peptide (-KLLLAVIDK-) identified by Maxquant.

Table S26. Detected PQQ-associated proteins compared to known and predicted PQQ-dependent enzymes

Annotation Status	Protein Name
Known **PQQ**-dependent enzymes	YliI, Gcd, PedH, PedE
Predicted **PQQ**-dependent enzymes	QuiA, BamB
Detected **PQQ**-associated proteins in this study	YliI, Gcd, PedH, BamB

Predicted proteins are considered to be **PQQ**-dependent enzymes if they are bioinformatically predicted by at least one of those databases (PANTHER, SUPFAM) and at least 2 of those databases (InterPro, NCBIfam, Pfam and SMART).

Table S27. Detected PQQ-binding proteins compared to known and predicted PQQ-binding proteins

Annotation Status	Protein Name
Known **PQQ**-binding proteins	YliI, Gcd, PedH, PedE, BamB, PqqC, PqqU
Predicted **PQQ**-binding proteins	QuiA, PP_5538, PP_2666, PP_2668, PP_2669, PP_2678
Detected **PQQ**-binding proteins in this study	YliI, Gcd, PedH, BamB, PqqC, Tig, HtpG, SurA, FklB, RuvB, PpiD, MBP

Predicted proteins are considered to be **PQQ**-binding proteins if they are bioinformatically predicted by at least 2 of those databases (InterPro, NCBIfam, Pfam and SMART).

4. NMR Spectra

^{1}H NMR of **1**

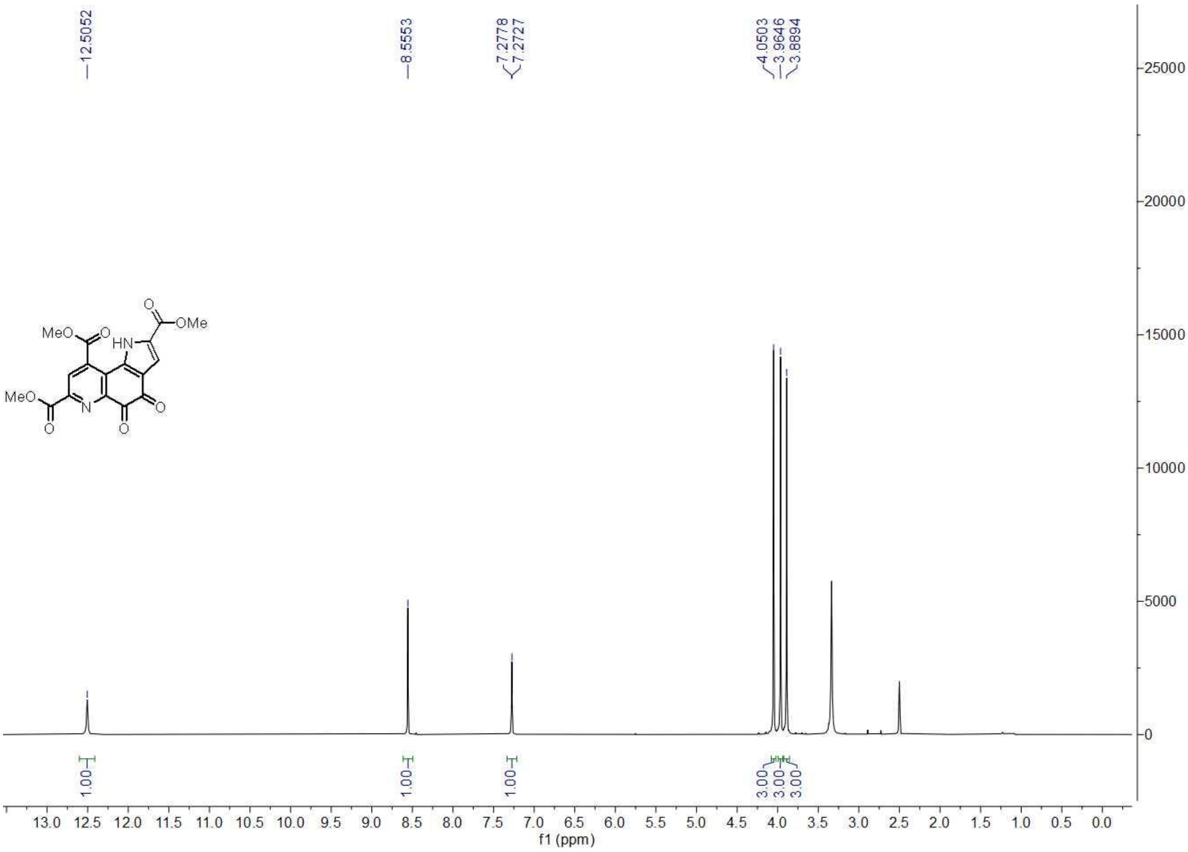

^{13}C NMR of **1**

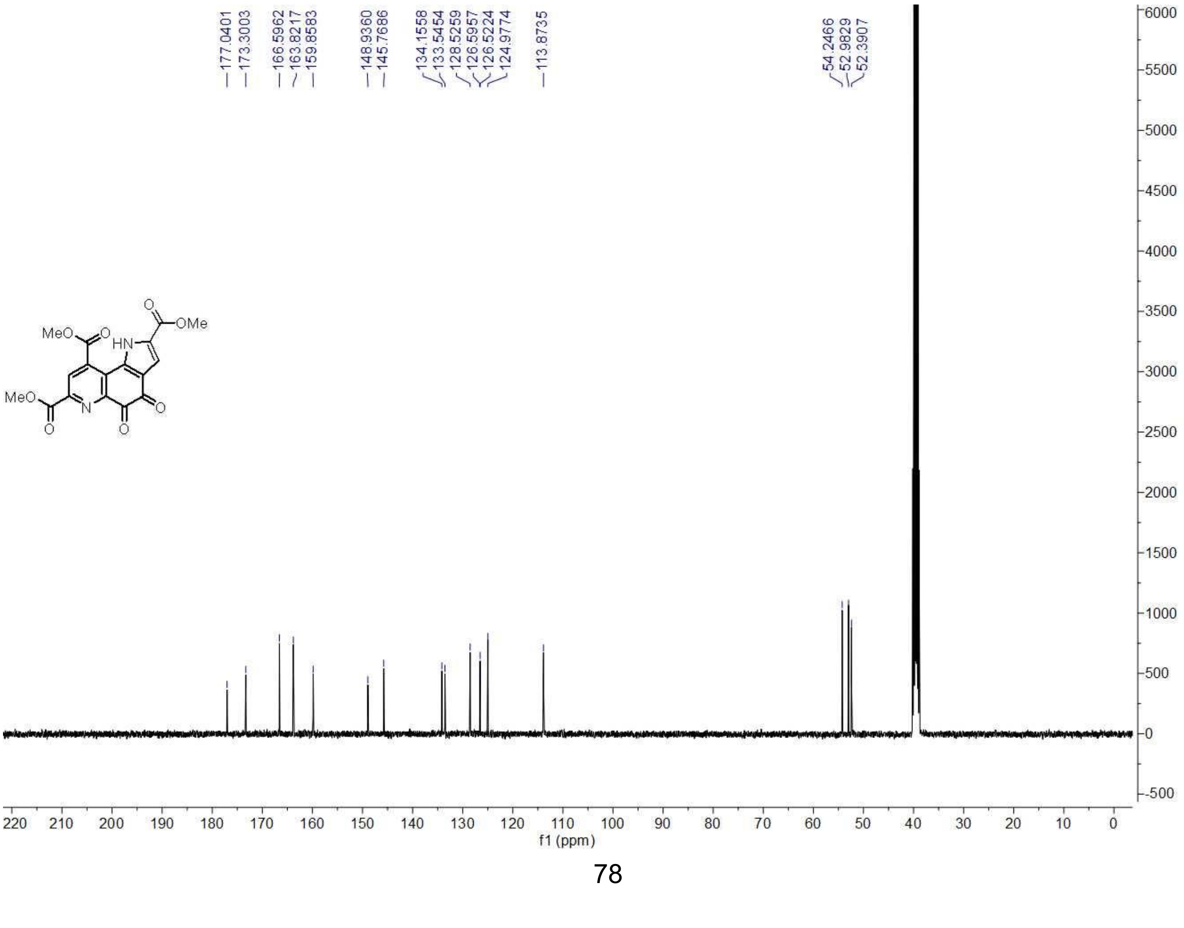

^{1}H NMR of **2**

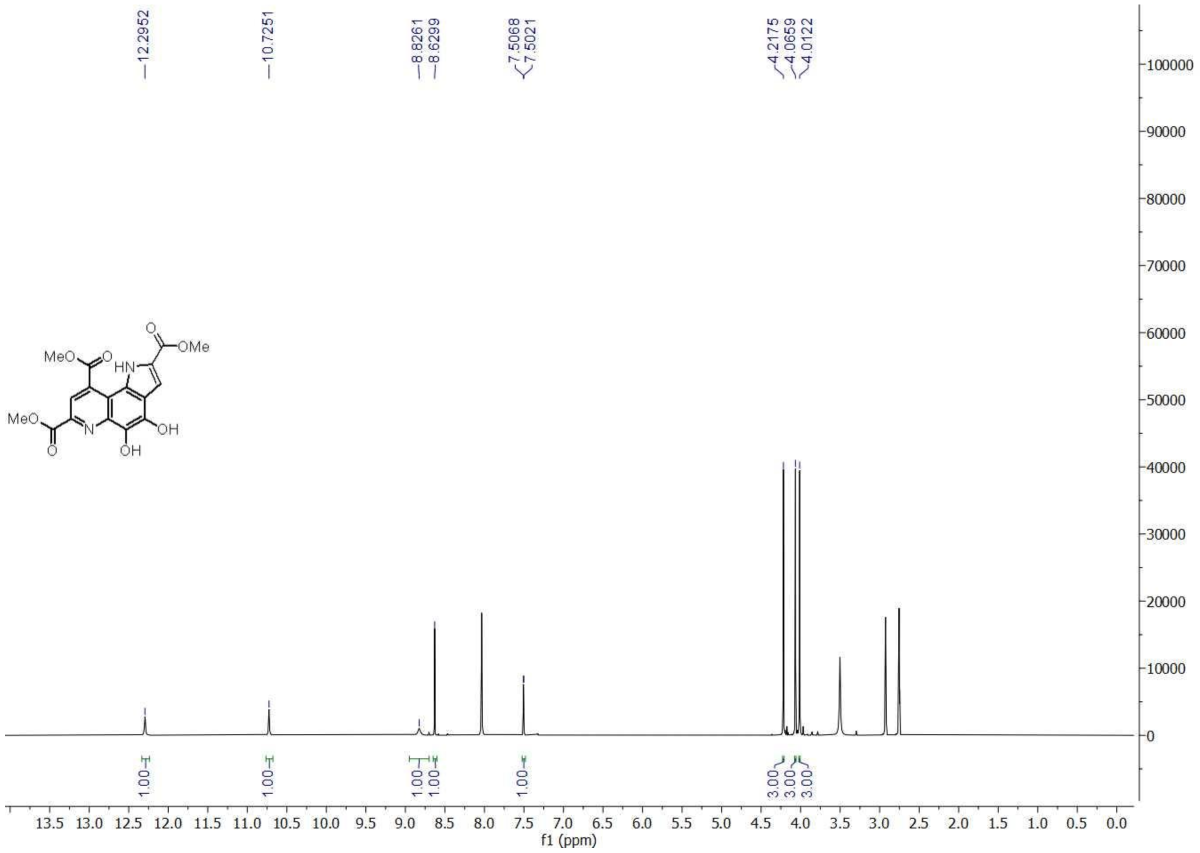

^{13}C NMR of **2**

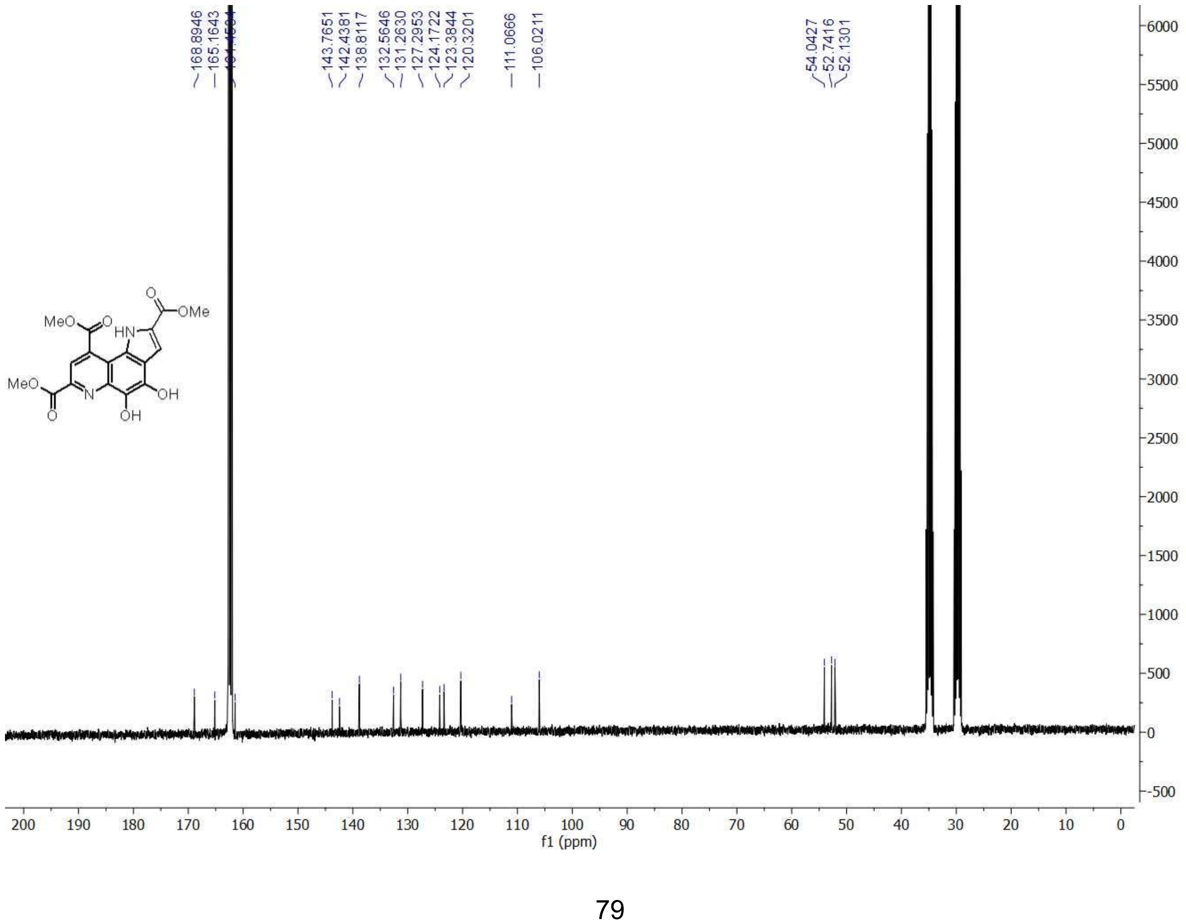

^{1}H NMR of **PQQ1**

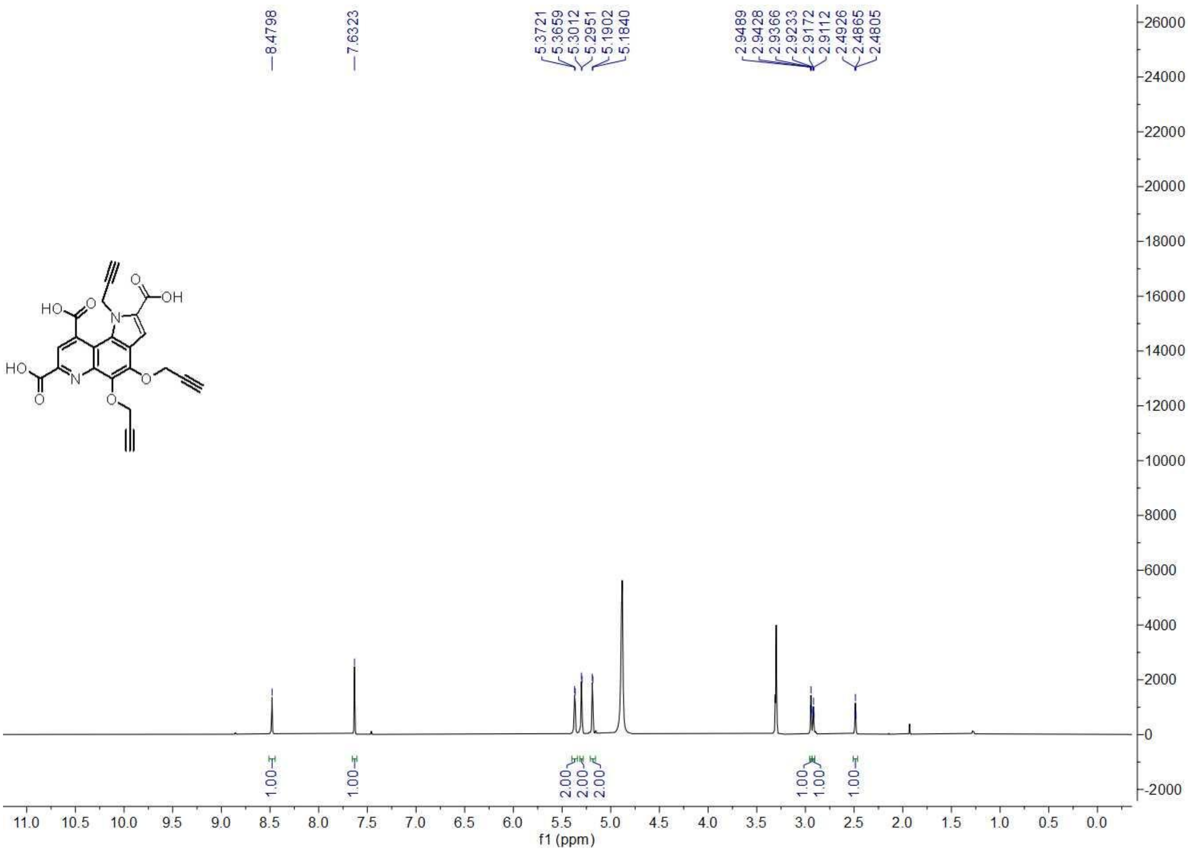

^{13}C NMR of **PQQ1**

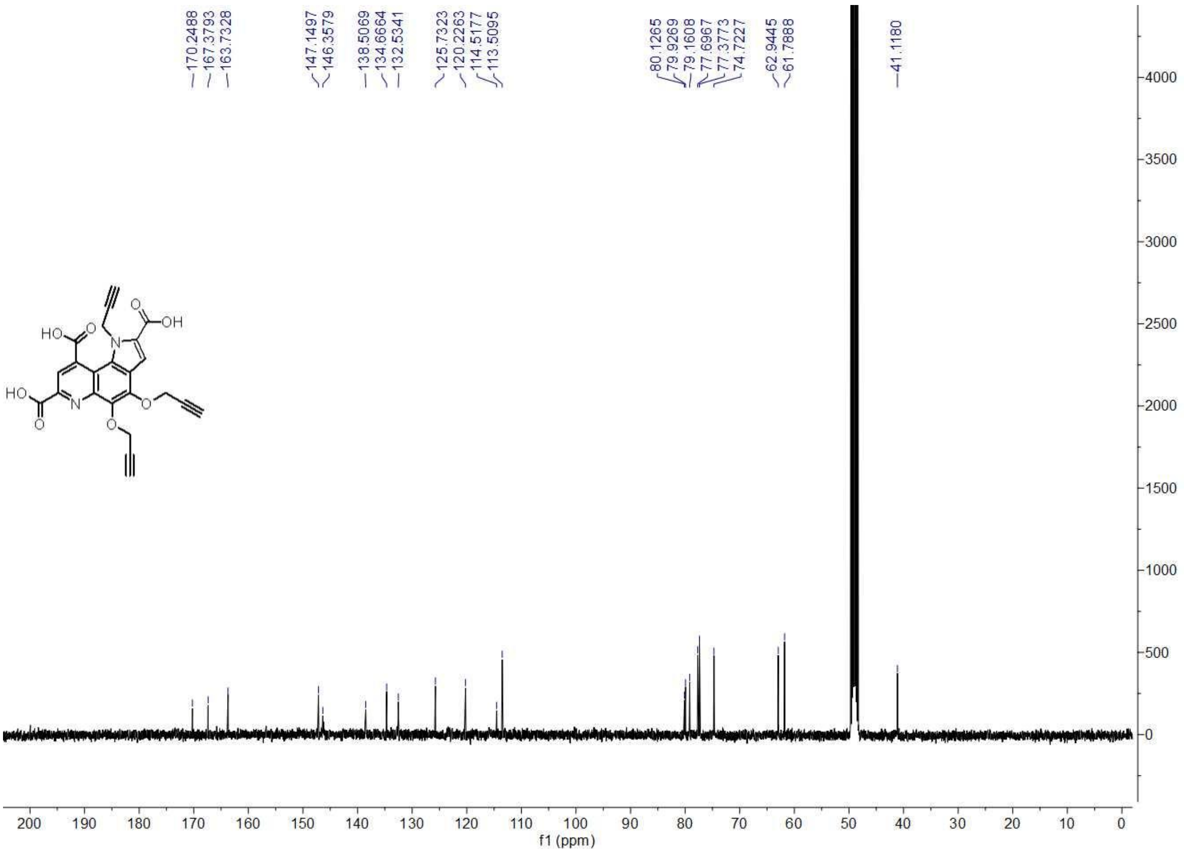

^{1}H NMR of **3**

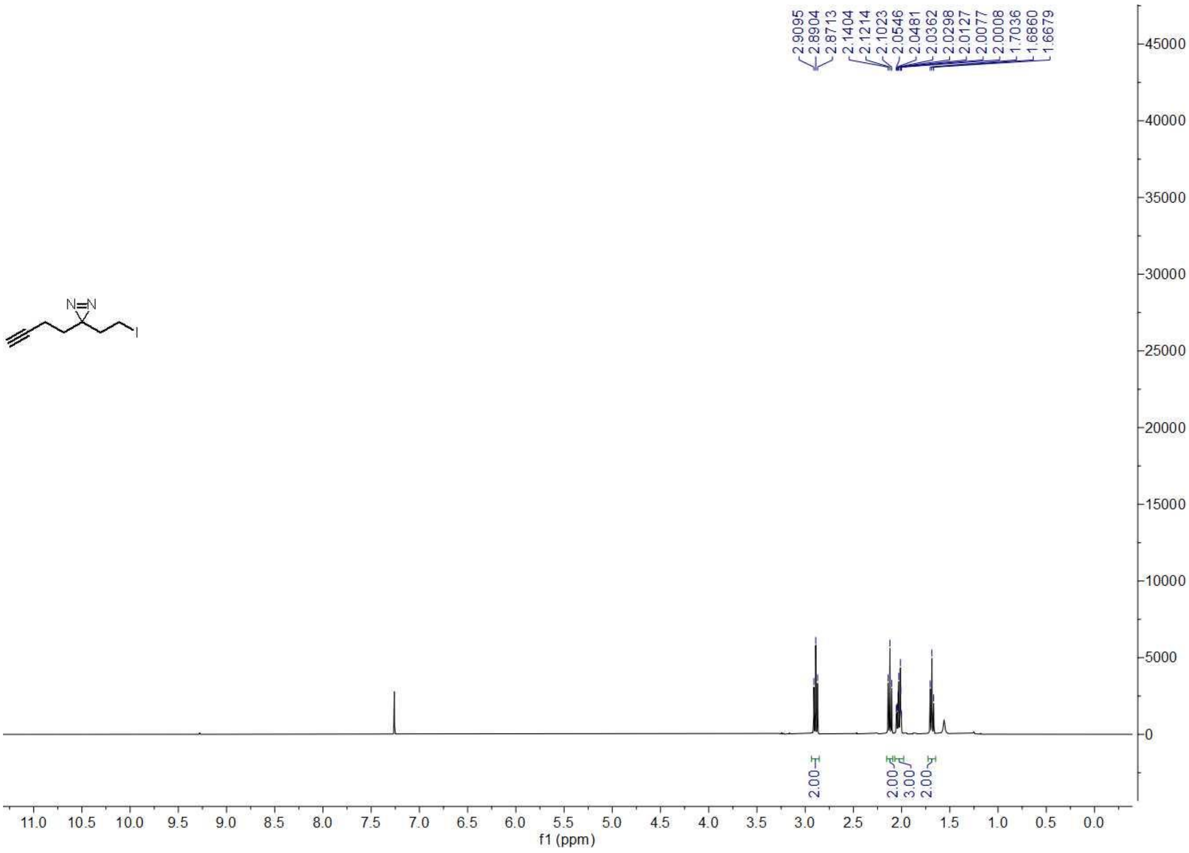

^{13}C NMR of **3**

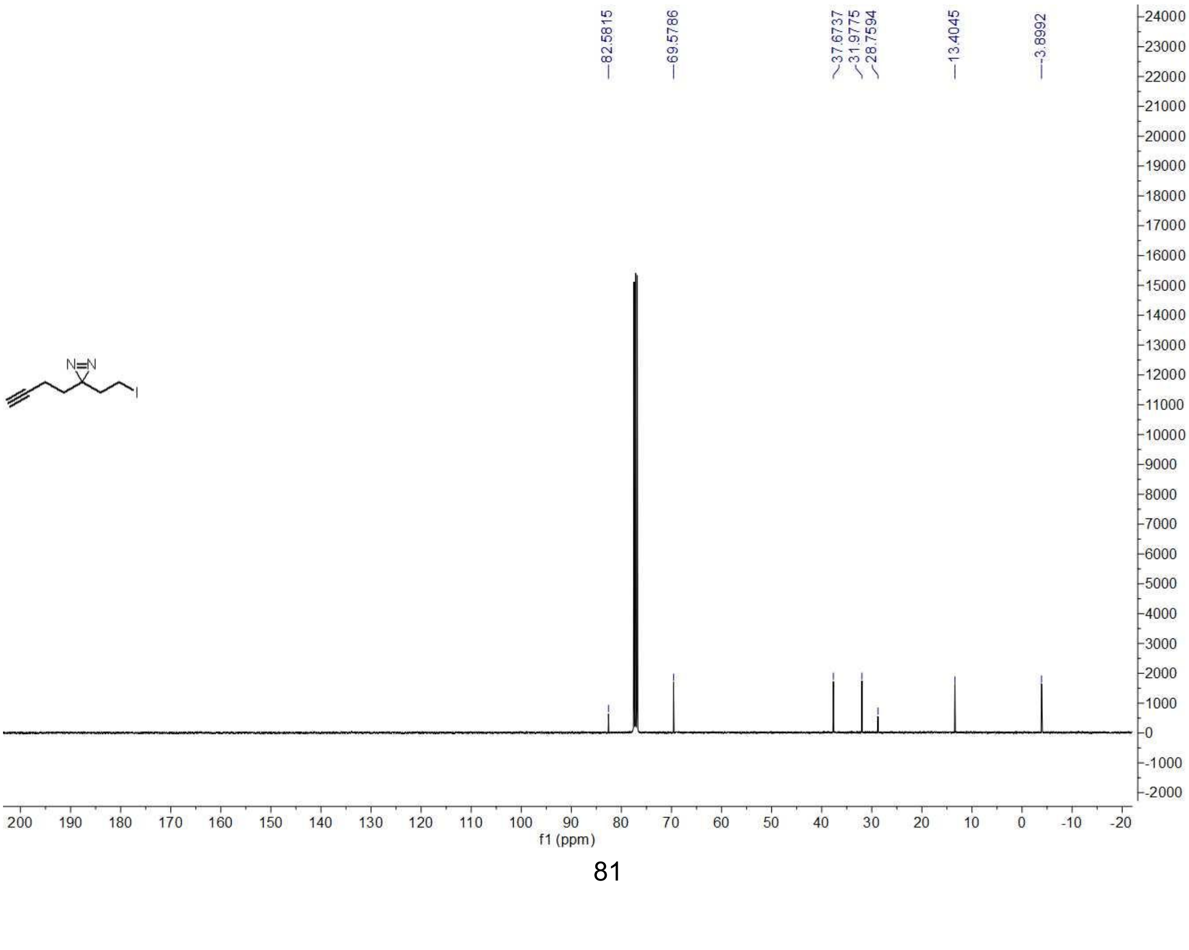

^{1}H NMR of **PQQ2**

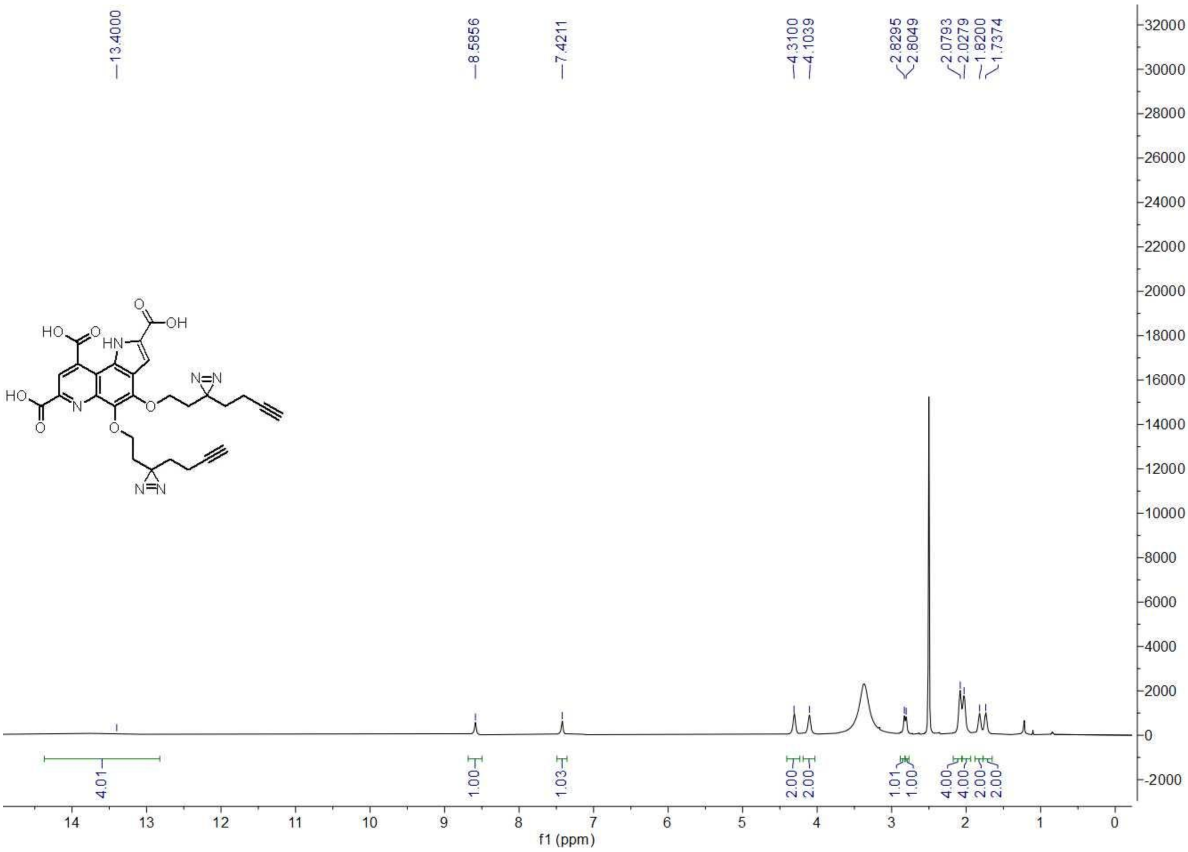

^{13}C NMR of **PQQ2**

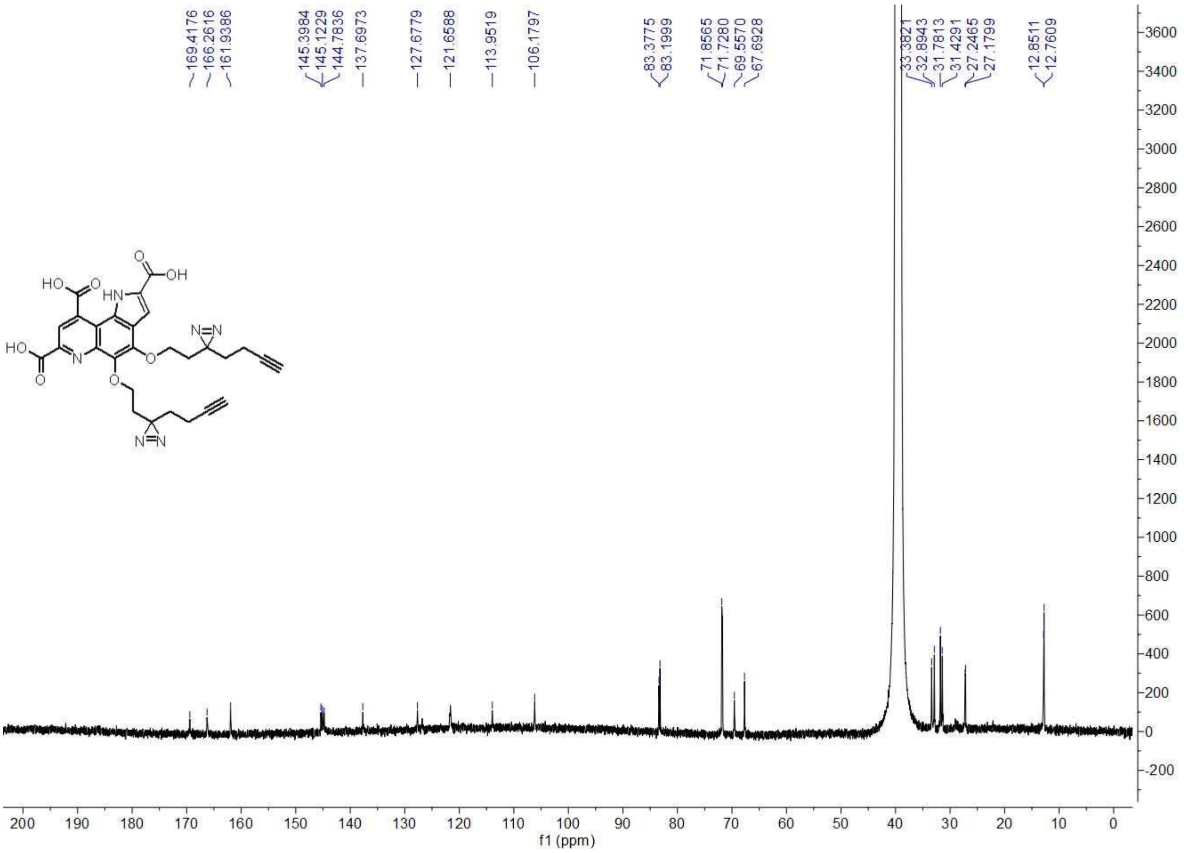

^{1}H NMR of **4**

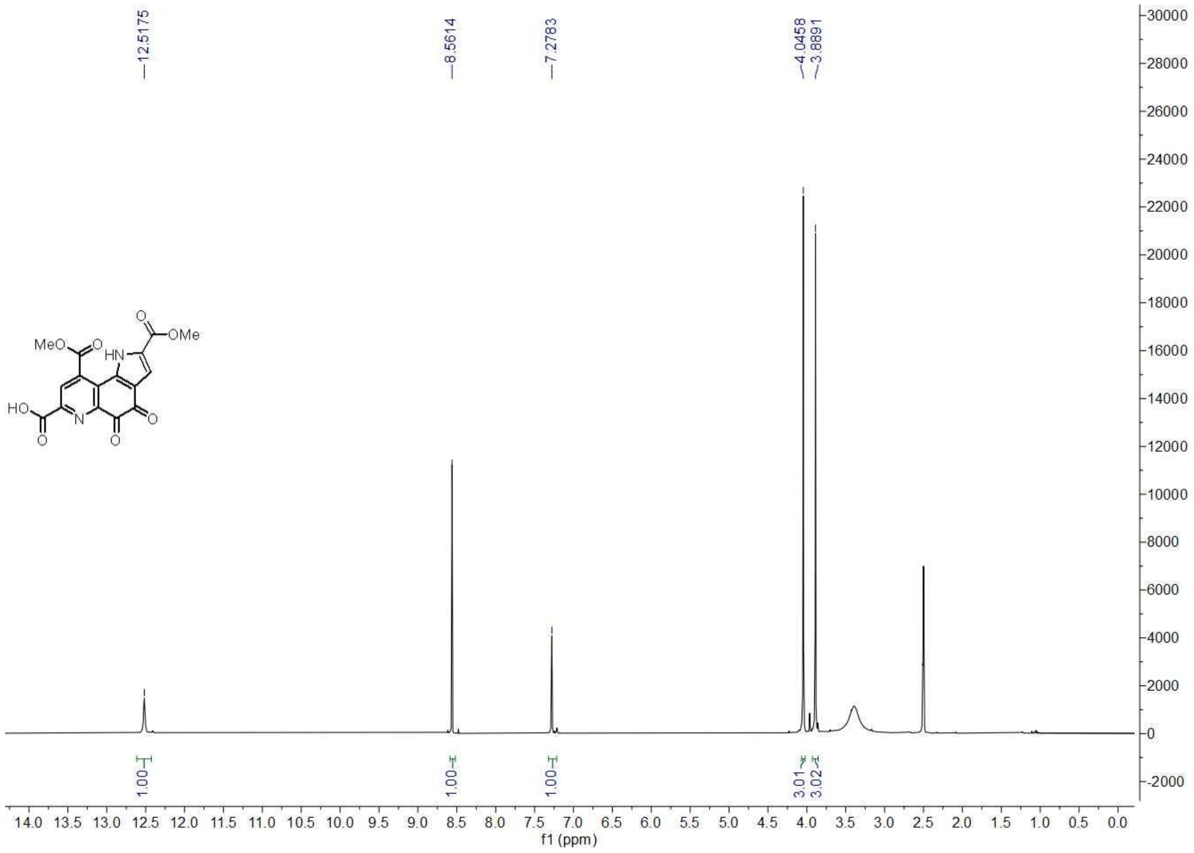

^{13}C NMR of **4**

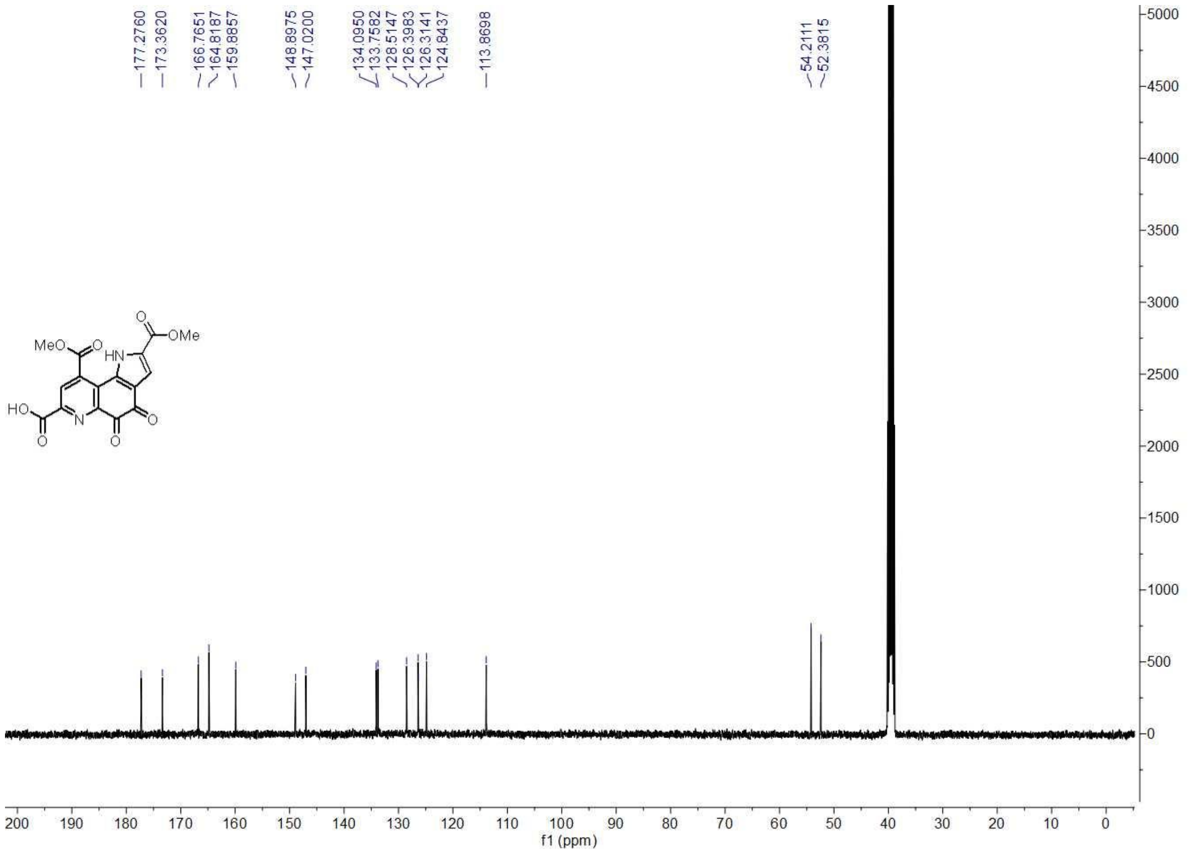

^{1}H NMR of **PQQ4**

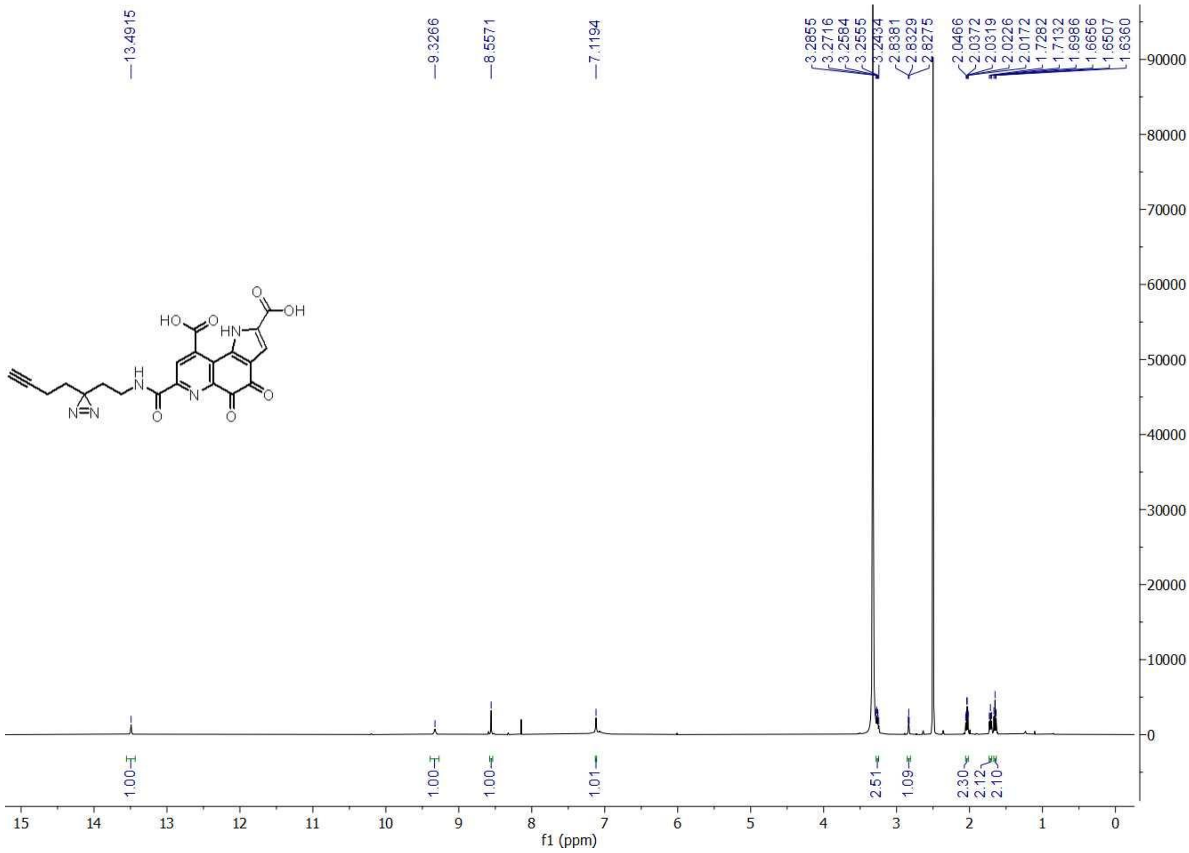

^{13}C NMR of **PQQ4**

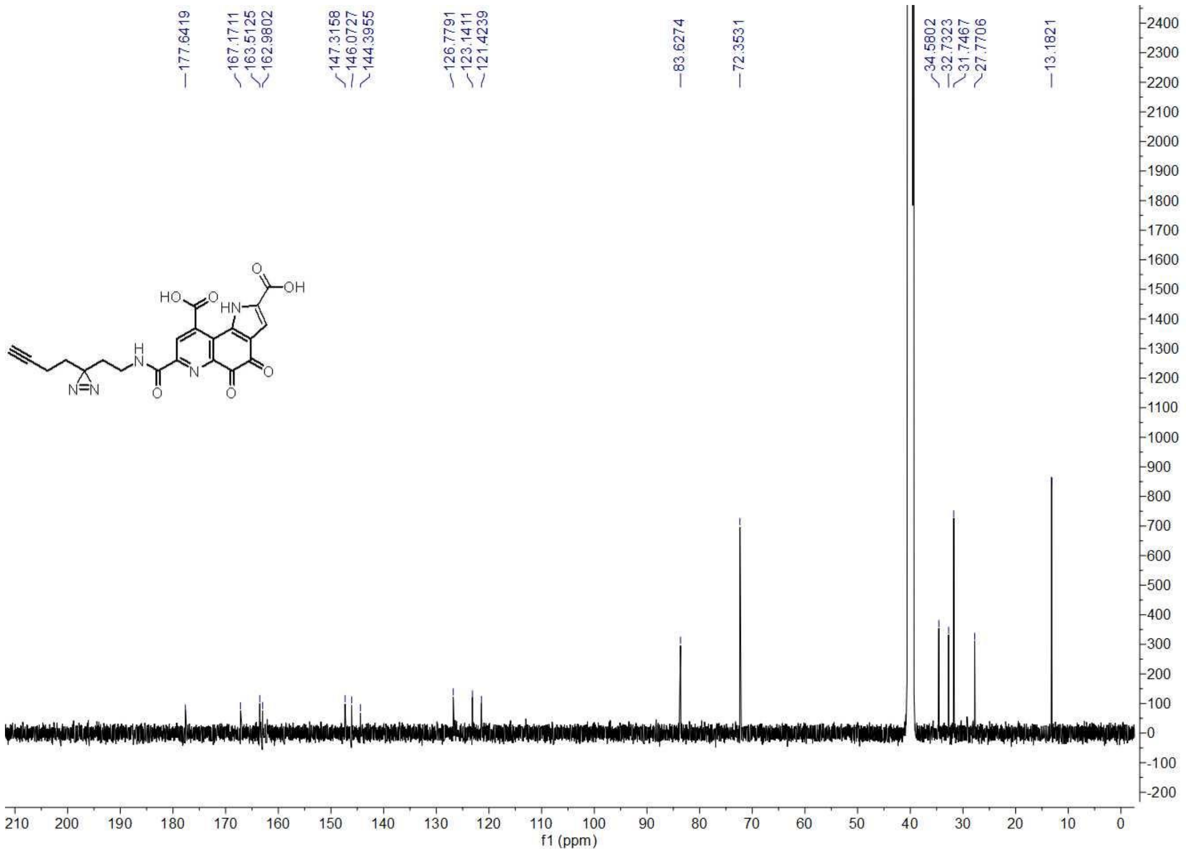

^{1}H NMR of **6**

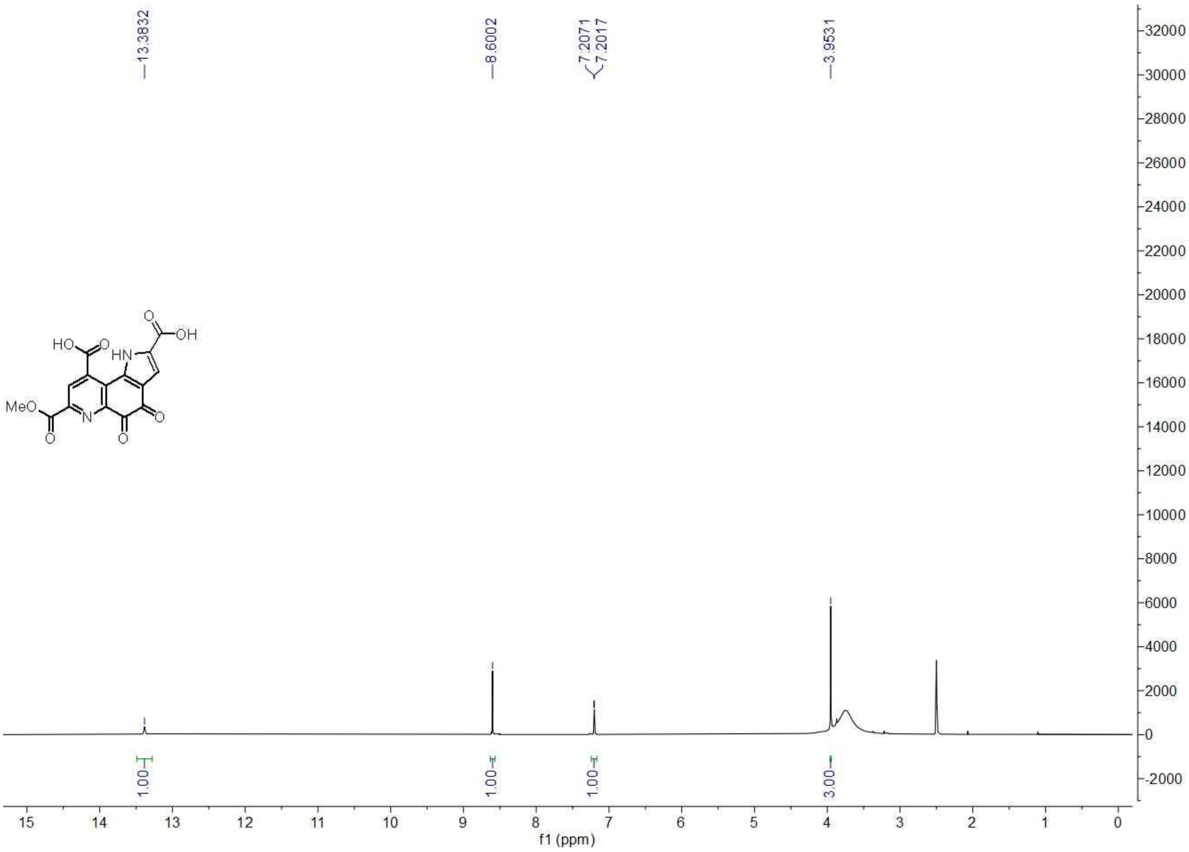

^{13}C NMR of **6**

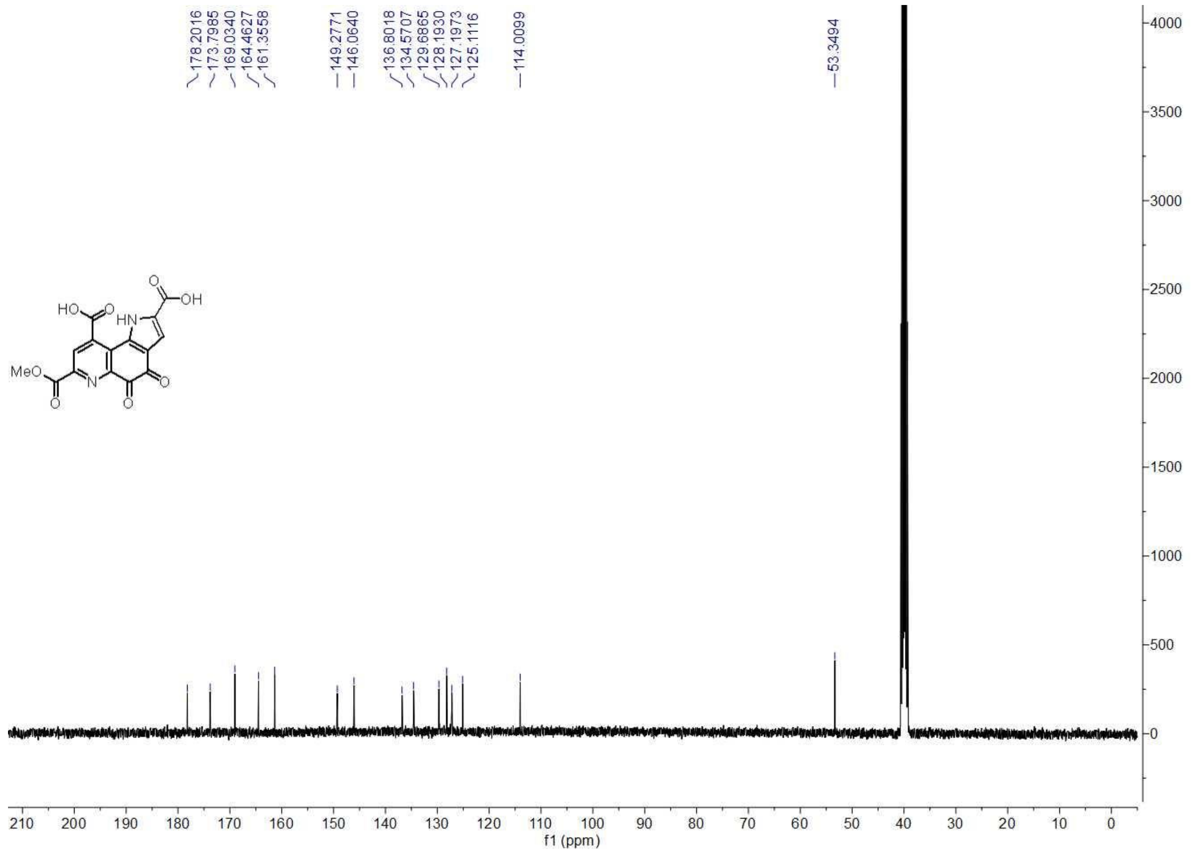

^{1}H NMR of **7**

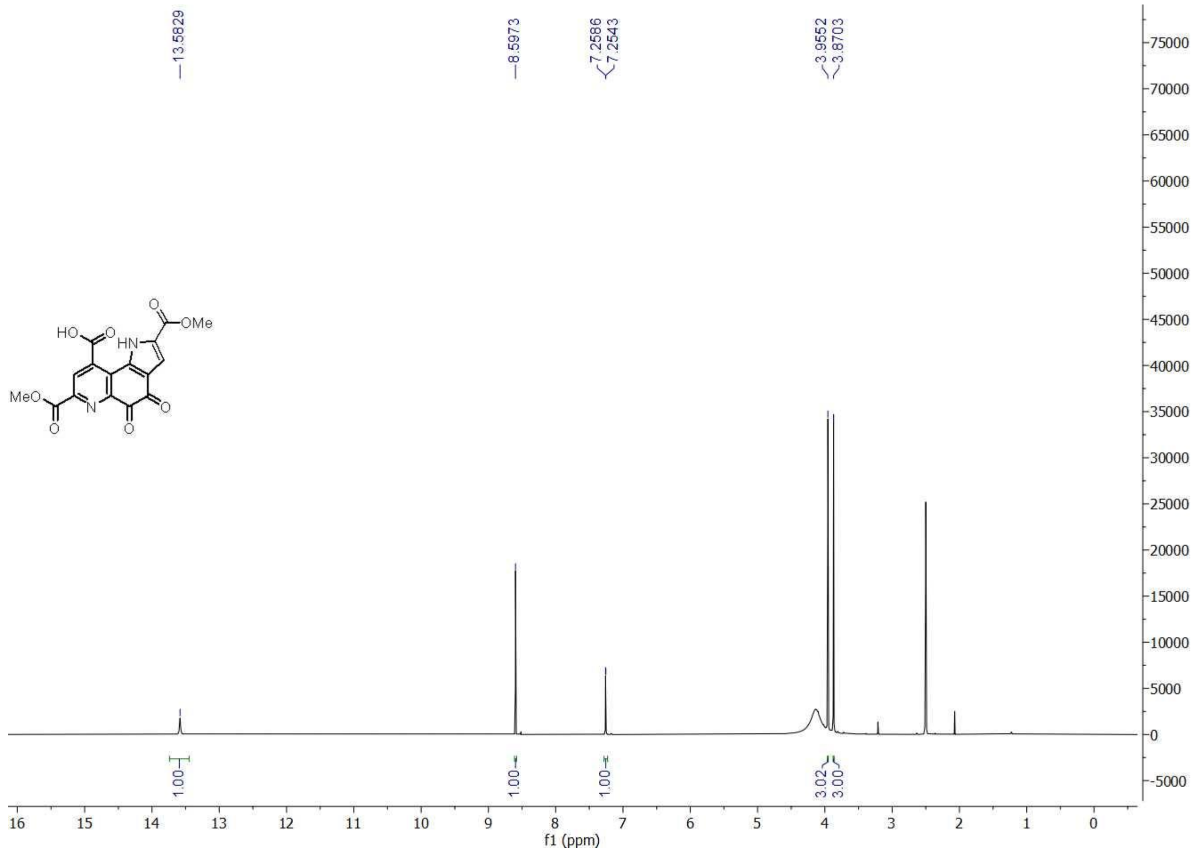

^{13}C NMR of **7**

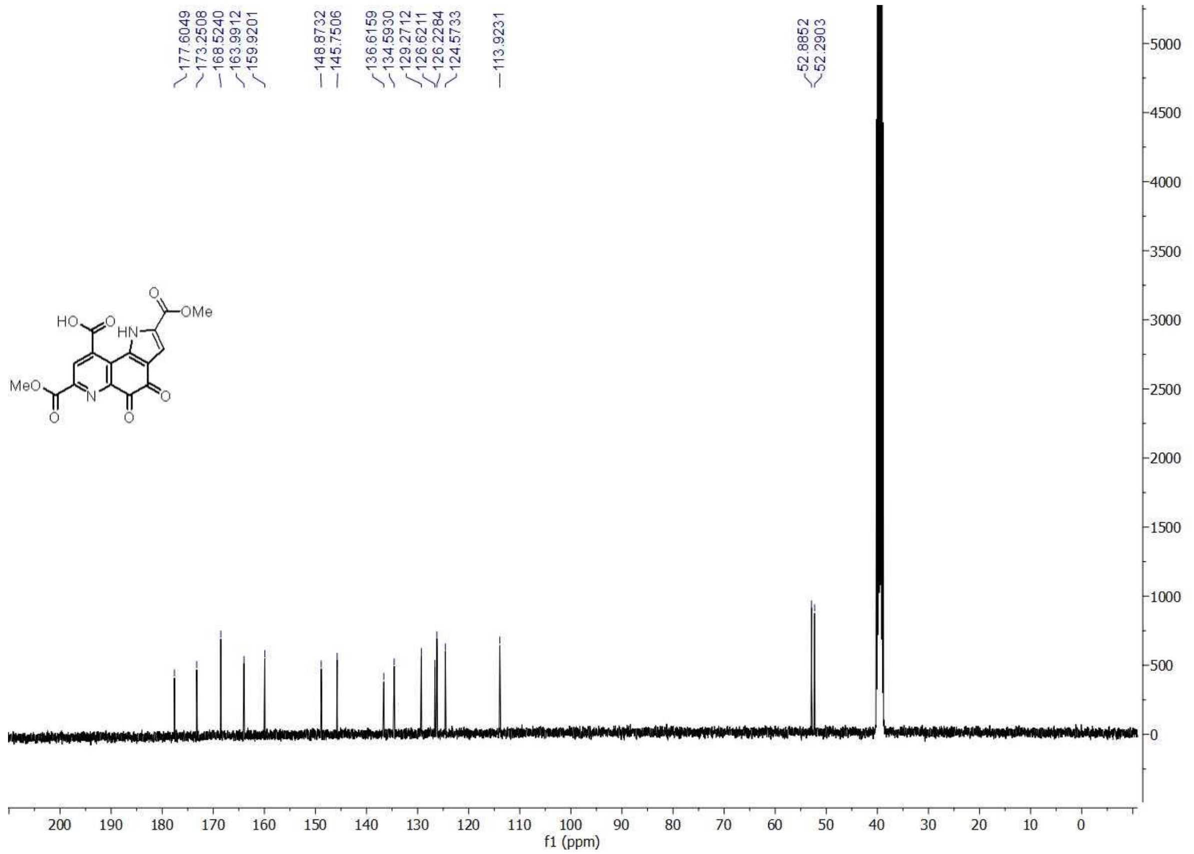

^{1}H NMR of **PQQ6**

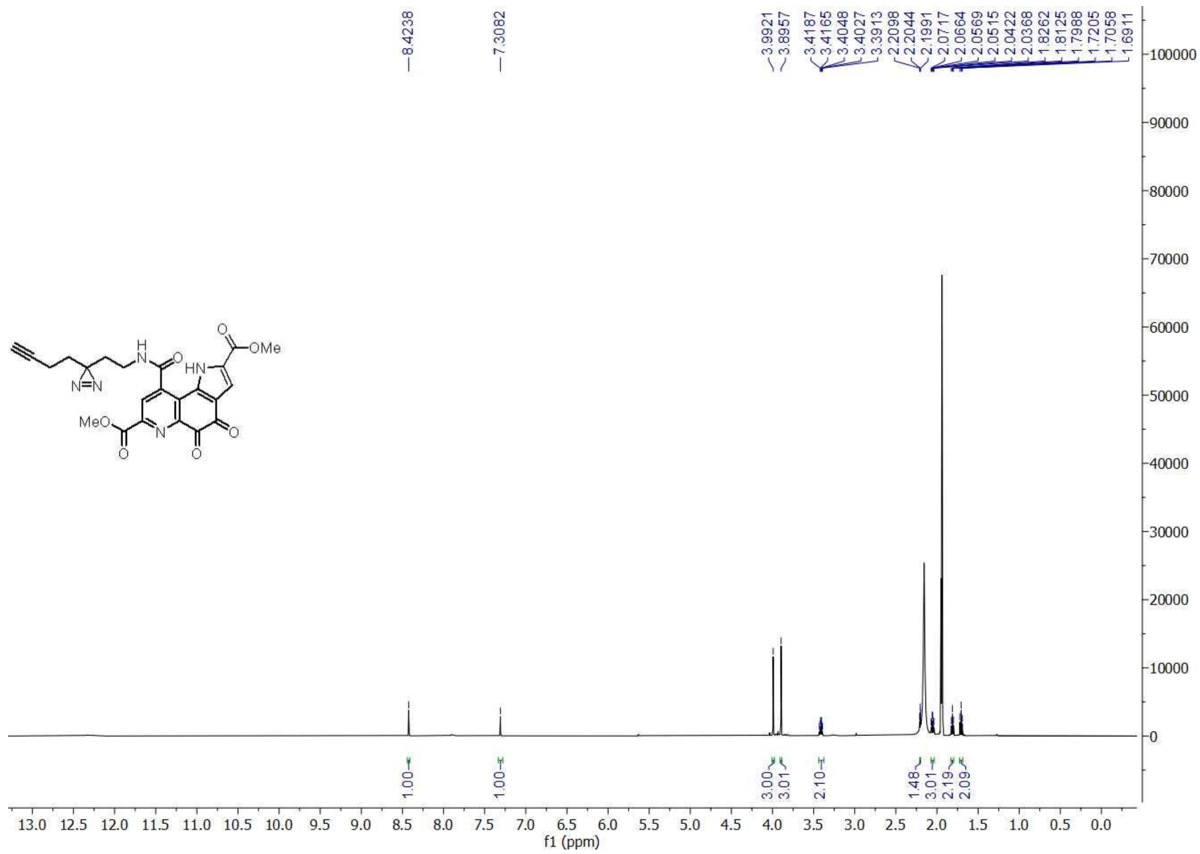

^{13}C NMR of **PQQ6**

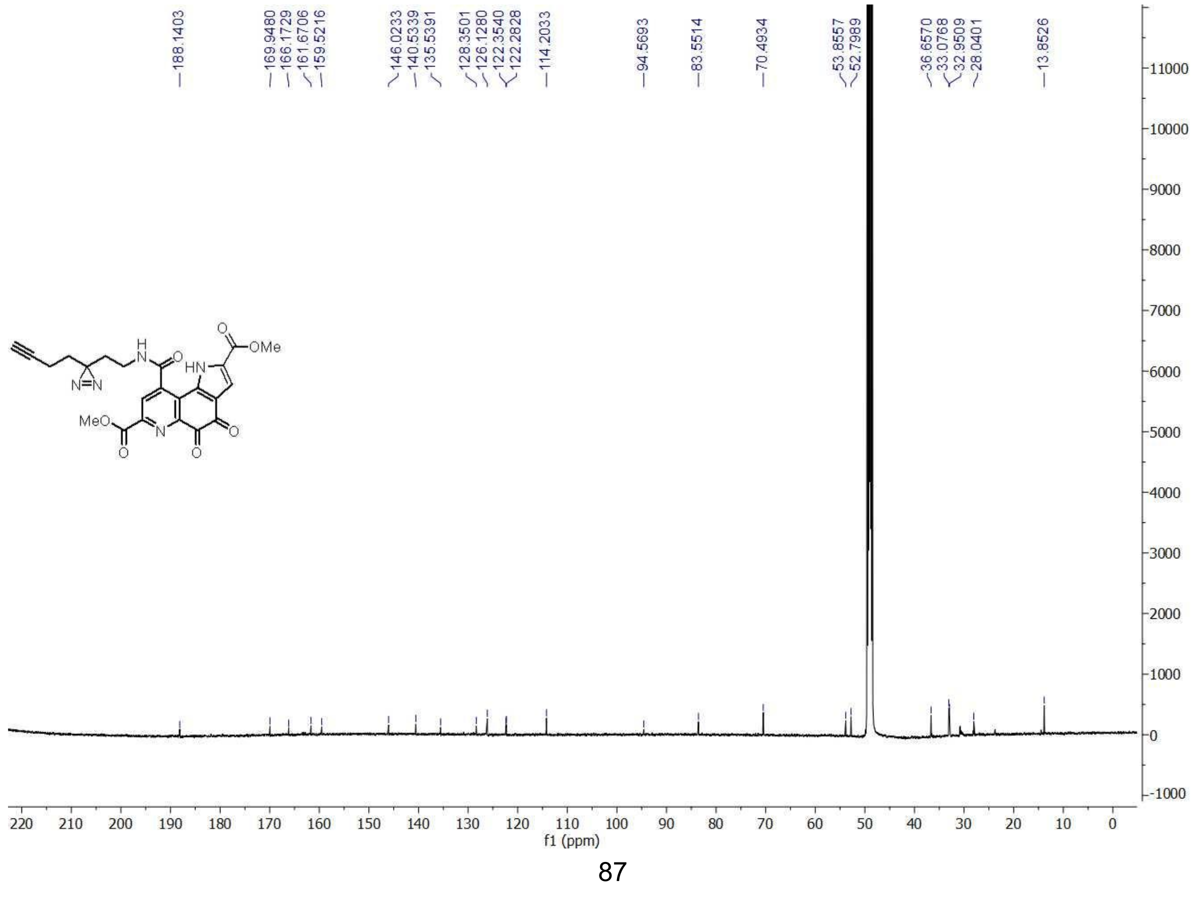

^{1}H NMR of **9**

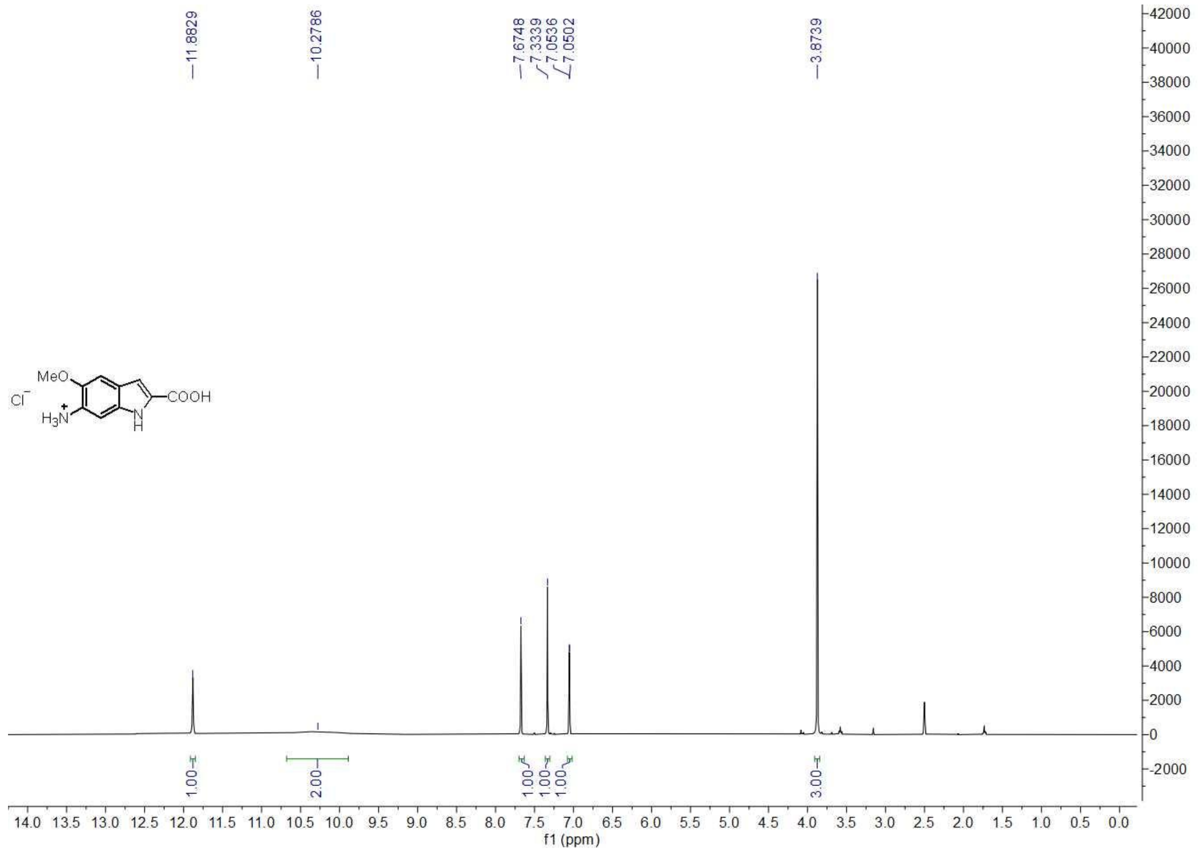

^{13}C NMR of **9**

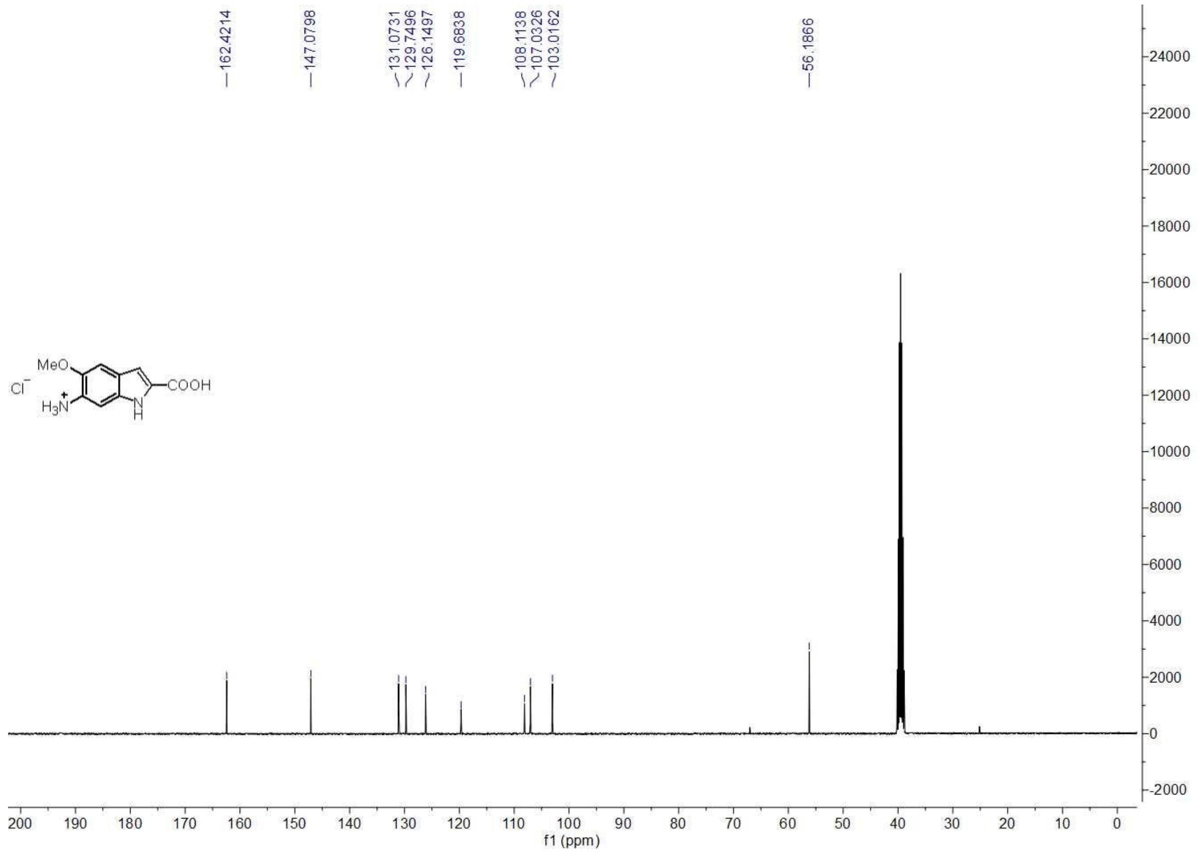

^{1}H NMR of **10**

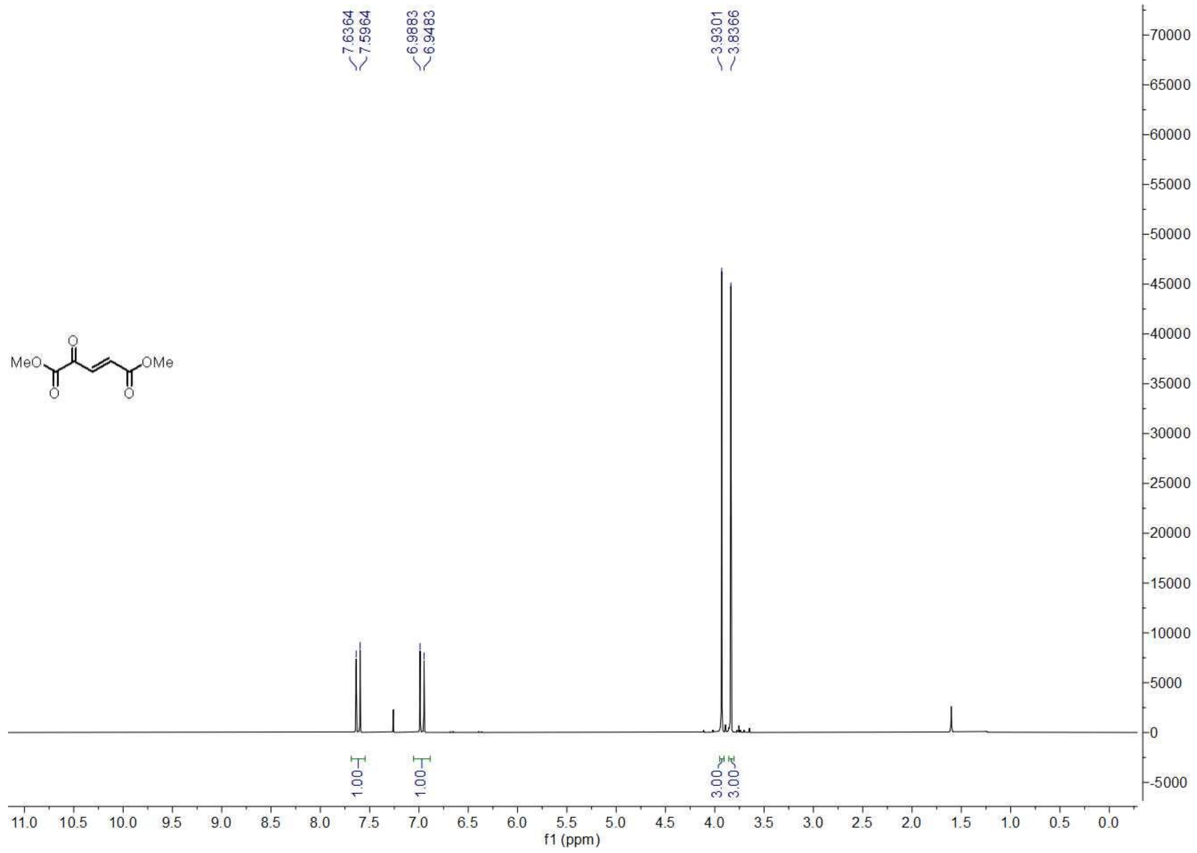

^{13}C NMR of **10**

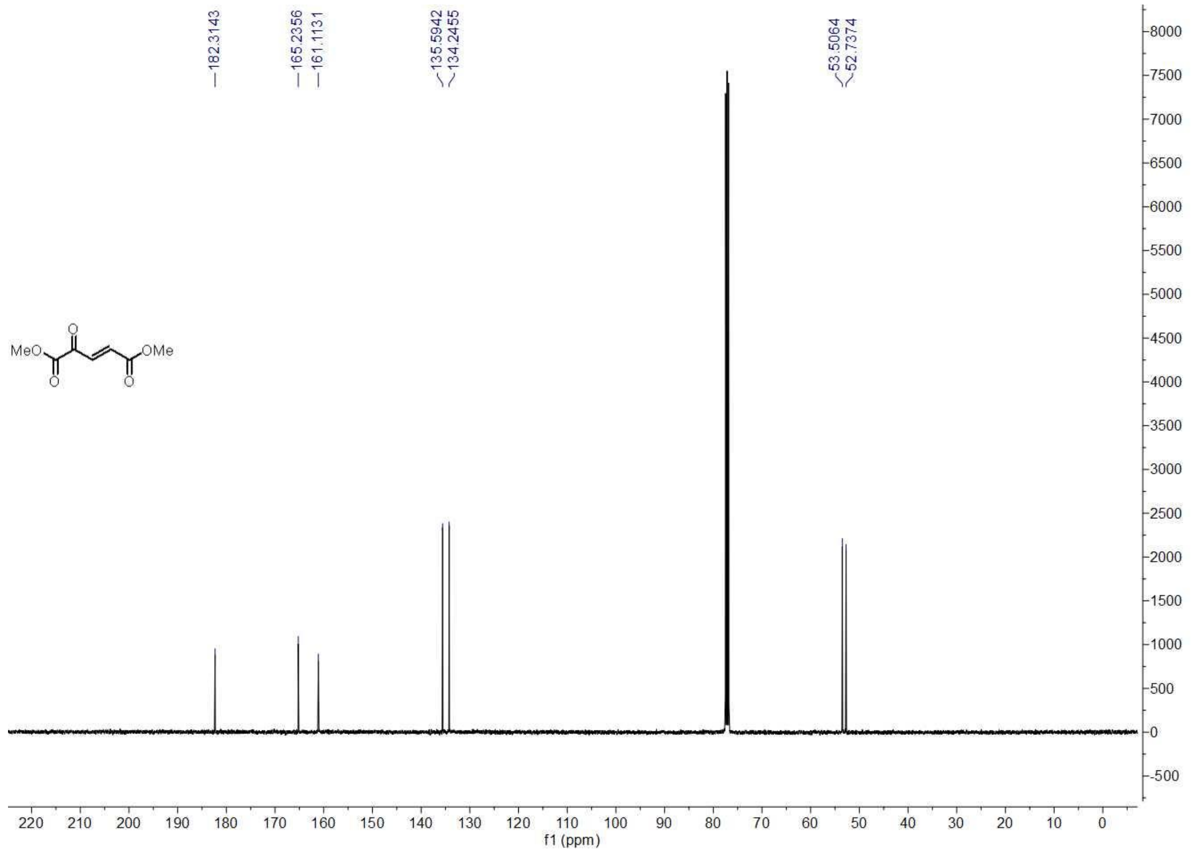

^{1}H NMR of **12**

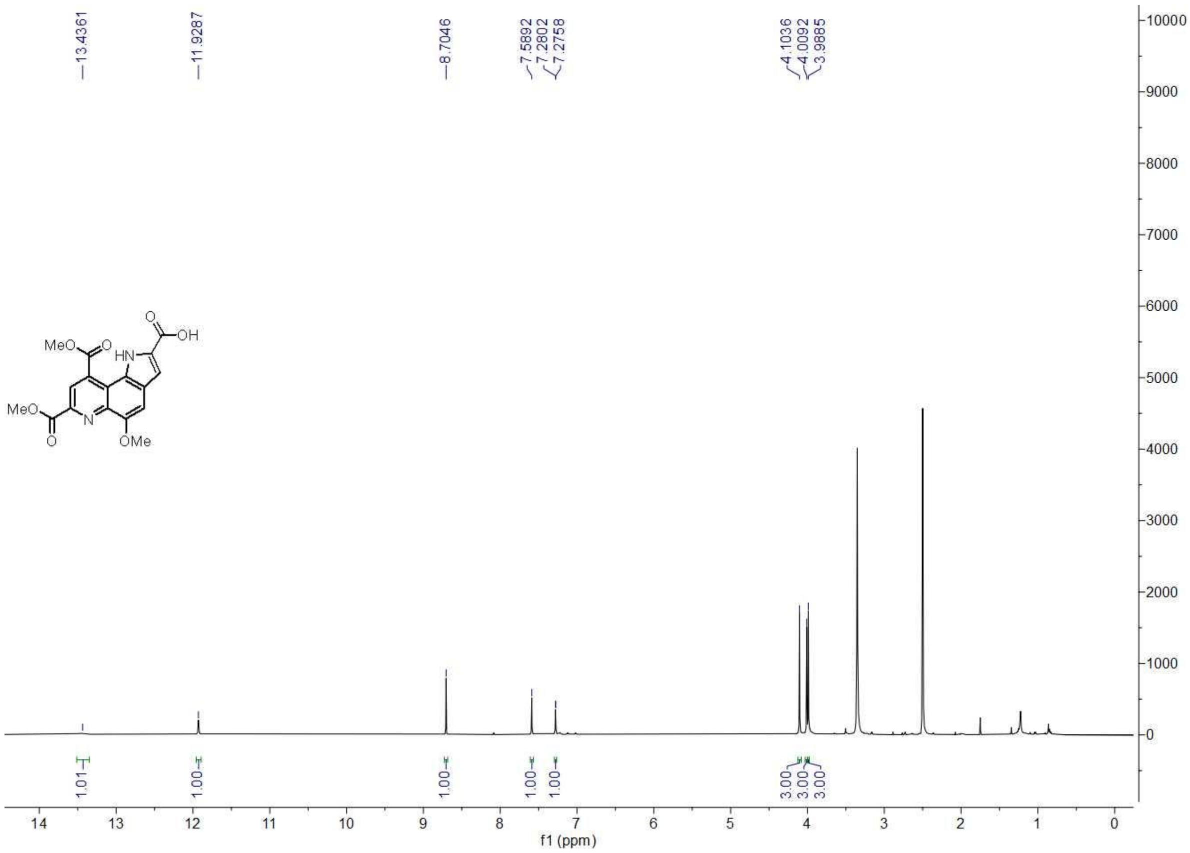

^{13}C NMR of **12**

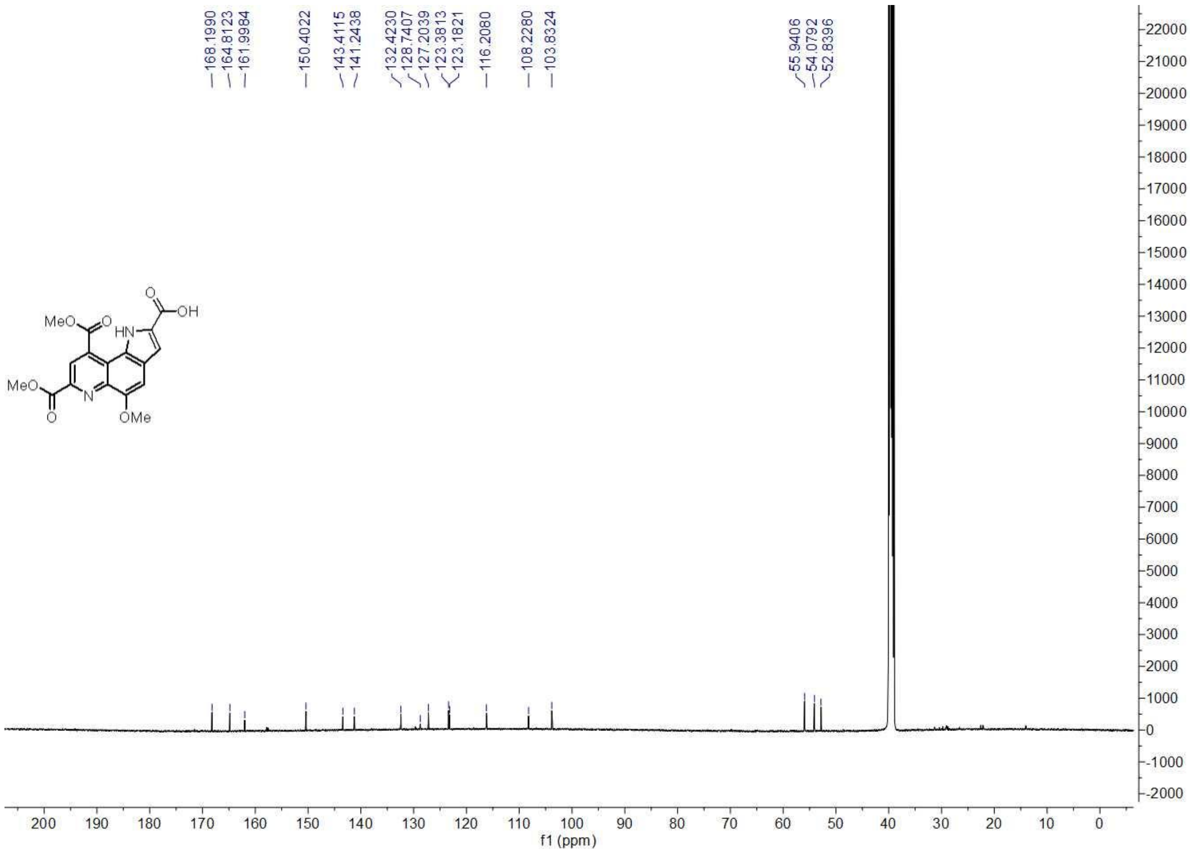

^{1}H NMR of **13**

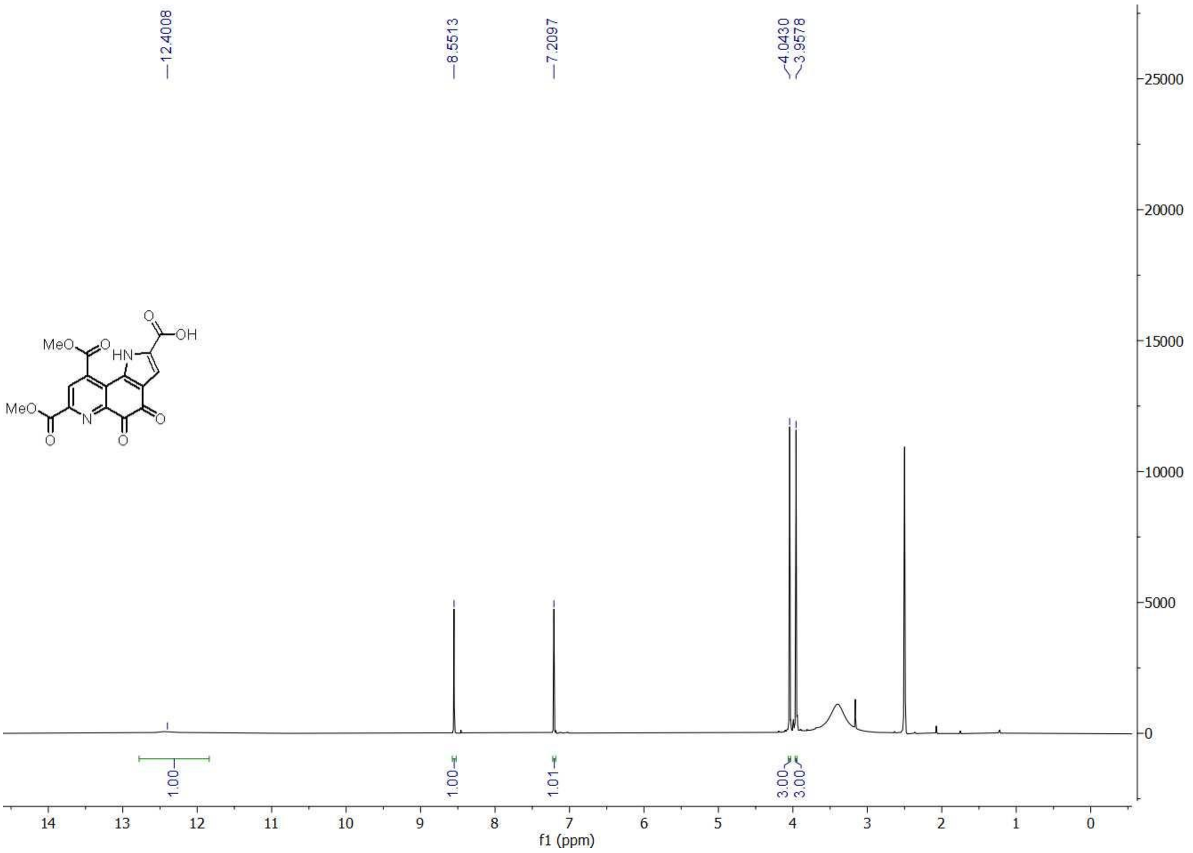

^{13}C NMR of **13**

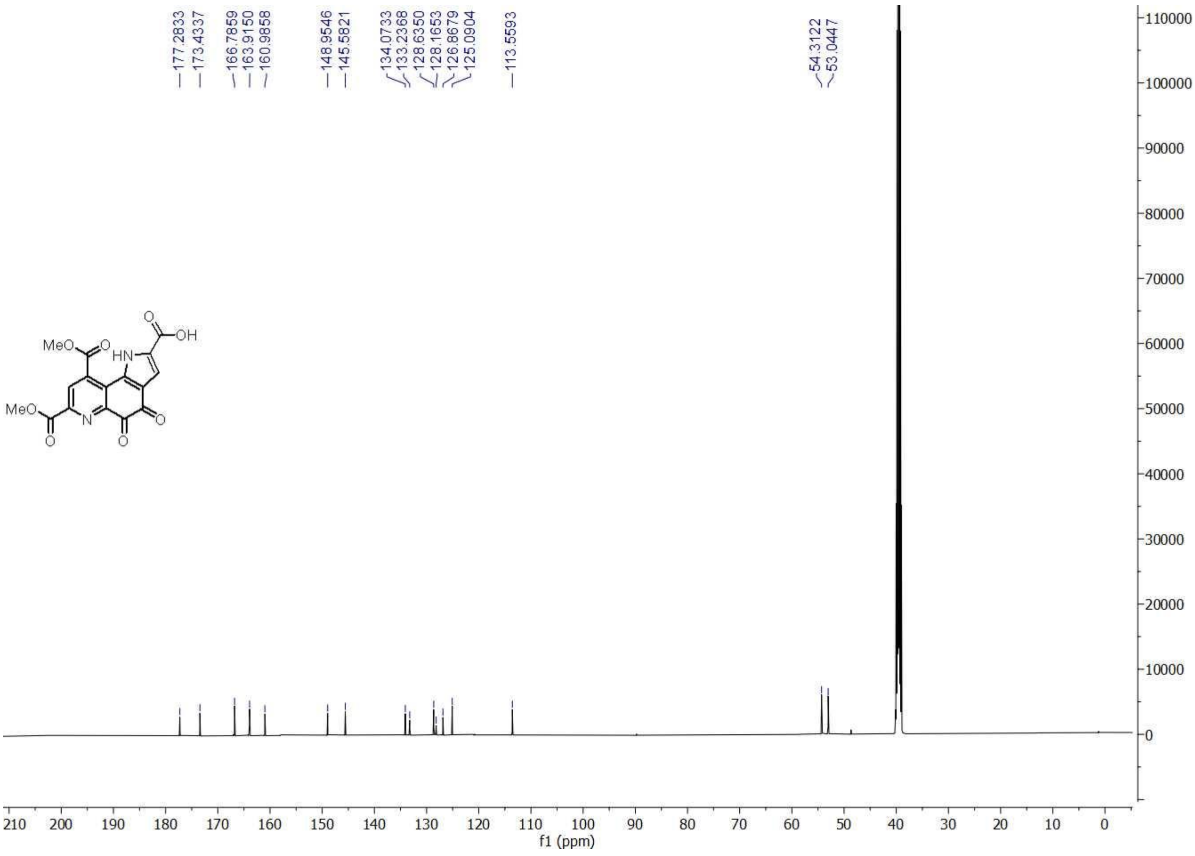

^{1}H NMR of **PQQ8**

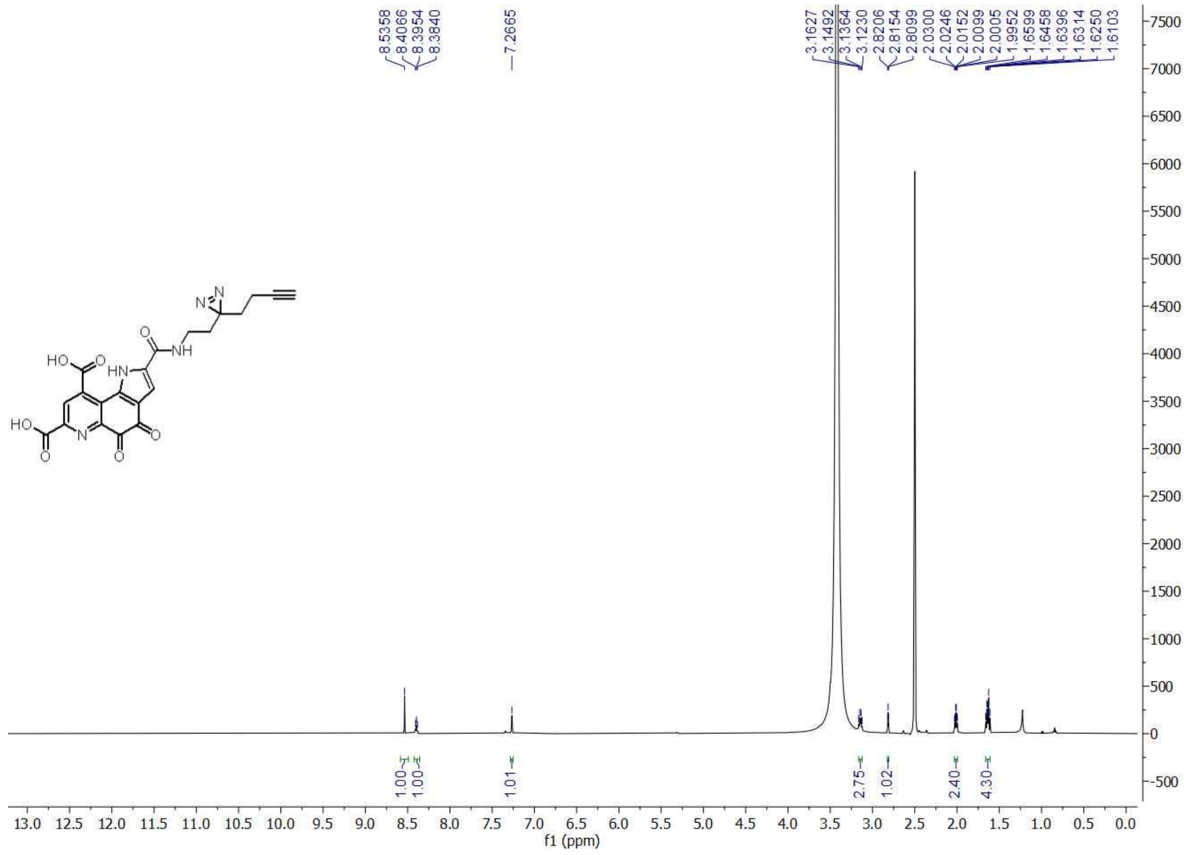

^{13}C NMR of **PQQ8**

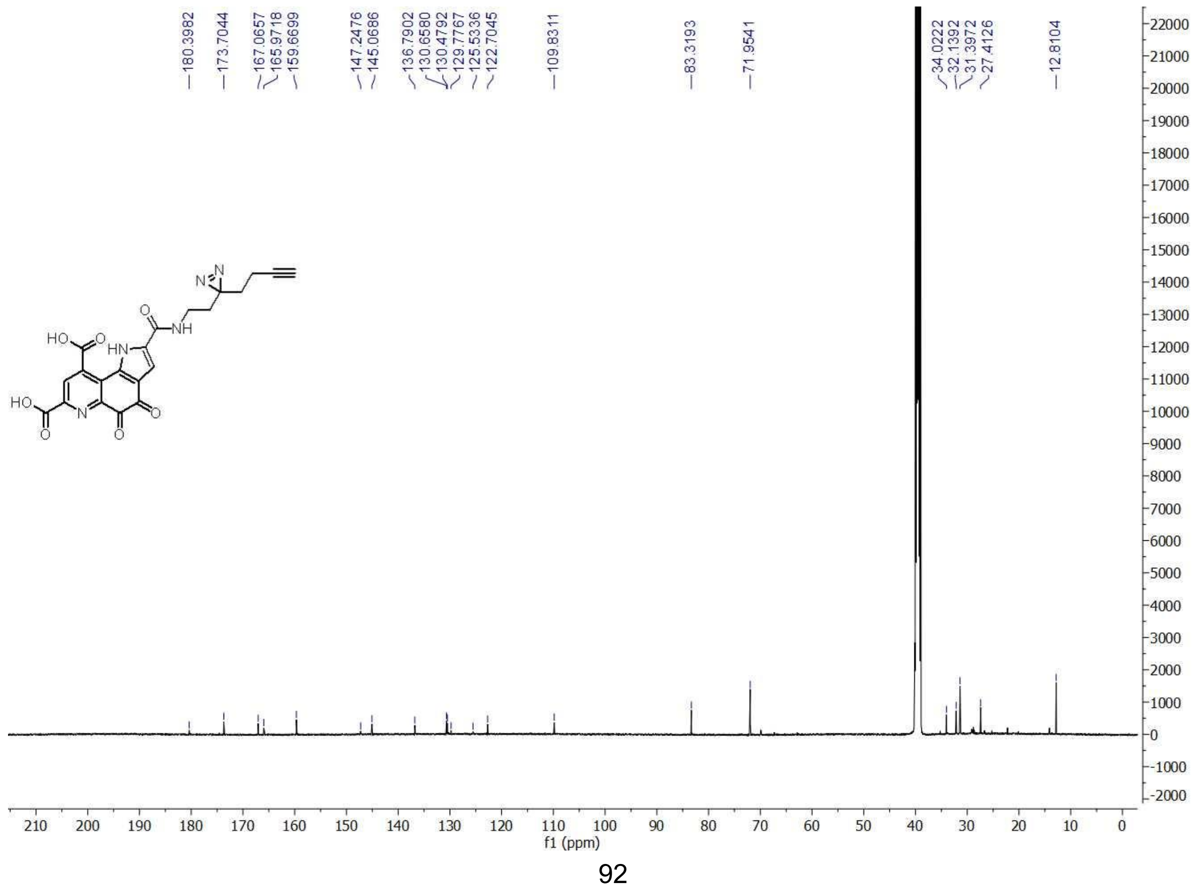

^{1}H NMR of **PhotoX**

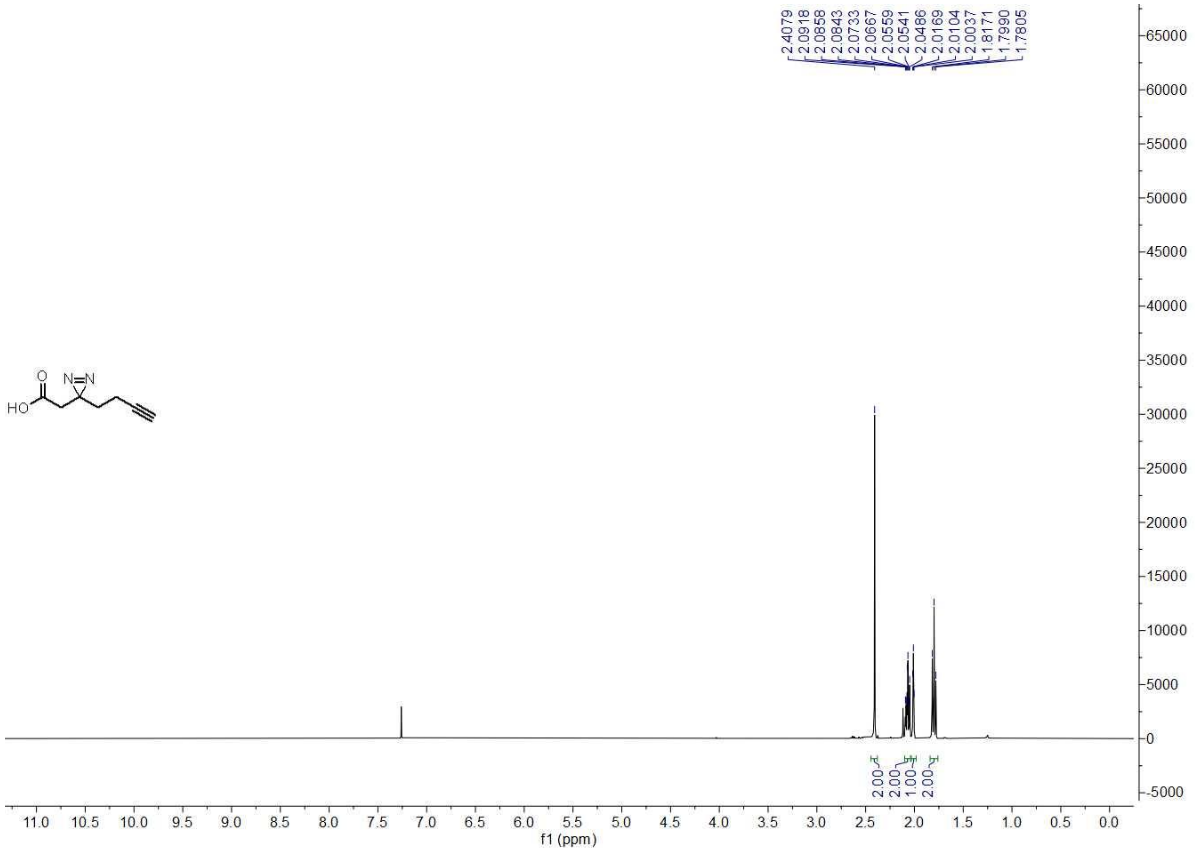

^{13}C NMR of **PhotoX**

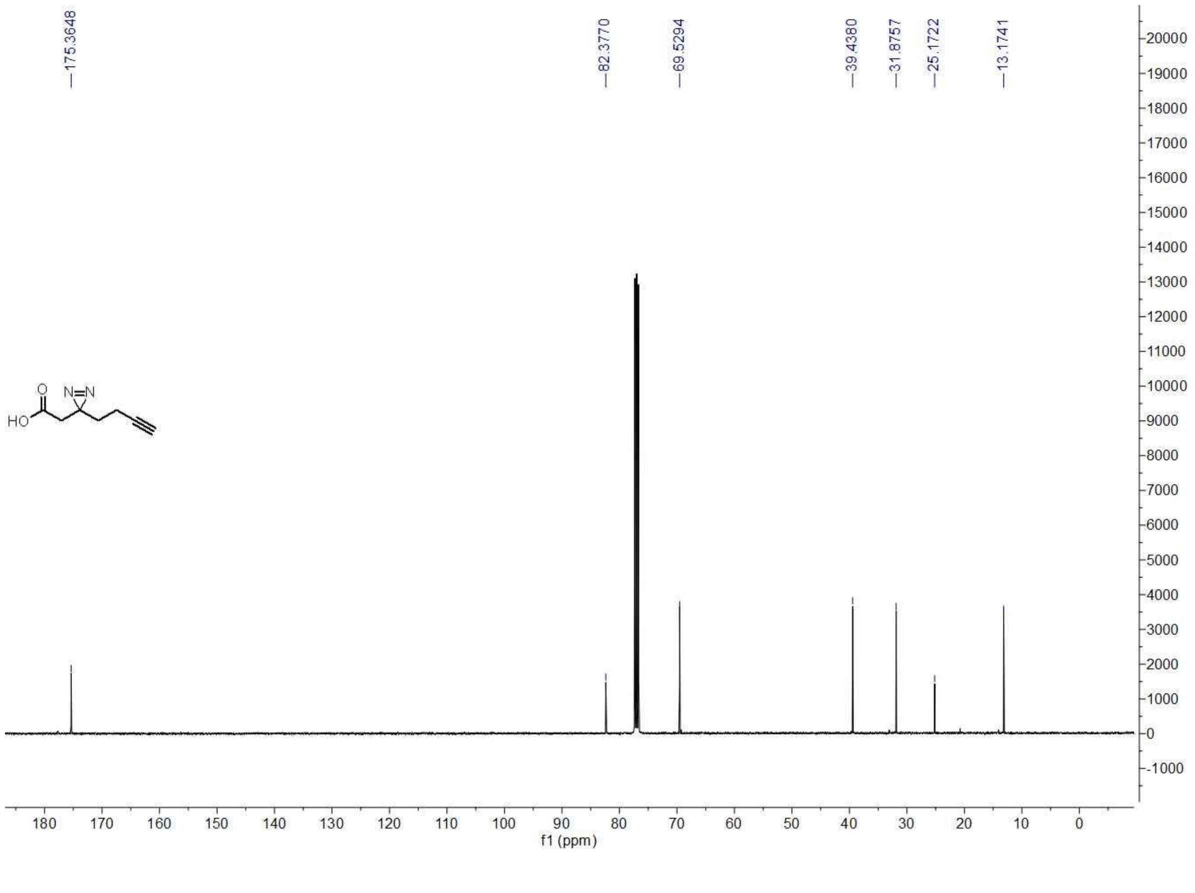

5. References

(1) Vetsova, V. A.; Fisher, K. R.; Lumpe, H.; Schafer, A.; Schneider, E. K.; Weis, P.; Daumann, L. J. Pyrroloquinoline quinone aza-crown ether complexes as biomimetics for lanthanide and calcium dependent alcohol dehydrogenases. *Chem. - Eur. J.* **2021**, *27*, 10087-10098.
(2) Mure, M.; Nii, K.; Inoue, T.; Itoh, S.; Ohshiro, Y. The reaction of coenzyme PQQ with hydrazines. *J. Chem. Soc. Perkin Trans. 2* **1990**, 315-320.
(3) Walko, M.; Hewitt, E.; Radford, S. E.; Wilson, A. J. Design and synthesis of cysteine-specific labels for photo-crosslinking studies. *RSC Adv.* **2019**, *9*, 7610-7614.
(4) Urakami, T.; Oda, M.; Narita, R. T., Akinori; Iesaka, H. Oxazopyrroloquinoline deivatives and use thereof. EP0670321 (A1), **1995**.
(5) Carrigan, C. N.; Bartlett, R. D.; Esslinger, C. S.; Cybulski, K. A.; Tongcharoensirikul, P.; Bridges, R. J.; Thompson, C. M. Synthesis and in vitro pharmacology of substituted quinoline-2,4-dicarboxylic acids as inhibitors of vesicular glutamate transport. *J. Med. Chem.* **2002**, *45*, 2260-2276.
(6) Sicker, D.; Stehfest, E.; Wilde, H.; Martin, P. Ein alternativer zugang zum PQQ-triester. *Helvetica. Chimica. Acta.* **1996**, *79*, 658-662.
(7) Martin, P.; Steiner, E.; Auer, K.; Winkler, T. Zur herstellung von PQQ in kg-mengen. *Helvetica. Chimica. Acta.* **1993**, *76*, 1667-1673.
(8) Kirsch, V. C.; Orgler, C.; Braig, S.; Jeremias, I.; Auerbach, D.; Muller, R.; Vollmar, A. M.; Sieber, S. A. The cytotoxic natural product vioprolide a targets nucleolar protein 14, which Is essential for ribosome biogenesis. *Angew. Chem., Int. Ed.* **2020**, *59*, 1595-1600.
(9) Seitchik, J. L.; Peeler, J. C.; Taylor, M. T.; Blackman, M. L.; Rhoads, T. W.; Cooley, R. B.; Refakis, C.; Fox, J. M.; Mehl, R. A. Genetically encoded tetrazine amino acid directs rapid site-specific in vivo bioorthogonal ligation with trans-cyclooctenes. *J. Am. Chem. Soc.* **2012**, *134*, 2898-2901.
(10) Rugbjerg, P.; Feist, A. M.; Sommer, M. O. A. Enhanced metabolite productivity of *Escherichia coli* adapted to glucose M9 minimal medium. *Front. Bioeng. Biotechnol.* **2018**, *6*, 166.
(11) Demichev, V.; Messner, C. B.; Vernardis, S. I.; Lilley, K. S.; Ralser, M. DIA-NN: neural networks and interference correction enable deep proteome coverage in high throughput. *Nat. Methods* **2020**, *17*, 41-44.
(12) Demichev, V.; Szyrwiel, L.; Yu, F.; Teo, G. C.; Rosenberger, G.; Niewienda, A.; Ludwig, D.; Decker, J.; Kaspar-Schoenefeld, S.; Lilley, K. S.; Mulleder, M.; Nesvizhskii, A. I.; Ralser, M. dia-PASEF data analysis using FragPipe and DIA-NN for deep proteomics of low sample amounts. *Nat. Commun.* **2022**, *13*, 3944.
(13) Frankenfield, A. M.; Ni, J.; Ahmed, M.; Hao, L. Protein contaminants matter: building universal protein contaminant libraries for DDA and DIA proteomics. *J. Proteome Res.* **2022**, *21*, 2104-2113.
(14) Tyanova, S.; Temu, T.; Sinitcyn, P.; Carlson, A.; Hein, M. Y.; Geiger, T.; Mann, M.; Cox, J. The Perseus computational platform for comprehensive analysis of (prote)omics data. *Nat. Methods* **2016**, *13*, 731-740.
(15) Cox, J.; Mann, M. 1D and 2D annotation enrichment: a statistical method integrating quantitative proteomics with complementary high-throughput data. *BMC Bioinformatics* **2012**, *13*, S12.
(16) Kong, A. T.; Leprevost, F. V.; Avtonomov, D. M.; Mellacheruvu, D.; Nesvizhskii, A. I. MSFragger: ultrafast and comprehensive peptide identification in mass spectrometry-based proteomics. *Nat. Methods* **2017**, *14*, 513-520.

(17) da Veiga Leprevost, F.; Haynes, S. E.; Avtonomov, D. M.; Chang, H. Y.; Shanmugam, A. K.; Mellacheruvu, D.; Kong, A. T.; Nesvizhskii, A. I. Philosopher: a versatile toolkit for shotgun proteomics data analysis. *Nat. Methods* **2020**, *17*, 869-870.
(18) Yu, F.; Haynes, S. E.; Nesvizhskii, A. I. IonQuant enables accurate and sensitive label-free quantification with FDR-controlled match-between-runs. *Mol. Cell. Proteomics* **2021**, *20*, 100077.
(19) Li, K.; Vaudel, M.; Zhang, B.; Ren, Y.; Wen, B. PDV: an integrative proteomics data viewer. *Bioinformatics* **2019**, *35*, 1249-1251.
(20) Yu, F.; Teo, G. C.; Kong, A. T.; Haynes, S. E.; Avtonomov, D. M.; Geiszler, D. J.; Nesvizhskii, A. I. Identification of modified peptides using localization-aware open search. *Nat. Commun.* **2020**, *11*, 4065.
(21) Zanon, P. R. A.; Yu, F.; Musacchio, P.; Lewald, L.; Zollo, M.; Krauskopf, K.; Mrdović, D.; Raunft, P.; Maher, T. E.; Cigler, M.; Chang, C. J.; Lang, K.; Toste, F. D.; Nesvizhskii, A. I.; Hacker, S. M. Profiling the proteome wide selectivity of diverse electrophiles. *ChemRxiv.* **2021**.
(22) Cox, J.; Mann, M. MaxQuant enables high peptide identification rates, individualized p.p.b.-range mass accuracies and proteome-wide protein quantification. *Nat. Biotechnol.* **2008**, *26*, 1367-1372.
(23) Cox, J.; Hein, M. Y.; Luber, C. A.; Paron, I.; Nagaraj, N.; Mann, M. Accurate proteome-wide label-free quantification by delayed normalization and maximal peptide ratio extraction, termed MaxLFQ. *Mol. Cell. Proteomics* **2014**, *13*, 2513-2526.
(24) Cox, J.; Neuhauser, N.; Michalski, A.; Scheltema, R. A.; Olsen, J. V.; Mann, M. Andromeda: a peptide search engine integrated into the MaxQuant environment. *J. Proteome Res.* **2011**, *10*, 1794-1805.
(25) Hartley, J. L.; Temple, G. F.; Brasch, M. A. DNA cloning using In vitro site-specific recombination. *Genome Res.* **2000**, *10*, 1788-1795.
(26) Gibson, D. G. Enzymatic Assembly of Overlapping DNA Fragments. In *Methods in Enzymology*; Academic Press, 2011.
(27) Wehrmann, M.; Elsayed, E. M.; Köbbing, S.; Bendz, L.; Lepak, A.; Schwabe, J.; Wierckx, N.; Bange, G.; Klebensberger, J. Engineered PQQ-dependent alcohol dehydrogenase for the oxidation of 5-(hydroxymethyl)furoic acid. *ACS Catal.* **2020**, *10*, 7836-7842.
(28) Southall, S. M.; Doel, J. J.; Richardson, D. J.; Oubrie, A. Soluble aldose sugar dehydrogenase from Escherichia coli: a highly exposed active site conferring broad substrate specificity. *J. Biol. Chem.* **2006**, *281*, 30650-30659.
(29) Jahn, B.; Jonasson, N. S. W.; Hu, H.; Singer, H.; Pol, A.; Good, N. M.; den Camp, H. J. M. O.; Martinez-Gomez, N. C.; Daumann, L. J. Understanding the chemistry of the artificial electron acceptors PES, PMS, DCPIP and Wurster's Blue in methanol dehydrogenase assays. *J. Biol. Inorg. Chem.* **2020**, *25*, 199-212.
(30) Marty, M. T.; Baldwin, A. J.; Marklund, E. G.; Hochberg, G. K. A.; Benesch, J. L. P.; Robinson, C. V. Bayesian deconvolution of mass and ion mobility spectra: from binary interactions to polydisperse ensembles. *Anal. Chem.* **2015**, *87*, 4370-4376.
(31) Wohlwend, J.; Corso, G.; Passaro, S.; Getz, N.; Reveiz, M.; Leidal, K.; Swiderski, W.; Atkinson, L.; Portnoi, T.; Chinn, I.; Silterra, J.; Jaakkola, T.; Barzilay, R. Boltz-1 democratizing biomolecular interaction modeling. *bioRxiv* **2025**.
(32) Passaro, S.; Corso, G.; Wohlwend, J.; Reveiz, M.; Thaler, S.; Somnath, V. R.; Getz, N.; Portnoi, T.; Roy, J.; Stark, H.; Kwabi-Addo, D.; Beaini, D.; Jaakkola, T.; Barzilay, R. Boltz-2: towards accurate and efficient binding affinity prediction. *bioRxiv* **2025**.
